# **Functional characteristics of membrane vesicles produced by** ***Streptococcus mitis***

**DOI:** 10.1080/20002297.2025.2557962

**Published:** 2025-09-23

**Authors:** Airi Matsumoto, Yuichi Oogai, Haruka Kurashige, Tomoko Sumitomo, Atsushi Tabata, Masanobu Nakata

**Affiliations:** a Department of Oral Microbiology, Kagoshima University Graduate School of Medical and Dental Sciences, Kagoshima, Japan; b Department of Periodontology, Kagoshima University Graduate School of Medical and Dental Sciences, Kagoshima, Japan; c Department of Oral Microbiology, Graduate School of Biomedical Sciences, Tokushima University, Tokushima, Japan; d Department of Bioengineering, Division of Bioscience and Bioindustry, Graduate School of Technology, Industrial and Social Sciences, Tokushima University Graduate School, Tokushima, Japan

**Keywords:** *Streptococcus pneumoniae*, *Streptococcus mitis*, mitis group streptococci, membrane vesicle, oral commensal streptococci, *Aggregatibacter actinomycetemcomitans*, *Streptococcus mutans*, pathogenic bacteria

## Abstract

**Objective:**

Mitis group streptococci (MGS) are the predominant oral bacteria that cause bacteremia and infective endocarditis. Although membrane vesicle (MV) secretion has been reported in *Streptococcus pneumoniae* among MGSs, comprehensive studies using various streptococcal species are limited. We aimed to determine whether MGS species produce MVs and to examine their biological functions.

**Materials and methods:**

MVs were isolated from MGS cultures using density gradient ultracentrifugation. The particle sizes of MVs were measured, and proteins in MVs were identified by liquid-chromatography tandem mass spectrometry analysis. Effects of MVs on host cells and oral pathogenic bacteria were investigated.

**Results:**

MV production was confirmed in *Streptococcus mitis* strains NCTC12261, Nm-65, and Nm-76, with particle diameters ranging from 100 to 120 nm. The MVs contained numerous cytoplasmic proteins. The MVs showed internalization into alveolar epithelial cells and induced the production of multiple cytokines, including TNF-*α*, IL-8, IL-6, IL-1β, and IL-10, in macrophages while suppressing phagocytic activity. In neutrophil-differentiated cells, MVs induced IL-8 but not TNF-*α* production. MVs from *S. pneumoniae* TIGR4 and *S. mitis* Nm-65 inhibited biofilm formation of *Aggregatibacter actinomycetemcomitans*.

**Conclusions:**

MVs play crucial roles in MGS survival strategies through immune modulation and interspecies competition, contributing to their pathogenicity and host-pathogen interactions.

## Key messages

• Membrane vesicles (MVs) play crucial roles in mitis group streptococci (MGS) survival strategies through immune modulation and interspecies competition, contributing to their pathogenicity and host-pathogen interactions.
• This study provides new insights into the functions of MVs produced by MGS, particularly *S. mitis*.
• These findings expand our understanding of streptococcal pathogenicity and open new avenues for the development of preventive measures, including novel MV-based vaccines and anti-biofilm agents.

## Introduction

Among oral commensal bacteria, certain species are associated with the development of dental caries and periodontitis, whereas beneficial commensals can support host immunity and promote their own survival in the densely populated oral niche [1–4]. Oral streptococci, as early colonizers, compete with other bacteria through hydrogen peroxide production, bacteriocin secretion, and competitive binding to pellicle [5,6]. Initial colonization patterns influence the selection of secondary colonizers, thereby shaping the entire oral ecosystem. The influence of oral streptococci extends beyond the oral cavity. For instance, studies have shown that oral streptococci can migrate to the lungs of individuals with cystic fibrosis and that their presence correlates with improved lung function [7,8]. Both commensal and pathogenic streptococci from the oral cavity can enter the bloodstream, leading to systemic infections, such as infective endocarditis [9,10].

*Streptococcus mitis*, a key component of normal oral and pharyngeal microbiota, attaches to human oral mucosal cells and salivary pellicles via adhesins on their surfaces [2]. *S. mitis*, which belongs to the mitis group streptococci (MGS), is also classified as a member of the viridans group streptococci based on its *α*-hemolytic characteristic (incomplete hemolysis). Although it typically exhibits low virulence, it can act as an opportunistic pathogen, causing bacteremia and infective endocarditis on entering the bloodstream [2,11]. Particularly, cancer patients with neutropenia are particularly vulnerable to bacteremia caused by viridans group streptococci, with *S. mitis* being the predominant causative agent [12]. These infections can lead to severe complications, such as viridans streptococcal shock syndrome, which has a high mortality rate ranging from 40 to 100% among pediatric patients [12,13]. The pathogenic potential of *S. mitis* is further supported by the presence of genes encoding various virulence factors homologous to those found in *Streptococcus pneumoniae*, including pneumolysin (Ply) and autolysin [14].

In gram-negative bacteria, membrane vesicles (MVs), termed outer membrane vesicles (OMVs), mediate diverse functions, including horizontal gene transfer, nutrient acquisition, and induction of host inflammation [15]. These OMVs contain outer membrane components, whereas MVs from Gram-positive bacteria originate from the cell membrane, resulting in distinct cargo compositions. Recent studies have shown that *S. pneumoniae* TIGR4, a member of MGS, secretes abundant MVs containing both cytoplasmic and transmembrane proteins, along with multiple virulence factors [16−18]. These MVs exhibit diverse biological activities, including protective activity against *S. pneumoniae* infection in murine models and induction of inflammatory cytokines (interleukin [IL]-6, IL-8, and tumor necrosis factor-alpha [TNF-*α*]) in human dendritic cells [16−19]. MVs are promising candidates for cell-free vaccines based on their immunogenicity and biological stability.

Despite advances in the understanding of pneumococcal MVs, whether commensal oral streptococci, such as *S. mitis* produce MVs remains unknown, as do the functional roles of these MVs in both commensal and pathogenic contexts. Based on the close genetic relatedness between *S. pneumoniae* and *S. mitis*, along with the reported production of MVs in *S. pneumoniae*, we hypothesized that *S. mitis* also produces biologically active MVs that influence host-bacterial and interbacterial interactions.

This study aimed to confirm and characterize MV production by *S. mitis*, identify the protein components of these MVs, determine their effects on human cells, and examine their roles in modulating their interactions with pathogenic oral bacteria. The resulting data will provide critical insights into the role of *S. mitis* as both a commensal organism and an opportunistic pathogen.

## Materials and methods

### **Bacterial strains and culture conditions**

In this study, *S. pneumoniae* TIGR4, *S. mitis* SK142, NCTC12261, Nm-65, Nm-76, *Streptococcus salivarius* HHT, *Streptococcus sanguinis* SK36, *Streptococcus gordonii* SK7, *Streptococcus mutans* UA159, and *Aggregatibacter actinomycetemcomitans* (*Aa*) ATCC29523 were used (Table 1). All strains were cultured at 37 °C in a humidified 5% CO_2_ incubator. Growth media included brain-heart infusion (BHI) broth or BHI agar plates (Becton Dickinson and Company, Franklin Lakes, NJ, USA) for *S. pneumoniae*, *S. mitis*, *S. salivarius*, *S. sanguinis* and *S. gordonii*; Trypticase Soy Broth (TSB; Becton Dickinson and Company) for *S. mutans*; and *Aggregatibacter actinomycetemcomitans* growth medium (AAGM) [20] for *Aa*. AAGM consists of 2.5% TSB, 0.6% yeast extract (Nacalai Tesque, Kyoto, Japan), 1% glucose [FUJIFILM Wako Pure Chemical Corporation (FUJIFILM Wako), Osaka, Japan], and 0.4% sodium bicarbonate (Nacalai Tesque).

**Table 1.** Bacterial strains used in this study.

Species	Strains	Characteristics	Sources
*Streptococcus pneumoniae*	TIGR4	Isolated from human blood	[21]
*Streptococcus mitis*	NCTC12261	Type strain	Public Health England
	Nm-65	Isolated from oral cavity of a patient with Kawasaki disease	[22]
	Nm-76	Isolated from oral cavity of a patient with Kawasaki disease	[23]
	SK142	Type strain	M. Killian
*Streptococcus sanguinis*	SK36	Isolated from human dental plaque	[24]
*Streptococcus gordonii*	SK7	Unknown	M. Killian
*Streptococcus salivarius*	HHT	Isolated from human oral flora	[25]
*Streptococcus mutans*	UA159	Isolated from child with active caries (serotype c)	[26]
*Aggregatibacter actinomycetemcomitans*	ATCC29523	Serotype a	[27]

### **MV isolation and purification**

*Streptococcus* strains were cultured overnight at 37 °C in BHI medium in a 5% CO_2_ atmosphere and harvested by centrifugation (3,562 × *g*, 30 min, 4 °C). Culture supernatants were filtered through a 0.22-μm pore size polyvinylidene difluoride filter (Millipore, Massachusetts, USA) and ultracentrifuged (120,000 × *g*, 1.5 h, 4 °C) to precipitate the vesicles. The pellet was rinsed with phosphate-buffered saline (PBS) and resuspended in PBS. The crude membrane vesicle fractions were further purified by density gradient ultracentrifugation using OptiPrep density gradient medium (Sigma-Aldrich, St. Louis, USA) according to a previously reported method [18]. The MV solution was adjusted to 50% (w/v) OptiPrep in a total volume of 0.8 mL. A layer of 3.0 mL of 30% (w/v) OptiPrep adjusted with PBS was added, followed by a layer of 1.0 mL of 5% (w/v) OptiPrep to create a density gradient. The gradient was centrifuged at 155,000 × *g* for 3 h at 4 °C, and the top 1.4 mL (containing the MVs) was collected. The MVs were washed with PBS by ultracentrifugation (155,000 × *g* for 1.5 h at 4 °C), and protein concentrations were measured using a Pierce Bicinchoninic Acid Protein Assay kit (Thermo Fisher Scientific, Massachusetts, USA) with BSA (FUJIFILM Wako) as the standard. Then, 10 µL of the purified MV solution was mixed with two-fold concentrated Laemmli sample buffer with reducing, heated at 95 °C for 10 min, and separated by sodium dodecyl sulfate-polyacrylamide gel electrophoresis (SDS-PAGE). Subsequently, the separated proteins were subjected to Coomassie brilliant blue (CBB) staining. Staining was performed in 25% isopropanol, 0.06% CBB G-250 (Nacalai Tesque), and 10% acetic acid for 1 h. After staining, the gels were de-stained in a mixture of 5% acetic acid and 10% methanol for 3 h. The particle size of the purified MVs was determined using a zeta potential and particle size analyzer (ELSZ-2000ZS, Otsuka Electronics, Osaka, Japan).

### **Observation and quantification of MVs**

Ten microliters of the MV solution was dropped onto a collodion-coated copper grid (Nisshin-EM, Tokyo, Japan) and incubated for 10 min. The grid was stained by inverting onto a drop of 2% sodium phosphotungstate for 5 s and blotted using filter paper. The MVs were visualized by transmission electron microscopy (TEM; H-7600, HITACHI, Tokyo, Japan) operating at 80 kV. MV yield was calculated by counting particles in TEM images and expressed as MVs per colony forming unit (CFU) based on the original bacterial cell count.

### **Proteomic analysis in MVs**

To analyze the proteome, 20 µL protein-solubilized sample was mixed with 2 µL of 1 M Tris-HCl (pH 7.5), followed by reduction and alkylation using 10 mM dithiothreitol and 55 mM iodoacetamide, respectively, at room temperature for 30 min. To the processing sample, we added 300 µL of 100 mM Tris-HCl (pH 7.5), 20 µL of Sera-Mag Carboxylate-Modified Magnetic SpeedBeads (GE Healthcare, Chicago, IL), and a final concentration of 70% acetonitrile for 10 min [28]. The supernatant was discarded, and the beads were washed with 70% ethanol, followed by 100% acetonitrile. The beads were then resuspended in 50 µL of 50 mM triethylammonium bicarbonate, and proteolytic digestion was performed by adding 1 µL of trypsin (Thermo Scientific) and incubating overnight at 37 °C. The digested proteins were eluted by adding 50 µL of 2% dimethyl sulfoxide (DMSO). The eluted peptides were acidified with 10 µL of 10% formic acid. Dried peptides were resuspended in 3% acetonitrile containing 0.1% trifluoroacetic acid.

Approximately 200 ng of each peptide was analyzed using an Orbitrap Fusion mass spectrometer equipped with a nanoelectrospray ion source coupled to an EASY-nLC 1200 UHPLC system (Thermo Fisher Scientific) [29].

Raw data were processed using Proteome Discoverer version 2.5 (Thermo Fisher Scientific) and Sequest HT search engine. Peptides and proteins were filtered at a false discovery rate of 1% using a percolator node.

### **Analysis of MV binding and internalization by human cells**

A549 human alveolar epithelial cells (RCB3677, RIKEN BRC, Ibaraki, Japan) were maintained in Eagle's minimum essential medium (EMEM; FUJIFILM Wako) supplemented with 10% (v/v) fetal bovine serum (FBS; Thermo Fisher Scientific), 100 U/mL penicillin G (Nacalai Tesque), and 0.1 mg/mL streptomycin sulfate (Nacalai Tesque). A549 cells were seeded into 24-well plates at 2.0 × 10^5^ cells per well and incubated overnight at 37 °C in a 5% CO_2_ atmosphere. Prior to treatment with MVs, the culture medium was replaced with serum-free EMEM. MVs were fluorescently labeled with 0.02 mM PKH67 (Green Fluorescent Cell Linker Midi Kit; Sigma-Aldrich). The labeled MVs (10.0 µg) were added to the cells for overnight incubation at 37 °C. After washing with PBS, the cells were fixed with 4% paraformaldehyde (Cosmo Bio, Tokyo, Japan) for 20 min and permeabilized with PBS containing 0.5% Triton X-100 (Nacalai Tesque) for 5 min. The actin cytoskeleton was visualized using Acti-stain^TM^ 555 phalloidin (Cytoskeleton, Inc., Denver, USA) and incubated for 30 min. Coverslips (Matsunami Glass, Osaka, Japan) were mounted using Fluoroshield Mounting Medium with 4′,6-diamidino-2-phenylindole (ImmunoBioScience Corp., Washington, USA). Cell morphology and fluorescence were analyzed using an LSM900 confocal microscope (ZEISS, Oberkochen, Germany).

### **Phagocytosis assay using human THP-1 macrophages**

THP-1 human monocytes derived from acute monocytic leukemia (RCB1189; RIKEN BRC) were maintained in EMEM supplemented with 10% (v/v) FBS, 100 U/mL penicillin G, and 0.1 mg/mL streptomycin sulfate. THP-1 cells were seeded in 24-well plates at 2.0 × 10^5^ cells/well and differentiated with 50 ng/mL of phorbol 12-myristate 13-acetate (PMA; Sigma-Aldrich) for 48 h. Human plasma was obtained from a healthy volunteer and preincubated with MVs (10.0 µg) from TIGR4, NCTC12261, Nm-65, or Nm-76 at 37 °C for 2 h. After preincubation, this MV-treated plasma (final concentration: 20%) was incubated with *S. mitis* SK142 at 37 °C for 30 min to opsonize the bacteria. Following opsonization, the opsonized *S. mitis* was added to the THP-1 macrophages at a multiplicity of infection (MOI) of 50 and incubated at 37 °C for 1.5 h. Cells were washed with PBS to remove non-associated bacteria. To assess phagocytosis, extracellular bacteria were killed by adding EMEM supplemented with gentamicin (750 µg/mL; FUJIFILM Wako) and incubating at 37 °C for 15 min. Cells were lysed with 1% saponin (FUJIFILM Wako) at 37 °C for 15 min. The lysates were serially diluted in PBS and plated on BHI agar plates for bacterial enumeration.

### **Quantification of cytokine production**

THP-1 macrophages (1.0 × 10^5^ cells/well) were incubated with MVs (10 µg) or PBS at 37 °C for 4 h in a 5% CO_2_ atmosphere. Cytokine levels (interferon gamma [IFN-*γ*], IL-1α, IL-1β, IL-2, IL-6, IL-8, IL-10, IL-12, IL-18, and TNF-*α*) in the cell-free culture supernatants were quantified using a MILLIPLEX MAP Human Cytokine–Chemokine Panel (Millipore). Samples were assayed according to the manufacturer's instructions. Briefly, standards and samples were loaded into 96-well plates, followed by addition of antibody-immobilized beads. Plates were incubated at 4 °C overnight with gentle shaking and then washed three times with wash buffer. Detection antibodies were then added to the wells and incubated at room temperature for 1 h. Finally, streptavidin-phycoerythrin was added to the wells and incubated at room temperature for 30 min. Plates were washed gently three times with wash buffer, and beads were resuspended in sheath fluid for analysis on a MAGPIX (Merck Millipore). A standard curve was generated using the xPONENT software (Luminex Corporation, Austin, TX, USA) with a five-parameter logistic curve for each mediator ranging from 10,000 to 3.2 pg/mL, and cytokine concentrations were calculated accordingly. For neutrophil-differentiated HL-60 cells, we used enzyme-linked immunosorbent assays (ELISAs) to measure TNF-*α* and IL-8 levels in culture supernatants using the DuoSet Kit (R&D Systems, Minnesota, USA) according to the manufacturer's instructions.

### **Evaluation of oral bacteria growth kinetics**

*S. mutans* and *Aa* overnight cultures were diluted 1000-fold in their respective media, and 80 µL of each dilution was mixed with 20 µL of MVs (2.0 or 10.0 µg in PBS) or PBS into a 96-well plates, and bacterial growth was monitored by measuring optical density at 660 nm (OD_660_) during incubation at 37 °C in a 5% CO_2_ atmosphere.

### **Evaluation of oral bacterial biofilm formation**

*S. mutans* and *Aa* were cultured overnight at 37 °C in a 5% CO_2_ atmosphere. The bacterial cultures were adjusted to an OD_660_ of 1.0 in their respective media (BHI supplemented with 1% sucrose for *S. mutans*, AAGM for *Aa*). Precisely10 µL of the culture was mixed with 90 µL of various concentrations of MVs (0.2, 1.0, 2.0, or 10.0 µg in PBS) or PBS in 96-well flatbottom plates, and the plates were incubated overnight at 37 °C in a 5% CO_2_ atmosphere. After incubation, the culture medium was removed, and the wells were washed thrice with distilled water using a shaker (TOKYO RIKAKIKA, Tokyo, Japan; 200 rpm, 10 min per wash) to remove planktonic bacteria. The biofilms were fixed with 100% methanol for 10 min and stained with 0.1% crystal violet for 10 min. Following three additional washes with water, the dye was eluted with 100 µL of ethanol from the biofilms for 30 min. Biofilm mass was quantified by measuring the OD_590_ of the elutions.

### **Statistical analyses**

Data are shown from one representative experiment out of the same experiment performed three or more times. Statistical analyses were performed using Dunnett's or Tukey's test. All data were obtained using triplicate samples and presented as the mean ± standard deviation. Differences were considered significant when the probability value was less than 5% (*p *< 0.05).

## Results

### **Selective production of MVs among oral streptococcal species**

First, we investigated whether various oral streptococcal species produce MVs. Our analysis using CBB staining and protein concentration measurements confirmed MV production in four of the eight tested strains (Table 2): the previously reported *S. pneumoniae* TIGR4 strain and three *S. mitis* strains (NCTC12261, Nm-65, and Nm-76). In contrast, no MV production was detected in *S. mitis* SK142, *S. sanguinis* ATCC10556, *S. gordonii* SK7, or *S. salivarius* HHT, suggesting species- and strain-specific variations in the ability to produce MVs. In MV-non-producing strains, protein concentrations were below the detection limit, and no bands were detected by CBB staining.

**Table 2.** MV production status of strains examined in this study.

Species	Strain	MV production
*Streptococcus pneumoniae*	TIGR4	+
*Streptococcus mitis*	NCTC12261	+
	Nm-65	+
	Nm-76	+
	SK142	−
*Streptococcus sanguinis*	SK36	−
*Streptococcus gordonii*	SK7	−
*Streptococcus salivarius*	HHT	−

**Figure 1.**
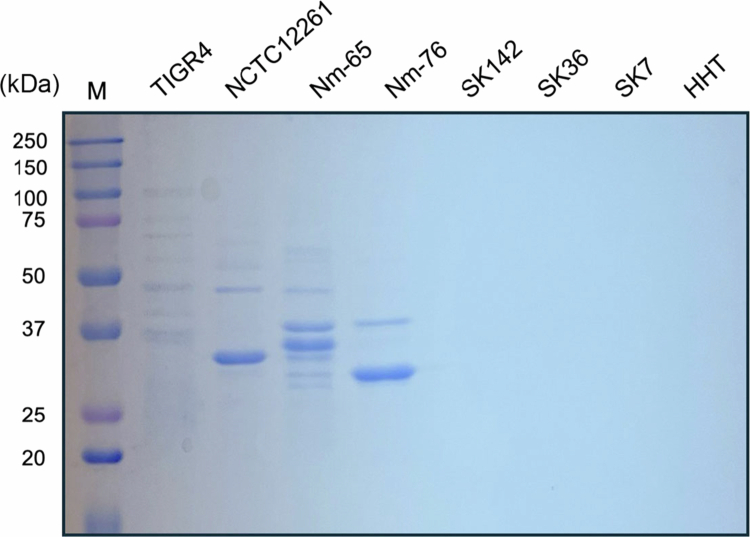
Protein profiles of streptococcal MVs. SDS-PAGE and CBB staining of MVs purified from culture supernatants of *S. pneumoniae* TIGR4, *S. mitis* NCTC12261, *S. mitis* Nm-65, *S. mitis* Nm-76, *S. mitis* SK142, *S. sanguinis* SK36, *S. gordonii* SK7, and *S. salivarius* HHT. Samples were loaded at 10 µg protein/lane where protein concentration could be determined using the bicinchoninic acid assay, or 10 µL/lane when protein could not be detected. M, molecular weight markers.

To characterize the protein content of the purified MVs, MVs were separated on a polyacrylamide gel and stained with CBB staining (Figure 1). Protein profiles revealed distinct band patterns among MV-producing strains, indicating diverse protein compositions. Notably, these MV protein patterns differed significantly from those observed in the whole-cell extracts and culture supernatant fractions of the corresponding strains (Supplementary Figure 1), suggesting that MV formation involves selective protein incorporation.

**Figure 2.**
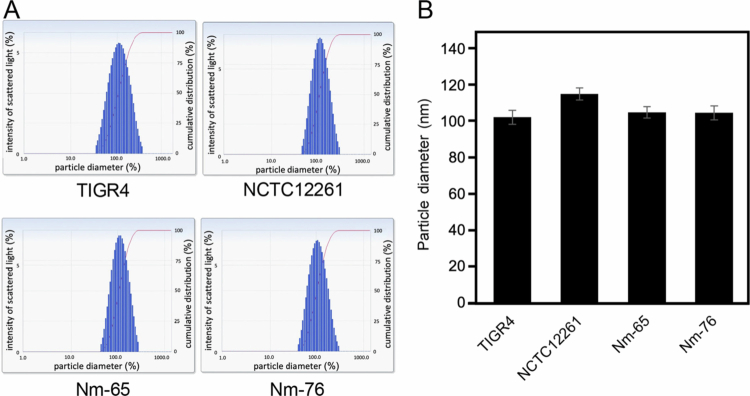
Size distribution of streptococcal MVs. The MV sizes were analyzed using a zeta-potential and particle size analyzer. Average diameters were as follows: TIGR4, 102.6 nm; NCTC12261, 115.5 nm; Nm-65, 105.4 nm; and Nm-76, 105.0 nm (A, B). Data represent mean ± standard deviation from three independent experiments.

**Figure 3.**
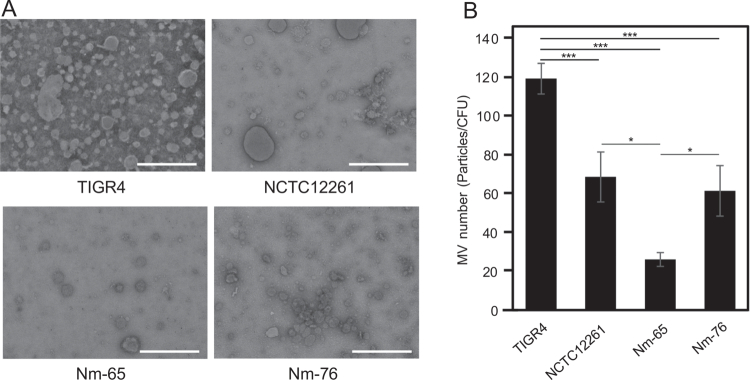
Observation and quantification of MVs. (A) Transmission electron microscopy images of MVs. Scale bar, 500 nm. (B) MV yield was calculated based on bacterial cell counts and expressed as MVs/CFU. Data represent mean ± standard deviation of triplicate samples (*p < 0.05, ***p < 0.005, Tukey's test). Data shown are representative of three independent experiments.

Further characterization of the purified MVs using particle size analysis showed relatively consistent dimensions among all MV-producing strains. Measurements using a zeta potential and particle size analyzer showed a single peak, indicating the production of uniform vesicles (Figure 2A). The average diameters were 102.6 nm for TIGR4, 115.5 nm for NCTC12261, 105.4 nm for Nm-65, and 105.0 nm for Nm-76 (Figure 2B). Consistent with these results, TEM observations revealed vesicles of similar size (Figure 3A). The size consistency observed among the tested strains, with diameters ranging from 100 to 120 nm, suggests the presence of conserved mechanisms governing MV biogenesis in these streptococcal species. Additionally, MV yield per CFU was calculated as follows: 119.5 MVs/CFU for TIGR4, 68.9 MVs/CFU for NCTC12261, 26.4 MVs/CFU for Nm-65, and 61.7 MVs/CFU for Nm-76 (Figure 3B).

### **Comprehensive proteomic characterization of streptococcal MVs**

**Figure 4.**
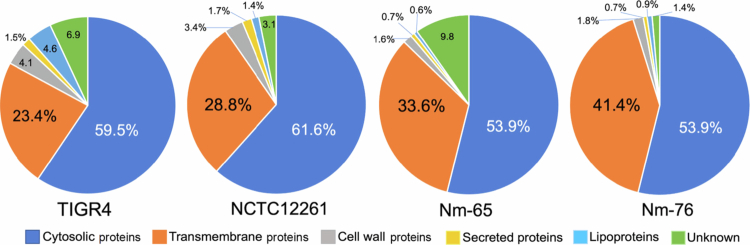
Subcellular localization of MV proteins. LC-MS/MS analysis of delipidized trypsin-digested MV. Data were analyzed using proteome discoverer version 2.4. The distribution of proteins identified by subcellular localization is shown.

To characterize the protein composition of streptococcal MVs, we performed liquid chromatography-tandem mass spectrometry analysis of the purified MVs. As shown in Figure 4, the MVs predominantly contained cytoplasmic components in all the analyzed strains (TIGR4, 59.5%; NCTC12261, 61.6%; Nm-65, 53.9%; Nm-76, 53.9%). The remaining composition included proteins with transmembrane proteins (TIGR4, 23.4%; NCTC12261, 28.8%; Nm-65, 33.6%; Nm-76, 41.4%), lipoproteins (TIGR4, 4.6%; NCTC12261, 1.4%; Nm-65, 0.6%; Nm-76, 0.9%), cell wall proteins consisting of proteins with choline-binding domains or LPXTG motif (TIGR4, 4.1%; NCTC12261, 3.4%; Nm-65, 1.6%; Nm-76, 1.8%), and secreted proteins (TIGR4, 1.5%; NCTC12261, 1.7%; Nm-65, 0.7%; Nm-76, 0.7%) (Figure 3).

Proteomic analysis identified 659 proteins in MVs from *S. pneumoniae* TIGR4 (Supplementary Table 1), including known membrane proteins, such as the sialic acid ABC transporter SatA [30] and pyruvate oxidase SpxB [31]. Ply, zinc metalloproteinase (Zmp) [32], and autolysin were also detected. MV formation occurred independently of Zmp expression, as demonstrated by the successful isolation of MVs from *zmpA*-deficient, *zmpB*-deficient, and *zmpC*-deficient mutants (data not shown).

*S. mitis* strains NCTC12261, Nm-65, and Nm-76 contained 706, 809, and 440 proteins in their MVs, respectively (Supplementary Tables 2−4). These MVs contain proteins with choline-binding domains that interact with choline residues in membrane-associated lipoteichoic acids. Additionally, we identified several major surface-associated virulence factors, including the cholesterol-dependent cytolysin, which is a homolog of Ply.

### **Cellular internalization of streptococcal MVs by epithelial cells**

**Figure 5.**
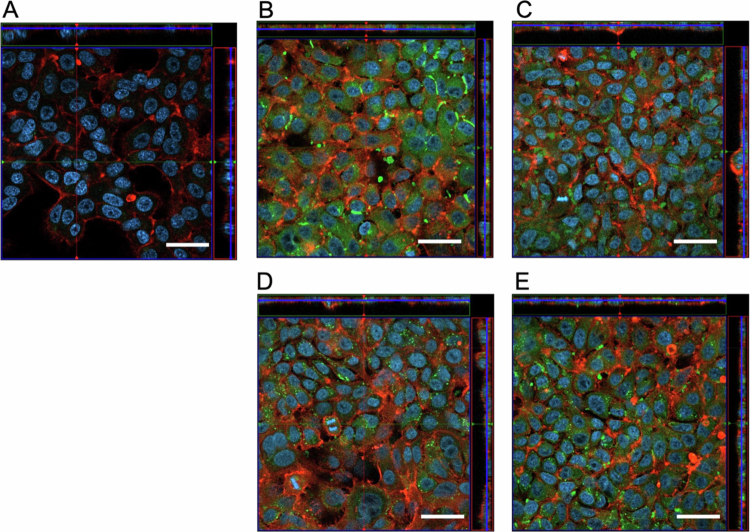
MV internalization by A549 cells. Confocal microscopy images of A549 cells after incubation with PKH67-labeled MVs (10 µｇ). Red: actin cytoskeleton; blue: nuclei; green: MVs (PKH67). A, control; B, TIGR4; C, NCTC12261; D, Nm-65; E, Nm-76. Scale bar, 20 µm.

To assess MV internalization by epithelial cells, the human alveolar epithelial cell line A549 was treated with PKH67-labeled MVs, and MV internalization was examined using confocal microscopy. All tested MVs (from TIGR4, NCTC12261, Nm-65, and Nm-76) exhibited strong fluorescence and were clearly detected within the actin-stained layer (red) of A549 cells (Figure 5). We confirmed the intracellular localization of MVs using z-stack images, demonstrating that the vesicles were internalized rather than merely attached to the cell surface. Although cellular uptake was observed, the microscopic examination revealed no cell detachment or cytotoxicity (Figure 5).

### **Inhibition of phagocytosis by streptococcal MVs**

**Figure 6.**
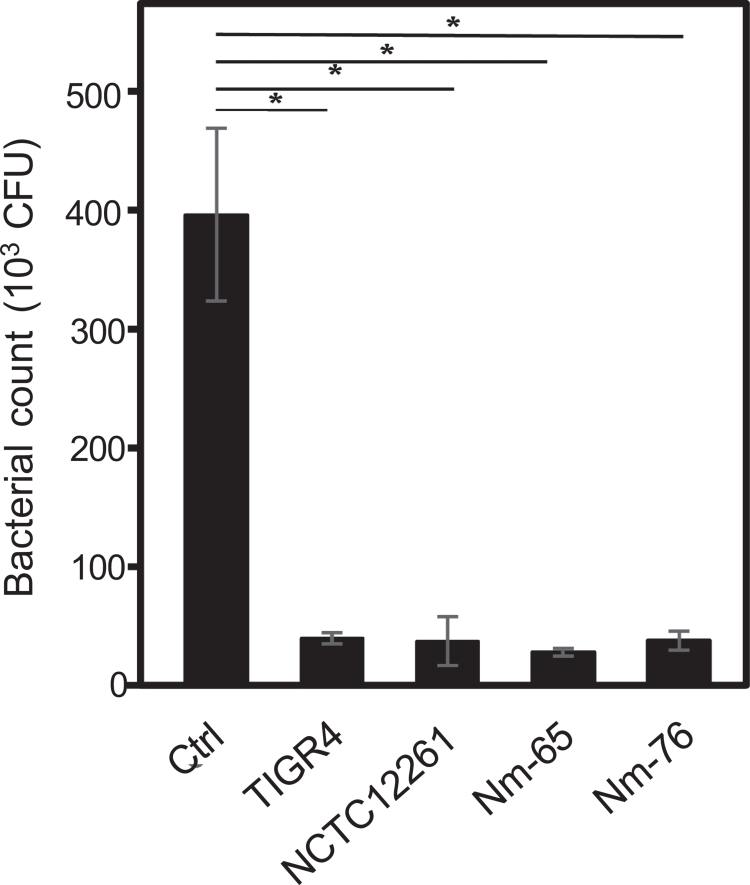
Effects of MVs on THP-1 cell phagocytosis. Phagocytic activity of PMA-differentiated THP-1 cells against *S. mitis* SK142 (MOI = 50) in the presence of MVs or PBS control (Ctrl). After gentamicin treatment to kill extracellular bacteria, the cells were lysed, and viable bacteria were enumerated on BHI agar. Data represent mean ± standard deviation from one representative experiment out of three independent experiments (*p < 0.05, Dunnett's test).

Next, we examined whether streptococcal MVs influence host immune cell function. To assess the effects of MVs on phagocytosis, human THP-1 macrophages were infected with *S. mitis* SK142 pre-incubated with either human plasma alone or human plasma containing MVs. As shown in Figure 6, MVs from both *S. pneumoniae* TIGR4 and *S. mitis* strains (NCTC12261, Nm-65, and Nm-76) significantly reduced the phagocytic activity by approximately 90.1%−93.0% compared to the control.

### **Differential cytokine responses to MVs in immune cells**

To determine whether *S. mitis* MVs could modulate host inflammatory responses similarly to *S. pneumoniae* TIGR4 MVs, we analyzed cytokine production in activated macrophages and neutrophils following MV exposure. Using a MILLIPLEX panel and THP-1 macrophage culture supernatants, we measured concentrations of multiple cytokines (IFN-*γ*, IL-1α, IL-1β, IL-2, IL-6, IL-8, IL-10, IL-12, IL-18, and TNF-*α*). For HL-60 neutrophil culture supernatants, we quantified IL-8 and TNF-*α* levels using ELISA.

As shown in Table 3, MV treatment of THP-1 macrophages tended to enhance the production of five cytokines (IL-1β, IL-6, IL-8, IL-10, and TNF-*α*). In contrast, no notable changes were observed in IL-1α, IL-2, IL-12, or IL-18 levels following MV exposure.

**Table 3.** MV-induced cytokine production in THP-1 macrophages.

Strain	IFN-γ	IL-1α	IL-1β	IL-2	IL-6	IL-8	IL-10	IL-12p70	IL-18	TNF-α
TIGR4	10.76	6.41	49.42	<0.64	188.28	7024.29	6565.98	0.41	2.42	15005.60
NCTC12261	8.50	7.09	39.94	<0.64	136.85	5750.71	5659.09	0.49	2.25	8680.67
Nm-65	6.11	3.98	27.67	<0.64	96.94	1810.15	3997.88	0.17	0.61	2344.20
Nm-76	8.95	5.36	25.90	<0.64	130.81	4762.15	4926.98	0.66	2.09	10320.74
Ctrl*	5.34	3.18	1.66	<0.64	<0.64	508.10	126.74	0.02	4.12	98.27

The values shown represent the mean of two independent experiments. Values denote cytokine concentrations in pg/ml.

* Ctrl, without MV.

**Figure 7.**
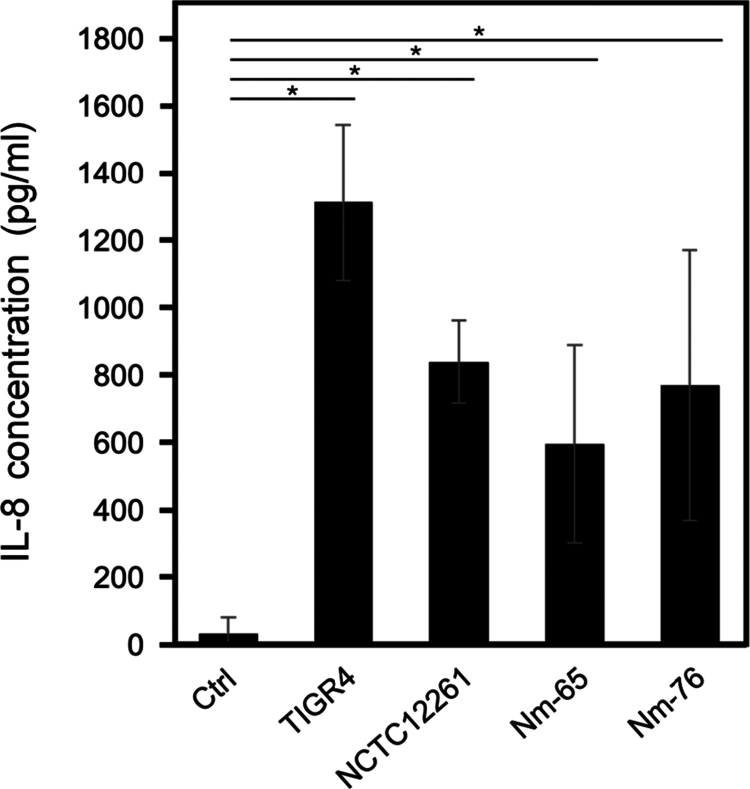
MV-induced IL-8 production by HL-60 neutrophils. IL-8 levels in the culture supernatants of DMSO-differentiated HL-60 cells after 4-h of treatment with MVs (10 µg) or PBS (Ctrl) were measured by enzyme-linked immunosorbent assay. Data represent mean ± standard deviation from one representative experiment out of three independent experiments (*p < 0.05, Dunnett's test).

In HL-60 neutrophils, we observed a different pattern of cytokine response to MV exposure (Figure 7). MVs significantly enhanced IL-8 production while TNF-*α* release remained below the detection limits. Although MVs from *S. mitis* induced slightly lower cytokine production in both macrophages and neutrophils than TIGR4 MVs, similar response patterns among all MV sources suggested a shared immunomodulatory mechanism.

### **Effect of MV on oral bacterial growth and biofilm formation**

Finally, we investigated whether streptococcal MVs could influence planktonic and biofilm growth of other bacterial species commonly found in the oral cavity. We focused on two species: *S. mutans*, a causative agent of dental caries, and *A. actinomycetemcomitans* (*Aa*), a periodontal pathogen.

**Figure 8.**
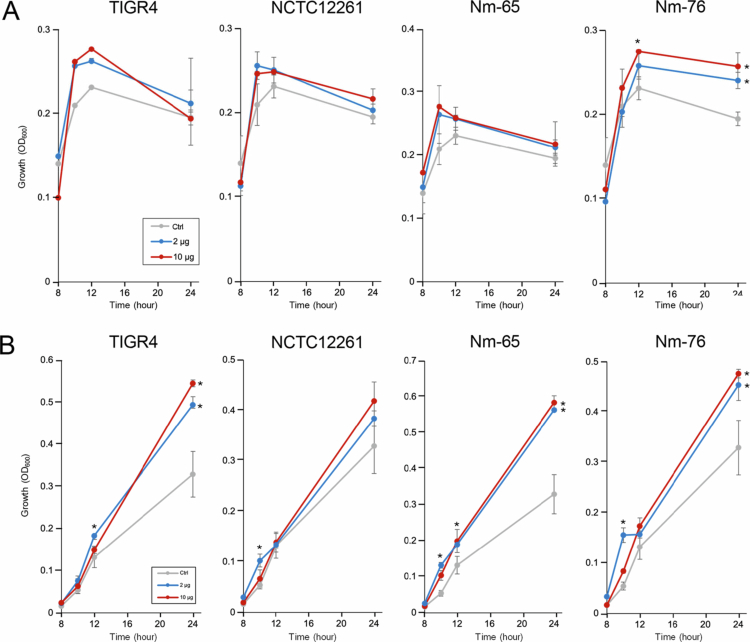
Effects of MVs on oral pathogen growth. Growth curves of (A) *S. mutans* and (B) *A. actinomycetemcomitans* cultured in the presence of MVs (2 or 10 µg) or PBS (Ctrl). Growth was monitored by OD_600_ measurements during incubation at 37 °C in a 5% CO_2_ atmosphere. Data are presented as mean ± standard deviation of triplicate wells from one representative experiment out of three independent experiments. Statistical significance was determined by Dunnett's test (*p < 0.05 versus Ctrl at the same time-points).

As shown in Figure 8, the MVs had distinctly different effects on the growth of these two species. For *S. mutans*, the addition of MVs from TIGR4, NCTC12261, and Nm-65 had no significant effect on bacterial growth compared to the PBS control; however, MVs from Nm-76 promoted bacterial growth after 12 h at the higher concentration (10 µg) and after 24 h at both concentrations (2 and 10 µg). In contrast, MVs from all four strains promoted *Aa* growth in a concentration-dependent manner, although the effect of NCTC12261 MVs was not statistically significant.

**Figure 9.**
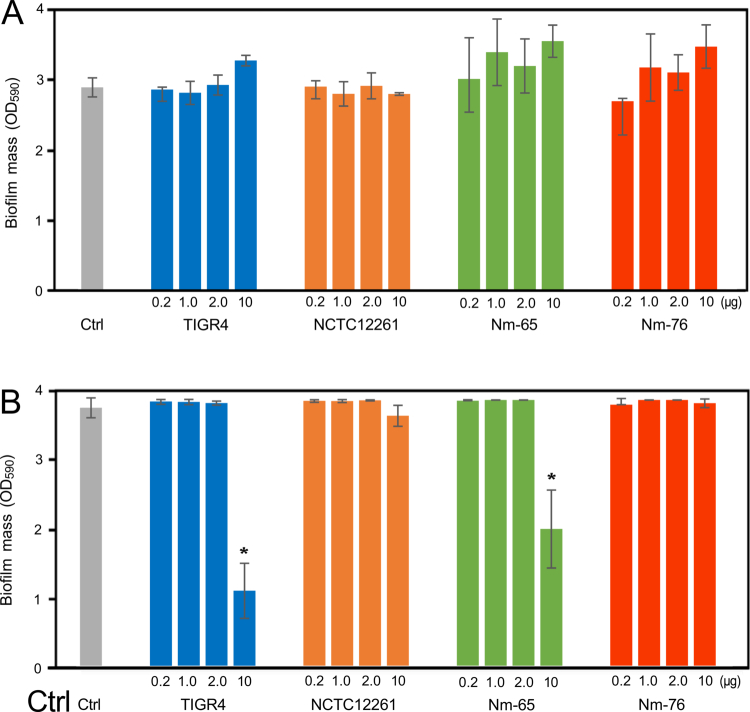
Effects of MVs on oral pathogen biofilm formation. Biofilm formation by (A) *S. mutans* and (B) *A. actinomycetemcomitans* in the presence of MVs (0.2, 1.0, 2.0, or 10.0 µg) or PBS (Ctrl). Bacterial suspensions (OD_660_ = 1.0) were incubated overnight at 37 °C in a 5% CO_2_ atmosphere. Crystal violet-stained biofilms were quantified using OD_590_ measurements. Data are presented as mean ± standard deviation of triplicate wells from one representative experiment out of three independent experiments. Statistical significance was determined by Dunnett's test (*p < 0.05 versus Ctrl at the same time-points).

We also observed species-specific effects of MVs on biofilm formation (Figure 9). *S. mutans* biofilm formation by *S. mutans* was largely unaffected by the presence of MVs from the four strains at all tested concentrations. In contrast, *Aa* biofilm formation was influenced in a strain-specific manner. Notably, MVs from TIGR4 and Nm-76 significantly reduced *Aa* biofilm formation at the highest concentration (10 µg) despite their growth-promoting effects. These data suggest a dissociation between the effects of these MVs on bacterial growth and biofilm formation, potentially involving specific anti-biofilm factors present in the MVs from these particular strains.

## Discussion

In this study, we investigated MV production by oral streptococci, focusing on *S. mitis*, and characterized the interactions between these MVs and host cells/oral bacteria. Our findings extend the current understanding of streptococcal MVs beyond that of previously reported *S. pneumoniae* MVs and provide new insights into the potential roles of these structures in both commensal and pathogenic contexts.

Among the eight examined *Streptococcus* strains, MV production was confirmed in four strains (Table 2), including three *S. mitis* strains (NCTC12261, Nm-65, and Nm-76). We observed strain-specific variation in the ability to produce MVs within the *S. mitis* species. MV contain numerous cytoplasmic proteins and, as previously reported [18,19], contain major pathogenic factors. The large number of proteins identified in this study is likely attributable to differences in the cutoff values used for protein selection. Two MV-producing strains, Nm-65 and Nm-76, contain a novel virulence factor homologous to Ply [22,23], whereas NCTC12261 lacks a Ply homolog. Thus, both Ply homolog-positive and -negative strains produced MVs, suggesting that Ply is not essential for MV production. This was further supported by the observation of MV production in a mitilysin (Ply homolog)-deficient strain of Nm-65 (data not shown). The inability to detect MVs in *S. mitis* SK142 and the other tested species (*S. sanguinis*, *S. gordonii*, and *S. salivarius*) raises questions regarding the genetic or environmental factors that may regulate MV production. *S. mitis* NCTC12261 and SK142 have been reported to have duplicate registrations due to their high genetic similarity [33], and it is possible that SK142 lost the ability to produce MVs during the subculture process. Previous studies have suggested that stress responses influence strain-specific MV production in *Cupriavidus necator* [34]. In addition, it has been reported that *S. sanguinis* produces MVs when cultured in chemically defined medium (CDM) supplemented with high glucose. Consistent with these findings [35,36], we also confirmed MV production by *S. sanguinis, S. gordonii*, and *S. salivarius* when grown in CDM containing 3.4% glucose (data not shown). Furthermore, MV production has also been reported in *S. salivarius* when cultured in BHI. However, this likely results from strain-specific differences [37]. Additional studies investigating multiple strains in different growth phases under various environmental conditions are warranted to fully understand the regulation of MV production by oral streptococci.

Our proteomic analysis revealed distinct protein compositions in purified MVs compared to those in whole-cell extracts and culture supernatants, supporting the notion that MV formation is a regulated process rather than random membrane blebbing. The MVs displayed relatively consistent diameters (102−116 nm) among the strains, and while they were larger than those previously reported for TIGR4 MVs [18], they were still within the size range observed in that study. This size consistency among the tested strains suggests conserved mechanisms of MV biogenesis among MGS. Furthermore, MV yield was highest in TIGR4, and notable differences were also observed among *S. mitis* strains. Previous studies of MVs in *Streptococcus pyogenes* have reported similar counts, suggesting a potential correlation between invasiveness and MV production [38].

A key finding of this study was that MVs were readily internalized by A549 epithelial cells, with fluorescence persisting for approximately three days (Figure 5). Microscopic examination revealed no cell detachment or apparent cytotoxicity during this period following MV internalization, suggesting that the MVs did not directly harm epithelial cells. Although this intracellular uptake is consistent with observations in the MVs of other gram-positive bacteria, the specific uptake pathway remains unclear. Previous studies on different bacterial species have suggested various uptake pathways, including cholesterol-dependent mechanisms and clathrin-dependent endocytosis [39–41]. Further studies employing specific inhibitors of different endocytic pathways would be valuable for elucidating precise uptake mechanisms.

In addition to interactions between MVs and epithelial cells, our study demonstrated that the presence of MVs significantly reduced the phagocytic activity of THP-1 macrophages (Figure 6). Notably, the similar levels of inhibition observed among MVs from all sources suggested a shared mechanism of action, potentially involving interference with opsonization or phagocyte recognition. Previous studies have shown that MVs from *S. pneumoniae* bind to complement regulators, thereby interfering with opsonization [18]. The choline-binding protein PspC, the major factor H-binding protein contained in *S. pneumoniae* MVs, provides protection against complement-mediated killing through the modulation of alternative pathway activation [42,43]. Proteomic analysis has revealed that *S. mitis* MVs contain abundant choline-binding proteins. Based on the fact that *S. mitis* causes bloodstream infections, these proteins likely provide a similar complement-evasion function that promotes bacterial survival during bacteremia, potentially contributing to the development of complications, such as infective endocarditis.

Previous studies have reported that human macrophages respond to *S. aureus* MVs via TLR2 signaling and the activation of NLRP3 inflammasomes through K^+^ efflux, leading to the recruitment of ASC and the activation of caspase-1 [44]. Furthermore, it has been demonstrated that *S. sanguinis* MVs, when added to human gingival epithelial cells, induce the expression of inflammatory cytokines such as IL-6, IL-8, TNF-*α*, IL-1β, and Gro-*α* [35,36]. The immunomodulatory effects of MVs were further demonstrated by cytokine analysis. MVs tend to induce production of five cytokines (IL-1β, IL-6, IL-8, IL-10, and TNF-*α*) in macrophages (Table 3). Given that these measurements were conducted with only two biological replicates (*n* = 2) per condition, additional investigation is warranted to ensure robust statistical analysis. In contrast, neutrophils showed increased IL-8 production but no TNF-*α* release following MV exposure (Figure 7). These findings are consistent with those of previous studies showing that *S. pneumoniae* MVs induce cytokine responses [18], which likely depend on membrane components such as lipoteichoic acid and lipoproteins acting as TLR2 agonists [18,45]. Our observations on MV-mediated immune modulation also corroborate recent studies showing that streptococcal MVs have dual effects on immune cells, namely, enhancing macrophage mobilization while promoting bacterial survival [46]. Additionally, recent studies have demonstrated that CD169^+^ splenic macrophages serve as reservoirs for pneumococcal replication [47]. Based on the genetic relationship between *S. pneumoniae* and *S. mitis*, we propose that *S. mitis* MVs modulate macrophage function to influence bacterial clearance or persistence. Overall, streptococcal MVs appear to act as crucial mediators of bacteria-host interactions that may influence infection outcomes from clearance to systemic dissemination.

Our investigation into the effects of MVs on oral bacteria revealed intriguing ecological implications. Unlike previous studies on *Burkholderia thailandensis* OMVs, which exhibited anti-biofilm activity against *S. mutans* and reduced bacterial viability [48], our study found that *S. mitis* MVs had a limited impact on *S. mutans* biofilm formation or bacterial growth, showing a slight stimulatory effect instead. This difference highlights the species-specific nature of the vesicle-mediated bacterial interactions. Furthermore, *S. mitis* MVs enhanced the growth of *Aa*, possibly because of their high protein content, which serves as a nutrient source. However, at high concentrations, MVs from both *S. pneumoniae* TIGR4 and *S. mitis* Nm-65 inhibited *Aa* biofilm formation (Figure 9), suggesting a concentration-dependent dual role for these MVs. The contradictory effects of growth promotion and the inhibition of biofilm formation may be a survival strategy in densely populated oral niches. The strain-specific nature of this inhibition warrants further investigation by comparing MVs from strains TIGR4 and Nm-65 with those from strains Nm-76 and NCTC12261 to identify the specific factors responsible for this activity. Understanding these factors may lead to the development of novel MV-based therapeutic approaches targeting biofilm-forming periodontal pathogens .

## Conclusions

This study provides new insights into the functions of MVs produced by the MGS, particularly *S. mitis*. Our findings suggest that *S. mitis* employs MVs as a strategy to evade phagocyte-mediated killing and influences the growth and biofilm formation of other bacterial species. The immunogenicity of MVs highlights their potential use in vaccine development. These findings expand our understanding of streptococcal pathogenicity and open new avenues for developing preventive measures, including novel MV-based vaccines and anti-biofilm agents.

## Acknowledgments

The authors would like to thank Dr. H. Shinchi (Kagoshima University) for permitting the use of transmission electron microscopy, S. Kobaru and *N*. Mure (Kagoshima University) for technical assistance, Dr. Y. Niidome (Kagoshima University) for granting access to the zeta-potential and particle size analyzer and for technical assistance, and Dr. K. Nishino (Tokushima University) for technical assistance with LC-MS/MS analysis. We also appreciate Dr. M. Killian (Arhus University) and Dr. T. Hoshino (Meikai University School of Dentistry) for providing *S. sanguinis* strain SK36 and *S. mitis* strains SK142 and NCTC12261, respectively.

## Author contributions

**Airi Matsumoto**: study design, literature search, figures, data collection, data analysis, data interpretation, writing, funding acquisition. **Yuichi Oogai**: data collection, data analysis, data interpretation, funding acquisition. **Haruka Kurashige**: data collection, data analysis. **Tomoko Sumitomo**: data collection, data analysis. **Atsushi Tabata**: data collection, data analysis. **Masanobu Nakata**: study design, data analysis, data interpretation, writing, funding acquisition.

## Supplementary Material

Supplementary material Supplementary Table 1

Supplementary material Supplementary Figure

Supplementary material Supplementary Table 2

Supplementary material Supplementary Table 3

Supplementary material Supplementary Table 4

## Data Availability

The authors confirm that the data supporting the findings of this study are available within the article and its supplemental material.
