# Supplementary material for: Functional characteristics of membrane vesicles produced by Streptococcus mitis
Source: J Oral Microbiol. 2025 Sep 23;17(1):2557962. doi: 10.1080/20002297.2025.2557962 (PMC12459155; doi:10.1080/20002297.2025.2557962)
Supplement: Supplementary material — Supplementary Table 1 [file ZJOM_A_2557962_SM1731.docx]

**Supplementary Table 1.** Mass spectrometry identification of *S. pneumoniae* TIGR4 proteins present in MVs and their topology prediction

| Accession | Description | # PSMs | # Peptides | MW [kDa] | Score | Gene | Localization |
| --- | --- | --- | --- | --- | --- | --- | --- |
| WP_001035362.1 | pneumococcal surface protein A | 320 | 47 | 82.7 | 987.43 | *pspA* | Cell wall |
| WP_012386737.1 | glucosaminidase domain-containing protein | 173 | 55 | 78.8 | 521.64 | *lytB* | Cell wall |
| WP_000515174.1 | LPXTG-anchored adhesin/beta-galactosidase BgaA | 186 | 113 | 247.1 | 512.7 | *bgaA* | Cell wall |
| WP_000472995.1 | LPXTG cell wall anchor domain-containing protein | 162 | 109 | 213.4 | 456.7 | *zmpB* | Cell wall |
| WP_000458188.1 | choline-binding protein CbpA | 163 | 44 | 77.7 | 441.61 | *cbpA* | Cell wall |
| WP_001042526.1 | N-acetylmuramoyl-L-alanine amidase family protein | 154 | 20 | 37.6 | 359.61 | *cbpL* | Cell wall |
| WP_000753888.1 | choline-binding protein PcpA | 108 | 45 | 69.2 | 332.29 | *pcpA* | Cell wall |
| WP_001032461.1 | SpGH101 family endo-alpha-N-acetylgalactosaminidase | 116 | 80 | 195.9 | 309.56 |  | Cell wall |
| WP_000088129.1 | zinc metalloprotease ZmpC | 102 | 71 | 206.6 | 303.9 | *zmpC* | Cell wall |
| WP_000728269.1 | phosphorylcholine esterase CbpE | 95 | 35 | 72 | 275.87 | *cbpE* | Cell wall |
| WP_000750044.1 | S8 family serine peptidase | 68 | 58 | 240.3 | 157.86 |  | Cell wall |
| WP_000417180.1 | G5 domain-containing protein | 51 | 41 | 223.8 | 134.23 | *iga* | Cell wall |
| WP_000771084.1 | choline-binding protein CbpC | 35 | 17 | 39.5 | 99.01 | *cbpC* | Cell wall |
| WP_000698047.1 | choline-binding protein CbpJ | 43 | 11 | 38.9 | 97.67 | *cbpJ* | Cell wall |
| WP_000771060.1 | choline-binding protein CbpF | 33 | 17 | 39.8 | 75.4 | *cbpF* | Cell wall |
| WP_001232766.1 | LPXTG-anchored pullulanase SpuA | 33 | 30 | 142.5 | 72.32 | *spuA* | Cell wall |
| WP_000476386.1 | cell wall hydrolase Pmp23 | 29 | 10 | 23 | 68.54 | *pvaA* | Cell wall |
| WP_000638508.1 | 2-C-methyl-D-erythritol 4-phosphate cytidylyltransferase | 19 | 8 | 26.2 | 60.19 | *tarI* | Cell wall |
| WP_000405234.1 | MULTISPECIES: N-acetylmuramoyl-L-alanine amidase LytA | 28 | 14 | 36.5 | 59.08 | *lytA* | Cell wall |
| WP_001199652.1 | MULTISPECIES: phosphorylcholine transferase LicD | 8 | 7 | 32.1 | 23.41 | *licD2* | cell wall |
| WP_001224760.1 | serine hydrolase | 10 | 9 | 48.1 | 21.31 |  | cell wall |
| WP_010963222.1 | PI-1 pilus minor pilin RrgC | 9 | 6 | 44.7 | 14.2 | *rrgC* | Cell wall |
| WP_000048088.1 | UDP-N-acetylmuramate--L-alanine ligase | 4 | 4 | 49.9 | 12.3 | *murC* | Cell wall |
| WP_000446975.1 | glycosyltransferase | 3 | 2 | 38.1 | 10.18 | *wcaA* | Cell wall |
| WP_001808632.1 | peptidoglycan bridge formation alanyltransferase MurM | 4 | 4 | 47.4 | 9.23 | *murM* | Cell wall |
| WP_000919868.1 | D-alanyl-lipoteichoic acid biosynthesis protein DltD | 2 | 2 | 48.8 | 5.89 | *dltD* | Cell wall |
| WP_000835918.1 | type IV teichoic acid flippase TacF | 3 | 2 | 56.5 | 2.72 | *tacF* | Cell wall |
| WP_000260666.1 | MULTISPECIES: type I glyceraldehyde-3-phosphate dehydrogenase | 332 | 28 | 35.8 | 887.73 | *gapA* | Cytosolic |
| WP_000228766.1 | MULTISPECIES: DNA-directed RNA polymerase subunit beta' | 242 | 100 | 136.9 | 627.21 | *rpoC* | Cytosolic |
| WP_000022813.1 | MULTISPECIES: phosphopyruvate hydratase | 161 | 34 | 47.1 | 431.22 | *eno* | Cytosolic |
| WP_001042809.1 | pyruvate kinase | 152 | 44 | 54.7 | 424.07 | *pyk* | Cytosolic |
| WP_000907151.1 | DNA-directed RNA polymerase subunit beta | 137 | 66 | 134.3 | 392.48 | *rpoB* | Cytosolic |
| WP_000191798.1 | MULTISPECIES: pyruvate oxidase | 95 | 31 | 65.2 | 293.2 | *spxB* | Cytosolic |
| WP_000512911.1 | MULTISPECIES: 50S ribosomal protein L2 | 113 | 20 | 29.9 | 286.4 | *rplB* | Cytosolic |
| WP_000205044.1 | MULTISPECIES: adenylosuccinate synthase | 93 | 32 | 47.5 | 281.47 | *purA* | Cytosolic |
| WP_001085675.1 | MULTISPECIES: 50S ribosomal protein L1 [Bacteria] | 88 | 12 | 24.5 | 204.83 | *rplA* | Cytosolic |
| WP_000013542.1 | MULTISPECIES: 50S ribosomal protein L5 [Bacteria] | 80 | 15 | 19.8 | 204.7 | *rplE* | Cytosolic |
| WP_000608356.1 | threonine--tRNA ligase | 72 | 34 | 74.7 | 203.15 | *thrS* | Cytosolic |
| WP_000094354.1 | MULTISPECIES: F0F1 ATP synthase subunit beta | 66 | 30 | 50.9 | 187.51 | *atpD* | Cytosolic |
| WP_000204727.1 | MULTISPECIES: L-lactate dehydrogenase | 52 | 21 | 35.3 | 172.99 | *ldh* | Cytosolic |
| WP_000229961.1 | sn-glycerol-3-phosphate ABC transporter ATP-binding protein UgpC | 57 | 27 | 41.8 | 167.89 | *msmK* | Cytosolic |
| WP_000991674.1 | MULTISPECIES: aminopeptidase C | 49 | 28 | 50.2 | 157.63 | *pepC* | Cytosolic |
| WP_000031573.1 | MULTISPECIES: chaperonin GroEL | 57 | 27 | 57.1 | 155.21 | *groL* | Cytosolic |
| WP_000067853.1 | transketolase | 46 | 27 | 71.1 | 149.73 | *tkt* | Cytosolic |
| WP_000990607.1 | catabolite control protein A | 57 | 25 | 37.1 | 147.93 | *ccpA* | Cytosolic |
| WP_000034665.1 | molecular chaperone DnaK | 56 | 33 | 64.8 | 145.59 | *dnaK* | Cytosolic |
| WP_001096747.1 | phosphoglycerate kinase | 44 | 27 | 41.9 | 144.38 | *pgk* | Cytosolic |
| WP_000874201.1 | MULTISPECIES: 30S ribosomal protein S5 | 41 | 10 | 17 | 140.98 | *rpsE* | Cytosolic |
| WP_000515972.1 | MULTISPECIES: uracil phosphoribosyltransferase | 39 | 16 | 22.9 | 136.98 | *upp* | Cytosolic |
| WP_001287278.1 | MULTISPECIES: 50S ribosomal protein L10 [Bacteria] | 40 | 17 | 17.5 | 134.11 | *rplJ* | Cytosolic |
| WP_000090344.1 | elongation factor G | 45 | 27 | 76.8 | 130.17 | *fusA* | Cytosolic |
| WP_000159554.1 | MULTISPECIES: ABC transporter ATP-binding protein | 46 | 25 | 39.5 | 125.45 | *amiE* | Cytosolic |
| WP_000032175.1 | valine--tRNA ligase | 48 | 32 | 100.7 | 125.26 | *valS* | Cytosolic |
| WP_000649468.1 | zinc-dependent alcohol dehydrogenase family protein | 47 | 16 | 38.1 | 124.6 |  | Cytosolic |
| WP_000158781.1 | MULTISPECIES: NADP-dependent phosphogluconate dehydrogenase | 46 | 27 | 52.5 | 122.2 | *gnd* | Cytosolic |
| WP_001809310.1 | pur operon repressor | 46 | 26 | 30.2 | 118.88 | *purR* | Cytosolic |
| WP_000818137.1 | MULTISPECIES: 50S ribosomal protein L22 | 39 | 12 | 12.2 | 117.47 | *rplV* | Cytosolic |
| WP_001808444.1 | MULTISPECIES: SPFH domain-containing protein | 42 | 26 | 33 | 114.83 |  | Cytosolic |
| WP_001291293.1 | MULTISPECIES: ATP-binding cassette domain-containing protein | 39 | 16 | 34.8 | 111.47 | *amiF* | Cytosolic |
| WP_000121704.1 | phospho-sugar mutase | 36 | 22 | 62.6 | 110.87 | *pgm* | Cytosolic |
| WP_000011793.1 | leucine--tRNA ligase | 38 | 29 | 94.4 | 107 | *leuS* | Cytosolic |
| WP_001122904.1 | type I glutamate--ammonia ligase | 40 | 22 | 50.3 | 102.07 | *glnA* | Cytosolic |
| WP_001196049.1 | MULTISPECIES: hypothetical protein | 35 | 13 | 12.9 | 101.76 |  | Cytosolic |
| WP_000255160.1 | dihydroorotate oxidase | 37 | 15 | 34.5 | 101.07 | *pyrDA* | Cytosolic |
| WP_001285241.1 | MULTISPECIES: tRNA guanosine(34) transglycosylase Tgt | 34 | 8 | 43.1 | 100.77 | *tgt* | Cytosolic |
| WP_000533766.1 | MULTISPECIES: 30S ribosomal protein S19 [Bacteria] | 37 | 5 | 10.7 | 100.26 | *rpsS* | Cytosolic |
| WP_000010163.1 | MULTISPECIES: ribose-phosphate diphosphokinase | 31 | 16 | 35.4 | 100.21 | *prs1* | Cytosolic |
| WP_001040724.1 | MULTISPECIES: elongation factor Tu [Bacteria] | 46 | 20 | 43.9 | 99.73 | *tuf* | Cytosolic |
| WP_000120722.1 | L-lactate oxidase | 37 | 22 | 41.5 | 98.77 | *lctO-2* | Cytosolic |
| WP_000568988.1 | MULTISPECIES: DNA-directed RNA polymerase subunit alpha | 46 | 19 | 34.2 | 97.62 | *rpoA* | Cytosolic |
| WP_001274091.1 | preprotein translocase subunit SecA | 36 | 26 | 95 | 93.54 | *secA1* | Cytosolic |
| WP_000146522.1 | cell division regulator GpsB | 23 | 9 | 12.6 | 91.93 | *gpsB* | Cytosolic |
| WP_000039617.1 | NAD(P)H-dependent oxidoreductase | 27 | 12 | 19.8 | 90.27 |  | Cytosolic |
| WP_000018264.1 | glucose-6-phosphate isomerase | 27 | 19 | 49.8 | 89.71 | *pgi* | Cytosolic |
| WP_000124836.1 | MULTISPECIES: 50S ribosomal protein L20 | 36 | 8 | 13.7 | 88.68 | *rplT* | Cytosolic |
| WP_001019003.1 | MULTISPECIES: fructose-bisphosphate aldolase | 30 | 13 | 31.4 | 87.27 | *fba* | Cytosolic |
| WP_000064115.1 | Asp23/Gls24 family envelope stress response protein | 30 | 17 | 21.8 | 87.11 |  | Cytosolic |
| WP_000086634.1 | 50S ribosomal protein L6 | 30 | 11 | 19.4 | 86.95 | *rplF* | Cytosolic |
| WP_000092756.1 | MULTISPECIES: 30S ribosomal protein S4 [Bacteria] | 41 | 17 | 23 | 85.7 | *rpsD* | Cytosolic |
| WP_000946463.1 | phosphate signaling complex protein PhoU | 33 | 16 | 25 | 84.94 |  | Cytosolic |
| WP_000763969.1 | bifunctional acetaldehyde-CoA/alcohol dehydrogenase | 36 | 29 | 97.2 | 81.59 |  | Cytosolic |
| WP_000109217.1 | MULTISPECIES: phosphate ABC transporter ATP-binding protein PstB | 29 | 13 | 30.3 | 81.51 | *pstB2* | Cytosolic |
| WP_000940729.1 | GTP-sensing pleiotropic transcriptional regulator CodY | 31 | 16 | 29.8 | 81.03 | *codY* | Cytosolic |
| WP_000202234.1 | UTP--glucose-1-phosphate uridylyltransferase GalU | 26 | 15 | 33.3 | 80.76 | *cap4C* | Cytosolic |
| WP_000649161.1 | MULTISPECIES: alcohol dehydrogenase AdhP | 23 | 12 | 35.7 | 80.67 |  | Cytosolic |
| WP_001096313.1 | MULTISPECIES: amino acid ABC transporter ATP-binding protein | 30 | 8 | 27.3 | 80.59 |  | Cytosolic |
| WP_000960946.1 | MULTISPECIES: 50S ribosomal protein L16 | 23 | 6 | 15.4 | 79.96 | *rplP* | Cytosolic |
| WP_001284639.1 | MULTISPECIES: HU family DNA-binding protein | 24 | 8 | 9.6 | 77.7 | *hup* | Cytosolic |
| WP_000143747.1 | Asp-tRNA(Asn)/Glu-tRNA(Gln) amidotransferase subunit GatA | 28 | 20 | 52 | 77.09 | *gatA* | Cytosolic |
| WP_001018251.1 | MULTISPECIES: 30S ribosomal protein S15 [Bacteria] | 28 | 7 | 10.5 | 76.1 | *rpsO* | Cytosolic |
| WP_000022268.1 | cell division ATP-binding protein FtsE | 30 | 16 | 25.8 | 75.37 | *ftsE* | Cytosolic |
| WP_000744528.1 | zinc-binding lipoprotein AdcAII | 30 | 16 | 33.9 | 74.02 | *Imb* | Cytosolic |
| WP_000201898.1 | RNA polymerase sigma factor RpoD | 26 | 15 | 42 | 73.33 | *sigA* | Cytosolic |
| WP_001118385.1 | MULTISPECIES: 30S ribosomal protein S11 [Bacteria] | 29 | 4 | 13.4 | 72.23 | *rpsK* | Cytosolic |
| WP_000087875.1 | 30S ribosomal protein S7 | 25 | 10 | 17.7 | 72.16 | *rpsG* | Cytosolic |
| WP_000268465.1 | 30S ribosomal protein S2 | 24 | 15 | 28.8 | 71.83 | *rpsB* | Cytosolic |
| WP_000774063.1 | MULTISPECIES: beta-ketoacyl-ACP synthase II | 21 | 16 | 43.9 | 71.37 | *fabF* | Cytosolic |
| WP_000245505.1 | MULTISPECIES: 30S ribosomal protein S8 [Bacteria] | 23 | 9 | 14.7 | 70.78 | *rpsH* | Cytosolic |
| WP_000209199.1 | MULTISPECIES: glutamyl aminopeptidase | 26 | 11 | 38 | 70.67 | *pepA* | Cytosolic |
| WP_000763053.1 | MULTISPECIES: 3-oxoacyl-[acyl-carrier-protein] reductase | 22 | 10 | 25.7 | 70.6 | *fabG* | Cytosolic |
| WP_000529936.1 | MULTISPECIES: 30S ribosomal protein S3 | 20 | 12 | 24 | 69.53 | *rpsC* | Cytosolic |
| WP_000869659.1 | aspartate kinase | 26 | 16 | 50.2 | 68.66 |  | Cytosolic |
| WP_000260643.1 | MULTISPECIES: formate C-acetyltransferase | 25 | 18 | 87.8 | 68.44 | *pfl* | Cytosolic |
| WP_000497691.1 | MULTISPECIES: 50S ribosomal protein L24 [Bacteria] | 20 | 10 | 11 | 67.85 | *rplX* | Cytosolic |
| WP_000830893.1 | aspartate--tRNA ligase | 32 | 21 | 66.3 | 67.51 | *aspS* | Cytosolic |
| WP_000747993.1 | MULTISPECIES: aspartate--ammonia ligase | 22 | 16 | 37.6 | 65.63 | *asnA* | Cytosolic |
| WP_000160197.1 | MULTISPECIES: 50S ribosomal protein L3 | 22 | 8 | 22.2 | 64.91 | *rplC* | Cytosolic |
| WP_000021975.1 | MULTISPECIES: PTS sugar transporter subunit IIB | 26 | 15 | 35.4 | 64.83 | *manL* | Cytosolic |
| WP_001149111.1 | M1 family metallopeptidase | 23 | 20 | 95.2 | 64.8 | *pepN* | Cytosolic |
| WP_000138135.1 | MULTISPECIES: phosphoenolpyruvate--protein phosphotransferase | 23 | 17 | 63.3 | 64.54 | *ptsI* | Cytosolic |
| WP_000077424.1 | capsular polysaccharide biosynthesis protein Cps4J | 26 | 19 | 39.1 | 64.21 | *cap4J* | Cytosolic |
| WP_000199387.1 | NADP-dependent glyceraldehyde-3-phosphate dehydrogenase | 28 | 21 | 51.1 | 63.12 | *gapN* | Cytosolic |
| WP_001044624.1 | MULTISPECIES: 50S ribosomal protein L13 | 23 | 9 | 16.1 | 62.6 | *rplM* | Cytosolic |
| WP_001099502.1 | MULTISPECIES: 6,7-dimethyl-8-ribityllumazine synthase | 20 | 8 | 16.7 | 62.35 | *ribH* | Cytosolic |
| WP_000775862.1 | MULTISPECIES: histidine--tRNA ligase | 28 | 22 | 48.6 | 62.25 | *hisS* | Cytosolic |
| WP_000910909.1 | MULTISPECIES: DUF6110 family protein | 25 | 9 | 10 | 62.19 |  | Cytosolic |
| WP_000116479.1 | trigger factor | 18 | 15 | 47.2 | 59.57 | *tig* | Cytosolic |
| WP_000902274.1 | polysaccharide pyruvyl transferase family protein | 18 | 11 | 40.8 | 58.3 |  | Cytosolic |
| WP_001055347.1 | MULTISPECIES: 50S ribosomal protein L23 [Bacteria] | 22 | 9 | 10.8 | 57.85 | *rplW* | Cytosolic |
| WP_001827240.1 | polyribonucleotide nucleotidyltransferase | 26 | 19 | 81 | 57.37 | *pnp* | Cytosolic |
| WP_000974746.1 | MULTISPECIES: superoxide dismutase SodA | 21 | 13 | 22.4 | 57.25 | *sodA* | Cytosolic |
| WP_000857427.1 | MULTISPECIES: enoyl-[acyl-carrier-protein] reductase FabK | 20 | 13 | 34.2 | 57.04 | *fabK* | Cytosolic |
| WP_000996639.1 | F0F1 ATP synthase subunit alpha | 25 | 12 | 54.6 | 56.22 | *atpA* | Cytosolic |
| WP_000929853.1 | ABC transporter ATP-binding protein | 21 | 18 | 55.1 | 55.55 |  | Cytosolic |
| WP_000199560.1 | NADP-specific glutamate dehydrogenase | 22 | 13 | 48.8 | 55.48 | *gdhA* | Cytosolic |
| WP_000024543.1 | MULTISPECIES: 50S ribosomal protein L4 | 16 | 9 | 22.1 | 55.44 | *rplD* | Cytosolic |
| WP_000546887.1 | tyrosine--tRNA ligase | 21 | 13 | 47.5 | 55.19 | *tyrS* | Cytosolic |
| WP_000128639.1 | phosphate ABC transporter ATP-binding protein PstB | 20 | 16 | 27.9 | 54.58 | *pstB1* | Cytosolic |
| WP_000575209.1 | MULTISPECIES: ABC transporter ATP-binding protein | 24 | 14 | 27 | 53.67 | *proV* | Cytosolic |
| WP_000624044.1 | MULTISPECIES: 50S ribosomal protein L18 [Terrabacteria group] | 16 | 7 | 12.9 | 53.65 | *rplR* | Cytosolic |
| WP_000931182.1 | GMP reductase | 22 | 16 | 35.9 | 53.23 | *guaC* | Cytosolic |
| WP_000090397.1 | MULTISPECIES: cell division protein FtsA | 27 | 13 | 49.4 | 53.18 | *ftsA* | Cytosolic |
| WP_000036793.1 | FAD-dependent oxidoreductase | 21 | 16 | 50.2 | 52.72 | *nox* | Cytosolic |
| WP_000950154.1 | glycogen/starch/alpha-glucan family phosphorylase | 19 | 15 | 85.1 | 52.26 | *malP* | Cytosolic |
| WP_000090781.1 | MULTISPECIES: 30S ribosomal protein S13 [Bacteria] | 21 | 12 | 13.4 | 50.25 | *rpsM* | Cytosolic |
| WP_000331980.1 | MULTISPECIES: ribonuclease J | 23 | 16 | 61 | 50.02 | *rnj* | Cytosolic |
| WP_000747261.1 | DUF5590 domain-containing protein | 16 | 8 | 18.3 | 49.88 |  | Cytosolic |
| WP_001068669.1 | MULTISPECIES: 50S ribosomal protein L19 | 21 | 10 | 13.1 | 48.86 | *rplS* | Cytosolic |
| WP_000065723.1 | glutamine-hydrolyzing GMP synthase | 15 | 11 | 57.4 | 48.57 | *guaA* | Cytosolic |
| WP_000523249.1 | MULTISPECIES: class 1b ribonucleoside-diphosphate reductase subunit alpha | 21 | 18 | 81.5 | 47.62 | *nrdE* | Cytosolic |
| WP_000040908.1 | Xaa-Pro peptidase family protein | 17 | 10 | 40.2 | 47.59 | *pepQ* | Cytosolic |
| WP_000510591.1 | MULTISPECIES: Asp23/Gls24 family envelope stress response protein | 19 | 9 | 14.1 | 47.39 |  | Cytosolic |
| WP_000216382.1 | MULTISPECIES: homoserine dehydrogenase | 18 | 15 | 46.1 | 46.84 | *hom* | Cytosolic |
| WP_000590970.1 | MULTISPECIES: amino acid ABC transporter ATP-binding protein | 17 | 12 | 27.4 | 46.31 |  | Cytosolic |
| WP_000848180.1 | MULTISPECIES: translation initiation factor IF-3 | 16 | 7 | 20.1 | 45.53 | *infC* | Cytosolic |
| WP_001222228.1 | MULTISPECIES: rRNA pseudouridine synthase | 19 | 13 | 27.1 | 45.48 | *rluB* | Cytosolic |
| WP_000717458.1 | phosphate acyltransferase PlsX | 15 | 11 | 34.8 | 45.45 | *plsX* | Cytosolic |
| WP_000619143.1 | MULTISPECIES: metal ABC transporter ATP-binding protein | 18 | 13 | 26.8 | 45.42 | *psaB* | Cytosolic |
| WP_000039196.1 | translation initiation factor IF-2 | 22 | 17 | 105.7 | 45.19 | *infB* | Cytosolic |
| WP_001156518.1 | UDP-glucose 4-epimerase GalE | 15 | 8 | 37.4 | 45.08 | *galE-1* | Cytosolic |
| WP_000087897.1 | triose-phosphate isomerase | 15 | 10 | 26.5 | 44.92 | *tpiA* | Cytosolic |
| WP_001808386.1 | cell division protein DivIVA | 15 | 10 | 30.1 | 44.89 | *divIVA* | Cytosolic |
| WP_000673690.1 | NAD(P)H-dependent oxidoreductase | 18 | 12 | 45.8 | 44 |  | Cytosolic |
| WP_000666479.1 | pyridoxal phosphate-dependent aminotransferase | 20 | 12 | 45.7 | 43.33 |  | Cytosolic |
| WP_000240129.1 | MULTISPECIES: phosphoglycerate mutase | 22 | 15 | 26 | 43 | *gpmA* | Cytosolic |
| WP_000085659.1 | methionine ABC transporter ATP-binding protein | 14 | 11 | 38.8 | 41.36 | *metN* | Cytosolic |
| WP_000199265.1 | endopeptidase PepO | 15 | 15 | 71.9 | 41.05 | *pepO* | Cytosolic |
| WP_000301212.1 | F0F1 ATP synthase subunit gamma | 14 | 10 | 32.3 | 40.96 | *atpG* | Cytosolic |
| WP_001212037.1 | Xaa-Pro dipeptidyl-peptidase | 16 | 14 | 86.8 | 39.37 | *pepX* | Cytosolic |
| WP_000094620.1 | arginine deiminase | 14 | 12 | 46.6 | 39.36 | *arcA* | Cytosolic |
| WP_000079148.1 | MULTISPECIES: universal stress protein [Bacteria] | 12 | 6 | 16.6 | 39.05 |  | Cytosolic |
| WP_000766087.1 | MULTISPECIES: 50S ribosomal protein L15 [Lactobacillales] | 16 | 7 | 15.4 | 38.93 | *rplO* | Cytosolic |
| WP_001818763.1 | MULTISPECIES: phenylalanine--tRNA ligase subunit alpha | 15 | 10 | 39.1 | 38.84 | *pheS* | Cytosolic |
| WP_000354338.1 | ribonuclease Z | 15 | 12 | 34.1 | 38.28 | *rnz* | Cytosolic |
| WP_000065637.1 | MULTISPECIES: ribonuclease J | 15 | 11 | 61.1 | 38.26 | *rnj* | Cytosolic |
| WP_001008681.1 | Asp-tRNA(Asn)/Glu-tRNA(Gln) amidotransferase subunit GatB | 17 | 12 | 53.6 | 38.2 | *gatB* | Cytosolic |
| WP_000820852.1 | MULTISPECIES: 6-phosphofructokinase | 12 | 10 | 35.2 | 37.83 | *pfkA* | Cytosolic |
| WP_000268761.1 | MULTISPECIES: 30S ribosomal protein S16 | 12 | 5 | 10.2 | 37.83 | *rpsP* | Cytosolic |
| WP_000594468.1 | capsular polysaccharide biosynthesis protein Cps4K | 19 | 12 | 46.6 | 37.7 | *cps4K* | Cytosolic |
| WP_000096161.1 | glucose-6-phosphate dehydrogenase | 21 | 16 | 56.8 | 37.69 | *zwf* | Cytosolic |
| WP_000891772.1 | amino acid ABC transporter ATP-binding protein | 12 | 9 | 23.1 | 37.61 |  | Cytosolic |
| WP_000083696.1 | methylenetetrahydrofolate--tRNA-(uracil(54)-C(5))-methyltransferase (FADH(2)-oxidizing) TrmFO | 13 | 10 | 49.2 | 37.34 | *trmFO* | Cytosolic |
| WP_001142502.1 | capsular polysaccharide biosynthesis protein Cps4D | 18 | 7 | 24.9 | 37.24 | *cpsD* | Cytosolic |
| WP_001284513.1 | MULTISPECIES: 30S ribosomal protein S10 [Terrabacteria group] | 16 | 8 | 11.6 | 36.81 | *rpsJ* | Cytosolic |
| WP_000334262.1 | glutamine--fructose-6-phosphate transaminase (isomerizing) | 12 | 10 | 65.6 | 36.08 | *glmS* | Cytosolic |
| WP_000916509.1 | MULTISPECIES: 50S ribosomal protein L27 [Bacteria] | 12 | 4 | 10.5 | 35.98 | *rpmA* | Cytosolic |
| WP_000743611.1 | DUF1846 domain-containing protein | 14 | 13 | 55 | 35.85 |  | Cytosolic |
| WP_000892185.1 | hypoxanthine phosphoribosyltransferase | 18 | 12 | 20.2 | 35.68 | *hpt* | Cytosolic |
| WP_000811773.1 | alanine--tRNA ligase | 13 | 12 | 96.4 | 35.64 | *alaS* | Cytosolic |
| WP_000640786.1 | 1-phosphofructokinase | 13 | 10 | 32.6 | 35.32 |  | Cytosolic |
| WP_000687067.1 | glucose-1-phosphate adenylyltransferase subunit GlgD | 10 | 8 | 42.6 | 34.78 | *glgD* | Cytosolic |
| WP_000775037.1 | MULTISPECIES: guanylate kinase | 12 | 8 | 23.7 | 34.45 | *gmk* | Cytosolic |
| WP_001092737.1 | arginine--tRNA ligase | 14 | 12 | 63.4 | 34.24 | *argS* | Cytosolic |
| WP_000331494.1 | 50S ribosomal protein L17 | 13 | 6 | 14.5 | 34.21 | *rplQ* | Cytosolic |
| WP_001007202.1 | MULTISPECIES: GntR family transcriptional regulator | 12 | 8 | 27.9 | 34.15 |  | Cytosolic |
| WP_001196960.1 | MULTISPECIES: 50S ribosomal protein L7/L12 [Bacteria] | 9 | 6 | 12.4 | 33.83 | *rplL* | Cytosolic |
| WP_000218054.1 | ketol-acid reductoisomerase | 12 | 8 | 37.3 | 33.36 | *ilvC* | Cytosolic |
| WP_001808726.1 | 3-phosphoshikimate 1-carboxyvinyltransferase | 11 | 10 | 45.7 | 33.24 | *aroA* | Cytosolic |
| WP_000814067.1 | proline--tRNA ligase | 14 | 13 | 68.6 | 32.83 | *proS* | Cytosolic |
| WP_001001626.1 | 30S ribosomal protein S1 | 15 | 12 | 43.9 | 32.38 | *rpsA* | Cytosolic |
| WP_000864060.1 | 3-deoxy-7-phosphoheptulonate synthase | 8 | 6 | 38.7 | 31.47 |  | Cytosolic |
| WP_001229501.1 | peptidoglycan bridge formation alanyltransferase MurN | 14 | 11 | 47.3 | 30.87 | *fibB* | Cytosolic |
| WP_000768087.1 | isoleucine--tRNA ligase | 12 | 10 | 105.4 | 30.84 | *ileS* | Cytosolic |
| WP_000884253.1 | serine--tRNA ligase | 14 | 10 | 47.7 | 30.66 | *serS* | Cytosolic |
| WP_000185363.1 | ornithine carbamoyltransferase | 11 | 9 | 37.9 | 30.57 | *arcB* | Cytosolic |
| WP_000062224.1 | ABC transporter ATP-binding protein | 15 | 9 | 25.7 | 30.19 | *livF* | Cytosolic |
| WP_001106629.1 | ATP-binding protein | 11 | 8 | 47.1 | 29.74 |  | Cytosolic |
| WP_000144280.1 | cell division protein FtsZ | 13 | 9 | 44.4 | 28.86 | *ftsZ* | Cytosolic |
| WP_000366370.1 | MULTISPECIES: pyridoxal phosphate-dependent aminotransferase | 11 | 10 | 43.2 | 28.69 | *araT* | Cytosolic |
| WP_001085809.1 | MULTISPECIES: 50S ribosomal protein L11 | 12 | 5 | 14.8 | 28.64 | *rplK* | Cytosolic |
| WP_001142332.1 | MULTISPECIES: 30S ribosomal protein S12 [Bacteria] | 14 | 6 | 15.1 | 28.58 | *rpsL* | Cytosolic |
| WP_000075966.1 | 30S ribosomal protein S9 | 14 | 5 | 14.2 | 28.32 | *rpsI* | Cytosolic |
| WP_000415103.1 | NAD(P)H-dependent glycerol-3-phosphate dehydrogenase | 9 | 7 | 36.8 | 28.25 | *gpsA* | Cytosolic |
| WP_000033830.1 | MULTISPECIES: CBS-HotDog domain-containing transcription factor SpxR | 10 | 7 | 47.6 | 27.87 |  | Cytosolic |
| WP_000451584.1 | phosphate acetyltransferase | 8 | 6 | 35.1 | 27.5 | *pta* | Cytosolic |
| WP_000183297.1 | tRNA dihydrouridine synthase DusB | 11 | 9 | 35.8 | 27.46 |  | Cytosolic |
| WP_000002996.1 | MULTISPECIES: UMP kinase | 10 | 9 | 26.4 | 26.85 | *pyrH* | Cytosolic |
| WP_000429305.1 | ribose-5-phosphate isomerase RpiA | 8 | 7 | 24.8 | 26.64 | *rpiA* | Cytosolic |
| WP_000575527.1 | serine hydroxymethyltransferase | 10 | 10 | 45.2 | 25.99 | *glyA* | Cytosolic |
| WP_161801414.1 | asparagine--tRNA ligase | 12 | 11 | 51.2 | 25.51 | *asnS* | Cytosolic |
| WP_000036661.1 | DAK2 domain-containing protein | 10 | 9 | 60.1 | 24.92 |  | Cytosolic |
| WP_001029580.1 | MULTISPECIES: thioredoxin | 8 | 5 | 11.4 | 24.89 | *trx* | Cytosolic |
| WP_000127471.1 | 2,3,4,5-tetrahydropyridine-2,6-dicarboxylate N-acetyltransferase | 8 | 5 | 23.9 | 24.48 | *depH* | Cytosolic |
| WP_000958925.1 | FAD-containing oxidoreductase | 7 | 5 | 47.1 | 24.41 |  | Cytosolic |
| WP_001808393.1 | MULTISPECIES: type B 50S ribosomal protein L31 | 6 | 4 | 11.1 | 24.38 | *rpmE2* | Cytosolic |
| WP_000873990.1 | lipoate--protein ligase | 12 | 8 | 37.7 | 24.36 |  | Cytosolic |
| WP_000105241.1 | CTP synthase | 7 | 7 | 59.2 | 23.98 | *pyrG* | Cytosolic |
| WP_000627748.1 | MULTISPECIES: type I restriction-modification system subunit M | 8 | 8 | 56.6 | 23.95 |  | Cytosolic |
| WP_000653403.1 | MULTISPECIES: redox-sensing transcriptional repressor Rex | 11 | 7 | 24.2 | 23.77 | *rex* | Cytosolic |
| WP_000036043.1 | manganese-dependent inorganic pyrophosphatase | 9 | 8 | 33.5 | 23.56 | *ppaC* | Cytosolic |
| WP_001042995.1 | MULTISPECIES: arginine repressor [Bacteria] | 6 | 4 | 17.8 | 23.4 | *argR* | Cytosolic |
| WP_000125043.1 | dipeptidase PepV | 9 | 7 | 50.8 | 22.8 | *pepV* | Cytosolic |
| WP_000243919.1 | MULTISPECIES: aminopeptidase | 7 | 5 | 45.2 | 22.79 | *pepS* | Cytosolic |
| WP_000759278.1 | MULTISPECIES: redoxin family protein | 11 | 8 | 20.8 | 22.35 |  | Cytosolic |
| WP_000599104.1 | MULTISPECIES: ribosome-associated translation inhibitor RaiA | 8 | 6 | 21.1 | 22.24 | *yfi hpf* | Cytosolic |
| WP_001281507.1 | dihydrodipicolinate synthase family protein | 11 | 8 | 33.4 | 21.98 |  | Cytosolic |
| WP_001151785.1 | MULTISPECIES: 30S ribosomal protein S6 [Bacteria] | 10 | 6 | 11.1 | 21.91 | *rpsF* | Cytosolic |
| WP_000151960.1 | hydroxymethylglutaryl-CoA synthase | 11 | 9 | 43.5 | 21.53 | *pksG* | Cytosolic |
| WP_000808063.1 | translation elongation factor Ts | 9 | 7 | 37.3 | 21.03 | *tsf* | Cytosolic |
| WP_000742290.1 | MULTISPECIES: YbbR-like domain-containing protein | 7 | 5 | 27.9 | 20.65 |  | Cytosolic |
| WP_000068664.1 | MULTISPECIES: 30S ribosomal protein S18 [Bacteria] | 7 | 5 | 9.2 | 20.53 | *rpsR* | Cytosolic |
| WP_001227958.1 | helix-turn-helix domain-containing protein | 8 | 6 | 30.2 | 20.32 | *pyrE* | Cytosolic |
| WP_000109141.1 | MULTISPECIES: 50S ribosomal protein L21 [Bacteria] | 20 | 2 | 11.2 | 20.21 | *rplU* | Cytosolic |
| WP_000119153.1 | MULTISPECIES: GntR family transcriptional regulator | 8 | 6 | 14 | 19.78 |  | Cytosolic |
| WP_000058203.1 | phosphoenolpyruvate carboxylase | 12 | 10 | 103.3 | 19.6 | *ppc* | Cytosolic |
| WP_001283896.1 | TrkA family potassium uptake protein | 10 | 8 | 23.9 | 19.42 |  | Cytosolic |
| WP_001229146.1 | tagatose-bisphosphate aldolase | 8 | 8 | 36.4 | 19.01 |  | Cytosolic |
| WP_001273594.1 | 30S ribosome-binding factor RbfA | 9 | 5 | 13.3 | 19.01 | *rbfA* | Cytosolic |
| WP_000777779.1 | pyridoxal phosphate-dependent aminotransferase | 9 | 6 | 43 | 18.91 |  | Cytosolic |
| WP_000048055.1 | MULTISPECIES: 30S ribosomal protein S21 [Bacteria] | 10 | 6 | 7 | 18.88 | *rpsU* | Cytosolic |
| WP_000702172.1 | 3-dehydroquinate synthase | 5 | 5 | 38.9 | 18.76 |  | Cytosolic |
| WP_000229874.1 | DNA starvation/stationary phase protection protein | 8 | 6 | 19.2 | 18.69 |  | Cytosolic |
| WP_001253803.1 | MULTISPECIES: acetolactate synthase small subunit | 6 | 5 | 17.7 | 18.46 |  | Cytosolic |
| WP_000222983.1 | ROK family protein | 9 | 5 | 31.6 | 18.4 |  | Cytosolic |
| WP_000167757.1 | acetate kinase | 10 | 9 | 43.3 | 18.29 | *ackA* | Cytosolic |
| WP_001134540.1 | DegT/DnrJ/EryC1/StrS aminotransferase family protein | 8 | 7 | 45.6 | 18.16 |  | Cytosolic |
| WP_000125408.1 | ABC transporter ATP-binding protein | 7 | 5 | 28.4 | 17.81 |  | Cytosolic |
| WP_000102456.1 | lysine--tRNA ligase | 8 | 6 | 56.7 | 17.68 | *lysS* | Cytosolic |
| WP_000164110.1 | translational GTPase TypA | 11 | 7 | 68.2 | 17.61 |  | Cytosolic |
| WP_001186003.1 | ABC transporter ATP-binding protein | 11 | 10 | 28.3 | 17.55 | *livG* | Cytosolic |
| WP_000138517.1 | MULTISPECIES: pyridoxal 5'-phosphate synthase lyase subunit PdxS | 7 | 6 | 31.7 | 17.49 | *pdxS* | Cytosolic |
| WP_000672589.1 | pneumococcal histidine triad protein PhtE | 6 | 5 | 114.6 | 17.47 | *phtE* | Cytosolic |
| WP_000961511.1 | phenylalanine--tRNA ligase subunit beta | 6 | 6 | 87.1 | 17.31 | *pheT* | Cytosolic |
| WP_001099678.1 | aldo/keto reductase | 9 | 7 | 31.4 | 17.2 |  | Cytosolic |
| WP_000358028.1 | UDP-N-acetylglucosamine 1-carboxyvinyltransferase | 9 | 9 | 45.8 | 17.19 | *murA1* | Cytosolic |
| WP_000440801.1 | MULTISPECIES: 30S ribosomal protein S17 [Bacteria] | 7 | 3 | 10 | 17.17 | *rpsQ* | Cytosolic |
| WP_000232209.1 | 3-deoxy-7-phosphoheptulonate synthase | 6 | 5 | 38.8 | 17.09 |  | Cytosolic |
| WP_000915958.1 | NAD(P)H-dependent oxidoreductase | 10 | 7 | 22.6 | 16.88 |  | Cytosolic |
| WP_001152989.1 | DNA gyrase subunit A | 13 | 13 | 92 | 16.82 | *gyrA* | Cytosolic |
| WP_001200084.1 | MULTISPECIES: tRNA 4-thiouridine(8) synthase ThiI | 8 | 8 | 45.1 | 16.47 |  | Cytosolic |
| WP_000858730.1 | MULTISPECIES: DUF1836 domain-containing protein | 7 | 5 | 16.9 | 16.25 |  | Cytosolic |
| WP_000654907.1 | ribonuclease R | 7 | 6 | 89.2 | 16.15 | *rnr* | Cytosolic |
| WP_000451370.1 | class 1b ribonucleoside-diphosphate reductase subunit beta | 6 | 5 | 37 | 14.94 |  | Cytosolic |
| WP_000031087.1 | glutamate--tRNA ligase | 13 | 12 | 55.9 | 14.77 |  | Cytosolic |
| WP_001283813.1 | MULTISPECIES: ribose-phosphate diphosphokinase | 6 | 4 | 35.1 | 14.54 | *prs2* | Cytosolic |
| WP_000418402.1 | MULTISPECIES: DNA-directed RNA polymerase subunit delta | 4 | 3 | 22.1 | 14.34 |  | Cytosolic |
| WP_000177121.1 | threonine synthase | 4 | 4 | 53.7 | 13.63 |  | Cytosolic |
| WP_000033106.1 | phosphopentomutase | 7 | 7 | 44.2 | 13.43 |  | Cytosolic |
| WP_000493842.1 | MULTISPECIES: riboflavin synthase | 6 | 5 | 23.2 | 13.39 |  | Cytosolic |
| WP_000273863.1 | MULTISPECIES: PadR family transcriptional regulator | 4 | 2 | 12.4 | 13.29 |  | Cytosolic |
| WP_000380916.1 | MULTISPECIES: GTP cyclohydrolase I FolE | 7 | 5 | 20.7 | 13.14 | *folE* | Cytosolic |
| WP_000863628.1 | MULTISPECIES: signal recognition particle protein | 8 | 8 | 57.7 | 13 | *ffh* | Cytosolic |
| WP_000411859.1 | U32 family peptidase | 4 | 3 | 35.5 | 12.98 | *rlhA* | Cytosolic |
| WP_000066732.1 | D-alanine--poly(phosphoribitol) ligase subunit DltA | 6 | 5 | 57.4 | 12.78 | *dltA* | Cytosolic |
| WP_000165444.1 | tryptophan--tRNA ligase | 6 | 4 | 38.4 | 12.74 | *trpS* | Cytosolic |
| WP_000616545.1 | MULTISPECIES: 50S ribosomal protein L14 [Bacteria] | 6 | 5 | 13 | 12.6 |  | Cytosolic |
| WP_000226001.1 | LacI family DNA-binding transcriptional regulator | 4 | 4 | 35.2 | 12.59 |  | Cytosolic |
| WP_000758382.1 | capsular polysaccharide biosynthesis protein Cps4I | 4 | 4 | 41.3 | 12.43 | *cps4I* | Cytosolic |
| WP_001017398.1 | acetyl-CoA carboxylase carboxyl transferase subunit alpha | 4 | 3 | 28.2 | 12.36 | *accA* | Cytosolic |
| WP_001291370.1 | methionine--tRNA ligase | 7 | 7 | 75.6 | 12.29 |  | Cytosolic |
| WP_000772918.1 | MULTISPECIES: 50S ribosomal protein L29 [Bacteria] | 6 | 4 | 8 | 12.12 |  | Cytosolic |
| WP_000221613.1 | DnaD domain-containing protein | 6 | 5 | 25.6 | 12.11 | *dnaD* | Cytosolic |
| WP_001274002.1 | MULTISPECIES: 30S ribosomal protein S20 | 4 | 2 | 8.5 | 12.05 |  | Cytosolic |
| WP_000591095.1 | cysteine--tRNA ligase | 5 | 5 | 51 | 11.87 |  | Cytosolic |
| WP_000488675.1 | MULTISPECIES: acetyl-CoA carboxylase biotin carboxylase subunit | 6 | 6 | 49.5 | 11.82 | *accC* | Cytosolic |
| WP_000695335.1 | capsular polysaccharide biosynthesis protein Cps4L | 6 | 5 | 44.2 | 11.72 | *cps4L* | Cytosolic |
| WP_000686416.1 | prephenate dehydratase | 6 | 5 | 31.6 | 11.72 |  | Cytosolic |
| WP_000167622.1 | MULTISPECIES: ACP S-malonyltransferase | 6 | 5 | 33.1 | 11.56 |  | Cytosolic |
| WP_000597970.1 | endonuclease/exonuclease/phosphatase family protein | 5 | 5 | 37.7 | 11.22 |  | Cytosolic |
| WP_000060169.1 | MULTISPECIES: ribosome assembly RNA-binding protein YhbY [Bacteria] | 5 | 2 | 11.7 | 11.02 | *yhbY* | Cytosolic |
| WP_000850024.1 | bifunctional pyr operon transcriptional regulator/uracil phosphoribosyltransferase PyrR | 5 | 4 | 19.6 | 11.01 | *pyrR* | Cytosolic |
| WP_000041909.1 | septum formation initiator family protein | 3 | 2 | 14.8 | 10.96 |  | Cytosolic |
| WP_000386347.1 | MULTISPECIES: MarR family transcriptional regulator | 4 | 3 | 16.7 | 10.81 | *marR* | Cytosolic |
| WP_000260012.1 | cell elongation regulator EloR | 7 | 6 | 37.1 | 10.62 |  | Cytosolic |
| WP_000699369.1 | MULTISPECIES: pyroglutamyl-peptidase I | 5 | 5 | 23.4 | 10.41 | *pcp* | Cytosolic |
| WP_001272941.1 | MULTISPECIES: peptide deformylase | 3 | 3 | 22.7 | 10.41 |  | Cytosolic |
| WP_001216856.1 | 16S rRNA (adenine(1518)-N(6)/adenine(1519)-N(6))-dimethyltransferase RsmA | 5 | 5 | 32.2 | 10.12 | *rsmA* | Cytosolic |
| WP_000799053.1 | MULTISPECIES: NAD kinase | 5 | 5 | 31 | 9.94 | *ppnK* | Cytosolic |
| WP_001269860.1 | chorismate synthase | 5 | 4 | 42.8 | 9.79 | *aroC* | Cytosolic |
| WP_001050086.1 | MULTISPECIES: DUF1149 family protein | 4 | 2 | 14.4 | 9.63 |  | Cytosolic |
| WP_001278301.1 | MULTISPECIES: HIT family protein | 6 | 3 | 15 | 9.56 |  | Cytosolic |
| WP_001293838.1 | aspartate carbamoyltransferase catalytic subunit | 5 | 5 | 34.7 | 9.38 |  | Cytosolic |
| WP_001262223.1 | ribosome recycling factor | 3 | 3 | 20.6 | 9.16 |  | Cytosolic |
| WP_000700303.1 | pneumococcal histidine triad protein PhtD | 7 | 6 | 93.6 | 9.13 | *phtD* | Cytosolic |
| WP_000754663.1 | FAD-binding oxidoreductase | 4 | 4 | 40.6 | 9.12 | *dadA* | Cytosolic |
| WP_000864220.1 | MULTISPECIES: 50S ribosomal protein L9 | 3 | 3 | 16.5 | 9.03 |  | Cytosolic |
| WP_000767464.1 | exodeoxyribonuclease III | 4 | 4 | 31 | 8.93 |  | Cytosolic |
| WP_000202452.1 | hydroxyethylthiazole kinase | 3 | 3 | 27.6 | 8.88 |  | Cytosolic |
| WP_000699501.1 | phosphopantothenate--cysteine ligase | 3 | 3 | 25.7 | 8.87 |  | Cytosolic |
| WP_000551587.1 | type II toxin-antitoxin system RelE/ParE family toxin | 5 | 4 | 14.5 | 8.73 |  | Cytosolic |
| WP_000747291.1 | 4-alpha-glucanotransferase | 4 | 4 | 58 | 8.57 |  | Cytosolic |
| WP_000078572.1 | ROK family glucokinase | 5 | 4 | 33.4 | 8.5 |  | Cytosolic |
| WP_000794247.1 | MULTISPECIES: helix-turn-helix transcriptional regulator | 3 | 3 | 10.7 | 8.4 |  | Cytosolic |
| WP_000179649.1 | type I DNA topoisomerase | 4 | 4 | 79.1 | 8.24 |  | Cytosolic |
| WP_001085703.1 | MULTISPECIES: 30S ribosomal protein S14 [Bacteria] | 4 | 2 | 10.1 | 8.23 | *rpsN* | Cytosolic |
| WP_000057241.1 | MULTISPECIES: 50S ribosomal protein L30 [Bacteria] | 3 | 3 | 6.4 | 8.12 |  | Cytosolic |
| WP_000146947.1 | MULTISPECIES: phosphocarrier protein HPr [Bacteria] | 4 | 2 | 8.9 | 8.09 |  | Cytosolic |
| WP_000697760.1 | peroxide stress protein YaaA | 3 | 2 | 27.5 | 8.09 | *yaaA* | Cytosolic |
| WP_001096229.1 | DEAD/DEAH box helicase | 5 | 4 | 118.2 | 8.08 | *hepA* | Cytosolic |
| WP_001066295.1 | MULTISPECIES: molecular chaperone DnaJ | 3 | 3 | 40.5 | 7.82 | *dnaJ* | Cytosolic |
| WP_000852948.1 | MULTISPECIES: ketoacyl-ACP synthase III | 2 | 2 | 34.9 | 7.77 |  | Cytosolic |
| WP_000163932.1 | aminoacyl-tRNA hydrolase | 4 | 3 | 21.4 | 7.74 |  | Cytosolic |
| WP_001265622.1 | MULTISPECIES: 50S ribosomal protein L33 [Bacteria] | 4 | 3 | 5.9 | 7.57 |  | Cytosolic |
| WP_001140948.1 | MULTISPECIES: 50S ribosomal protein L28 [Bacteria] | 3 | 2 | 6.9 | 7.45 | *rpmB* | Cytosolic |
| WP_000206761.1 | MULTISPECIES: carboxymuconolactone decarboxylase family protein | 4 | 4 | 19.8 | 7.32 | *yciW* | Cytosolic |
| WP_000290419.1 | MULTISPECIES: 50S ribosomal protein L32 | 2 | 2 | 6.8 | 7.32 |  | Cytosolic |
| WP_000434650.1 | thioredoxin | 2 | 2 | 12.9 | 7.3 |  | Cytosolic |
| WP_000181374.1 | tRNA (cytidine(34)-2'-O)-methyltransferase | 2 | 2 | 19.5 | 7.26 |  | Cytosolic |
| WP_001051747.1 | SsrA-binding protein SmpB | 4 | 4 | 17.7 | 7.19 | *smpB* | Cytosolic |
| WP_000795171.1 | bacterial Ig-like domain-containing protein | 4 | 4 | 183.1 | 7.14 | *yabE* | Cytosolic |
| WP_000855736.1 | 23S rRNA (guanosine(2251)-2'-O)-methyltransferase RlmB | 3 | 3 | 26.5 | 7.05 | *rlmB* | Cytosolic |
| WP_000027902.1 | 4-hydroxy-tetrahydrodipicolinate reductase | 3 | 3 | 27.8 | 7.04 | *dapB* | Cytosolic |
| WP_000201315.1 | MULTISPECIES: ribosome biogenesis GTPase YlqF | 4 | 3 | 32.1 | 6.98 | *ylpF* | Cytosolic |
| WP_000171229.1 | alpha,alpha-phosphotrehalase | 2 | 2 | 62.8 | 6.91 | *treC* | Cytosolic |
| WP_000522333.1 | signal recognition particle-docking protein FtsY | 2 | 2 | 47.8 | 6.89 | *ftsY* | Cytosolic |
| WP_000789949.1 | MULTISPECIES: LacI family transcriptional regulator | 4 | 4 | 37.6 | 6.85 | *purR* | Cytosolic |
| WP_000567914.1 | Mini-ribonuclease 3 | 2 | 2 | 14.6 | 6.75 | *mrnC* | Cytosolic |
| WP_000818763.1 | transcription elongation factor GreA | 2 | 2 | 17.6 | 6.71 | *greA* | Cytosolic |
| WP_001049323.1 | adenine phosphoribosyltransferase | 3 | 2 | 18.7 | 6.69 | *apt* | Cytosolic |
| WP_000671113.1 | DEAD/DEAH box helicase | 3 | 2 | 58.8 | 6.68 | *cshA* | Cytosolic |
| WP_001050241.1 | Mur ligase family protein | 2 | 2 | 49.6 | 6.53 | *murT* | Cytosolic |
| WP_000844505.1 | bifunctional biotin--[acetyl-CoA-carboxylase] ligase/biotin operon repressor BirA | 2 | 2 | 35.2 | 6.43 | *birA* | Cytosolic |
| WP_000979235.1 | MULTISPECIES: DNA-directed RNA polymerase subunit omega | 2 | 2 | 11.9 | 6.37 |  | Cytosolic |
| WP_001234978.1 | MULTISPECIES: RNA-binding S4 domain-containing protein | 2 | 2 | 10.1 | 6.35 |  | Cytosolic |
| WP_000137358.1 | MULTISPECIES: dihydroxy-acid dehydratase | 2 | 2 | 59.8 | 6.29 | *ilvD* | Cytosolic |
| WP_000134039.1 | DNA topoisomerase (ATP-hydrolyzing) subunit B | 3 | 3 | 72.2 | 6.24 | *gyrB* | Cytosolic |
| WP_000711388.1 | class I SAM-dependent RNA methyltransferase | 2 | 2 | 43.2 | 6.23 |  | Cytosolic |
| WP_000818547.1 | MULTISPECIES: cell division protein FtsL | 3 | 2 | 12.2 | 6.21 | *ftsL* | Cytosolic |
| WP_000568640.1 | MULTISPECIES: elongation factor P [Bacteria] | 3 | 2 | 20.6 | 6.16 | *efp* | Cytosolic |
| WP_000331293.1 | tryptophan synthase subunit beta | 3 | 3 | 44.2 | 5.93 | *trpB* | Cytosolic |
| WP_000219939.1 | MULTISPECIES: DegV family protein | 2 | 2 | 30.6 | 5.86 |  | Cytosolic |
| WP_001036519.1 | MULTISPECIES: DNA-directed RNA polymerase subunit beta [Bacteria] | 2 | 2 | 6.8 | 5.76 |  | Cytosolic |
| WP_000222054.1 | peptidase T | 2 | 2 | 44.8 | 5.75 | *pepT* | Cytosolic |
| WP_000689766.1 | 5'-methylthioadenosine/adenosylhomocysteine nucleosidase | 2 | 2 | 24.7 | 5.75 |  | Cytosolic |
| WP_000863037.1 | UDP-N-acetylmuramoyl-L-alanine--D-glutamate ligase | 3 | 2 | 48.5 | 5.74 | *murD* | Cytosolic |
| WP_001219120.1 | LytTR family DNA-binding domain-containing protein | 2 | 2 | 28.7 | 5.74 | *lytTR* | Cytosolic |
| WP_000845295.1 | formate--tetrahydrofolate ligase | 2 | 2 | 59.6 | 5.66 |  | Cytosolic |
| WP_001025729.1 | LysR family transcriptional regulator | 2 | 2 | 35 | 5.61 | *lysR* | Cytosolic |
| WP_000922204.1 | MULTISPECIES: sugar transferase | 2 | 2 | 26.8 | 5.59 |  | Cytosolic |
| WP_001063405.1 | ATP-dependent Clp protease ATP-binding subunit | 2 | 2 | 77.6 | 5.57 | *clpA* | Cytosolic |
| WP_000958770.1 | MULTISPECIES: ATP-binding cassette domain-containing protein | 2 | 2 | 60.8 | 5.23 |  | Cytosolic |
| WP_000521411.1 | phosphoglucosamine mutase | 3 | 3 | 48.1 | 5.18 | *glmM* | Cytosolic |
| WP_001284128.1 | MULTISPECIES: phosphopantothenoylcysteine decarboxylase | 3 | 2 | 19.9 | 5.18 | *coaC* | Cytosolic |
| WP_001152899.1 | excinuclease ABC subunit UvrA | 4 | 4 | 104.1 | 5.07 | *uvrA* | Cytosolic |
| WP_001217820.1 | glycerol dehydrogenase | 2 | 2 | 39.1 | 5.06 | *gldA* | Cytosolic |
| WP_001126999.1 | tryptophan synthase subunit alpha | 2 | 2 | 27.7 | 5.02 | *trpA* | Cytosolic |
| WP_000053388.1 | cell division protein SepF | 2 | 2 | 20.6 | 5.01 | *sepF* | Cytosolic |
| WP_001166880.1 | 16S rRNA (cytidine(1402)-2'-O)-methyltransferase | 2 | 2 | 32.4 | 4.9 |  | Cytosolic |
| WP_000073428.1 | MULTISPECIES: IMP dehydrogenase | 3 | 3 | 52.4 | 4.88 | *guaB* | Cytosolic |
| WP_000856519.1 | diaminopimelate decarboxylase | 3 | 2 | 46.5 | 4.82 |  | Cytosolic |
| WP_000163693.1 | methionyl-tRNA formyltransferase | 3 | 2 | 34 | 4.8 |  | Cytosolic |
| WP_000359134.1 | RNA-binding transcriptional accessory protein | 4 | 4 | 78.7 | 4.76 |  | Cytosolic |
| WP_000164779.1 | glycine--tRNA ligase subunit beta | 3 | 3 | 75.4 | 4.72 | *glyS* | Cytosolic |
| WP_000339018.1 | MULTISPECIES: bifunctional Cof-type HAD-IIB family hydrolase/peptidylprolyl isomerase | 4 | 4 | 52 | 4.66 | *ppiB* | Cytosolic |
| WP_000379621.1 | MULTISPECIES: RNA-binding protein KphA | 2 | 2 | 9 | 4.63 | *kphA* | Cytosolic |
| WP_000850585.1 | DEAD/DEAH box helicase | 2 | 2 | 41.3 | 4.62 | *srmB* | Cytosolic |
| WP_000290025.1 | esterase family protein | 2 | 2 | 29.9 | 4.56 | *frmB* | Cytosolic |
| WP_000593569.1 | Cof-type HAD-IIB family hydrolase | 2 | 2 | 30 | 4.56 |  | Cytosolic |
| WP_000565519.1 | 3-hydroxyacyl-ACP dehydratase FabZ | 4 | 4 | 15.3 | 4.53 | *fabZ* | Cytosolic |
| WP_001028831.1 | peptide chain release factor 1 | 3 | 3 | 40.6 | 4.48 | *prfA* | Cytosolic |
| WP_000143265.1 | MULTISPECIES: GTPase Era | 3 | 3 | 34 | 4.46 |  | Cytosolic |
| WP_001085462.1 | MULTISPECIES: recombinase RecA | 3 | 3 | 41.9 | 4.44 | *recA* | Cytosolic |
| WP_000003934.1 | methionine adenosyltransferase | 2 | 2 | 43.1 | 4.36 | *metK* | Cytosolic |
| WP_001125943.1 | MULTISPECIES: 50S ribosomal protein L35 [Terrabacteria group] | 5 | 3 | 7.8 | 4.35 | *rpmI* | Cytosolic |
| WP_000802934.1 | 16S rRNA (guanine(527)-N(7))-methyltransferase RsmG | 2 | 2 | 27.2 | 4.35 | *rsmG* | Cytosolic |
| WP_000038726.1 | glycine--tRNA ligase subunit alpha | 2 | 2 | 34.9 | 4.33 | *glyQ* | Cytosolic |
| WP_000153039.1 | Cof-type HAD-IIB family hydrolase | 2 | 2 | 29.6 | 4.2 |  | Cytosolic |
| WP_000936166.1 | GntR family transcriptional regulator | 2 | 2 | 26.8 | 4.07 | *mngR* | Cytosolic |
| WP_000161399.1 | DegV family protein | 2 | 2 | 30.6 | 4.03 | *degV* | Cytosolic |
| WP_000283118.1 | nicotinate phosphoribosyltransferase | 2 | 2 | 55.1 | 4.03 |  | Cytosolic |
| WP_000263194.1 | MULTISPECIES: type 1 glutamine amidotransferase | 2 | 2 | 29.2 | 3.76 | *gatD* | Cytosolic |
| WP_000400073.1 | MULTISPECIES: enoyl-CoA hydratase | 2 | 2 | 28.7 | 3.73 |  | Cytosolic |
| WP_000992862.1 | DNA helicase PcrA | 2 | 2 | 85.9 | 3.66 | *pcrA* | Cytosolic |
| WP_001126406.1 | carbamoyl-phosphate synthase large subunit | 4 | 4 | 116 | 3.64 | *carB* | Cytosolic |
| WP_001284361.1 | cholesterol-dependent cytolysin pneumolysin | 2 | 2 | 52.9 | 3.63 | *ply* | Cytosolic |
| WP_000171676.1 | MULTISPECIES: GTP pyrophosphokinase family protein | 3 | 3 | 26.2 | 3.45 | *accD* | Cytosolic |
| WP_000924508.1 | MULTISPECIES: dihydroorotase | 2 | 2 | 45.3 | 3.43 | *pyrC* | Cytosolic |
| WP_001264338.1 | capsular polysaccharide biosynthesis protein Cps4G | 2 | 2 | 42.6 | 3.34 |  | Cytosolic |
| WP_000444687.1 | DNA recombination protein RmuC | 3 | 3 | 47.9 | 3.28 | *rmuC* | Cytosolic |
| WP_000587447.1 | bifunctional riboflavin kinase/FAD synthetase | 3 | 3 | 34.5 | 3.25 |  | Cytosolic |
| WP_000665420.1 | MULTISPECIES: PadR family transcriptional regulator | 3 | 3 | 12.2 | 3.21 |  | Cytosolic |
| WP_000022084.1 | purine-nucleoside phosphorylase | 2 | 2 | 26.1 | 3.21 | *deoD* | Cytosolic |
| WP_000560886.1 | anaerobic ribonucleoside-triphosphate reductase | 2 | 2 | 83.7 | 2.94 | *nrdD* | Cytosolic |
| WP_000011009.1 | DEAD/DEAH box helicase | 2 | 2 | 50.7 | 2.83 | *cshB* | Cytosolic |
| WP_001203672.1 | MULTISPECIES: transcriptional regulator NrdR | 2 | 2 | 18.4 | 2.8 | *nrdR* | Cytosolic |
| WP_000815629.1 | MULTISPECIES: magnesium transporter CorA family protein | 3 | 3 | 36.4 | 2.79 | *engB* | Cytosolic |
| WP_000164302.1 | ROK family protein | 2 | 2 | 31.9 | 2.78 | *scrK* | Cytosolic |
| WP_000023507.1 | MULTISPECIES: sugar-phosphatase | 2 | 2 | 29.7 | 2.66 | *yidA* | Cytosolic |
| WP_000004139.1 | MULTISPECIES: TetR/AcrR family transcriptional regulator | 2 | 2 | 21.7 | 2.57 |  | Cytosolic |
| WP_000867616.1 | DEAD/DEAH box helicase | 2 | 2 | 49.6 | 2.45 |  | Cytosolic |
| WP_000159169.1 | MULTISPECIES: transcription repressor NadR | 2 | 2 | 19.7 | 2.44 | *nadR* | Cytosolic |
| WP_000219018.1 | branched-chain amino acid aminotransferase | 2 | 2 | 37.5 | 2.43 |  | Cytosolic |
| WP_000411215.1 | choline kinase LicA | 2 | 2 | 33.4 | 2.34 | *pck* | Cytosolic |
| WP_000453137.1 | Cof-type HAD-IIB family hydrolase | 2 | 2 | 51.9 | 2.14 |  | Cytosolic |
| WP_000638907.1 | MULTISPECIES: cysteine desulfurase | 3 | 3 | 41.7 | 2.13 | *nifC* | Cytosolic |
| WP_000643971.1 | NTP transferase domain-containing protein | 2 | 2 | 26.9 | 2.01 | *licC* | Cytosolic |
| WP_001042585.1 | NAD-dependent DNA ligase LigA | 2 | 2 | 72.1 | 0 | *ligA* | Cytosolic |
| WP_000704390.1 | hydroxymethylglutaryl-CoA reductase, degradative | 2 | 2 | 46.2 | 0 |  | Cytosolic |
| WP_000661480.1 | MULTISPECIES: ribonuclease III | 2 | 2 | 26.2 | 0 |  | Cytosolic |
| WP_001036132.1 | BMP family protein | 850 | 46 | 36.7 | 2942.23 | *pnrA* | Lipoproteins |
| WP_000733059.1 | metal ABC transporter substrate-binding lipoprotein/adhesin PsaA | 776 | 36 | 34.6 | 1938.03 | *psaA* | Lipoproteins |
| WP_000095467.1 | extracellular solute-binding protein | 473 | 46 | 45.3 | 1633.5 | *malX* | Lipoproteins |
| WP_000727952.1 | MULTISPECIES: peptidylprolyl isomerase PrsA | 489 | 43 | 34.4 | 1497.01 | *prsA* | Lipoproteins |
| WP_000672121.1 | ABC transporter substrate-binding protein | 290 | 31 | 48.3 | 852.78 | *satA* | Lipoproteins |
| WP_000790743.1 | MULTISPECIES: iron-siderophore ABC transporter substrate-binding protein | 228 | 37 | 37.5 | 634.62 | *PiuA* | Lipoproteins |
| WP_000742235.1 | peptide ABC transporter substrate-binding protein | 191 | 42 | 72.4 | 541.67 | *amiA* | Lipoproteins |
| WP_000726149.1 | MULTISPECIES: ABC transporter substrate-binding protein | 168 | 31 | 40.4 | 503.06 | *livJ* | Lipoproteins |
| WP_000747771.1 | M15 family metallopeptidase | 147 | 17 | 26.4 | 434.16 | *dacB* | Lipoproteins |
| WP_000858264.1 | siderophore ABC transporter substrate-binding protein | 139 | 36 | 34.8 | 403.14 | *piaA* | Lipoproteins |
| WP_000842584.1 | peptide ABC transporter substrate-binding protein | 144 | 51 | 73 | 398.75 | *aliA* | Lipoproteins |
| WP_000754546.1 | MULTISPECIES: ABC transporter substrate-binding protein | 121 | 30 | 37.8 | 367.26 |  | Lipoproteins |
| WP_000759187.1 | amino acid ABC transporter substrate-binding protein | 140 | 34 | 31 | 330.91 | *aatB* | Lipoproteins |
| WP_000724932.1 | amino acid ABC transporter substrate-binding protein | 109 | 28 | 30.6 | 325.71 |  | Lipoproteins |
| WP_000837349.1 | ABC transporter substrate-binding protein | 101 | 29 | 29.3 | 300.05 |  | Lipoproteins |
| WP_000748873.1 | peptide ABC transporter substrate-binding protein | 98 | 41 | 72.5 | 261.31 | *aliB* | Lipoproteins |
| WP_000731916.1 | peptidylprolyl isomerase | 85 | 24 | 29.1 | 249.49 | *SlrA* | Lipoproteins |
| WP_000694504.1 | MetQ/NlpA family ABC transporter substrate-binding protein | 92 | 27 | 31.2 | 246.97 | *metQ* | Lipoproteins |
| WP_000724057.1 | zinc ABC transporter substrate-binding lipoprotein AdcA | 65 | 29 | 56.2 | 161.45 | *adcA* | Lipoproteins |
| WP_000746338.1 | phosphate ABC transporter substrate-binding protein PstS | 55 | 15 | 31.2 | 159.37 | *pstS1* | Lipoproteins |
| WP_000738366.1 | extracellular solute-binding protein | 45 | 25 | 39.2 | 131.91 |  | Lipoproteins |
| WP_001267410.1 | MULTISPECIES: M57 family metalloprotease | 33 | 10 | 26.7 | 96.02 |  | Lipoproteins |
| WP_000800407.1 | MULTISPECIES: ABC transporter substrate-binding protein | 34 | 18 | 54.5 | 87.6 |  | Lipoproteins |
| WP_000757369.1 | thiol-disulfide oxidoreductase-associated lipoprotein SdbB | 27 | 11 | 20.7 | 70.89 | *Etrx2* | Lipoproteins |
| WP_000725133.1 | hypothetical protein | 20 | 11 | 21 | 45.91 |  | Lipoproteins |
| WP_000749601.1 | DUF4300 family protein | 18 | 7 | 32.7 | 36.31 |  | Lipoproteins |
| WP_000455420.1 | extracellular solute-binding protein | 13 | 11 | 46.6 | 31.65 | *rafE* | Lipoproteins |
| WP_000283071.1 | type I pullulanase | 12 | 10 | 86.5 | 21.91 |  | Lipoproteins |
| WP_000738763.1 | sugar ABC transporter substrate-binding protein | 5 | 5 | 49.6 | 17.58 |  | Lipoproteins |
| WP_001838232.1 | SP_0198 family lipoprotein | 3 | 3 | 19.9 | 6.09 |  | Lipoproteins |
| WP_001227736.1 | DUF1002 domain-containing protein | 136 | 29 | 35.4 | 400.62 |  | Secreted proteins |
| WP_000792163.1 | ABC transporter substrate-binding protein | 79 | 20 | 35.1 | 247.81 |  | Secreted proteins |
| WP_001078983.1 | MULTISPECIES: rod shape-determining protein MreC | 48 | 21 | 29.7 | 154.23 | *mreC* | Secreted proteins |
| WP_000719158.1 | D-alanyl-D-alanine carboxypeptidase PBP3 | 45 | 20 | 45.2 | 134.23 | *dacA* | Secreted proteins |
| WP_000681597.1 | trypsin-like peptidase domain-containing protein | 35 | 18 | 41.8 | 105 |  | Secreted proteins |
| WP_000239283.1 | MULTISPECIES: LCP family protein | 50 | 19 | 37.5 | 101.4 |  | Secreted proteins |
| WP_000902984.1 | MULTISPECIES: ABC transporter permease subunit Vex3 | 35 | 20 | 50 | 94.64 |  | Secreted proteins |
| WP_000727012.1 | MULTISPECIES: CHAP domain-containing protein | 26 | 18 | 41.7 | 87.64 | *usp45* | Secreted proteins |
| WP_001230165.1 | lactococcin 972 family bacteriocin | 6 | 2 | 10.5 | 8.92 |  | Secreted proteins |
| WP_001156825.1 | alpha-glucosidase | 3 | 3 | 62 | 8.79 |  | Secreted proteins |
| WP_001291663.1 | endolytic transglycosylase MltG | 221 | 54 | 60.8 | 626.67 | *mltG* | transmembrane |
| WP_001040013.1 | penicillin-binding protein PBP1A | 208 | 47 | 79.7 | 578.82 | *ponA* | transmembrane |
| WP_000872275.1 | penicillin-binding protein 2X | 169 | 46 | 82.3 | 467.07 | *pbpX* | transmembrane |
| WP_001808499.1 | polysaccharide deacetylase family protein | 127 | 41 | 52.7 | 368.82 | *pgdA* | transmembrane |
| WP_001829432.1 | penicillin-binding protein 2B | 102 | 38 | 74.4 | 278.39 | *penA* | transmembrane |
| WP_000032347.1 | MULTISPECIES: polyisoprenyl-teichoic acid--peptidoglycan teichoic acid transferase Psr | 94 | 32 | 47.7 | 257.12 |  | transmembrane |
| WP_000728641.1 | efflux RND transporter periplasmic adaptor subunit | 90 | 28 | 41.8 | 249.95 |  | transmembrane |
| WP_000726272.1 | ABC transporter substrate-binding protein/permease | 71 | 31 | 78.3 | 197.41 |  | transmembrane |
| WP_000664160.1 | capsular polysaccharide biosynthesis protein CpsC | 43 | 16 | 25.4 | 151.9 | *cpsC* | transmembrane |
| WP_000759901.1 | MULTISPECIES: ABC transporter permease | 63 | 20 | 55.6 | 151.76 | *amiC* | transmembrane |
| WP_000744538.1 | ATP-dependent zinc metalloprotease FtsH | 54 | 33 | 71.3 | 143.54 | *ftsH* | transmembrane |
| WP_000836217.1 | PI-1 pilus major pilin RrgB | 47 | 28 | 71.3 | 134.2 |  | transmembrane |
| WP_000136861.1 | MULTISPECIES: PTS system mannose/fructose/sorbose family transporter subunit IID | 40 | 12 | 33.8 | 124.49 |  | transmembrane |
| WP_000757844.1 | PI-1 pilus tip adhesin RrgA | 45 | 30 | 98.8 | 119.62 | *rrgA* | transmembrane |
| WP_000762628.1 | penicillin-binding protein PBP2A | 47 | 31 | 80.8 | 117.3 | *pbp2A* | transmembrane |
| WP_001820908.1 | signal peptidase I | 63 | 16 | 23.4 | 115.82 | *lepB* | transmembrane |
| WP_000091082.1 | capsular polysaccharide biosynthesis protein Cps4A | 43 | 20 | 53.5 | 110.85 | *cps4A* | transmembrane |
| WP_001233696.1 | DUF4097 domain-containing protein | 35 | 16 | 34.2 | 98.75 |  | transmembrane |
| WP_000900658.1 | RIP metalloprotease RseP | 34 | 23 | 45.9 | 98.35 |  | transmembrane |
| WP_000032460.1 | cation-translocating P-type ATPase | 33 | 21 | 97 | 97.26 |  | transmembrane |
| WP_000241147.1 | PI-1 pilus system sortase SrtC-1 | 33 | 12 | 34.1 | 96.86 |  | transmembrane |
| WP_000461505.1 | hypothetical protein | 35 | 20 | 38.7 | 91.76 |  | transmembrane |
| WP_001227315.1 | ABC transporter substrate-binding protein/permease | 27 | 13 | 57.4 | 89.21 |  | transmembrane |
| WP_000039291.1 | mid-cell-anchored protein MapZ | 29 | 18 | 51.5 | 87.71 | *mapZ* | transmembrane |
| WP_000370514.1 | PI-1 pilus system sortase SrtC-2 | 25 | 13 | 34.2 | 86.35 | *srtC* | transmembrane |
| WP_001036779.1 | DNA-entry nuclease EndA | 28 | 13 | 29.9 | 84.46 | *endA* | transmembrane |
| WP_000182964.1 | ABC transporter permease/substrate-binding protein | 32 | 19 | 55.5 | 81.51 | *proWX* | transmembrane |
| WP_000822867.1 | hypothetical protein | 28 | 23 | 59.4 | 79.01 |  | transmembrane |
| WP_000625536.1 | MULTISPECIES: permease-like cell division protein FtsX | 27 | 13 | 34.3 | 67.5 | *ftsX* | transmembrane |
| WP_000614538.1 | Stk1 family PASTA domain-containing Ser/Thr kinase | 25 | 20 | 72.2 | 65.22 | *stkP* | transmembrane |
| WP_001040907.1 | hypothetical protein | 21 | 4 | 16.8 | 59.4 |  | transmembrane |
| WP_001077206.1 | M42 family metallopeptidase | 21 | 14 | 38 | 57.37 |  | transmembrane |
| WP_001180981.1 | penicillin-binding protein PBP1B | 25 | 22 | 89.5 | 56.39 | *pbp1B* | transmembrane |
| WP_000808443.1 | membrane protein | 19 | 9 | 40.3 | 54.13 |  | transmembrane |
| WP_000359036.1 | MULTISPECIES: F0F1 ATP synthase subunit delta | 21 | 12 | 20.5 | 51.36 | *atpH* | transmembrane |
| WP_000514307.1 | PTS system trehalose-specific EIIBC component | 19 | 11 | 69.7 | 51.29 |  | transmembrane |
| WP_000974062.1 | PTS transporter subunit IIBC | 14 | 9 | 77.9 | 47.59 | *exp5* | transmembrane |
| WP_000669493.1 | MULTISPECIES: phosphate-binding protein | 15 | 11 | 30.7 | 46.01 | *pstS2* | transmembrane |
| WP_000259256.1 | MULTISPECIES: rhodanese-like domain-containing protein | 21 | 8 | 14.6 | 44 |  | transmembrane |
| WP_000703352.1 | FtsW/RodA/SpoVE family cell cycle protein | 16 | 7 | 45.4 | 43.46 |  | transmembrane |
| WP_000078847.1 | sortase SrtA | 18 | 12 | 28.1 | 43.03 | *srtA_1* | transmembrane |
| WP_000360054.1 | cation-translocating P-type ATPase | 17 | 11 | 85.5 | 42.67 | *exp7* | transmembrane |
| WP_000173753.1 | ABC transporter ATP-binding subunit Vex2 | 13 | 9 | 23.8 | 41.62 | *vex2* | transmembrane |
| WP_000219823.1 | MULTISPECIES: LemA family protein | 16 | 9 | 20.6 | 41.26 | *lemA* | transmembrane |
| WP_000356590.1 | ABC transporter ATP-binding protein | 17 | 9 | 27.1 | 41.21 |  | transmembrane |
| WP_000091735.1 | PI-1 pilus system sortase SrtC-3 | 18 | 5 | 31.9 | 39.74 |  | transmembrane |
| WP_000834688.1 | YfhO family protein | 19 | 13 | 97.2 | 34.98 |  | transmembrane |
| WP_000348128.1 | aquaporin family protein | 11 | 7 | 30.7 | 33.44 |  | transmembrane |
| WP_000701427.1 | fructose-specific PTS transporter subunit EIIC | 15 | 10 | 66.9 | 33.19 |  | transmembrane |
| WP_000974212.1 | iron-containing alcohol dehydrogenase | 10 | 8 | 41.2 | 31.44 |  | transmembrane |
| WP_000103700.1 | MULTISPECIES: ABC transporter permease | 10 | 5 | 34.6 | 28.95 | *amiD* | transmembrane |
| WP_000031159.1 | cell division protein DivIB | 10 | 6 | 45.8 | 28.87 | *divIB* | transmembrane |
| WP_000068050.1 | MULTISPECIES: F0F1 ATP synthase subunit epsilon | 10 | 8 | 15.6 | 28.71 | *atpC* | transmembrane |
| WP_001231235.1 | glycoside hydrolase family 25 protein | 10 | 8 | 30 | 28.68 | *lytC* | transmembrane |
| WP_000729030.1 | hypothetical protein | 8 | 3 | 19.8 | 28.02 |  | transmembrane |
| WP_000078022.1 | MULTISPECIES: DUF1129 domain-containing protein | 8 | 4 | 25.8 | 26.09 |  | transmembrane |
| WP_000558554.1 | MULTISPECIES: F0F1 ATP synthase subunit B | 12 | 8 | 18 | 25.93 | *atpF* | transmembrane |
| WP_010963212.1 | capsular polysaccharide biosynthesis protein Cps4E | 11 | 7 | 24 | 25.85 | *cps4E* | transmembrane |
| WP_000835955.1 | MULTISPECIES: membrane protein insertase YidC | 9 | 6 | 34.1 | 25.79 | *yidC1* | transmembrane |
| WP_001180425.1 | ABC transporter permease | 9 | 8 | 45.3 | 24.8 |  | transmembrane |
| WP_000364990.1 | MULTISPECIES: YneF family protein [Bacteria] | 10 | 6 | 9.1 | 24.76 |  | transmembrane |
| WP_000038659.1 | MULTISPECIES: ABC transporter permease | 8 | 5 | 37.4 | 24.73 |  | transmembrane |
| WP_000617836.1 | TIGR03943 family protein | 7 | 5 | 31.1 | 24.22 |  | transmembrane |
| WP_001035700.1 | MULTISPECIES: polysaccharide biosynthesis protein | 9 | 8 | 69.2 | 24.09 |  | transmembrane |
| WP_001068289.1 | ABC transporter permease | 10 | 10 | 46.5 | 23.6 | *vex1* | transmembrane |
| WP_000677519.1 | ABC transporter ATP-binding protein | 6 | 5 | 29.6 | 22.49 |  | transmembrane |
| WP_000185298.1 | MULTISPECIES: PTS system mannose/fructose/sorbose family transporter subunit IID | 12 | 5 | 30 | 22.06 |  | transmembrane |
| WP_000189477.1 | MULTISPECIES: amino acid ABC transporter ATP-binding protein | 7 | 7 | 27.9 | 22.02 |  | transmembrane |
| WP_000811482.1 | MULTISPECIES: ABC transporter ATP-binding protein | 8 | 6 | 25.7 | 21.77 |  | transmembrane |
| WP_001016917.1 | Bax inhibitor-1/YccA family protein | 17 | 4 | 24.7 | 21.48 | *ybhL* | transmembrane |
| WP_000714614.1 | amino acid ABC transporter substrate-binding protein | 10 | 5 | 29.7 | 21.2 |  | transmembrane |
| WP_001192007.1 | MULTISPECIES: carbohydrate ABC transporter permease | 10 | 4 | 31.2 | 21.2 |  | transmembrane |
| WP_000727277.1 | MULTISPECIES: PDZ domain-containing protein | 7 | 4 | 37.5 | 20.35 |  | transmembrane |
| WP_000170915.1 | orotate phosphoribosyltransferase | 6 | 5 | 22.8 | 20.32 |  | transmembrane |
| WP_000889924.1 | ABC transporter ATP-binding protein | 9 | 7 | 27 | 20 |  | transmembrane |
| WP_000500194.1 | 1-acyl-sn-glycerol-3-phosphate acyltransferase | 9 | 8 | 28.9 | 19.85 |  | transmembrane |
| WP_000338731.1 | PTS galactitol transporter subunit IIC | 6 | 4 | 53 | 19.33 |  | transmembrane |
| WP_000022879.1 | ABC transporter permease | 5 | 5 | 33.7 | 18.66 |  | transmembrane |
| WP_000008792.1 | heavy metal translocating P-type ATPase | 9 | 9 | 77.6 | 17.42 |  | transmembrane |
| WP_000825267.1 | bacteriocin-associated integral membrane family protein | 7 | 6 | 75.4 | 17.41 |  | transmembrane |
| WP_000724262.1 | bacteriocin-associated integral membrane family protein | 7 | 7 | 80.7 | 17.26 |  | transmembrane |
| WP_000745389.1 | signal peptidase II | 6 | 2 | 17.1 | 16.65 |  | transmembrane |
| WP_000136830.1 | SPFH domain-containing protein | 8 | 7 | 36.3 | 16.12 |  | transmembrane |
| WP_001038481.1 | ABC transporter substrate-binding protein | 6 | 5 | 29.2 | 15.97 |  | transmembrane |
| WP_000280773.1 | undecaprenyl-diphosphate phosphatase | 4 | 2 | 31.8 | 14.94 |  | transmembrane |
| WP_000114489.1 | MULTISPECIES: Fe-S cluster assembly ATPase SufC | 6 | 5 | 28.4 | 14.62 |  | transmembrane |
| WP_001220853.1 | acetyltransferase | 4 | 4 | 67.2 | 13.59 |  | transmembrane |
| WP_000891470.1 | sodium:alanine symporter family protein | 5 | 3 | 46.6 | 13.51 | *alsT* | transmembrane |
| WP_000719713.1 | site-2 protease family protein | 4 | 3 | 41.1 | 13.02 |  | transmembrane |
| WP_000565352.1 | capsular polysaccharide biosynthesis protein Cps4B | 5 | 5 | 28.1 | 13.01 |  | transmembrane |
| WP_000670146.1 | nitroreductase family protein | 7 | 4 | 22.7 | 12.97 |  | transmembrane |
| WP_000414968.1 | MULTISPECIES: ABC transporter permease subunit | 6 | 2 | 47.7 | 12.91 | *ugpA* | transmembrane |
| WP_001103449.1 | MULTISPECIES: amino acid ABC transporter permease | 7 | 4 | 28.9 | 12.75 |  | transmembrane |
| WP_000470785.1 | MULTISPECIES: phospho-N-acetylmuramoyl-pentapeptide-transferase | 7 | 4 | 36.1 | 12.65 | *mraY* | transmembrane |
| WP_000908145.1 | multidrug efflux ABC transporter subunit PatA | 7 | 7 | 62.1 | 12.63 |  | transmembrane |
| WP_001290381.1 | YfcC family protein | 4 | 3 | 54 | 12.31 | *yfcC* | transmembrane |
| WP_000520411.1 | cell wall synthase accessory phosphoprotein MacP | 5 | 4 | 11.8 | 12.13 |  | transmembrane |
| WP_000830659.1 | MULTISPECIES: FtsW/RodA/SpoVE family cell cycle protein | 5 | 4 | 45.2 | 11.49 |  | transmembrane |
| WP_000886660.1 | prolipoprotein diacylglyceryl transferase | 5 | 5 | 30.3 | 11.22 | *lgt* | transmembrane |
| WP_001218714.1 | redox-regulated ATPase YchF | 4 | 4 | 41.1 | 11.18 | *ychF* | transmembrane |
| WP_000616999.1 | MULTISPECIES: PTS mannose/fructose/sorbose/N-acetylgalactosamine transporter subunit IIC | 7 | 4 | 31.9 | 11.14 | *manY* | transmembrane |
| WP_001813450.1 | NCS2 family nucleobase:cation symporter | 3 | 3 | 44.8 | 10.69 | *uraA* | transmembrane |
| WP_001011637.1 | amino acid ABC transporter permease | 4 | 3 | 24.9 | 10.23 | *hisM* | transmembrane |
| WP_000429499.1 | MULTISPECIES: membrane protein | 4 | 4 | 15.1 | 10.16 |  | transmembrane |
| WP_000748394.1 | MptD family putative ECF transporter S component | 5 | 3 | 20.1 | 9.85 |  | transmembrane |
| WP_000064814.1 | septation ring formation regulator EzrA | 4 | 4 | 66.5 | 9.77 | *ezrA* | transmembrane |
| WP_000009144.1 | YeiH family protein | 3 | 2 | 36 | 9.64 | *yeiH* | transmembrane |
| WP_001220357.1 | YbaN family protein | 4 | 2 | 13.7 | 9.63 |  | transmembrane |
| WP_000655960.1 | ABC transporter permease | 3 | 3 | 45 | 9.4 | *yadH* | transmembrane |
| WP_000565078.1 | O-antigen polysaccharide polymerase Wzy family protein | 4 | 3 | 54.8 | 9.26 |  | transmembrane |
| WP_000801715.1 | glycerophosphoryl diester phosphodiesterase membrane domain-containing protein | 6 | 6 | 68.9 | 9.1 |  | transmembrane |
| WP_000835623.1 | O-antigen ligase family protein | 3 | 2 | 45.2 | 8.74 |  | transmembrane |
| WP_000742913.1 | MULTISPECIES: ABC transporter ATP-binding protein | 3 | 3 | 44.1 | 7.98 |  | transmembrane |
| WP_001269474.1 | metal ABC transporter ATP-binding protein | 2 | 2 | 26.5 | 7.83 |  | transmembrane |
| WP_000489362.1 | ABC transporter permease | 3 | 3 | 74.3 | 7.71 |  | transmembrane |
| WP_000022715.1 | PTS mannose/fructose/sorbose transporter subunit IIC | 3 | 2 | 27.2 | 7.71 |  | transmembrane |
| WP_001193768.1 | O-antigen polysaccharide polymerase Wzy | 5 | 3 | 53.8 | 7.68 | *wzy* | transmembrane |
| WP_000727904.1 | MULTISPECIES: membrane protein insertase YidC | 4 | 3 | 31 | 7.64 | *yidC* | transmembrane |
| WP_000571329.1 | ABC transporter ATP-binding protein | 4 | 4 | 23.6 | 7.63 |  | transmembrane |
| WP_000244116.1 | oligoendopeptidase F | 5 | 5 | 69.8 | 7.5 | *pepF* | transmembrane |
| WP_000565531.1 | MULTISPECIES: ABC transporter ATP-binding protein | 3 | 3 | 23.9 | 7.48 |  | transmembrane |
| WP_001245351.1 | ABC transporter permease | 4 | 4 | 43.9 | 7.32 | *natB* | transmembrane |
| WP_000136310.1 | heavy metal translocating P-type ATPase | 4 | 4 | 80.5 | 7.19 |  | transmembrane |
| WP_000033948.1 | Pr6Pr family membrane protein | 3 | 3 | 24.6 | 6.99 |  | transmembrane |
| WP_001011058.1 | MULTISPECIES: diadenylate cyclase CdaA | 2 | 2 | 31.8 | 6.29 | *cdaA* | transmembrane |
| WP_000465397.1 | MULTISPECIES: preprotein translocase subunit SecY | 3 | 2 | 47.3 | 6.24 | *secY* | transmembrane |
| WP_000076183.1 | TVP38/TMEM64 family protein | 3 | 2 | 23.3 | 6.09 |  | transmembrane |
| WP_000510412.1 | energy-coupling factor transporter ATPase | 3 | 2 | 30.5 | 5.98 |  | transmembrane |
| WP_001065636.1 | MULTISPECIES: sugar ABC transporter permease | 3 | 3 | 31.5 | 5.94 | *malG* | transmembrane |
| WP_000895734.1 | zinc metalloprotease HtpX | 3 | 2 | 32.8 | 5.86 | *htpX* | transmembrane |
| WP_000725682.1 | ABC transporter substrate-binding protein | 4 | 3 | 41 | 5.64 |  | transmembrane |
| WP_000725749.1 | membrane protein | 2 | 2 | 24.9 | 5.46 | *dsbD* | transmembrane |
| WP_000859866.1 | multidrug efflux ABC transporter subunit PatB | 2 | 2 | 65.6 | 5.37 | *patB* | transmembrane |
| WP_001002570.1 | ABC transporter ATP-binding protein | 3 | 3 | 23.8 | 5.1 | *ccmA* | transmembrane |
| WP_000377893.1 | MULTISPECIES: energy-coupling factor transporter transmembrane protein EcfT | 3 | 3 | 29.5 | 5.08 | *ecfT* | transmembrane |
| WP_001225550.1 | MULTISPECIES: energy coupling factor transporter S component ThiW | 2 | 2 | 19.2 | 5.01 | *thiW* | transmembrane |
| WP_001210991.1 | MULTISPECIES: preprotein translocase subunit SecE [Bacteria] | 2 | 2 | 7.1 | 4.84 | *secE* | transmembrane |
| WP_000709032.1 | biotin transporter BioY | 3 | 2 | 18.6 | 4.47 | *bioY* | transmembrane |
| WP_000017791.1 | polysaccharide biosynthesis protein | 2 | 2 | 60.1 | 4.39 | *spoVB* | transmembrane |
| WP_000435455.1 | AI-2E family transporter | 2 | 2 | 43.3 | 4.09 | *perM* | transmembrane |
| WP_000392877.1 | threonine/serine exporter ThrE family protein | 2 | 2 | 27.2 | 4.02 | *thrE* | transmembrane |
| WP_000835715.1 | energy-coupling factor transporter ATPase | 2 | 2 | 31.1 | 3.95 |  | transmembrane |
| WP_000359282.1 | MULTISPECIES: NCS2 family permease | 3 | 2 | 49.2 | 3.77 |  | transmembrane |
| WP_001074656.1 | branched-chain amino acid transport system II carrier protein | 2 | 2 | 46.6 | 3.76 |  | transmembrane |
| WP_000140951.1 | amino acid ABC transporter ATP-binding protein | 3 | 2 | 26.9 | 3.38 |  | transmembrane |
| WP_000173361.1 | MULTISPECIES: ABC transporter ATP-binding protein | 2 | 2 | 28.1 | 2.74 |  | transmembrane |
| WP_001020311.1 | amino acid permease | 2 | 2 | 49.9 | 2.67 | *potE* | transmembrane |
| WP_001201789.1 | MULTISPECIES: phosphate ABC transporter permease PstA | 2 | 2 | 31.6 | 2.63 | *pstA* | transmembrane |
| WP_000392544.1 | ClC family H(+)/Cl(-) exchange transporter | 2 | 2 | 56.4 | 2.53 |  | transmembrane |
| WP_000026645.1 | MULTISPECIES: Na/Pi cotransporter family protein | 2 | 2 | 59.5 | 2.36 |  | transmembrane |
| WP_001070908.1 | MULTISPECIES: phosphate ABC transporter permease subunit PstC | 2 | 2 | 32.7 | 2.05 | *pstC* | transmembrane |
| WP_000679952.1 | LPXTG-anchored beta-N-acetylhexosaminidase StrH | 2 | 2 | 144.5 | 1.68 | *strH* | transmembrane |
| WP_001193686.1 | LPXTG-anchored hyaluronate lyase | 2 | 2 | 120.7 | 0 |  | transmembrane |
| WP_000156969.1 | PTS system mannose/fructose/N-acetylgalactosamine-transporter subunit IIB | 2 | 2 | 17.2 | 0 |  | transmembrane |
| WP_000428163.1 | hypothetical protein | 47 | 22 | 34.7 | 146.06 |  |  |
| WP_000038619.1 | helicase | 34 | 12 | 14.3 | 103.71 |  |  |
| WP_001051780.1 | MULTISPECIES: DUF3042 family protein | 15 | 7 | 6 | 41.69 |  |  |
| WP_000216436.1 | MULTISPECIES: Asp23/Gls24 family envelope stress response protein | 12 | 6 | 12.9 | 37.17 |  |  |
| WP_000454809.1 | MULTISPECIES: DUF969 domain-containing protein | 10 | 6 | 25 | 31.44 |  |  |
| WP_000079358.1 | MULTISPECIES: DUF956 family protein | 8 | 5 | 13.7 | 23.67 |  |  |
| WP_000065987.1 | YlbF/YmcA family competence regulator | 9 | 5 | 12.4 | 22.98 |  |  |
| WP_000777760.1 | MULTISPECIES: hypothetical protein | 7 | 5 | 10.9 | 22.94 |  |  |
| WP_000354896.1 | PFL family protein | 8 | 7 | 46.3 | 21.45 |  |  |
| WP_000198689.1 | DUF1700 domain-containing protein | 7 | 4 | 21.3 | 19.49 |  |  |
| WP_001002614.1 | DUF2130 domain-containing protein | 7 | 7 | 49.1 | 19.38 |  |  |
| WP_001130038.1 | MULTISPECIES: hypothetical protein | 7 | 4 | 8.2 | 18.92 |  |  |
| WP_000917330.1 | MULTISPECIES: co-chaperone GroES | 6 | 4 | 9.9 | 17.7 |  |  |
| WP_001134456.1 | N-acetylglucosamine-6-phosphate deacetylase | 8 | 7 | 41.7 | 17.47 |  |  |
| WP_001216918.1 | MULTISPECIES: galactose-6-phosphate isomerase subunit LacB | 5 | 3 | 18.9 | 17.09 |  |  |
| WP_000862488.1 | TRZ/ATZ family protein | 7 | 6 | 46.5 | 16.77 |  |  |
| WP_000542476.1 | aspartate-semialdehyde dehydrogenase | 6 | 6 | 39 | 16.59 |  |  |
| WP_000938258.1 | DUF1189 domain-containing protein | 6 | 5 | 30.6 | 16.39 |  |  |
| WP_000517378.1 | YtxH domain-containing protein | 5 | 5 | 14.3 | 15.94 |  |  |
| WP_001162128.1 | MULTISPECIES: flavodoxin | 4 | 3 | 16 | 14.37 |  |  |
| WP_001844147.1 | YqeG family HAD IIIA-type phosphatase | 5 | 4 | 22.6 | 14.05 |  |  |
| WP_000847054.1 | MULTISPECIES: LURP-one-related family protein | 4 | 2 | 18.8 | 12.32 |  |  |
| WP_000109957.1 | CsbD family protein | 3 | 2 | 7 | 11.07 |  |  |
| WP_000073937.1 | hypothetical protein | 6 | 4 | 15.7 | 10.89 |  |  |
| WP_001140412.1 | RidA family protein | 4 | 3 | 13.7 | 9.32 |  |  |
| WP_000810872.1 | hypothetical protein | 5 | 4 | 22 | 9.31 |  |  |
| WP_000030213.1 | alkaline shock response membrane anchor protein AmaP | 6 | 5 | 21.8 | 8.67 |  |  |
| WP_000241392.1 | DJ-1/PfpI family protein | 4 | 3 | 20.2 | 8.4 |  |  |
| WP_000507059.1 | MULTISPECIES: IreB family regulatory phosphoprotein | 3 | 2 | 10.2 | 7.74 |  |  |
| WP_001261452.1 | ClbS/DfsB family four-helix bundle protein | 5 | 4 | 20.5 | 7.4 |  |  |
| WP_001812957.1 | TIGR00341 family protein | 2 | 2 | 38.8 | 7.03 |  |  |
| WP_001140478.1 | prephenate dehydrogenase | 2 | 2 | 41 | 6.55 |  |  |
| WP_000411764.1 | acetolactate synthase large subunit | 3 | 3 | 61.7 | 6.39 |  |  |
| WP_000895040.1 | MULTISPECIES: DUF948 domain-containing protein | 2 | 2 | 13.7 | 5.51 |  |  |
| WP_000158639.1 | MULTISPECIES: thymidylate synthase | 3 | 2 | 32.5 | 5.17 |  |  |
| WP_000712374.1 | DUF3278 domain-containing protein | 3 | 3 | 20.6 | 4.81 |  |  |
| WP_001808792.1 | hypothetical protein | 2 | 2 | 21.9 | 4.41 |  |  |
| WP_000767193.1 | DUF1827 family protein | 2 | 2 | 11.2 | 4.17 |  |  |
| WP_001037922.1 | hypothetical protein | 2 | 2 | 20.8 | 4.1 |  |  |
| WP_000886114.1 | N-acetyldiaminopimelate deacetylase | 2 | 2 | 41.6 | 2.89 |  |  |
| WP_000440254.1 | MULTISPECIES: 3'-5' exonuclease | 2 | 2 | 22.1 | 2.56 |  |  |
| WP_000875454.1 | TIGR01457 family HAD-type hydrolase | 2 | 2 | 28.2 | 2.46 |  |  |
| WP_000863509.1 | MULTISPECIES: DUF1831 domain-containing protein | 2 | 2 | 13.5 | 2.26 |  |  |
| WP_000525408.1 | MULTISPECIES: DUF3397 domain-containing protein | 2 | 2 | 14 | 2.04 |  |  |
| WP_000613477.1 | MULTISPECIES: ATP-dependent Clp protease proteolytic subunit ClpP | 3 | 3 | 21.3 | 1.72 |  |  |
| WP_001216108.1 | hypothetical protein | 2 | 2 | 12.3 | 1.6 |  |  |

Accession, Identification numbers assigned to the nucleotide sequences of genes published by the DNA Data Base of Japan. #PSM, Peptide-spectrum match. #peptides, Different types of detection peptides derived from the same protein. Score, Values obtained from mass spectrometry measurement results.
