# Supplementary material for: Functional characteristics of membrane vesicles produced by Streptococcus mitis
Source: J Oral Microbiol. 2025 Sep 23;17(1):2557962. doi: 10.1080/20002297.2025.2557962 (PMC12459155; doi:10.1080/20002297.2025.2557962)
Supplement: Supplementary material — Supplementary Figure [file ZJOM_A_2557962_SM1730.pdf]

Supplementary Figure 1.

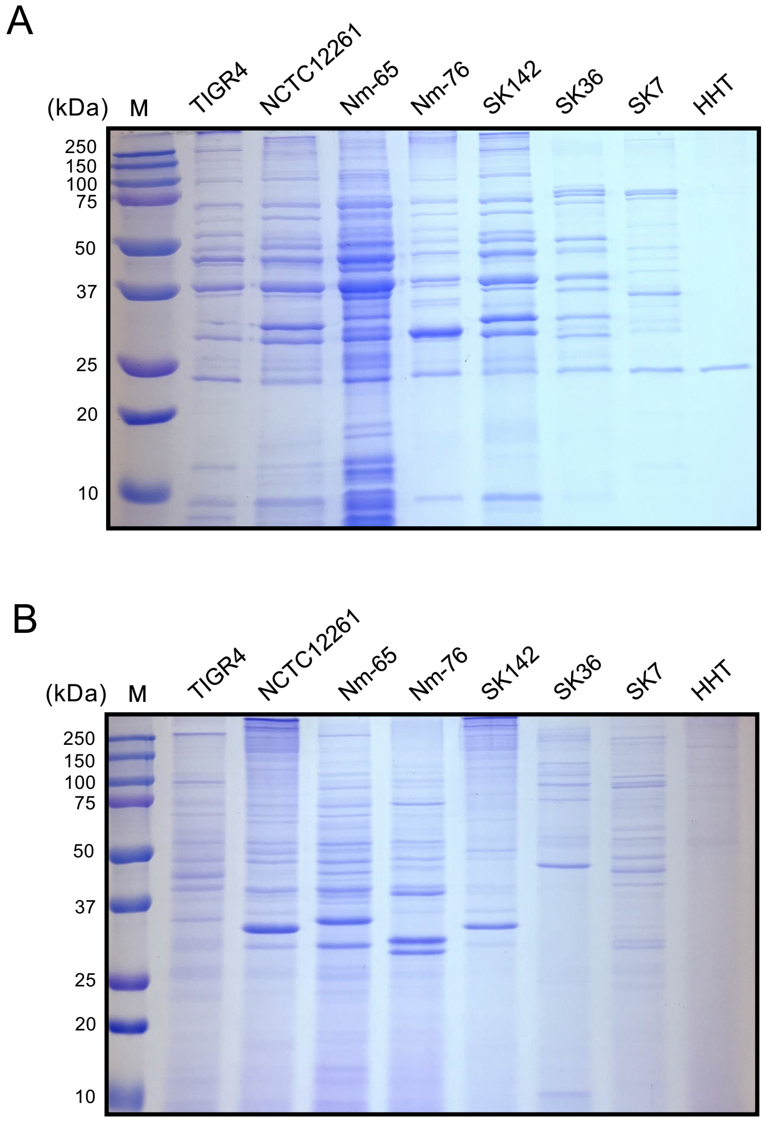

Supplementary Figure 1. Protein profiles of streptococcal cell lysates and culture supernatants.

SDS-PAGE and CBB staining of cell lysates (A) and culture supernatants (B) of *S. pneumoniae* TIGR4, *S. mitis* NCTC12261, *S. mitis* Nm-65, *S. mitis* Nm-76, *S. mitis* SK142, *S. sanguinis* SK36, *S. gordonii* SK7, and *S. salivarius* HHT. M, molecular-weight markers.
