# Supplementary material for: Functional characteristics of membrane vesicles produced by Streptococcus mitis
Source: J Oral Microbiol. 2025 Sep 23;17(1):2557962. doi: 10.1080/20002297.2025.2557962 (PMC12459155; doi:10.1080/20002297.2025.2557962)
Supplement: Supplementary material — Supplementary Table 2 [file ZJOM_A_2557962_SM1728.docx]

**Supplementary Table 2.** Mass spectrometry identification of *S. mitis* NCTC12261 proteins present in MVs and their topology prediction

| Accession | Description | # PSMs | # Peptides | MW [kDa] | Score | Gene | Localization |
| --- | --- | --- | --- | --- | --- | --- | --- |
| WP_001232997.1 | N-acetylmuramoyl-L-alanine amidase family protein | 659 | 53 | 57.1 | 2043.38 |  | cell wall |
| WP_000728241.1 | phosphorylcholine esterase CbpE | 166 | 45 | 71.7 | 539.47 | *cbpE* | cell wall |
| WP_000748703.1 | N-acetylmuramoyl-L-alanine amidase family protein | 146 | 28 | 44.3 | 453.88 |  | cell wall |
| WP_000789621.1 | MULTISPECIES: substrate-binding domain-containing protein | 96 | 28 | 34 | 353.26 |  | cell wall |
| WP_000748697.1 | N-acetylmuramoyl-L-alanine amidase family protein | 111 | 24 | 43.8 | 351.29 |  | cell wall |
| WP_000748707.1 | N-acetylmuramoyl-L-alanine amidase family protein | 105 | 19 | 44.7 | 300.62 |  | cell wall |
| WP_000835272.1 | N-acetylmuramoyl-L-alanine amidase | 67 | 32 | 54.9 | 226.24 |  | cell wall |
| WP_000792066.1 | YSIRK-type signal peptide-containing protein | 52 | 39 | 258.1 | 178.1 |  | cell wall |
| WP_161969979.1 | N-acetylmuramoyl-L-alanine amidase family protein | 53 | 26 | 41.2 | 154.46 |  | cell wall |
| WP_000469140.1 | LPXTG cell wall anchor domain-containing protein | 34 | 33 | 183.7 | 102.31 |  | cell wall |
| WP_000287308.1 | MucBP domain-containing protein | 30 | 20 | 208.5 | 87.33 |  | cell wall |
| WP_161969981.1 | LPXTG cell wall anchor domain-containing protein | 22 | 21 | 293 | 67.63 |  | cell wall |
| WP_001042490.1 | excalibur calcium-binding domain-containing protein | 19 | 7 | 21.3 | 60.97 |  | cell wall |
| WP_001035392.1 | LPXTG cell wall anchor domain-containing protein | 23 | 19 | 115.4 | 55.51 |  | cell wall |
| WP_000638494.1 | 2-C-methyl-D-erythritol 4-phosphate cytidylyltransferase | 18 | 9 | 26.2 | 46.75 | *tarI* | cell wall |
| WP_023947700.1 | cell wall hydrolase Pmp23 | 11 | 7 | 22.9 | 46.01 | *pvaA* | cell wall |
| WP_001199635.1 | phosphorylcholine transferase LicD | 10 | 7 | 32.1 | 36.41 | *licD* | cell wall |
| WP_000792778.1 | bacterial Ig-like domain-containing protein | 13 | 12 | 193.4 | 33.39 |  | cell wall |
| WP_078228276.1 | N-acetylmuramoyl-L-alanine amidase family protein | 13 | 9 | 36.6 | 31.23 |  | cell wall |
| WP_029689639.1 | LTA synthase family protein | 11 | 11 | 80.2 | 30.8 |  | cell wall |
| WP_000648064.1 | alanine racemase | 6 | 4 | 39.9 | 25.25 |  | cell wall |
| WP_000018498.1 | class A sortase | 6 | 5 | 28.2 | 16.43 |  | cell wall |
| WP_000263185.1 | type 1 glutamine amidotransferase | 4 | 3 | 29.2 | 16.27 |  | cell wall |
| WP_078228405.1 | pneumococcal-type histidine triad protein | 2 | 2 | 131.1 | 2.14 |  | cell wall |
| WP_001040721.1 | MULTISPECIES: elongation factor Tu | 315 | 35 | 43.9 | 1165.2 | *tuf* | cytosolic |
| WP_000260633.1 | formate C-acetyltransferase | 230 | 57 | 87.5 | 817.05 | *pflB* | cytosolic |
| WP_025169744.1 | bifunctional acetaldehyde-CoA/alcohol dehydrogenase | 267 | 50 | 97.5 | 729.1 |  | cytosolic |
| WP_020902894.1 | pyruvate kinase | 133 | 40 | 54.7 | 451.18 | *pyk* | cytosolic |
| WP_000334287.1 | glutamine--fructose-6-phosphate transaminase (isomerizing) | 122 | 39 | 65.4 | 443.95 | *glmS* | cytosolic |
| WP_001085675.1 | MULTISPECIES: 50S ribosomal protein L1 [Bacteria] | 114 | 11 | 24.5 | 411.53 |  | cytosolic |
| WP_000013542.1 | MULTISPECIES: 50S ribosomal protein L5 [Bacteria] | 129 | 19 | 19.8 | 409.09 |  | cytosolic |
| WP_000608360.1 | threonine--tRNA ligase | 149 | 50 | 74.5 | 405.9 | *thrS* | cytosolic |
| WP_000774068.1 | beta-ketoacyl-ACP synthase II | 117 | 27 | 43.8 | 400.32 | *fabF* | cytosolic |
| WP_000228758.1 | DNA-directed RNA polymerase subunit beta' | 115 | 65 | 137 | 345.46 |  | cytosolic |
| WP_000090348.1 | MULTISPECIES: elongation factor G | 96 | 37 | 76.8 | 331.55 | *fusA* | cytosolic |
| WP_000820863.1 | MULTISPECIES: 6-phosphofructokinase | 87 | 29 | 35.3 | 324.15 | *pfkA* | cytosolic |
| WP_078228389.1 | phosphoenolpyruvate carboxylase | 98 | 52 | 103.3 | 320.36 |  | cytosolic |
| WP_000268459.1 | MULTISPECIES: 30S ribosomal protein S2 | 92 | 21 | 28.8 | 318.38 |  | cytosolic |
| WP_000759264.1 | MULTISPECIES: redoxin family protein | 141 | 19 | 20.7 | 257.23 |  | cytosolic |
| WP_000673651.1 | NAD(P)H-dependent oxidoreductase | 63 | 23 | 45.4 | 234.8 |  | cytosolic |
| WP_000907164.1 | MULTISPECIES: DNA-directed RNA polymerase subunit beta | 72 | 42 | 133.9 | 227.63 | *ropB* | cytosolic |
| WP_025169748.1 | adenylosuccinate synthase | 74 | 30 | 47.5 | 214.7 | *purA* | cytosolic |
| WP_000240129.1 | MULTISPECIES: phosphoglycerate mutase | 91 | 22 | 26 | 213.79 | *gpmA* | cytosolic |
| WP_000523256.1 | class 1b ribonucleoside-diphosphate reductase subunit alpha | 70 | 33 | 81.5 | 203.61 |  | cytosolic |
| WP_000067880.1 | transketolase | 57 | 27 | 71.1 | 203.16 | *tkt* | cytosolic |
| WP_000260662.1 | MULTISPECIES: type I glyceraldehyde-3-phosphate dehydrogenase | 58 | 21 | 35.9 | 192.05 | *gap* | cytosolic |
| WP_000191819.1 | MULTISPECIES: pyruvate oxidase | 50 | 21 | 65.2 | 191.36 | *pox5* | cytosolic |
| WP_000743565.1 | DUF1846 domain-containing protein | 55 | 31 | 55 | 182.15 |  | cytosolic |
| WP_000092756.1 | MULTISPECIES: 30S ribosomal protein S4 [Bacteria] | 56 | 21 | 23 | 179.99 | *rpsD* | cytosolic |
| WP_000857456.1 | enoyl-[acyl-carrier-protein] reductase FabK | 43 | 16 | 34.1 | 177.11 | *fabK* | cytosolic |
| WP_000961526.1 | phenylalanine--tRNA ligase subunit beta | 42 | 26 | 87 | 173.53 | *pheT* | cytosolic |
| WP_000515974.1 | MULTISPECIES: uracil phosphoribosyltransferase | 42 | 14 | 22.9 | 168.31 | *upp* | cytosolic |
| WP_000814058.1 | proline--tRNA ligase | 53 | 31 | 68.6 | 167.18 | *proS* | cytosolic |
| WP_000102445.1 | lysine--tRNA ligase | 53 | 30 | 56.6 | 166.93 | *lysS* | cytosolic |
| WP_000039186.1 | translation initiation factor IF-2 | 48 | 29 | 102.6 | 151.31 | *infB* | cytosolic |
| WP_000512911.1 | MULTISPECIES: 50S ribosomal protein L2 | 59 | 14 | 29.9 | 149.61 |  | cytosolic |
| WP_000488686.1 | acetyl-CoA carboxylase biotin carboxylase subunit | 42 | 18 | 49.5 | 148.32 | *accC* | cytosolic |
| WP_161969984.1 | asparagine--tRNA ligase | 46 | 27 | 51.2 | 140.98 |  | cytosolic |
| WP_000164733.1 | MULTISPECIES: glycine--tRNA ligase subunit beta | 40 | 28 | 75.4 | 138.14 | *glyS* | cytosolic |
| WP_001287267.1 | 50S ribosomal protein L10 | 40 | 16 | 17.5 | 137.62 |  | cytosolic |
| WP_000158771.1 | MULTISPECIES: NADP-dependent phosphogluconate dehydrogenase | 39 | 20 | 52.5 | 131.78 | *gndA* | cytosolic |
| WP_001291392.1 | methionine--tRNA ligase | 46 | 29 | 75.7 | 131.19 | *metG* | cytosolic |
| WP_000884220.1 | serine--tRNA ligase | 41 | 18 | 47.7 | 131.14 | *serS* | cytosolic |
| WP_000036656.1 | DAK2 domain-containing protein | 39 | 20 | 59.9 | 131.1 |  | cytosolic |
| WP_000950173.1 | glycogen/starch/alpha-glucan family phosphorylase | 38 | 24 | 85.2 | 130.94 | *glgP* | cytosolic |
| WP_001200059.1 | tRNA 4-thiouridine(8) synthase ThiI | 38 | 20 | 45 | 129.75 | *thiI* | cytosolic |
| WP_001274066.1 | preprotein translocase subunit SecA | 41 | 26 | 94.7 | 128.09 | *secA* | cytosolic |
| WP_000087873.1 | MULTISPECIES: 30S ribosomal protein S7 [Bacteria] | 43 | 16 | 17.7 | 127.65 |  | cytosolic |
| WP_000169061.1 | MULTISPECIES: U32 family peptidase | 33 | 21 | 47.8 | 127.58 |  | cytosolic |
| WP_000529936.1 | MULTISPECIES: 30S ribosomal protein S3 | 39 | 15 | 24 | 127.14 |  | cytosolic |
| WP_001017402.1 | acetyl-CoA carboxylase carboxyl transferase subunit alpha | 33 | 13 | 28.2 | 126.46 |  | cytosolic |
| WP_001176199.1 | 3-oxoacyl-[acyl-carrier-protein] reductase | 31 | 12 | 25.7 | 123.04 | *fabG* | cytosolic |
| WP_000164111.1 | MULTISPECIES: translational GTPase TypA | 42 | 24 | 68.1 | 117.54 | *typA* | cytosolic |
| WP_000882498.1 | MULTISPECIES: ATP-dependent Clp protease ATP-binding subunit | 35 | 21 | 83.7 | 114.99 | *clpE* | cytosolic |
| WP_000687028.1 | glucose-1-phosphate adenylyltransferase subunit GlgD | 32 | 22 | 42.7 | 113.05 | *glgD* | cytosolic |
| WP_000874202.1 | MULTISPECIES: 30S ribosomal protein S5 [Bacteria] | 29 | 8 | 17 | 112.91 |  | cytosolic |
| WP_000866073.1 | MULTISPECIES: competence system response regulator transcription factor ComE | 33 | 20 | 29.9 | 110.25 | *aglA* | cytosolic |
| WP_000141489.1 | dTDP-glucose 4,6-dehydratase | 34 | 19 | 39.2 | 109.01 | *rfbB* | cytosolic |
| WP_000144257.1 | MULTISPECIES: cell division protein FtsZ | 32 | 20 | 44.1 | 107.9 | *ftsZ* | cytosolic |
| WP_001001630.1 | MULTISPECIES: 30S ribosomal protein S1 | 39 | 23 | 43.8 | 104.38 |  | cytosolic |
| WP_000863620.1 | signal recognition particle protein | 38 | 19 | 57.7 | 99.66 | *ffh* | cytosolic |
| WP_000649187.1 | alcohol dehydrogenase AdhP | 27 | 13 | 35.8 | 98.19 | *adhP* | cytosolic |
| WP_000775866.1 | MULTISPECIES: histidine--tRNA ligase | 33 | 20 | 48.2 | 98 | *hisS* | cytosolic |
| WP_004238844.1 | MULTISPECIES: pur operon repressor | 31 | 19 | 30.2 | 96.17 | *pruR* | cytosolic |
| WP_000359160.1 | MULTISPECIES: RNA-binding transcriptional accessory protein | 27 | 20 | 78.6 | 95.18 |  | cytosolic |
| WP_001025437.1 | MULTISPECIES: peptide chain release factor 3 | 39 | 22 | 58.4 | 92.59 | *prfC* | cytosolic |
| WP_000160193.1 | MULTISPECIES: 50S ribosomal protein L3 | 21 | 8 | 22.1 | 92.4 |  | cytosolic |
| WP_004239141.1 | phenylalanine--tRNA ligase subunit alpha | 34 | 21 | 39.2 | 91.86 | *pheS* | cytosolic |
| WP_000105246.1 | MULTISPECIES: CTP synthase | 24 | 16 | 59.2 | 90.22 |  | cytosolic |
| WP_000960946.1 | MULTISPECIES: 50S ribosomal protein L16 | 28 | 8 | 15.4 | 87.92 |  | cytosolic |
| WP_000811735.1 | alanine--tRNA ligase | 36 | 26 | 96.3 | 87.49 | *alaS* | cytosolic |
| WP_078228340.1 | helicase | 27 | 11 | 14.3 | 86.1 |  | cytosolic |
| WP_000031049.1 | MULTISPECIES: glutamate--tRNA ligase | 29 | 23 | 55.9 | 85.98 |  | cytosolic |
| WP_001196038.1 | MULTISPECIES: hypothetical protein | 23 | 11 | 12.9 | 85.5 | *nrdD* | cytosolic |
| WP_001055347.1 | MULTISPECIES: 50S ribosomal protein L23 [Bacteria] | 24 | 10 | 10.8 | 85.43 |  | cytosolic |
| WP_001049320.1 | MULTISPECIES: adenine phosphoribosyltransferase | 28 | 7 | 18.7 | 82.02 | *apt* | cytosolic |
| WP_000666474.1 | pyridoxal phosphate-dependent aminotransferase | 27 | 16 | 45.7 | 81.74 |  | cytosolic |
| WP_000096121.1 | glucose-6-phosphate dehydrogenase | 28 | 17 | 56.7 | 79.74 | *zdf* | cytosolic |
| WP_000048085.1 | UDP-N-acetylmuramate--L-alanine ligase | 21 | 15 | 49.9 | 78.6 | *murC* | cytosolic |
| WP_000546884.1 | MULTISPECIES: tyrosine--tRNA ligase | 20 | 13 | 47.4 | 78.51 | *tyrS* | cytosolic |
| WP_000768062.1 | isoleucine--tRNA ligase | 23 | 21 | 105.1 | 76.58 | *ileS* | cytosolic |
| WP_000021967.1 | MULTISPECIES: PTS sugar transporter subunit IIB | 25 | 14 | 35.5 | 75.64 | *manX* | cytosolic |
| WP_001109666.1 | ATP-dependent Clp protease ATP-binding subunit | 23 | 21 | 90.1 | 75.6 | *clpC* | cytosolic |
| WP_000366338.1 | pyridoxal phosphate-dependent aminotransferase | 25 | 17 | 43.2 | 75.08 |  | cytosolic |
| WP_000083722.1 | methylenetetrahydrofolate--tRNA-(uracil(54)-C(5))-methyltransferase (FADH(2)-oxidizing) TrmFO | 21 | 16 | 49.2 | 74.79 |  | cytosolic |
| WP_001096741.1 | phosphoglycerate kinase | 20 | 14 | 41.8 | 74.61 | *pgk* | cytosolic |
| WP_000777770.1 | pyridoxal phosphate-dependent aminotransferase | 19 | 13 | 42.8 | 74.46 |  | cytosolic |
| WP_023946545.1 | MULTISPECIES: Asp-tRNA(Asn)/Glu-tRNA(Gln) amidotransferase subunit GatA | 21 | 16 | 51.9 | 69.09 | *gatA* | cytosolic |
| WP_000804766.1 | 23S rRNA (adenine(2503)-C(2))-methyltransferase RlmN | 25 | 16 | 41.3 | 68.65 | *rlmN* | cytosolic |
| WP_001068669.1 | MULTISPECIES: 50S ribosomal protein L19 | 30 | 11 | 13.1 | 66.75 |  | cytosolic |
| WP_001156540.1 | MULTISPECIES: UDP-glucose 4-epimerase GalE | 18 | 13 | 37.4 | 63.5 | *lnpD* | cytosolic |
| WP_000204728.1 | MULTISPECIES: L-lactate dehydrogenase | 16 | 12 | 35.3 | 61.34 | *ldh* | cytosolic |
| WP_000830899.1 | aspartate--tRNA ligase | 24 | 18 | 66 | 61.33 |  | cytosolic |
| WP_001173357.1 | acetyl-CoA carboxylase, carboxyltransferase subunit beta | 21 | 14 | 31.8 | 60.99 | *accD* | cytosolic |
| WP_001285237.1 | MULTISPECIES: tRNA guanosine(34) transglycosylase Tgt | 17 | 6 | 43.1 | 60.83 | *tgt* | cytosolic |
| WP_000892173.1 | MULTISPECIES: hypoxanthine phosphoribosyltransferase | 22 | 14 | 20.1 | 60.38 | *htp* | cytosolic |
| WP_000208439.1 | MULTISPECIES: tryptophan synthase subunit beta | 17 | 12 | 42.4 | 60.22 | *trpB* | cytosolic |
| WP_000036778.1 | MULTISPECIES: FAD-dependent oxidoreductase | 17 | 14 | 50.3 | 59.58 | *nox* | cytosolic |
| WP_000201321.1 | MULTISPECIES: ribosome biogenesis GTPase YlqF | 21 | 13 | 32.1 | 59.18 | *ylqF* | cytosolic |
| WP_000609878.1 | ribitol-5-phosphate dehydrogenase | 16 | 11 | 38.7 | 58.98 | *tarJ* | cytosolic |
| WP_078228268.1 | recombinase RecA | 21 | 13 | 41.3 | 57.56 | *recA* | cytosolic |
| WP_000034661.1 | MULTISPECIES: molecular chaperone DnaK | 20 | 15 | 64.7 | 56.7 | *dnaK* | cytosolic |
| WP_000024547.1 | MULTISPECIES: 50S ribosomal protein L4 | 14 | 9 | 22.2 | 56.06 |  | cytosolic |
| WP_000245504.1 | MULTISPECIES: 30S ribosomal protein S8 | 16 | 7 | 14.8 | 55.89 |  | cytosolic |
| WP_000869665.1 | MULTISPECIES: aspartate kinase | 17 | 14 | 50.2 | 55.17 |  | cytosolic |
| WP_000151944.1 | hydroxymethylglutaryl-CoA synthase | 16 | 9 | 43.4 | 54.83 |  | cytosolic |
| WP_000073419.1 | IMP dehydrogenase | 16 | 14 | 52.5 | 54.49 | *guaB* | cytosolic |
| WP_000065716.1 | glutamine-hydrolyzing GMP synthase | 15 | 11 | 57.4 | 54.3 | *guaA* | cytosolic |
| WP_000183308.1 | MULTISPECIES: tRNA dihydrouridine synthase DusB | 15 | 10 | 35.7 | 53.46 | *dusB* | cytosolic |
| WP_000940738.1 | MULTISPECIES: GTP-sensing pleiotropic transcriptional regulator CodY | 16 | 9 | 29.7 | 52.73 | *codY* | cytosolic |
| WP_020902835.1 | MULTISPECIES: UDP-N-acetylglucosamine 1-carboxyvinyltransferase | 15 | 12 | 45 | 52.28 | *murA* | cytosolic |
| WP_000090781.1 | MULTISPECIES: 30S ribosomal protein S13 [Bacteria] | 21 | 12 | 13.4 | 51.83 |  | cytosolic |
| WP_000599102.1 | MULTISPECIES: ribosome-associated translation inhibitor RaiA | 14 | 9 | 21.1 | 51.55 | *raiA* | cytosolic |
| WP_001284513.1 | MULTISPECIES: 30S ribosomal protein S10 [Terrabacteria group] | 20 | 8 | 11.6 | 51.04 |  | cytosolic |
| WP_000086626.1 | MULTISPECIES: 50S ribosomal protein L6 | 17 | 10 | 19.4 | 47.99 |  | cytosolic |
| WP_000221903.1 | MULTISPECIES: tRNA uridine-5-carboxymethylaminomethyl(34) synthesis enzyme MnmG | 19 | 15 | 71 | 47.76 | *mnmG* | cytosolic |
| WP_000143254.1 | GTPase Era | 19 | 14 | 34 | 47.4 |  | cytosolic |
| WP_001142527.1 | tyrosine-protein kinase | 16 | 9 | 25.5 | 46.85 |  | cytosolic |
| WP_000360137.1 | 1,4-alpha-glucan branching protein GlgB | 18 | 13 | 75.4 | 46.7 | *glgB* | cytosolic |
| WP_000290683.1 | MULTISPECIES: ketol-acid reductoisomerase | 13 | 9 | 37.3 | 46.58 | *ilvC* | cytosolic |
| WP_001229119.1 | MULTISPECIES: tagatose-bisphosphate aldolase | 13 | 10 | 36.3 | 46.55 | *lacD* | cytosolic |
| WP_000429276.1 | MULTISPECIES: ribose-5-phosphate isomerase RpiA | 12 | 9 | 24.8 | 45.86 | *rpiA* | cytosolic |
| WP_000010168.1 | MULTISPECIES: ribose-phosphate diphosphokinase | 13 | 9 | 35.4 | 45.15 | *prs* | cytosolic |
| WP_000146535.1 | cell division regulator GpsB | 10 | 5 | 12.5 | 44.99 | *gpsB* | cytosolic |
| WP_001126417.1 | carbamoyl-phosphate synthase large subunit | 14 | 13 | 116.3 | 44.9 | *carB* | cytosolic |
| WP_000002996.1 | MULTISPECIES: UMP kinase | 13 | 9 | 26.4 | 44.44 | *pirH* | cytosolic |
| WP_001288288.1 | MULTISPECIES: pyruvate formate lyase-activating protein | 12 | 6 | 30.1 | 44.32 | *pflA* | cytosolic |
| WP_000568988.1 | MULTISPECIES: DNA-directed RNA polymerase subunit alpha | 15 | 10 | 34.2 | 44.25 | *rpoA* | cytosolic |
| WP_001207696.1 | MULTISPECIES: ribosome biogenesis GTPase Der | 12 | 10 | 49.1 | 43.51 | *engA* | cytosolic |
| WP_000411901.1 | UDP-N-acetylglucosamine 1-carboxyvinyltransferase | 19 | 17 | 45.9 | 43.32 | *murA* | cytosolic |
| WP_000202214.1 | MULTISPECIES: UTP--glucose-1-phosphate uridylyltransferase GalU | 13 | 9 | 33.1 | 43.22 | *galU* | cytosolic |
| WP_000033090.1 | phosphopentomutase | 14 | 9 | 44 | 42.35 | *deoB* | cytosolic |
| WP_004239081.1 | anaerobic ribonucleoside-triphosphate reductase | 13 | 12 | 83.8 | 42.11 |  | cytosolic |
| WP_001044624.1 | MULTISPECIES: 50S ribosomal protein L13 | 14 | 5 | 16.1 | 41.91 | *rplM* | cytosolic |
| WP_001019008.1 | fructose-bisphosphate aldolase | 14 | 7 | 31.4 | 41.29 |  | cytosolic |
| WP_001152871.1 | excinuclease ABC subunit UvrA | 16 | 16 | 104 | 40.59 | *uvrA* | cytosolic |
| WP_000852951.1 | ketoacyl-ACP synthase III | 11 | 10 | 34.9 | 40.37 | *fabH* | cytosolic |
| WP_000787285.1 | glucose-1-phosphate adenylyltransferase | 13 | 11 | 41.6 | 39.36 | *glgC* | cytosolic |
| WP_135781822.1 | DNA gyrase subunit A | 17 | 14 | 91.9 | 39.26 |  | cytosolic |
| WP_001035049.1 | endonuclease MutS2 | 12 | 9 | 87.5 | 39.24 |  | cytosolic |
| WP_000038733.1 | MULTISPECIES: glycine--tRNA ligase subunit alpha | 11 | 8 | 34.8 | 39.09 | *glyQ* | cytosolic |
| WP_000064112.1 | Asp23/Gls24 family envelope stress response protein | 14 | 11 | 21.7 | 38.81 |  | cytosolic |
| WP_001118385.1 | MULTISPECIES: 30S ribosomal protein S11 [Bacteria] | 13 | 5 | 13.4 | 38.44 |  | cytosolic |
| WP_000670177.1 | MULTISPECIES: nitroreductase family protein | 13 | 9 | 22.7 | 37.88 |  | cytosolic |
| WP_115262376.1 | CshA/CshB family fibrillar adhesin-related protein | 21 | 15 | 467.3 | 37.61 |  | cytosolic |
| WP_000075964.1 | MULTISPECIES: 30S ribosomal protein S9 | 13 | 4 | 14.2 | 37.35 |  | cytosolic |
| WP_001006413.1 | type I restriction-modification system subunit M | 12 | 11 | 60.3 | 37.02 |  | cytosolic |
| WP_001018251.1 | MULTISPECIES: 30S ribosomal protein S15 [Bacteria] | 9 | 4 | 10.5 | 36.99 |  | cytosolic |
| WP_004238723.1 | MULTISPECIES: DEAD/DEAH box helicase | 14 | 13 | 50.7 | 35.06 |  | cytosolic |
| WP_000179940.1 | DNA mismatch repair protein MutS | 10 | 10 | 94.7 | 34.76 | *mutS* | cytosolic |
| WP_000201903.1 | MULTISPECIES: RNA polymerase sigma factor RpoD | 10 | 8 | 42 | 34.41 | *rpoD* | cytosolic |
| WP_000167778.1 | acetate kinase | 12 | 9 | 43.3 | 34.17 | *ackA* | cytosolic |
| WP_000451571.1 | MULTISPECIES: phosphate acetyltransferase | 10 | 6 | 35.1 | 34.08 | *pta* | cytosolic |
| WP_000283125.1 | nicotinate phosphoribosyltransferase | 12 | 10 | 55.1 | 33.62 |  | cytosolic |
| WP_000391192.1 | MULTISPECIES: ribosome biogenesis GTPase YqeH | 11 | 10 | 40.9 | 33.53 | *yqeH* | cytosolic |
| WP_001269850.1 | chorismate synthase | 13 | 10 | 42.8 | 33.17 | *aroC* | cytosolic |
| WP_000699520.1 | MULTISPECIES: phosphopantothenate--cysteine ligase | 9 | 7 | 25.7 | 32.71 | *coaB* | cytosolic |
| WP_000711362.1 | class I SAM-dependent RNA methyltransferase | 9 | 7 | 43.2 | 32.52 |  | cytosolic |
| WP_000958913.1 | FAD-containing oxidoreductase | 7 | 6 | 47.2 | 32.2 |  | cytosolic |
| WP_000766087.1 | MULTISPECIES: 50S ribosomal protein L15 [Lactobacillales] | 9 | 6 | 15.4 | 31.63 |  | cytosolic |
| WP_000331493.1 | MULTISPECIES: 50S ribosomal protein L17 [Bacteria] | 11 | 6 | 14.5 | 31.58 |  | cytosolic |
| WP_000440801.1 | MULTISPECIES: 30S ribosomal protein S17 [Bacteria] | 18 | 5 | 10 | 31.24 |  | cytosolic |
| WP_000649460.1 | zinc-dependent alcohol dehydrogenase family protein | 8 | 5 | 38.1 | 31.01 |  | cytosolic |
| WP_001066310.1 | MULTISPECIES: molecular chaperone DnaJ | 11 | 8 | 40.3 | 30.95 | *dnaJ* | cytosolic |
| WP_001142332.1 | MULTISPECIES: 30S ribosomal protein S12 [Bacteria] | 12 | 6 | 15.1 | 30.05 |  | cytosolic |
| WP_004241506.1 | MULTISPECIES: phosphoenolpyruvate--protein phosphotransferase | 8 | 7 | 63.2 | 29.88 | *ptsP* | cytosolic |
| WP_000165449.1 | tryptophan--tRNA ligase | 10 | 7 | 38.4 | 29.86 |  | cytosolic |
| WP_000660620.1 | chromosomal replication initiator protein DnaA | 11 | 9 | 51.8 | 29.09 | *dnaA* | cytosolic |
| WP_000818137.1 | MULTISPECIES: 50S ribosomal protein L22 | 8 | 6 | 12.2 | 28.98 |  | cytosolic |
| WP_000717442.1 | phosphate acyltransferase PlsX | 10 | 8 | 35 | 28.97 | *plsX* | cytosolic |
| WP_000946458.1 | MULTISPECIES: phosphate signaling complex protein PhoU | 9 | 7 | 25 | 28.5 | *phoU* | cytosolic |
| WP_000923603.1 | DNA repair protein RecN | 8 | 8 | 62.8 | 28.36 | *rexN* | cytosolic |
| WP_001008638.1 | MULTISPECIES: Asp-tRNA(Asn)/Glu-tRNA(Gln) amidotransferase subunit GatB | 12 | 10 | 53.7 | 27.94 | *gatB* | cytosolic |
| WP_000451386.1 | MULTISPECIES: class 1b ribonucleoside-diphosphate reductase subunit beta | 9 | 7 | 36.9 | 27.89 | *gmk* | cytosolic |
| WP_000775045.1 | guanylate kinase | 9 | 8 | 23.8 | 27.89 | *nrdF* | cytosolic |
| WP_000844580.1 | MULTISPECIES: aldo/keto reductase | 10 | 9 | 31.6 | 27.71 |  | cytosolic |
| WP_049516646.1 | MULTISPECIES: DeoR/GlpR family DNA-binding transcription regulator | 9 | 8 | 28.7 | 27.55 |  | cytosolic |
| WP_161969983.1 | MULTISPECIES: dihydrolipoyl dehydrogenase | 11 | 9 | 59.6 | 27.54 |  | cytosolic |
| WP_000163673.1 | methionyl-tRNA formyltransferase | 7 | 6 | 33.9 | 27.39 | *fmt* | cytosolic |
| WP_000003942.1 | MULTISPECIES: methionine adenosyltransferase | 10 | 8 | 43.1 | 27.25 | *metK* | cytosolic |
| WP_000079148.1 | MULTISPECIES: universal stress protein [Bacteria] | 8 | 5 | 16.6 | 27.2 |  | cytosolic |
| WP_001283818.1 | MULTISPECIES: ribose-phosphate diphosphokinase | 6 | 6 | 35.1 | 27.13 |  | cytosolic |
| WP_001007193.1 | MULTISPECIES: GntR family transcriptional regulator | 9 | 7 | 27.7 | 27.02 | *gntR* | cytosolic |
| WP_001028815.1 | MULTISPECIES: peptide chain release factor 1 | 13 | 10 | 40.6 | 26.99 |  | cytosolic |
| WP_001090627.1 | MULTISPECIES: catabolite control protein A | 9 | 5 | 37.1 | 26.9 | *ccpA* | cytosolic |
| WP_000915955.1 | NAD(P)H-dependent oxidoreductase | 12 | 8 | 22.6 | 26.89 |  | cytosolic |
| WP_000109141.1 | MULTISPECIES: 50S ribosomal protein L21 [Bacteria] | 10 | 2 | 11.2 | 26.84 |  | cytosolic |
| WP_000616545.1 | MULTISPECIES: 50S ribosomal protein L14 [Bacteria] | 12 | 6 | 13 | 26.57 |  | cytosolic |
| WP_001293905.1 | tRNA 2-thiouridine(34) synthase MnmA | 8 | 6 | 41.5 | 26.43 | *mnmA* | cytosolic |
| WP_001216910.1 | MULTISPECIES: galactose-6-phosphate isomerase subunit LacB | 8 | 4 | 18.9 | 26.39 | *lacB* | cytosolic |
| WP_000770028.1 | Mur ligase family protein | 8 | 6 | 49.6 | 26.27 | *murT* | cytosolic |
| WP_000127463.1 | MULTISPECIES: 2,3,4,5-tetrahydropyridine-2,6-dicarboxylate N-acetyltransferase | 6 | 4 | 23.9 | 26.18 | *dapD* | cytosolic |
| WP_000124834.1 | MULTISPECIES: 50S ribosomal protein L20 [Terrabacteria group] | 8 | 5 | 13.7 | 26.1 |  | cytosolic |
| WP_000671119.1 | DEAD/DEAH box helicase | 11 | 10 | 58.5 | 26.01 |  | cytosolic |
| WP_001003032.1 | ribosome silencing factor | 5 | 4 | 13 | 25.82 |  | cytosolic |
| WP_001203672.1 | MULTISPECIES: transcriptional regulator NrdR | 9 | 7 | 18.4 | 25.12 | *nrdR* | cytosolic |
| WP_000401876.1 | excinuclease ABC subunit UvrA | 6 | 6 | 83.8 | 24.64 | *uvrA* | cytosolic |
| WP_000121686.1 | MULTISPECIES: phospho-sugar mutase | 8 | 8 | 62.6 | 24.43 | *pgm* | cytosolic |
| WP_000963701.1 | MULTISPECIES: nicotinate-nucleotide adenylyltransferase | 7 | 5 | 24.2 | 24.26 | *nadD* | cytosolic |
| WP_000865703.1 | MULTISPECIES: tRNA (adenosine(37)-N6)-threonylcarbamoyltransferase complex dimerization subunit type 1 TsaB | 8 | 6 | 25.3 | 24.25 | *tsaB* | cytosolic |
| WP_000692425.1 | homoserine kinase | 7 | 7 | 31.4 | 23.86 | *thrB* | cytosolic |
| WP_000061642.1 | MULTISPECIES: GTPase ObgE | 7 | 7 | 48.3 | 23.44 | *obgE* | cytosolic |
| WP_000855747.1 | MULTISPECIES: 23S rRNA (guanosine(2251)-2'-O)-methyltransferase RlmB | 6 | 4 | 26.4 | 23.26 | *rlmB* | cytosolic |
| WP_000920651.1 | MULTISPECIES: DeoR/GlpR family DNA-binding transcription regulator | 9 | 7 | 27.2 | 22.46 | *fruR* | cytosolic |
| WP_000232190.1 | MULTISPECIES: 3-deoxy-7-phosphoheptulonate synthase | 9 | 7 | 38.9 | 22.32 |  | cytosolic |
| WP_078228336.1 | valine--tRNA ligase | 12 | 8 | 100.8 | 22.15 |  | cytosolic |
| WP_001181377.1 | MULTISPECIES: uridine kinase | 7 | 6 | 24.5 | 22.03 | *udk* | cytosolic |
| WP_000113945.1 | DEAD/DEAH box helicase family protein | 8 | 8 | 119.2 | 21.9 |  | cytosolic |
| WP_000037260.1 | DNA topoisomerase IV subunit B | 6 | 5 | 71.6 | 21.77 | *parE* | cytosolic |
| WP_000653417.1 | MULTISPECIES: redox-sensing transcriptional repressor Rex | 9 | 7 | 24.1 | 21.67 | *rex* | cytosolic |
| WP_000354330.1 | MULTISPECIES: ribonuclease Z | 8 | 6 | 34.1 | 21.62 | *rnz* | cytosolic |
| WP_000415098.1 | NAD(P)H-dependent glycerol-3-phosphate dehydrogenase | 6 | 5 | 36.8 | 21.58 | *gpsA* | cytosolic |
| WP_000640798.1 | MULTISPECIES: 1-phosphofructokinase | 7 | 5 | 32.6 | 21 | *pfkB* | cytosolic |
| WP_004238789.1 | exodeoxyribonuclease III | 7 | 6 | 31.3 | 20.83 | *eth* | cytosolic |
| WP_000018200.1 | DNA mismatch repair endonuclease MutL | 6 | 6 | 73.4 | 19.81 | *mutL* | cytosolic |
| WP_001103468.1 | MULTISPECIES: F0F1 ATP synthase subunit B | 6 | 5 | 17.8 | 19.79 |  | cytosolic |
| WP_001284640.1 | MULTISPECIES: HU family DNA-binding protein | 6 | 3 | 9.6 | 19.5 |  | cytosolic |
| WP_000040929.1 | Xaa-Pro peptidase family protein | 5 | 5 | 40.3 | 19.33 |  | cytosolic |
| WP_000522340.1 | signal recognition particle-docking protein FtsY | 6 | 5 | 47.8 | 19.31 | *ftsY* | cytosolic |
| WP_000795106.1 | acetylxylan esterase | 6 | 5 | 37.4 | 19.12 |  | cytosolic |
| WP_000255154.1 | dihydroorotate oxidase | 8 | 7 | 34.4 | 19.04 |  | cytosolic |
| WP_000497691.1 | MULTISPECIES: 50S ribosomal protein L24 [Bacteria] | 6 | 5 | 11 | 18.86 |  | cytosolic |
| WP_012972566.1 | MULTISPECIES: ATP-dependent Clp protease ATP-binding subunit ClpX | 10 | 10 | 45.7 | 18.85 | *clpX* | cytosolic |
| WP_000033398.1 | dTMP kinase | 6 | 5 | 23.5 | 18.8 |  | cytosolic |
| WP_000798432.1 | choline binding-anchored murein hydrolase CbpD | 8 | 8 | 41 | 18.32 | *cbpD* | cytosolic |
| WP_000191949.1 | galactokinase | 5 | 5 | 43.7 | 18.02 |  | cytosolic |
| WP_001279117.1 | ABC-F family ATP-binding cassette domain-containing protein | 5 | 5 | 70.8 | 18 |  | cytosolic |
| WP_000150262.1 | ROK family protein | 8 | 4 | 31.3 | 17.87 |  | cytosolic |
| WP_000222640.1 | response regulator transcription factor | 5 | 4 | 28.2 | 17.63 |  | cytosolic |
| WP_000036046.1 | MULTISPECIES: manganese-dependent inorganic pyrophosphatase | 6 | 5 | 33.4 | 17.62 |  | cytosolic |
| WP_000200820.1 | HAD family hydrolase | 6 | 4 | 23.4 | 17.46 |  | cytosolic |
| WP_000808076.1 | MULTISPECIES: translation elongation factor Ts | 5 | 3 | 37.4 | 17.44 |  | cytosolic |
| WP_000395967.1 | type 1 glycerol-3-phosphate oxidase | 8 | 7 | 66.7 | 17.35 |  | cytosolic |
| WP_000137346.1 | MULTISPECIES: dihydroxy-acid dehydratase | 4 | 3 | 59.8 | 17.3 |  | cytosolic |
| WP_000199366.1 | NADP-dependent glyceraldehyde-3-phosphate dehydrogenase | 6 | 5 | 50.8 | 17.08 |  | cytosolic |
| WP_000642690.1 | competence/damage-inducible protein A | 5 | 4 | 45.1 | 16.79 |  | cytosolic |
| WP_000196453.1 | 7-carboxy-7-deazaguanine synthase QueE | 5 | 4 | 26.7 | 16.56 | *queE* | cytosolic |
| WP_000931177.1 | MULTISPECIES: GMP reductase | 4 | 4 | 35.9 | 16.54 |  | cytosolic |
| WP_000031982.1 | cystathionine gamma-synthase | 6 | 6 | 40 | 16.49 |  | cytosolic |
| WP_000120723.1 | MULTISPECIES: L-lactate oxidase | 7 | 7 | 41.5 | 16.34 |  | cytosolic |
| WP_000533766.1 | MULTISPECIES: 30S ribosomal protein S19 [Bacteria] | 6 | 4 | 10.7 | 15.89 |  | cytosolic |
| WP_000119149.1 | MULTISPECIES: GntR family transcriptional regulator | 5 | 4 | 13.9 | 15.81 |  | cytosolic |
| WP_000283807.1 | DNA polymerase III subunit gamma/tau | 5 | 5 | 60.8 | 15.81 |  | cytosolic |
| WP_070527626.1 | MULTISPECIES: ferrochelatase | 5 | 5 | 42.5 | 15.76 |  | cytosolic |
| WP_000158610.1 | thymidylate synthase | 5 | 4 | 32.6 | 15.68 |  | cytosolic |
| WP_004238721.1 | FAD:protein FMN transferase | 6 | 6 | 33.8 | 15.68 |  | cytosolic |
| WP_000904719.1 | alanine dehydrogenase | 4 | 3 | 38.9 | 15.62 |  | cytosolic |
| WP_004239203.1 | 16S rRNA (cytosine(967)-C(5))-methyltransferase RsmB | 6 | 6 | 48.9 | 15.62 | *rsmB* | cytosolic |
| WP_001178420.1 | site-specific tyrosine recombinase XerD | 5 | 5 | 28.7 | 15.55 | *xerD* | cytosolic |
| WP_000769495.1 | SP_0198 family lipoprotein | 5 | 3 | 16.6 | 15.46 |  | cytosolic |
| WP_000131475.1 | dTDP-4-dehydrorhamnose 3,5-epimerase family protein | 4 | 4 | 22.3 | 15.41 |  | cytosolic |
| WP_000244445.1 | endonuclease III | 4 | 3 | 23.3 | 15.39 |  | cytosolic |
| WP_001145422.1 | LacI family DNA-binding transcriptional regulator | 6 | 6 | 36.8 | 15.35 |  | cytosolic |
| WP_000567554.1 | MULTISPECIES: epoxyqueuosine reductase QueH | 5 | 4 | 29.7 | 15.28 | *queH* | cytosolic |
| WP_000686917.1 | MULTISPECIES: tRNA (guanosine(37)-N1)-methyltransferase TrmD | 6 | 5 | 27.6 | 15.24 | *trmD* | cytosolic |
| WP_000844496.1 | bifunctional biotin--[acetyl-CoA-carboxylase] ligase/biotin operon repressor BirA | 5 | 3 | 35.2 | 15.14 | *birA* | cytosolic |
| WP_000568643.1 | MULTISPECIES: elongation factor P | 4 | 3 | 20.6 | 14.89 |  | cytosolic |
| WP_078228397.1 | tRNA uridine-5-carboxymethylaminomethyl(34) synthesis GTPase MnmE | 7 | 6 | 50.4 | 14.84 | *mnmE* | cytosolic |
| WP_000280915.1 | chromosome segregation protein SMC | 5 | 5 | 133.9 | 14.72 |  | cytosolic |
| WP_000952970.1 | MULTISPECIES: threonine ammonia-lyase IlvA | 5 | 5 | 45.4 | 14.6 | *llvA* | cytosolic |
| WP_000864047.1 | 3-deoxy-7-phosphoheptulonate synthase | 4 | 3 | 38.6 | 14.53 |  | cytosolic |
| WP_000076798.1 | glycerol kinase GlpK | 4 | 4 | 55.8 | 14.46 | *glpK* | cytosolic |
| WP_000661488.1 | ribonuclease III | 5 | 4 | 26.2 | 14.11 |  | cytosolic |
| WP_000331986.1 | MULTISPECIES: ribonuclease J | 6 | 6 | 61 | 14.07 |  | cytosolic |
| WP_001216850.1 | 16S rRNA (adenine(1518)-N(6)/adenine(1519)-N(6))-dimethyltransferase RsmA | 5 | 5 | 32.2 | 13.99 | *rsmA* | cytosolic |
| WP_000068664.1 | MULTISPECIES: 30S ribosomal protein S18 [Bacteria] | 4 | 3 | 9.2 | 13.69 |  | cytosolic |
| WP_078228339.1 | GNAT family N-acetyltransferase | 4 | 3 | 21.3 | 13.55 |  | cytosolic |
| WP_000136345.1 | heavy metal translocating P-type ATPase | 4 | 4 | 80.3 | 13.55 |  | cytosolic |
| WP_000517384.1 | YtxH domain-containing protein | 4 | 3 | 14.3 | 13.54 |  | cytosolic |
| WP_000087891.1 | MULTISPECIES: triose-phosphate isomerase [Bacteria] | 4 | 3 | 26.5 | 13.28 |  | cytosolic |
| WP_000878660.1 | sugar transferase | 4 | 4 | 40.6 | 13.18 |  | cytosolic |
| WP_000170898.1 | orotate phosphoribosyltransferase | 4 | 4 | 22.8 | 12.99 |  | cytosolic |
| WP_004239342.1 | DNA topoisomerase IV subunit A | 4 | 4 | 93 | 12.94 |  | cytosolic |
| WP_000811882.1 | phosphorylcholine transferase LicD | 4 | 3 | 31.9 | 12.76 | *licD* | cytosolic |
| WP_001232426.1 | glycosyltransferase family 1 protein | 6 | 6 | 44.6 | 12.65 |  | cytosolic |
| WP_000057241.1 | MULTISPECIES: 50S ribosomal protein L30 [Bacteria] | 4 | 3 | 6.4 | 12.52 |  | cytosolic |
| WP_078228400.1 | aspartate-semialdehyde dehydrogenase | 4 | 4 | 38.9 | 12.49 |  | cytosolic |
| WP_001281493.1 | MULTISPECIES: dihydrodipicolinate synthase family protein | 4 | 3 | 33.3 | 12.42 |  | cytosolic |
| WP_004255257.1 | MULTISPECIES: serine hydrolase | 4 | 4 | 48.1 | 12.34 |  | cytosolic |
| WP_001170063.1 | radical SAM family heme chaperone HemW | 4 | 4 | 43 | 12.28 | *hemW* | cytosolic |
| WP_078228416.1 | bifunctional oligoribonuclease/PAP phosphatase NrnA | 3 | 3 | 34.9 | 12.27 | *nrnA* | cytosolic |
| WP_000912013.1 | MULTISPECIES: GFA family protein | 4 | 3 | 13.9 | 12.17 |  | cytosolic |
| WP_001140859.1 | prephenate dehydrogenase | 3 | 3 | 41 | 12.08 |  | cytosolic |
| WP_000852498.1 | replicative DNA helicase | 3 | 3 | 50.2 | 12.03 |  | cytosolic |
| WP_000199543.1 | MULTISPECIES: NADP-specific glutamate dehydrogenase | 4 | 3 | 48.8 | 11.87 |  | cytosolic |
| WP_000762510.1 | shikimate dehydrogenase | 4 | 4 | 31.1 | 11.85 |  | cytosolic |
| WP_001114620.1 | DNA-formamidopyrimidine glycosylase | 4 | 3 | 31.1 | 11.81 |  | cytosolic |
| WP_001151778.1 | MULTISPECIES: 30S ribosomal protein S6 | 4 | 3 | 11.1 | 11.74 |  | cytosolic |
| WP_000116485.1 | MULTISPECIES: trigger factor | 5 | 5 | 47.3 | 11.57 |  | cytosolic |
| WP_000411223.1 | choline kinase LicA | 3 | 2 | 33.5 | 11.52 | *licA* | cytosolic |
| WP_000159416.1 | MULTISPECIES: 16S rRNA (cytosine(1402)-N(4))-methyltransferase RsmH | 4 | 3 | 36 | 11.48 | *rsmH* | cytosolic |
| WP_000743336.1 | gamma-glutamyl-gamma-aminobutyrate hydrolase family protein | 5 | 4 | 25.8 | 11.4 |  | cytosolic |
| WP_000018268.1 | MULTISPECIES: glucose-6-phosphate isomerase | 4 | 4 | 49.9 | 11.38 |  | cytosolic |
| WP_000801936.1 | 16S rRNA (guanine(527)-N(7))-methyltransferase RsmG | 4 | 4 | 27 | 11.37 | *rsmG* | cytosolic |
| WP_000560719.1 | CBS domain-containing protein | 3 | 2 | 17.3 | 11.35 |  | cytosolic |
| WP_000268760.1 | MULTISPECIES: 30S ribosomal protein S16 [Bacteria] | 4 | 4 | 10.2 | 11.35 |  | cytosolic |
| WP_000227853.1 | DNA primase | 3 | 3 | 67.2 | 11.32 |  | cytosolic |
| WP_001180405.1 | ABC transporter permease | 4 | 3 | 44.8 | 11.18 |  | cytosolic |
| WP_000624044.1 | MULTISPECIES: 50S ribosomal protein L18 [Terrabacteria group] | 3 | 3 | 12.9 | 11.16 |  | cytosolic |
| WP_001166865.1 | 16S rRNA (cytidine(1402)-2'-O)-methyltransferase | 4 | 2 | 32.8 | 11.14 |  | cytosolic |
| WP_001266106.1 | MULTISPECIES: tRNA (guanosine(46)-N7)-methyltransferase TrmB | 3 | 2 | 24.3 | 11.1 | *trmB* | cytosolic |
| WP_001211996.1 | Xaa-Pro dipeptidyl-peptidase | 4 | 4 | 87.4 | 11.09 |  | cytosolic |
| WP_000240964.1 | dihydropteroate synthase | 4 | 3 | 35.4 | 11.07 |  | cytosolic |
| WP_000060171.1 | MULTISPECIES: ribosome assembly RNA-binding protein YhbY | 4 | 3 | 11.7 | 11.03 | *yhbY* | cytosolic |
| WP_000345104.1 | class I SAM-dependent methyltransferase | 3 | 3 | 35.6 | 11 |  | cytosolic |
| WP_000922210.1 | MULTISPECIES: sugar transferase | 4 | 4 | 26.6 | 10.97 |  | cytosolic |
| WP_000027873.1 | MULTISPECIES: 4-hydroxy-tetrahydrodipicolinate reductase | 4 | 4 | 27.8 | 10.86 |  | cytosolic |
| WP_000134056.1 | MULTISPECIES: DNA topoisomerase (ATP-hydrolyzing) subunit B | 5 | 4 | 72.1 | 10.82 |  | cytosolic |
| WP_000230210.1 | MULTISPECIES: LacI family DNA-binding transcriptional regulator | 3 | 3 | 38.1 | 10.78 |  | cytosolic |
| WP_000974733.1 | MULTISPECIES: superoxide dismutase SodA | 3 | 3 | 22.4 | 10.61 | *sodA* | cytosolic |
| WP_000078104.1 | uracil-DNA glycosylase family protein | 4 | 4 | 22.1 | 10.6 |  | cytosolic |
| WP_000273858.1 | MULTISPECIES: PadR family transcriptional regulator | 4 | 2 | 12.4 | 10.48 |  | cytosolic |
| WP_000573124.1 | MULTISPECIES: SAP domain-containing protein | 6 | 5 | 24.5 | 10.42 |  | cytosolic |
| WP_000032285.1 | MULTISPECIES: transcription termination factor NusA | 3 | 3 | 42.7 | 10.33 | *nusA* | cytosolic |
| WP_000767784.1 | type I 3-dehydroquinate dehydratase | 3 | 3 | 25.7 | 10.25 |  | cytosolic |
| WP_078228466.1 | NAD(P)/FAD-dependent oxidoreductase | 5 | 3 | 42.2 | 10.22 |  | cytosolic |
| WP_020903001.1 | primosomal protein N' | 3 | 3 | 90.1 | 10.18 |  | cytosolic |
| WP_000224425.1 | 2,3-butanediol dehydrogenase | 3 | 2 | 37.5 | 10.15 |  | cytosolic |
| WP_001100267.1 | MULTISPECIES: dihydroxyacetone kinase subunit L | 3 | 3 | 20.5 | 10.14 |  | cytosolic |
| WP_000406256.1 | Stp1/IreP family PP2C-type Ser/Thr phosphatase | 3 | 3 | 26.8 | 10.04 |  | cytosolic |
| WP_001196965.1 | MULTISPECIES: 50S ribosomal protein L7/L12 | 3 | 2 | 12.4 | 9.86 |  | cytosolic |
| WP_001202960.1 | pyrimidine-nucleoside phosphorylase | 3 | 3 | 46 | 9.85 |  | cytosolic |
| WP_050278219.1 | MULTISPECIES: carbamoyl phosphate synthase small subunit | 4 | 3 | 39.8 | 9.84 |  | cytosolic |
| WP_000916509.1 | MULTISPECIES: 50S ribosomal protein L27 [Bacteria] | 3 | 2 | 10.5 | 9.71 |  | cytosolic |
| WP_000908846.1 | DNA translocase FtsK | 4 | 4 | 81.5 | 9.69 | *ftsK* | cytosolic |
| WP_000644747.1 | MULTISPECIES: bifunctional pyr operon transcriptional regulator/uracil phosphoribosyltransferase PyrR | 3 | 2 | 19.5 | 9.46 | *pyrR* | cytosolic |
| WP_001227984.1 | helix-turn-helix domain-containing protein | 2 | 2 | 30.2 | 9.41 |  | cytosolic |
| WP_000791756.1 | DEAD/DEAH box helicase | 3 | 3 | 41.4 | 9.4 |  | cytosolic |
| WP_000362999.1 | MULTISPECIES: phosphotransferase family protein | 4 | 4 | 31.1 | 9.26 |  | cytosolic |
| WP_000108228.1 | MULTISPECIES: 5-methyltetrahydropteroyltriglutamate--homocysteine S-methyltransferase | 3 | 2 | 84.6 | 9.24 |  | cytosolic |
| WP_000251212.1 | alpha-L-fucosidase | 3 | 2 | 62.9 | 9.19 |  | cytosolic |
| WP_000167635.1 | ACP S-malonyltransferase | 3 | 3 | 32.9 | 9.14 |  | cytosolic |
| WP_004238699.1 | 3-phosphoshikimate 1-carboxyvinyltransferase | 2 | 2 | 45.7 | 9.08 |  | cytosolic |
| WP_000125031.1 | dipeptidase PepV | 3 | 3 | 50.8 | 9.02 | *pepV* | cytosolic |
| WP_001050081.1 | MULTISPECIES: DUF1149 family protein | 3 | 2 | 14.4 | 8.96 |  | cytosolic |
| WP_001085697.1 | MULTISPECIES: type Z 30S ribosomal protein S14 [Bacteria] | 4 | 3 | 7.1 | 8.96 |  | cytosolic |
| WP_000720683.1 | dihydroxyacetone kinase subunit DhaK | 6 | 5 | 35.2 | 8.82 | *dhaK* | cytosolic |
| WP_078228463.1 | UDP-N-acetylmuramoyl-L-alanine--D-glutamate ligase | 5 | 5 | 48.5 | 8.75 |  | cytosolic |
| WP_000466726.1 | MULTISPECIES: isoprenyl transferase | 4 | 4 | 28.7 | 8.69 |  | cytosolic |
| WP_000207599.1 | MULTISPECIES: LacI family transcriptional regulator | 4 | 3 | 36.5 | 8.56 |  | cytosolic |
| WP_000448715.1 | MULTISPECIES: alpha-ketoacid dehydrogenase subunit beta | 3 | 2 | 35.8 | 8.54 |  | cytosolic |
| WP_000166429.1 | MULTISPECIES: polyribonucleotide nucleotidyltransferase [Bacilli] | 3 | 2 | 80.9 | 8.53 |  | cytosolic |
| WP_000799056.1 | MULTISPECIES: NAD kinase | 2 | 2 | 31 | 8.47 |  | cytosolic |
| WP_001079971.1 | class I SAM-dependent rRNA methyltransferase | 7 | 7 | 43.8 | 8.42 |  | cytosolic |
| WP_000500041.1 | MULTISPECIES: L-serine ammonia-lyase, iron-sulfur-dependent, subunit alpha | 4 | 3 | 30.3 | 8.39 |  | cytosolic |
| WP_000567247.1 | adenylosuccinate lyase | 3 | 2 | 49.4 | 8.32 |  | cytosolic |
| WP_000153517.1 | Cof-type HAD-IIB family hydrolase | 3 | 3 | 30.7 | 8.28 |  | cytosolic |
| WP_001003466.1 | HD domain-containing protein | 3 | 3 | 50.8 | 8.22 |  | cytosolic |
| WP_001107051.1 | dTDP-glucose 4,6-dehydratase | 4 | 4 | 37.8 | 8.21 |  | cytosolic |
| WP_001174044.1 | aminoacyltransferase | 4 | 3 | 47.1 | 8.16 |  | cytosolic |
| WP_000502082.1 | MULTISPECIES: Holliday junction branch migration DNA helicase RuvB | 3 | 3 | 36.1 | 8.13 | *ruvB* | cytosolic |
| WP_000613638.1 | GNAT family N-acetyltransferase | 2 | 2 | 33.7 | 8.11 |  | cytosolic |
| WP_000071371.1 | PolC-type DNA polymerase III | 3 | 3 | 164.6 | 8.05 |  | cytosolic |
| WP_000065614.1 | MULTISPECIES: ribonuclease J | 2 | 2 | 60.9 | 7.97 |  | cytosolic |
| WP_025169734.1 | MULTISPECIES: TIGR01212 family radical SAM protein | 5 | 4 | 37 | 7.85 |  | cytosolic |
| WP_000136976.1 | MULTISPECIES: cytidine/deoxycytidylate deaminase family protein | 2 | 2 | 17.4 | 7.83 |  | cytosolic |
| WP_000613475.1 | MULTISPECIES: ATP-dependent Clp protease proteolytic subunit ClpP | 2 | 2 | 21.3 | 7.78 | *clpP* | cytosolic |
| WP_000029880.1 | MULTISPECIES: cysteine synthase A | 3 | 2 | 32 | 7.64 |  | cytosolic |
| WP_001030024.1 | MULTISPECIES: rhodanese-related sulfurtransferase | 2 | 2 | 37.8 | 7.56 |  | cytosolic |
| WP_000423408.1 | GNAT family N-acetyltransferase | 2 | 2 | 19.1 | 7.47 |  | cytosolic |
| WP_078228442.1 | DNA repair protein RadA | 3 | 3 | 49.4 | 7.35 |  | cytosolic |
| WP_000373450.1 | diphosphomevalonate decarboxylase | 2 | 2 | 35.4 | 7.32 |  | cytosolic |
| WP_001265622.1 | MULTISPECIES: 50S ribosomal protein L33 [Bacteria] | 4 | 3 | 5.9 | 7.26 |  | cytosolic |
| WP_000165331.1 | SDR family NAD(P)-dependent oxidoreductase | 2 | 2 | 27.4 | 7.24 |  | cytosolic |
| WP_000210642.1 | type 2 isopentenyl-diphosphate Delta-isomerase | 3 | 3 | 37.8 | 7.21 |  | cytosolic |
| WP_000025262.1 | MULTISPECIES: thiamine pyrophosphate-dependent dehydrogenase E1 component subunit alpha | 4 | 3 | 35.1 | 7.17 |  | cytosolic |
| WP_001291454.1 | MULTISPECIES: glycosyltransferase | 2 | 2 | 31.7 | 7.05 |  | cytosolic |
| WP_000201106.1 | ribonuclease HII | 2 | 2 | 28.3 | 7 |  | cytosolic |
| WP_000864218.1 | 50S ribosomal protein L9 | 4 | 2 | 16.5 | 6.97 |  | cytosolic |
| WP_000658435.1 | bifunctional folylpolyglutamate synthase/dihydrofolate synthase | 2 | 2 | 48.8 | 6.96 |  | cytosolic |
| WP_000152689.1 | RNA methyltransferase | 2 | 2 | 26.8 | 6.75 |  | cytosolic |
| WP_000422605.1 | MULTISPECIES: ribosome biogenesis GTP-binding protein YihA/YsxC | 3 | 3 | 22.3 | 6.74 |  | cytosolic |
| WP_000405856.1 | GNAT family N-acetyltransferase | 2 | 2 | 18.3 | 6.73 |  | cytosolic |
| WP_000917337.1 | MULTISPECIES: co-chaperone GroES | 2 | 2 | 9.9 | 6.66 | *groES* | cytosolic |
| WP_000676151.1 | MULTISPECIES: glucose-1-phosphate thymidylyltransferase RfbA | 2 | 2 | 32.3 | 6.61 | *rfbA* | cytosolic |
| WP_000216437.1 | MULTISPECIES: Asp23/Gls24 family envelope stress response protein | 2 | 2 | 12.9 | 6.55 |  | cytosolic |
| WP_000773723.1 | deoxyribose-phosphate aldolase | 2 | 2 | 23 | 6.42 |  | cytosolic |
| WP_000775316.1 | MULTISPECIES: DUF402 domain-containing protein | 3 | 3 | 21.3 | 6.38 |  | cytosolic |
| WP_000234217.1 | MULTISPECIES: YqeG family HAD IIIA-type phosphatase | 3 | 2 | 20.2 | 6.37 |  | cytosolic |
| WP_000715136.1 | DHH family phosphoesterase | 2 | 2 | 73 | 6.36 |  | cytosolic |
| WP_001293839.1 | aspartate carbamoyltransferase catalytic subunit | 3 | 3 | 34.7 | 6.28 |  | cytosolic |
| WP_000491799.1 | two-component system sensor histidine kinase CiaH | 2 | 2 | 50.7 | 6.14 | *ciaH* | cytosolic |
| WP_001049393.1 | MULTISPECIES: YggS family pyridoxal phosphate-dependent enzyme | 2 | 2 | 25.7 | 6.07 |  | cytosolic |
| WP_000331264.1 | tryptophan synthase subunit beta | 2 | 2 | 44.1 | 6.05 |  | cytosolic |
| WP_000454517.1 | ATP-dependent RecD-like DNA helicase | 2 | 2 | 88.2 | 6 |  | cytosolic |
| WP_000380931.1 | MULTISPECIES: GTP cyclohydrolase I FolE | 2 | 2 | 20.7 | 5.96 | *folE* | cytosolic |
| WP_004239449.1 | NAD-dependent DNA ligase LigA | 2 | 2 | 72.1 | 5.92 | *ligA* | cytosolic |
| WP_000078545.1 | ROK family glucokinase | 2 | 2 | 33.6 | 5.84 |  | cytosolic |
| WP_000360341.1 | MurR/RpiR family transcriptional regulator | 2 | 2 | 32.6 | 5.81 |  | cytosolic |
| WP_000218997.1 | branched-chain amino acid aminotransferase | 2 | 2 | 37.4 | 5.79 |  | cytosolic |
| WP_001176226.1 | helix-turn-helix transcriptional regulator | 2 | 2 | 7.9 | 5.79 |  | cytosolic |
| WP_000255759.1 | MULTISPECIES: heat-inducible transcriptional repressor HrcA | 2 | 2 | 39.3 | 5.68 | *hrcA* | cytosolic |
| WP_001280330.1 | PTS mannose/fructose/sorbose transporter subunit IIC | 2 | 2 | 27.2 | 5.66 |  | cytosolic |
| WP_078228289.1 | MULTISPECIES: TIGR01457 family HAD-type hydrolase | 2 | 2 | 28.2 | 5.66 |  | cytosolic |
| WP_000180508.1 | type I pantothenate kinase | 2 | 2 | 35.6 | 5.66 |  | cytosolic |
| WP_001160245.1 | RsmF rRNA methyltransferase first C-terminal domain-containing protein | 2 | 2 | 48.3 | 5.63 |  | cytosolic |
| WP_000521355.1 | pyridoxal phosphate-dependent aminotransferase | 2 | 2 | 44.4 | 5.61 |  | cytosolic |
| WP_000424291.1 | insulinase family protein | 2 | 2 | 47.7 | 5.61 |  | cytosolic |
| WP_000281605.1 | YdcF family protein | 2 | 2 | 39.2 | 5.57 |  | cytosolic |
| WP_000163936.1 | MULTISPECIES: aminoacyl-tRNA hydrolase | 2 | 2 | 21.4 | 5.56 |  | cytosolic |
| WP_001267186.1 | M57 family metalloprotease | 2 | 2 | 26.6 | 5.51 |  | cytosolic |
| WP_000229882.1 | MULTISPECIES: DNA starvation/stationary phase protection protein | 2 | 2 | 19.3 | 5.44 |  | cytosolic |
| WP_000941427.1 | MULTISPECIES: branched-chain amino acid ABC transporter permease | 3 | 2 | 30.8 | 5.4 |  | cytosolic |
| WP_000243928.1 | aminopeptidase | 2 | 2 | 45 | 5.4 |  | cytosolic |
| WP_000567927.1 | MULTISPECIES: Mini-ribonuclease 3 [Bacilli] | 2 | 2 | 14.7 | 5.38 |  | cytosolic |
| WP_000870440.1 | TRZ/ATZ family protein | 2 | 2 | 46.4 | 5.32 |  | cytosolic |
| WP_000873983.1 | MULTISPECIES: lipoate--protein ligase | 3 | 3 | 37.7 | 5.3 |  | cytosolic |
| WP_000638813.1 | copper homeostasis protein CutC | 2 | 2 | 23.1 | 5.27 | *cutC* | cytosolic |
| WP_001085807.1 | MULTISPECIES: 50S ribosomal protein L11 | 3 | 2 | 14.8 | 5.26 |  | cytosolic |
| WP_023946939.1 | glycerophosphodiester phosphodiesterase | 2 | 2 | 68.7 | 5.19 |  | cytosolic |
| WP_000039631.1 | NAD(P)H-dependent oxidoreductase | 2 | 2 | 19.6 | 5.18 |  | cytosolic |
| WP_004238693.1 | O-antigen polysaccharide polymerase Wzy family protein | 2 | 2 | 56.2 | 5.17 |  | cytosolic |
| WP_000794771.1 | bifunctional hydroxymethylpyrimidine kinase/phosphomethylpyrimidine kinase | 2 | 2 | 27.6 | 5.13 |  | cytosolic |
| WP_000163055.1 | MULTISPECIES: RNase adapter RapZ | 2 | 2 | 33.9 | 5.12 | *rapZ* | cytosolic |
| WP_078228369.1 | Cof-type HAD-IIB family hydrolase | 3 | 3 | 52 | 5.08 |  | cytosolic |
| WP_000659993.1 | MULTISPECIES: ribonuclease M5 | 2 | 2 | 21.6 | 5.04 |  | cytosolic |
| WP_001019941.1 | response regulator transcription factor | 2 | 2 | 28.7 | 4.97 |  | cytosolic |
| WP_000011276.1 | MULTISPECIES: DNA-binding protein WhiA | 2 | 2 | 34 | 4.82 | *whiA* | cytosolic |
| WP_000225997.1 | LacI family DNA-binding transcriptional regulator | 2 | 2 | 35.2 | 4.78 |  | cytosolic |
| WP_000129696.1 | MULTISPECIES: cystathionine beta-lyase | 2 | 2 | 23.6 | 4.66 |  | cytosolic |
| WP_000521447.1 | phosphoglucosamine mutase | 2 | 2 | 48.1 | 4.66 |  | cytosolic |
| WP_000771883.1 | glycosyltransferase family 2 protein | 2 | 2 | 39 | 4.49 |  | cytosolic |
| WP_000885084.1 | N-acetyldiaminopimelate deacetylase | 2 | 2 | 41.7 | 4.41 |  | cytosolic |
| WP_001122889.1 | type I glutamate--ammonia ligase | 2 | 2 | 50.3 | 4.29 |  | cytosolic |
| WP_000188514.1 | metal-dependent transcriptional regulator | 2 | 2 | 24.8 | 4.2 |  | cytosolic |
| WP_000011743.1 | leucine--tRNA ligase | 3 | 3 | 94.2 | 4.14 |  | cytosolic |
| WP_000992841.1 | DNA helicase PcrA | 2 | 2 | 85.8 | 3.81 | *pcrA* | cytosolic |
| WP_000639688.1 | MULTISPECIES: cysteine desulfurase | 2 | 2 | 40.7 | 3.75 |  | cytosolic |
| WP_001219140.1 | response regulator transcription factor | 2 | 2 | 29.2 | 3.64 |  | cytosolic |
| WP_001061166.1 | excinuclease ABC subunit UvrC | 2 | 2 | 70.6 | 3.62 | *uvrC* | cytosolic |
| WP_023947518.1 | CBS-HotDog domain-containing transcription factor SpxR | 3 | 3 | 47.7 | 2.92 | *spxR* | cytosolic |
| WP_000091376.1 | glutamyl-tRNA synthetase | 2 | 2 | 49.4 | 2.91 |  | cytosolic |
| WP_000754641.1 | FAD-binding oxidoreductase | 2 | 2 | 40.3 | 2.75 |  | cytosolic |
| WP_000164311.1 | MULTISPECIES: ROK family protein | 2 | 2 | 31.9 | 2.73 |  | cytosolic |
| WP_078228410.1 | GNAT family N-acetyltransferase | 2 | 2 | 21.7 | 2.54 |  | cytosolic |
| WP_000739243.1 | MULTISPECIES: ribonuclease P protein component | 2 | 2 | 14.3 | 2.49 |  | cytosolic |
| WP_000867722.1 | DEAD/DEAH box helicase | 2 | 2 | 49.6 | 2.23 |  | cytosolic |
| WP_000241437.1 | glycosyltransferase | 3 | 3 | 48.1 | 1.94 |  | cytosolic |
| WP_000924473.1 | dihydroorotase | 5 | 2 | 45.3 | 0 |  | cytosolic |
| WP_000727954.1 | peptidylprolyl isomerase PrsA | 146 | 36 | 34.2 | 479.36 | *prsA* | Lipoproteins |
| WP_000694524.1 | MetQ/NlpA family ABC transporter substrate-binding protein | 98 | 30 | 31.2 | 321.7 |  | Lipoproteins |
| WP_001226813.1 | pullulanase | 85 | 58 | 144.4 | 275.17 |  | Lipoproteins |
| WP_000747755.1 | M15 family metallopeptidase | 69 | 14 | 26.4 | 236.12 |  | Lipoproteins |
| WP_000717398.1 | peptidylprolyl isomerase | 53 | 21 | 29.1 | 176.43 |  | Lipoproteins |
| WP_000724036.1 | zinc ABC transporter substrate-binding lipoprotein AdcA | 40 | 23 | 56.3 | 127.94 | *adcA* | Lipoproteins |
| WP_000757363.1 | thiol-disulfide oxidoreductase-associated lipoprotein SdbB | 17 | 8 | 21.1 | 58.28 | *sdbB* | Lipoproteins |
| WP_000730403.1 | zinc-binding lipoprotein AdcAII | 13 | 10 | 33.9 | 45.29 |  | Lipoproteins |
| WP_000872420.1 | extracellular solute-binding protein | 9 | 8 | 46.1 | 30.17 |  | Lipoproteins |
| WP_000216346.1 | MULTISPECIES: homoserine dehydrogenase | 5 | 5 | 46.2 | 15.46 |  | Lipoproteins |
| WP_000724612.1 | CAP domain-containing protein | 186 | 34 | 43.3 | 589.83 |  | Secreted proteins |
| WP_000736102.1 | glucosaminidase domain-containing protein | 97 | 37 | 65.3 | 332.17 | *lytB* | Secreted proteins |
| WP_001227733.1 | DUF1002 domain-containing protein | 54 | 21 | 35.4 | 174.41 |  | Secreted proteins |
| WP_000720658.1 | D-alanyl-D-alanine carboxypeptidase PBP3 | 44 | 21 | 45 | 132.02 |  | Secreted proteins |
| WP_000239266.1 | LCP family protein | 35 | 13 | 38.8 | 106.08 |  | Secreted proteins |
| WP_000022821.1 | MULTISPECIES: phosphopyruvate hydratase | 33 | 17 | 46.9 | 99.79 | *eno* | Secreted proteins |
| WP_000727023.1 | CHAP domain-containing protein | 28 | 14 | 43.8 | 86.53 |  | Secreted proteins |
| WP_000751577.1 | G5 domain-containing protein | 27 | 23 | 218 | 68.29 |  | Secreted proteins |
| WP_001078994.1 | rod shape-determining protein MreC | 23 | 15 | 29.7 | 63.96 | *mreC* | Secreted proteins |
| WP_001181037.1 | alpha-amylase | 17 | 10 | 55.9 | 57.44 |  | Secreted proteins |
| WP_001009816.1 | G5 domain-containing protein | 18 | 17 | 217.2 | 51.85 |  | Secreted proteins |
| WP_000483702.1 | type I pullulanase | 4 | 4 | 86.4 | 8.3 |  | Secreted proteins |
| WP_000733052.1 | metal ABC transporter substrate-binding lipoprotein/adhesin PsaA | 923 | 39 | 34.6 | 3194.41 | *psaA* | transmembrane |
| WP_001036137.1 | MULTISPECIES: BMP family protein | 701 | 42 | 36.6 | 2834.71 | *tmpC* | transmembrane |
| WP_000095474.1 | maltodextrin ABC transporter substrate-binding protein | 430 | 45 | 45.4 | 1493.59 |  | transmembrane |
| WP_001169031.1 | Ig-like domain-containing protein | 323 | 157 | 268.4 | 1079.6 |  | transmembrane |
| WP_000724955.1 | amino acid ABC transporter substrate-binding protein | 202 | 35 | 30.7 | 693.8 |  | transmembrane |
| WP_000800391.1 | MULTISPECIES: ABC transporter substrate-binding protein | 197 | 43 | 54.7 | 658.63 |  | transmembrane |
| WP_078228356.1 | choline binding-anchored murein hydrolase LytC | 255 | 56 | 62.8 | 651.39 | *lytC* | transmembrane |
| WP_000726117.1 | MULTISPECIES: ABC transporter substrate-binding protein | 169 | 30 | 40.4 | 558.26 |  | transmembrane |
| WP_000749582.1 | MULTISPECIES: peptide ABC transporter substrate-binding protein | 133 | 42 | 72.5 | 473.4 | *amiA* | transmembrane |
| WP_000837375.1 | MULTISPECIES: ABC transporter substrate-binding protein | 140 | 25 | 29.3 | 469.6 |  | transmembrane |
| WP_000672120.1 | MULTISPECIES: ABC transporter substrate-binding protein | 143 | 29 | 48 | 423.67 |  | transmembrane |
| WP_000792146.1 | MULTISPECIES: ABC transporter substrate-binding protein | 77 | 20 | 34.6 | 336.9 |  | transmembrane |
| WP_000748522.1 | peptide ABC transporter substrate-binding protein | 103 | 38 | 72.5 | 321.87 |  | transmembrane |
| WP_000998569.1 | peptide-methionine (R)-S-oxide reductase MsrB | 107 | 29 | 41.9 | 317.52 | *msrB* | transmembrane |
| WP_000748905.1 | peptide ABC transporter substrate-binding protein | 100 | 42 | 73 | 311.73 |  | transmembrane |
| WP_001225466.1 | sugar ABC transporter substrate-binding protein | 91 | 33 | 48.4 | 305.01 |  | transmembrane |
| WP_001093128.1 | peptide ABC transporter substrate-binding protein | 76 | 28 | 72.5 | 226.35 |  | transmembrane |
| WP_000837410.1 | MULTISPECIES: peptide ABC transporter substrate-binding protein | 54 | 30 | 72.7 | 201.15 |  | transmembrane |
| WP_001041321.1 | penicillin-binding protein PBP1A | 64 | 36 | 80.7 | 195.54 |  | transmembrane |
| WP_001180942.1 | penicillin-binding protein PBP1B | 54 | 34 | 89 | 183.4 |  | transmembrane |
| WP_000754556.1 | ABC transporter substrate-binding protein | 53 | 23 | 37.8 | 182.32 |  | transmembrane |
| WP_000090252.1 | cell division protein FtsA | 48 | 18 | 49.9 | 179.04 | *ftsA* | transmembrane |
| WP_001291438.1 | endolytic transglycosylase MltG | 57 | 33 | 62.2 | 170.19 | *mltG* | transmembrane |
| WP_000872180.1 | penicillin-binding protein PBP2X | 50 | 27 | 82.3 | 166.55 |  | transmembrane |
| WP_000229944.1 | MULTISPECIES: sn-glycerol-3-phosphate ABC transporter ATP-binding protein UgpC [Bacteria] | 48 | 23 | 41.7 | 165.37 | *ugpC* | transmembrane |
| WP_000753248.1 | ABC transporter substrate-binding protein | 43 | 21 | 36.7 | 149.23 |  | transmembrane |
| WP_000731167.1 | MULTISPECIES: ABC transporter substrate-binding protein | 40 | 21 | 37.8 | 137.96 |  | transmembrane |
| WP_004238750.1 | penicillin-binding protein PBP2B | 38 | 28 | 74.1 | 127.42 |  | transmembrane |
| WP_000681587.1 | S1C family serine protease | 31 | 16 | 41.7 | 125.34 |  | transmembrane |
| WP_000858281.1 | siderophore ABC transporter substrate-binding protein | 41 | 23 | 34.6 | 124.54 |  | transmembrane |
| WP_223339950.1 | CapA family protein | 38 | 21 | 39.8 | 118.2 | *capA* | transmembrane |
| WP_000094357.1 | MULTISPECIES: F0F1 ATP synthase subunit beta | 31 | 21 | 50.9 | 112.75 | *atpD* | transmembrane |
| WP_000361597.1 | polysaccharide deacetylase family protein | 33 | 21 | 52.6 | 107.21 |  | transmembrane |
| WP_078228414.1 | ABC transporter substrate-binding protein/permease | 29 | 19 | 78.3 | 106.6 |  | transmembrane |
| WP_000046506.1 | ABC transporter ATP-binding protein | 33 | 21 | 55.3 | 104.61 |  | transmembrane |
| WP_000493216.1 | MULTISPECIES: DUF4230 domain-containing protein | 30 | 17 | 21.5 | 102.57 |  | transmembrane |
| WP_001218713.1 | MULTISPECIES: redox-regulated ATPase YchF | 29 | 17 | 41.2 | 101.71 | *ychF* | transmembrane |
| WP_000701448.1 | MULTISPECIES: fructose-specific PTS transporter subunit EIIC | 38 | 22 | 67 | 101.23 | *fruA* | transmembrane |
| WP_000762651.1 | penicillin-binding protein PBP2A | 31 | 25 | 80.6 | 97.62 |  | transmembrane |
| WP_000461491.1 | hypothetical protein | 30 | 18 | 38.6 | 93.39 |  | transmembrane |
| WP_001233683.1 | DUF4097 family beta strand repeat-containing protein | 30 | 17 | 35.5 | 91.88 |  | transmembrane |
| WP_000091629.1 | LCP family protein | 33 | 22 | 53.4 | 87.08 |  | transmembrane |
| WP_000614511.1 | Stk1 family PASTA domain-containing Ser/Thr kinase | 27 | 20 | 69.8 | 86.13 | *pknB* | transmembrane |
| WP_000800788.1 | hypothetical protein | 26 | 15 | 31.8 | 84.81 |  | transmembrane |
| WP_000974075.1 | PTS transporter subunit IIBC | 24 | 14 | 77.8 | 83.75 |  | transmembrane |
| WP_000114489.1 | MULTISPECIES: Fe-S cluster assembly ATPase SufC | 22 | 9 | 28.4 | 74.11 | *sufC* | transmembrane |
| WP_000797054.1 | MULTISPECIES: Fe-S cluster assembly protein SufB | 24 | 14 | 52.7 | 73.22 | *sufB* | transmembrane |
| WP_000808472.1 | hypothetical protein | 24 | 13 | 40.5 | 72.19 |  | transmembrane |
| WP_000727271.1 | MULTISPECIES: PDZ domain-containing protein | 21 | 10 | 37.5 | 71.57 |  | transmembrane |
| WP_004239180.1 | signal peptidase I | 31 | 12 | 23.5 | 70.88 | *lepB* | transmembrane |
| WP_001229574.1 | amino acid ABC transporter ATP-binding protein | 27 | 15 | 28.1 | 70.19 |  | transmembrane |
| WP_049547091.1 | ABC transporter substrate-binding protein/permease | 20 | 11 | 57.4 | 68.87 |  | transmembrane |
| WP_000032421.1 | cation-transporting P-type ATPase | 21 | 17 | 96.8 | 68.21 |  | transmembrane |
| WP_000159550.1 | MULTISPECIES: ABC transporter ATP-binding protein | 17 | 12 | 39.5 | 67.79 | *amiE* | transmembrane |
| WP_000472064.1 | YhgE/Pip domain-containing protein | 19 | 15 | 92.9 | 66.39 |  | transmembrane |
| WP_000022269.1 | MULTISPECIES: cell division ATP-binding protein FtsE | 24 | 14 | 25.7 | 64.9 | *ftsE* | transmembrane |
| WP_001096315.1 | MULTISPECIES: amino acid ABC transporter ATP-binding protein | 20 | 10 | 27.3 | 61.63 |  | transmembrane |
| WP_000857579.1 | peptide ABC transporter substrate-binding protein | 21 | 15 | 73 | 58.7 |  | transmembrane |
| WP_033685330.1 | MULTISPECIES: toxic anion resistance protein | 16 | 10 | 46.2 | 57.96 |  | transmembrane |
| WP_000039297.1 | cell division site-positioning protein MapZ family protein | 16 | 11 | 51.9 | 57.37 | *mapZ* | transmembrane |
| WP_000801588.1 | competence pheromone export protein ComB | 18 | 15 | 49.8 | 56.13 | *comB* | transmembrane |
| WP_000136857.1 | MULTISPECIES: PTS system mannose/fructose/sorbose family transporter subunit IID | 15 | 6 | 33.1 | 54.85 |  | transmembrane |
| WP_000662188.1 | MULTISPECIES: Wzz/FepE/Etk N-terminal domain-containing protein | 15 | 8 | 25.5 | 54.48 |  | transmembrane |
| WP_000016890.1 | septation ring formation regulator EzrA | 13 | 11 | 66.5 | 53.84 | *ezrA* | transmembrane |
| WP_000032338.1 | LCP family protein | 21 | 13 | 48.8 | 53.46 |  | transmembrane |
| WP_001047209.1 | MULTISPECIES: translation elongation factor 4 | 15 | 14 | 67.6 | 50.15 |  | transmembrane |
| WP_001219441.1 | MULTISPECIES: glycosyltransferase family 4 protein | 17 | 12 | 50.5 | 49.19 |  | transmembrane |
| WP_000301989.1 | hypothetical protein | 17 | 12 | 58.2 | 48.92 |  | transmembrane |
| WP_000759915.1 | MULTISPECIES: ABC transporter permease | 18 | 12 | 55.8 | 48.44 | *amiC* | transmembrane |
| WP_000278883.1 | response regulator transcription factor | 13 | 11 | 49.5 | 47.96 |  | transmembrane |
| WP_001291284.1 | MULTISPECIES: ATP-binding cassette domain-containing protein | 14 | 9 | 34.8 | 45.94 | *oppF* | transmembrane |
| WP_000411747.1 | MULTISPECIES: acetolactate synthase large subunit | 14 | 11 | 61.6 | 39.53 | *ilvA* | transmembrane |
| WP_000919856.1 | D-alanyl-lipoteichoic acid biosynthesis protein DltD | 16 | 13 | 48.9 | 39.2 | *dltD* | transmembrane |
| WP_000625545.1 | MULTISPECIES: permease-like cell division protein FtsX | 12 | 9 | 34.2 | 37.99 | *ftsX* | transmembrane |
| WP_000259256.1 | MULTISPECIES: rhodanese-like domain-containing protein | 12 | 6 | 14.6 | 37.04 | *moeZ* | transmembrane |
| WP_000900673.1 | RIP metalloprotease RseP | 12 | 9 | 45.9 | 36.83 | *rseP* | transmembrane |
| WP_000404934.1 | MULTISPECIES: ribonuclease Y | 15 | 14 | 60.2 | 36.61 | *rny* | transmembrane |
| WP_000746323.1 | phosphate ABC transporter substrate-binding protein PstS family protein | 10 | 7 | 30.8 | 36.38 | *pstS* | transmembrane |
| WP_000140954.1 | MULTISPECIES: amino acid ABC transporter ATP-binding protein | 12 | 9 | 26.9 | 36.11 |  | transmembrane |
| WP_000910909.1 | MULTISPECIES: DUF6110 family protein [Bacteria] | 16 | 5 | 10 | 35.63 |  | transmembrane |
| WP_000747274.1 | MULTISPECIES: 4-alpha-glucanotransferase | 10 | 9 | 58 | 34.98 | *malQ* | transmembrane |
| WP_000189477.1 | MULTISPECIES: amino acid ABC transporter ATP-binding protein | 11 | 9 | 27.9 | 34.32 |  | transmembrane |
| WP_001040919.1 | MULTISPECIES: hypothetical protein | 10 | 4 | 17.5 | 32.45 |  | transmembrane |
| WP_000996629.1 | MULTISPECIES: F0F1 ATP synthase subunit alpha | 15 | 11 | 54.5 | 32.24 |  | transmembrane |
| WP_000025380.1 | MULTISPECIES: ATP-binding cassette domain-containing protein | 11 | 8 | 58.3 | 31.93 |  | transmembrane |
| WP_000744563.1 | ATP-dependent zinc metalloprotease FtsH | 14 | 12 | 71.4 | 31.79 | *ftsH* | transmembrane |
| WP_000049857.1 | phosphate ABC transporter ATP-binding protein PstB | 12 | 9 | 30.3 | 31.7 | *pstB* | transmembrane |
| WP_001035714.1 | polysaccharide biosynthesis protein | 11 | 9 | 69.2 | 30.17 |  | transmembrane |
| WP_000811472.1 | MULTISPECIES: ABC transporter ATP-binding protein | 8 | 4 | 25.7 | 30.1 |  | transmembrane |
| WP_000026673.1 | Na/Pi cotransporter family protein | 10 | 10 | 59.4 | 29.87 |  | transmembrane |
| WP_000386356.1 | MULTISPECIES: MarR family transcriptional regulator | 9 | 5 | 16.9 | 29.02 | *mprA* | transmembrane |
| WP_000908126.1 | multidrug efflux ABC transporter subunit PatA | 9 | 8 | 62.6 | 28.81 | *patA* | transmembrane |
| WP_000590982.1 | MULTISPECIES: amino acid ABC transporter ATP-binding protein | 10 | 8 | 27.4 | 28.76 | *tcyN* | transmembrane |
| WP_000062200.1 | MULTISPECIES: ABC transporter ATP-binding protein | 11 | 8 | 25.7 | 28.53 |  | transmembrane |
| WP_004235732.1 | MULTISPECIES: SPFH domain-containing protein | 10 | 9 | 33 | 28.44 |  | transmembrane |
| WP_000584858.1 | ABC-F family ATP-binding cassette domain-containing protein | 11 | 10 | 72 | 27.97 |  | transmembrane |
| WP_000198703.1 | DUF1700 domain-containing protein | 7 | 4 | 21.4 | 27.88 |  | transmembrane |
| WP_000219823.1 | MULTISPECIES: LemA family protein | 10 | 8 | 20.6 | 27.33 | *lemA* | transmembrane |
| WP_000729020.1 | hypothetical protein | 5 | 2 | 19.9 | 26.93 |  | transmembrane |
| WP_000737964.1 | MULTISPECIES: ABC transporter substrate-binding protein | 10 | 7 | 41 | 26.07 |  | transmembrane |
| WP_033687014.1 | MULTISPECIES: large conductance mechanosensitive channel protein MscL | 6 | 3 | 13.4 | 25.75 | *mscL* | transmembrane |
| WP_000364990.1 | MULTISPECIES: YneF family protein [Bacteria] | 9 | 5 | 9.1 | 25.75 |  | transmembrane |
| WP_000159649.1 | MULTISPECIES: Fe-S cluster assembly protein SufD | 10 | 8 | 46.2 | 25.52 | *sufD* | transmembrane |
| WP_000835964.1 | MULTISPECIES: membrane protein insertase YidC | 10 | 6 | 34 | 24.85 | *yidC* | transmembrane |
| WP_001245398.1 | ABC transporter permease | 7 | 6 | 43.8 | 24.82 |  | transmembrane |
| WP_000055449.1 | ABC transporter ATP-binding protein | 9 | 7 | 25.7 | 24.6 |  | transmembrane |
| WP_000895284.1 | MULTISPECIES: ABC transporter ATP-binding protein | 10 | 8 | 33.9 | 24.12 |  | transmembrane |
| WP_000958774.1 | MULTISPECIES: ATP-binding cassette domain-containing protein | 9 | 8 | 60.8 | 23.94 | *yheS* | transmembrane |
| WP_000087130.1 | glycosyltransferase | 7 | 5 | 51.2 | 23.3 |  | transmembrane |
| WP_000913393.1 | MULTISPECIES: LapA family protein [Bacteria] | 6 | 5 | 12.7 | 23.23 | *lapA* | transmembrane |
| WP_001156785.1 | alpha-glucosidase | 7 | 5 | 62.1 | 23.04 |  | transmembrane |
| WP_001185997.1 | MULTISPECIES: ABC transporter ATP-binding protein | 10 | 8 | 28.2 | 22.88 |  | transmembrane |
| WP_000731780.1 | ABC transporter ATP-binding protein/permease | 10 | 9 | 65.4 | 22.78 |  | transmembrane |
| WP_000724823.1 | UDP-N-acetylglucosamine--N-acetylmuramyl-(pentapeptide) pyrophosphoryl-undecaprenol N-acetylglucosamine transferase | 9 | 7 | 39.4 | 22.71 | *mugG* | transmembrane |
| WP_001069289.1 | MULTISPECIES: preprotein translocase subunit YajC | 6 | 4 | 11.2 | 22.45 | *yajC* | transmembrane |
| WP_000073950.1 | hypothetical protein | 11 | 6 | 15.8 | 21.45 |  | transmembrane |
| WP_000085675.1 | methionine ABC transporter ATP-binding protein | 8 | 5 | 38.6 | 21.06 |  | transmembrane |
| WP_000687783.1 | sugar ABC transporter ATP-binding protein | 7 | 6 | 54.7 | 20.63 |  | transmembrane |
| WP_020902616.1 | competence system sensor histidine kinase ComD | 6 | 6 | 51.1 | 20.61 | *comD* | transmembrane |
| WP_001227594.1 | glycoside hydrolase family 25 protein | 6 | 5 | 30.2 | 20.48 |  | transmembrane |
| WP_000822852.1 | hypothetical protein | 8 | 7 | 59.5 | 20.4 |  | transmembrane |
| WP_000008767.1 | heavy metal translocating P-type ATPase | 7 | 7 | 77.4 | 19.94 |  | transmembrane |
| WP_000111599.1 | MULTISPECIES: UDP-N-acetylmuramate dehydrogenase | 6 | 5 | 32.9 | 19.79 |  | transmembrane |
| WP_000891462.1 | MULTISPECIES: sodium:alanine symporter family protein | 6 | 4 | 46.6 | 19.39 |  | transmembrane |
| WP_000638920.1 | cysteine desulfurase | 8 | 8 | 41.7 | 19.33 |  | transmembrane |
| WP_000031175.1 | cell division protein FtsQ/DivIB | 7 | 6 | 48 | 19.17 |  | transmembrane |
| WP_000470817.1 | MULTISPECIES: phospho-N-acetylmuramoyl-pentapeptide-transferase | 8 | 4 | 35.9 | 18.3 |  | transmembrane |
| WP_000569200.1 | MULTISPECIES: ABC transporter ATP-binding protein | 5 | 4 | 27.6 | 18.06 |  | transmembrane |
| WP_000079350.1 | MULTISPECIES: DUF956 family protein | 6 | 4 | 13.7 | 17.57 |  | transmembrane |
| WP_000681132.1 | MULTISPECIES: extracellular solute-binding protein | 6 | 6 | 58.7 | 17.56 |  | transmembrane |
| WP_000668315.1 | peptide cleavage/export ABC transporter ComA | 5 | 5 | 80.1 | 17.32 | *comA* | transmembrane |
| WP_000656561.1 | MULTISPECIES: ABC transporter ATP-binding protein | 5 | 5 | 62.7 | 16.83 |  | transmembrane |
| WP_000182947.1 | MULTISPECIES: ABC transporter permease/substrate-binding protein | 6 | 6 | 55.5 | 16.37 |  | transmembrane |
| WP_000859879.1 | MULTISPECIES: multidrug efflux ABC transporter subunit PatB | 6 | 5 | 65.5 | 16.26 | *patB* | transmembrane |
| WP_000161409.1 | DegV family protein | 5 | 5 | 30.6 | 15.46 |  | transmembrane |
| WP_000084858.1 | MULTISPECIES: hypothetical protein | 4 | 3 | 9.7 | 14.98 |  | transmembrane |
| WP_000726197.1 | transporter substrate-binding domain-containing protein | 5 | 5 | 29.3 | 14.9 |  | transmembrane |
| WP_000747270.1 | DUF5590 domain-containing protein | 5 | 3 | 18.3 | 14.83 |  | transmembrane |
| WP_000348094.1 | MULTISPECIES: aquaporin family protein | 4 | 2 | 31.8 | 14.52 |  | transmembrane |
| WP_000359028.1 | MULTISPECIES: F0F1 ATP synthase subunit delta | 5 | 3 | 20.6 | 14.25 |  | transmembrane |
| WP_000721791.1 | ABC transporter substrate-binding protein | 5 | 5 | 38.3 | 13.56 |  | transmembrane |
| WP_000831084.1 | sensor histidine kinase | 5 | 5 | 63.8 | 13.4 |  | transmembrane |
| WP_000182482.1 | MULTISPECIES: amino acid ABC transporter substrate-binding protein | 5 | 5 | 31.7 | 13.05 |  | transmembrane |
| WP_000891750.1 | MULTISPECIES: amino acid ABC transporter ATP-binding protein | 3 | 3 | 23.2 | 12.86 |  | transmembrane |
| WP_004238732.1 | cation-translocating P-type ATPase | 4 | 4 | 85.4 | 12.08 |  | transmembrane |
| WP_000500197.1 | MULTISPECIES: 1-acyl-sn-glycerol-3-phosphate acyltransferase | 4 | 4 | 28.9 | 12.06 |  | transmembrane |
| WP_000742895.1 | MULTISPECIES: ABC transporter ATP-binding protein | 5 | 4 | 44.1 | 12 |  | transmembrane |
| WP_001062781.1 | MULTISPECIES: membrane protein | 5 | 3 | 48 | 11.84 |  | transmembrane |
| WP_000133483.1 | MULTISPECIES: phosphate ABC transporter ATP-binding protein PstB | 3 | 2 | 28 | 11.79 | *pstB* | transmembrane |
| WP_000103691.1 | MULTISPECIES: ABC transporter permease | 4 | 3 | 34.5 | 11.25 |  | transmembrane |
| WP_000189380.1 | DUF1189 domain-containing protein | 5 | 3 | 30.6 | 11.19 |  | transmembrane |
| WP_000632944.1 | excinuclease ABC subunit B | 5 | 5 | 75.7 | 11.18 |  | transmembrane |
| WP_000301224.1 | MULTISPECIES: F0F1 ATP synthase subunit gamma | 4 | 3 | 32.3 | 11.11 |  | transmembrane |
| WP_000147504.1 | DUF389 domain-containing protein | 4 | 4 | 38.7 | 11.07 |  | transmembrane |
| WP_001231080.1 | YvcK family protein | 4 | 4 | 36 | 10.97 |  | transmembrane |
| WP_000038651.1 | ABC transporter permease | 4 | 3 | 37.4 | 10.74 |  | transmembrane |
| WP_000517861.1 | MULTISPECIES: cell wall synthase accessory phosphoprotein MacP | 4 | 4 | 11.7 | 10.31 | *macP* | transmembrane |
| WP_000174457.1 | DUF421 domain-containing protein | 3 | 3 | 23.3 | 9.75 |  | transmembrane |
| WP_001245187.1 | helix-turn-helix domain-containing protein | 3 | 3 | 58.8 | 9.16 |  | transmembrane |
| WP_001106639.1 | ATP-binding protein | 3 | 2 | 47 | 8.85 |  | transmembrane |
| WP_000534926.1 | YSIRK-type signal peptide-containing protein | 4 | 4 | 80.5 | 8.55 |  | transmembrane |
| WP_078228271.1 | ABC transporter ATP-binding protein/permease | 3 | 3 | 64.6 | 8.48 |  | transmembrane |
| WP_000575245.1 | ABC transporter ATP-binding protein | 4 | 4 | 27.1 | 8.15 |  | transmembrane |
| WP_001209502.1 | sugar transferase | 3 | 3 | 51.5 | 8.01 |  | transmembrane |
| WP_233280454.1 | ATP-binding protein | 2 | 2 | 77.6 | 7.93 |  | transmembrane |
| WP_000791761.1 | DUF1542 domain-containing protein | 4 | 4 | 380.7 | 7.85 |  | transmembrane |
| WP_000854373.1 | ABC transporter ATP-binding protein | 2 | 2 | 25.8 | 7.83 |  | transmembrane |
| WP_001011069.1 | MULTISPECIES: diadenylate cyclase CdaA | 2 | 2 | 31.8 | 7.39 | *cdaA* | transmembrane |
| WP_000465403.1 | MULTISPECIES: preprotein translocase subunit SecY | 3 | 2 | 47.4 | 7.28 | *secY* | transmembrane |
| WP_000869843.1 | ABC transporter substrate-binding protein | 2 | 2 | 35.4 | 6.87 |  | transmembrane |
| WP_004238796.1 | MULTISPECIES: O-antigen ligase family protein | 2 | 2 | 45.1 | 6.85 |  | transmembrane |
| WP_000242267.1 | MULTISPECIES: magnesium transporter CorA family protein | 2 | 2 | 34.8 | 6.8 | *corA* | transmembrane |
| WP_001162125.1 | MULTISPECIES: flavodoxin | 2 | 2 | 16 | 6.71 |  | transmembrane |
| WP_000470453.1 | MucBP domain-containing protein | 2 | 2 | 184.1 | 6.67 |  | transmembrane |
| WP_000029126.1 | ABC transporter permease | 2 | 2 | 33.8 | 6.65 |  | transmembrane |
| WP_000507059.1 | MULTISPECIES: IreB family regulatory phosphoprotein | 2 | 2 | 10.2 | 6.36 |  | transmembrane |
| WP_000414973.1 | sugar ABC transporter permease | 2 | 2 | 47.6 | 5.98 |  | transmembrane |
| WP_000703370.1 | FtsW/RodA/SpoVE family cell cycle protein | 2 | 2 | 44.9 | 5.94 |  | transmembrane |
| WP_001011639.1 | MULTISPECIES: amino acid ABC transporter permease | 2 | 2 | 24.9 | 5.91 |  | transmembrane |
| WP_000554841.1 | aminodeoxychorismate synthase component I | 4 | 3 | 65.8 | 5.66 |  | transmembrane |
| WP_000709046.1 | biotin transporter BioY | 2 | 2 | 18.7 | 5.62 | *bioY* | transmembrane |
| WP_004255928.1 | UDP-glucose 4-epimerase GalE | 2 | 2 | 37.1 | 5.57 | *galE* | transmembrane |
| WP_000403207.1 | RluA family pseudouridine synthase | 2 | 2 | 32.9 | 5.51 |  | transmembrane |
| WP_001220352.1 | MULTISPECIES: YbaN family protein | 2 | 2 | 13.7 | 5.5 | *ybaN* | transmembrane |
| WP_000725716.1 | hypothetical protein | 3 | 3 | 24.6 | 5.44 |  | transmembrane |
| WP_001291280.1 | restriction endonuclease subunit S | 3 | 3 | 44 | 5.4 |  | transmembrane |
| WP_001188174.1 | 16S rRNA (uracil(1498)-N(3))-methyltransferase | 2 | 2 | 27.2 | 5.17 |  | transmembrane |
| WP_000412287.1 | alpha-galactosylglucosyldiacylglycerol synthase | 2 | 2 | 40.1 | 4.93 |  | transmembrane |
| WP_000477437.1 | MULTISPECIES: PTS transporter subunit EIIC | 2 | 2 | 54.5 | 4.9 |  | transmembrane |
| WP_000185285.1 | MULTISPECIES: PTS system mannose/fructose/sorbose family transporter subunit IID | 3 | 2 | 30.2 | 4.83 |  | transmembrane |
| WP_000748405.1 | MptD family putative ECF transporter S component | 2 | 2 | 20.1 | 4.76 |  | transmembrane |
| WP_001037324.1 | MULTISPECIES: lactose-specific PTS transporter subunit EIIC | 3 | 2 | 61 | 4.54 |  | transmembrane |
| WP_000843636.1 | energy-coupling factor transporter ATPase | 2 | 2 | 31.2 | 4.45 |  | transmembrane |
| WP_001269496.1 | MULTISPECIES: metal ABC transporter ATP-binding protein | 2 | 2 | 26.6 | 4.26 |  | transmembrane |
| WP_004238894.1 | membrane protein insertase YidC | 3 | 3 | 31.1 | 3.33 | *yidC* | transmembrane |
| WP_000571617.1 | ABC transporter ATP-binding protein | 2 | 2 | 24.7 | 3.04 |  | transmembrane |
| WP_161969980.1 | dihydroorotate dehydrogenase | 2 | 2 | 34.9 | 2.67 |  | transmembrane |
| WP_001149091.1 | M1 family metallopeptidase | 2 | 2 | 95.4 | 2.66 |  | transmembrane |
| WP_001016903.1 | MULTISPECIES: Bax inhibitor-1/YccA family protein | 3 | 2 | 24.9 | 2.18 |  | transmembrane |
| WP_000571163.1 | cell wall metabolism sensor histidine kinase VicK | 2 | 2 | 51.8 | 2.05 | *vicK* | transmembrane |
| WP_001259053.1 | ABC transporter permease | 2 | 2 | 40 | 1.84 |  | transmembrane |
| WP_000764978.1 | YfhO family protein | 2 | 2 | 97 | 1.7 | *yfhO* | transmembrane |
| WP_000889948.1 | ABC transporter ATP-binding protein | 2 | 2 | 27.1 | 0 |  | transmembrane |
| WP_000725153.1 | MULTISPECIES: hypothetical protein | 59 | 18 | 21 | 210.39 |  |  |
| WP_000272527.1 | UDP-galactopyranose mutase | 29 | 19 | 43.4 | 93.96 | *glf* |  |
| WP_000730894.1 | MULTISPECIES: hypothetical protein | 19 | 8 | 23.6 | 60.93 |  |  |
| WP_000856507.1 | diaminopimelate decarboxylase | 19 | 11 | 46.6 | 58.86 |  |  |
| WP_004238710.1 | tetratricopeptide repeat protein | 7 | 7 | 47.5 | 24.13 |  |  |
| WP_001230940.1 | hypothetical protein | 8 | 6 | 27.9 | 21.91 |  |  |
| WP_161969978.1 | hypothetical protein | 5 | 5 | 60 | 18.3 |  |  |
| WP_001051780.1 | MULTISPECIES: DUF3042 family protein | 5 | 5 | 6 | 13.72 |  |  |
| WP_161969976.1 | hypothetical protein | 3 | 3 | 18.5 | 12.76 |  |  |
| WP_078228331.1 | hypothetical protein | 3 | 3 | 33.9 | 12.56 |  |  |
| WP_000290006.1 | esterase family protein | 4 | 3 | 29.8 | 12.01 |  |  |
| WP_078228367.1 | Cof-type HAD-IIB family hydrolase | 3 | 3 | 30.7 | 11.53 |  |  |
| WP_000863906.1 | MULTISPECIES: PH domain-containing protein | 3 | 3 | 16.9 | 11.25 |  |  |
| WP_000858860.1 | magnesium-translocating P-type ATPase | 3 | 3 | 98.3 | 11.19 |  |  |
| WP_000078009.1 | DUF1129 domain-containing protein | 3 | 3 | 25.4 | 10.81 |  |  |
| WP_000290664.1 | GDSL-type esterase/lipase family protein | 3 | 3 | 24 | 9.39 |  |  |
| WP_000858732.1 | DUF1836 domain-containing protein | 5 | 4 | 16.9 | 9 |  |  |
| WP_000006744.1 | Rqc2 family fibronectin-binding protein PavA | 5 | 5 | 63.4 | 8.22 | *pavA* |  |
| WP_004239161.1 | hypothetical protein | 3 | 3 | 30.8 | 7.55 |  |  |
| WP_000415506.1 | hypothetical protein | 2 | 2 | 41.5 | 5.98 |  |  |
| WP_001102236.1 | proteinase | 2 | 2 | 27.6 | 5.48 |  |  |
| WP_078228436.1 | transketolase family protein | 2 | 2 | 34.5 | 4.73 |  |  |

Accession, Identification numbers assigned to the nucleotide sequences of genes published by the DNA Data Base of Japan. #PSM, Peptide-spectrum match. #peptides, Different types of detection peptides derived from the same protein. Score, Values obtained from mass spectrometry measurement results.
