# Supplementary material for: Functional characteristics of membrane vesicles produced by Streptococcus mitis
Source: J Oral Microbiol. 2025 Sep 23;17(1):2557962. doi: 10.1080/20002297.2025.2557962 (PMC12459155; doi:10.1080/20002297.2025.2557962)
Supplement: Supplementary material — Supplementary Table 3 [file ZJOM_A_2557962_SM1727.docx]

**Supplementary Table 3.** Mass spectrometry identification of *S. mitis* Nm-65 proteins present in MVs and their topology prediction

| Accession | Description | # PSMs | # Peptides | MW [kDa] | Score | Gene | Localization |
| --- | --- | --- | --- | --- | --- | --- | --- |
| BCJ09676.1 | choline-binding protein C | 553 | 36 | 43.6 | 4513.15 | *cbp4_1* | cell wall |
| BCJ11288.1 | choline-binding protein C | 217 | 25 | 45 | 704.68 | *cbp4_2* | cell wall |
| BCJ10066.1 | choline-binding protein A | 104 | 25 | 41.5 | 316.85 | *cbp5* | cell wall |
| BCJ09707.1 | choline-binding protein C | 100 | 22 | 43 | 282.89 | *cbpI* | cell wall |
| BCJ09890.1 | choline-binding protein J | 44 | 16 | 39.4 | 131.31 | *cbp9_1* | cell wall |
| BCJ10745.1 | choline-binding protein E | 38 | 21 | 70.1 | 120.37 | *pce1* | cell wall |
| BCJ11296.1 | choline-binding protein J | 13 | 7 | 35.3 | 41.14 | *cbp9_2* | cell wall |
| BCJ10861.1 | ribitol-5-phosphate cytidylyltransferase | 10 | 5 | 26.2 | 20.92 | *ispD;tarI* | cell wall |
| BCJ10717.1 | adhesion protein | 5 | 4 | 33.9 | 13.21 | *lmb* | cell wall |
| BCJ10276.1 | glutamine amidotransferase | 5 | 3 | 29.1 | 19.35 | *cobQ* | cell wall |
| BCJ10174.1 | alanine racemase | 3 | 2 | 39.9 | 11.01 |  | cell wall |
| BCJ09900.1 | N-acetylmuramoyl-L-alanine amidase | 2 | 2 | 36.5 | 3.18 | *lytA* | cell wall |
| BCJ10391.1 | phosphorylcholine transferase LicD | 2 | 2 | 32.5 | 7.08 | *licD3* | cell wall |
| BCJ11408.1 | aldehyde-alcohol dehydrogenase | 316 | 40 | 97.1 | 906.39 | *adhE* | cytosolic |
| BCJ10519.1 | elongation factor Tu | 280 | 27 | 44 | 855.25 | *tuf* | cytosolic |
| BCJ11147.1 | 1,4-beta-N-acetylmuramidase | 234 | 34 | 56.6 | 716.99 | *lytC* | cytosolic |
| BCJ11404.1 | glyceraldehyde-3-phosphate dehydrogenase | 222 | 21 | 35.9 | 738.84 | *gap* | cytosolic |
| BCJ09617.1 | phage capsid protein | 109 | 21 | 43.5 | 457.28 | *pi233* | cytosolic |
| BCJ09888.1 | DNA-directed RNA polymerase subunit beta' | 107 | 57 | 137 | 280.07 | *rpoC* | cytosolic |
| BCJ10251.1 | threonine--tRNA ligase | 99 | 33 | 74.6 | 278.96 | *thrRS* | cytosolic |
| BCJ11581.1 | 30S ribosomal protein S2 | 91 | 19 | 28.8 | 295.9 | *rpsB* | cytosolic |
| BCJ09821.1 | 50S ribosomal protein L2 | 83 | 12 | 29.9 | 220.5 | *rplB* | cytosolic |
| BCJ10567.1 | pyruvate kinase | 82 | 28 | 54.7 | 245.55 | *pykF* | cytosolic |
| BCJ10046.1 | translation initiation factor IF-2 | 78 | 29 | 103.1 | 228.39 | *infB* | cytosolic |
| BCJ09887.1 | DNA-directed RNA polymerase subunit beta | 75 | 39 | 133.9 | 217.78 | *rpoB* | cytosolic |
| BCJ11063.1 | lysozyme | 69 | 15 | 38.8 | 242.52 |  | cytosolic |
| BCJ09925.1 | formate acetyltransferase | 68 | 31 | 87.8 | 183.04 | *pfl* | cytosolic |
| BCJ09825.1 | 50S ribosomal protein L16 | 67 | 4 | 15.4 | 188.34 | *rplP* | cytosolic |
| BCJ09938.1 | 6-phospho-beta-glucosidase | 67 | 22 | 54.4 | 193.93 | *bglH* | cytosolic |
| BCJ10423.1 | peptidyl-prolyl cis-trans isomerase | 61 | 19 | 29.1 | 197.78 | *ppiA* | cytosolic |
| BCJ10548.1 | phosphoenolpyruvate carboxylase | 60 | 36 | 103.2 | 157.67 | *ppc* | cytosolic |
| BCJ11386.1 | elongation factor G | 58 | 31 | 76.8 | 180.04 | *fusA* | cytosolic |
| BCJ09881.1 | proline--tRNA ligase | 57 | 25 | 68.6 | 155.3 | *proS* | cytosolic |
| BCJ10923.1 | lysine--tRNA ligase | 55 | 23 | 56.6 | 158.82 | *lysS* | cytosolic |
| BCJ11022.1 | pyruvate oxidase | 53 | 20 | 65.2 | 181.15 | *spxB* | cytosolic |
| BCJ10210.1 | cell division protein FtsZ | 51 | 22 | 44.4 | 162.49 | *ftsZ* | cytosolic |
| BCJ11466.1 | alpha-1,4 glucan phosphorylase | 50 | 20 | 85.1 | 147.73 | *malP* | cytosolic |
| BCJ11276.1 | serine--tRNA ligase | 49 | 23 | 47.6 | 170.49 | *serS* | cytosolic |
| BCJ11412.1 | transketolase | 49 | 22 | 71.1 | 152.78 | *tktA* | cytosolic |
| BCJ10963.1 | metallophosphatase | 49 | 18 | 50.1 | 151.13 |  | cytosolic |
| BCJ10880.1 | signal recognition particle protein | 48 | 22 | 57.7 | 123.7 | *ffh* | cytosolic |
| BCJ10218.1 | 2,3-bisphosphoglycerate-dependent phosphoglycerate mutase | 48 | 15 | 26 | 113.45 | *gpmA;* | cytosolic |
| BCJ10215.1 | cell division protein DivIVA | 47 | 14 | 33.1 | 169.69 | *divIVA* | cytosolic |
| BCJ09914.1 | kinase | 47 | 20 | 60 | 147.03 |  | cytosolic |
| BCJ10698.1 | serine hydroxymethyltransferase | 46 | 23 | 45.2 | 126.47 | *glyA* | cytosolic |
| BCJ10985.1 | FMN reductase | 46 | 20 | 45.5 | 146.01 |  | cytosolic |
| BCJ09830.1 | 50S ribosomal protein L5 | 45 | 13 | 19.8 | 132.19 | *rplE* | cytosolic |
| BCJ11256.1 | glutamyl-tRNA(Gln) amidotransferase subunit A | 45 | 19 | 51.9 | 138.87 | *gatA* | cytosolic |
| BCJ10615.1 | ribonucleoside-diphosphate reductase | 45 | 26 | 81.5 | 113.61 | *nrdE* | cytosolic |
| BCJ11287.1 | trigger factor | 43 | 16 | 47.3 | 138.34 | *tig* | cytosolic |
| BCJ11156.1 | D-alanyl-D-alanine carboxypeptidase | 43 | 11 | 26.4 | 131.23 |  | cytosolic |
| BCJ11112.1 | asparagine--tRNA ligase | 41 | 24 | 51.1 | 109.86 | *asnS* | cytosolic |
| BCJ09941.1 | phenylalanine--tRNA ligase beta subunit | 40 | 21 | 87 | 131.64 | *pheT* | cytosolic |
| BCJ11042.1 | GTP-binding protein | 39 | 20 | 68.1 | 116.8 | *bipA;* | cytosolic |
| BCJ11474.1 | aspartate--tRNA ligase | 39 | 19 | 66.1 | 105.47 | *aspS* | cytosolic |
| BCJ09623.1 | phage tail protein | 38 | 8 | 21.4 | 109.66 | *pi239* | cytosolic |
| BCJ09841.1 | 30S ribosomal protein S13 | 37 | 15 | 13.4 | 84 | *rpsM* | cytosolic |
| BCJ10987.1 | glycine--tRNA ligase beta subunit | 37 | 21 | 72.2 | 115.31 | *glyS* | cytosolic |
| BCJ11387.1 | 30S ribosomal protein S7 | 36 | 10 | 17.7 | 116.13 | *rpsG* | cytosolic |
| BCJ10437.1 | methionine--tRNA ligase | 35 | 22 | 75.6 | 94.29 | *metS* | cytosolic |
| BCJ11302.1 | cell cycle protein GpsB | 34 | 7 | 12.9 | 126.81 | *gpsB* | cytosolic |
| BCJ10163.1 | GntR family transcriptional regulator | 34 | 12 | 14 | 107.01 |  | cytosolic |
| BCJ11167.1 | peptidoglycan branched peptide synthesis protein | 33 | 19 | 44.5 | 97.17 | *murN* | cytosolic |
| BCJ09824.1 | 30S ribosomal protein S3 | 32 | 12 | 24 | 83.45 | *rpsC* | cytosolic |
| BCJ09883.1 | glutamine--fructose-6-phosphate aminotransferase [isomerizing] | 32 | 19 | 65.4 | 106.44 | *glmS* | cytosolic |
| BCJ10023.1 | chaperone protein DnaK | 32 | 19 | 64.7 | 99.17 | *dnaK* | cytosolic |
| BCJ11254.1 | peptide chain release factor 3 | 31 | 18 | 58.4 | 87.87 | *prfC* | cytosolic |
| BCJ09818.1 | 50S ribosomal protein L3 | 30 | 4 | 22.2 | 80.67 | *rplC* | cytosolic |
| BCJ10568.1 | L-lactate dehydrogenase | 30 | 12 | 35.3 | 94.4 | *ldh* | cytosolic |
| BCJ10372.1 | alanine--tRNA ligase | 29 | 17 | 96.3 | 57.7 | *alaRS* | cytosolic |
| BCJ10013.1 | phosphoglycerate kinase | 29 | 16 | 41.9 | 96.52 | *pgk* | cytosolic |
| BCJ10566.1 | ATP-dependent 6-phosphofructokinase | 29 | 11 | 35.3 | 91.96 | *pfkA* | cytosolic |
| BCJ09979.1 | hypothetical protein SMNM65_04110 | 29 | 13 | 55.3 | 82.9 | *coaD* | cytosolic |
| BCJ10508.1 | 30S ribosomal protein S1 | 29 | 15 | 43.9 | 59.57 | *rpsA* | cytosolic |
| BCJ11168.1 | serine/alanine adding enzyme | 29 | 19 | 47.3 | 84.72 | *murM* | cytosolic |
| BCJ10830.1 | glucose-6-phosphate 1-dehydrogenase | 29 | 18 | 56.7 | 80.84 | *zwf* | cytosolic |
| BCJ11484.1 | histidine--tRNA ligase | 28 | 17 | 48.2 | 78.5 | *hisS* | cytosolic |
| BCJ11361.1 | aspartate aminotransferase | 28 | 16 | 45.7 | 76.18 | *aspC2* | cytosolic |
| BCJ10264.1 | UDP-glucose 4-epimerase | 28 | 16 | 37.4 | 86.05 |  | cytosolic |
| BCJ10517.1 | D-alanyl-D-alanine carboxypeptidase | 28 | 15 | 34.5 | 83.12 | *dacA* | cytosolic |
| BCJ10895.1 | 50S ribosomal protein L19 | 27 | 8 | 13.1 | 58.67 | *rplS* | cytosolic |
| BCJ09698.1 | adenylosuccinate lyase | 26 | 17 | 49.5 | 80.51 | *purB* | cytosolic |
| BCJ11266.1 | 3-oxoacyl-[acyl-carrier-protein] synthase 2 | 26 | 14 | 43.8 | 94.16 | *fabB* | cytosolic |
| BCJ11269.1 | enoyl-ACP reductase II | 26 | 13 | 34 | 92.36 | *fabK* | cytosolic |
| BCJ09638.1 | endonuclease | 26 | 7 | 12.1 | 89.47 |  | cytosolic |
| BCJ09587.1 | adenylosuccinate synthetase | 25 | 16 | 49.2 | 63.19 | *purA* | cytosolic |
| BCJ11564.1 | ATP-dependent DNA helicase RecG | 25 | 16 | 55.1 | 62.72 |  | cytosolic |
| BCJ10794.1 | phosphoglucomutase | 25 | 16 | 62.6 | 78.63 | *pgmA* | cytosolic |
| BCJ10905.1 | hypothetical protein SMNM65_13370 | 25 | 15 | 53.1 | 59.74 |  | cytosolic |
| BCJ09977.1 | aspartate--ammonia ligase | 24 | 12 | 37.5 | 78.01 | *asnA* | cytosolic |
| BCJ11298.1 | 6-phosphogluconate dehydrogenase, decarboxylating | 24 | 13 | 44.7 | 67.91 | *gnd* | cytosolic |
| BCJ10720.1 | thioredoxin | 24 | 10 | 20.7 | 66.7 |  | cytosolic |
| BCJ10403.1 | 50S ribosomal protein L10 | 23 | 9 | 17.5 | 63.94 | *rplJ* | cytosolic |
| BCJ11154.1 | 50S ribosomal protein L1 | 23 | 8 | 24.5 | 40.17 | *rl1; rplA* | cytosolic |
| BCJ10993.1 | DEAD-box ATP-dependent RNA helicase CshB | 23 | 15 | 52 | 64.91 | *rheB* | cytosolic |
| BCJ11580.1 | elongation factor Ts | 23 | 10 | 37.4 | 80.55 | *tsf* | cytosolic |
| BCJ09835.1 | 30S ribosomal protein S5 | 23 | 11 | 17 | 55.23 | *rpsE* | cytosolic |
| BCJ11374.1 | zinc-dependent alcohol dehydrogenase | 23 | 13 | 35.7 | 74.21 | *adhP* | cytosolic |
| BCJ10297.1 | fructose-bisphosphate aldolase | 23 | 12 | 31.4 | 69.16 | *fba; fbaA* | cytosolic |
| BCJ11219.1 | ribosome biogenesis GTPase YqeH | 23 | 12 | 40.9 | 71.82 |  | cytosolic |
| BCJ10252.1 | 30S ribosomal protein S15 | 22 | 6 | 10.5 | 68.43 | *rpsO* | cytosolic |
| BCJ09842.1 | 30S ribosomal protein S11 | 22 | 4 | 13.4 | 72.84 | *rpsK* | cytosolic |
| BCJ11596.1 | tryptophan--tRNA ligase | 22 | 12 | 38.4 | 51.55 | *trpS* | cytosolic |
| BCJ09833.1 | 50S ribosomal protein L6 | 22 | 7 | 19.5 | 69.51 | *rplF* | cytosolic |
| BCJ10516.1 | Fe-S cluster assembly protein SufB | 22 | 13 | 54.5 | 52.57 |  | cytosolic |
| BCJ09957.1 | pur operon repressor | 22 | 10 | 29.1 | 72.62 | *purR* | cytosolic |
| BCJ09820.1 | 50S ribosomal protein L23 | 21 | 8 | 10.8 | 50.5 | *rplW* | cytosolic |
| BCJ11180.1 | hypothetical protein SMNM65_16120 | 21 | 15 | 73.6 | 59.69 | *cysS* | cytosolic |
| BCJ09939.1 | phenylalanine--tRNA ligase alpha subunit | 21 | 12 | 39.1 | 47.11 | *pheS* | cytosolic |
| BCJ11257.1 | aspartyl/glutamyl-tRNA(Asn/Gln) amidotransferase subunit B | 21 | 12 | 53.7 | 63.14 | *gatB* | cytosolic |
| BCJ11388.1 | 30S ribosomal protein S12 | 21 | 7 | 15.1 | 55.02 | *rpsL* | cytosolic |
| BCJ10723.1 | 50S ribosomal protein L20 | 21 | 4 | 13.7 | 67.62 | *rplT* | cytosolic |
| BCJ11378.1 | aminopeptidase C | 21 | 16 | 50.2 | 53.24 | *pepC* | cytosolic |
| BCJ10939.1 | peptidase | 21 | 15 | 47.9 | 59.8 | *yhbU_1* | cytosolic |
| BCJ10982.1 | NADH oxidase | 21 | 11 | 50.2 | 66.96 | *nox* | cytosolic |
| BCJ09671.1 | phosphate acyltransferase | 20 | 11 | 35.1 | 60.1 | *plsX* | cytosolic |
| BCJ10609.1 | 6-phospho-beta-galactosidase | 20 | 16 | 53.8 | 59.05 | *lacG* | cytosolic |
| BCJ10864.1 | phosphorylcholine transferase LicD | 20 | 9 | 32 | 46.66 | *licD2* | cytosolic |
| BCJ10056.1 | helicase | 20 | 8 | 14.4 | 48.94 |  | cytosolic |
| BCJ09823.1 | 50S ribosomal protein L22 | 19 | 5 | 12.2 | 60.71 | *rplV* | cytosolic |
| BCJ10569.1 | DNA gyrase subunit A | 19 | 15 | 91.9 | 51.41 | *gyrA* | cytosolic |
| BCJ10618.1 | phosphoenolpyruvate-protein phosphotransferase | 19 | 10 | 63.2 | 55.63 | *ptsI* | cytosolic |
| BCJ10767.1 | glucose-1-phosphate adenylyltransferase subunit GlgD | 19 | 12 | 42.6 | 57.38 | *glgD* | cytosolic |
| BCJ10113.1 | tryptophan synthase beta chain | 19 | 10 | 42.5 | 64.69 | *trpB_2* | cytosolic |
| BCJ11146.1 | DNA starvation/stationary phase protection protein | 19 | 8 | 19.3 | 63.39 | *dps* | cytosolic |
| BCJ10216.1 | isoleucine--tRNA ligase | 18 | 14 | 105.2 | 42.02 | *ileS* | cytosolic |
| BCJ10953.1 | putative tRNA sulfurtransferase | 18 | 13 | 45.1 | 40.38 | *thiI* | cytosolic |
| BCJ11441.1 | glucose-6-phosphate isomerase | 18 | 11 | 49.9 | 53.49 | *gpi; pgi* | cytosolic |
| BCJ11152.1 | adenine phosphoribosyltransferase | 18 | 10 | 18.7 | 48.28 | *apt* | cytosolic |
| BCJ11546.1 | chaperone protein ClpB | 18 | 15 | 90.2 | 45.01 | *clpC* | cytosolic |
| BCJ09822.1 | 30S ribosomal protein S19 | 17 | 5 | 10.7 | 46.2 | *rplV* | cytosolic |
| BCJ09798.1 | UvrABC system protein A | 17 | 15 | 87.7 | 38.25 | *uvrA* | cytosolic |
| BCJ11183.1 | polyribonucleotide nucleotidyltransferase | 17 | 11 | 80.9 | 40.34 | *pnp_2* | cytosolic |
| BCJ09615.1 | phage portal protein | 17 | 11 | 45.3 | 48.09 | *pi231* | cytosolic |
| BCJ11007.1 | uracil phosphoribosyltransferase | 17 | 9 | 23.6 | 47.44 | *upp* | cytosolic |
| BCJ11272.1 | MarR family transcriptional regulator | 17 | 7 | 16.8 | 45.51 |  | cytosolic |
| BCJ10735.1 | uridylate kinase | 16 | 5 | 25.9 | 41.69 | *pyrH* | cytosolic |
| BCJ10237.1 | GTP pyrophosphokinase | 16 | 14 | 84.5 | 32.9 | *relA* | cytosolic |
| BCJ10464.1 | ATP-dependent Clp protease ATP-binding subunit | 16 | 13 | 86.4 | 42.82 | *clpE* | cytosolic |
| BCJ10943.1 | GMP synthase [glutamine-hydrolyzing] | 16 | 12 | 57.5 | 48.98 | *guaA* | cytosolic |
| BCJ10448.1 | aminopeptidase | 16 | 12 | 95.3 | 45.08 | *pepN* | cytosolic |
| BCJ11454.1 | 2,3,4,5-tetrahydropyridine-2,6-dicarboxylate N-acetyltransferase | 16 | 8 | 23.9 | 60.04 | *dapH* | cytosolic |
| BCJ11414.1 | DNA-binding protein | 16 | 5 | 37.2 | 26.89 |  | cytosolic |
| BCJ11261.1 | acetyl-coenzyme A carboxylase carboxyl transferase subunit alpha | 15 | 11 | 28.2 | 40.27 | *accA* | cytosolic |
| BCJ10279.1 | DEAD-box ATP-dependent RNA helicase CshA | 15 | 10 | 58.9 | 43.58 | *cshA_2* | cytosolic |
| BCJ11148.1 | triosephosphate isomerase | 15 | 7 | 26.5 | 46.23 | *tpiA* | cytosolic |
| BCJ10280.1 | GTP-sensing transcriptional pleiotropic repressor CodY | 14 | 10 | 29.8 | 37.77 | *codY* | cytosolic |
| BCJ10421.1 | putative dual-specificity RNA methyltransferase RlmN | 14 | 9 | 41.3 | 38.75 | *rlmN* | cytosolic |
| BCJ09898.1 | protein RecA | 14 | 9 | 38.8 | 40.98 | *recA* | cytosolic |
| BCJ10736.1 | methylenetetrahydrofolate--tRNA-(uracil-5-)-methyltransferase TrmFO | 14 | 9 | 49.2 | 53.68 | *gid* | cytosolic |
| BCJ11359.1 | 30S ribosomal protein S9 | 14 | 6 | 14.2 | 31.39 | *rpsI* | cytosolic |
| BCJ10787.1 | hypothetical protein SMNM65_12190 | 14 | 12 | 176.7 | 42.97 |  | cytosolic |
| BCJ10112.1 | capsular polysaccharide biosynthesis protein | 14 | 11 | 45.4 | 37.56 | *wecE* | cytosolic |
| BCJ10758.1 | hypothetical protein SMNM65_11900 | 14 | 8 | 47.6 | 38.82 |  | cytosolic |
| BCJ11010.1 | hypothetical protein SMNM65_14420 | 14 | 8 | 30.7 | 37.68 |  | cytosolic |
| BCJ09917.1 | ketol-acid reductoisomerase (NADP(+)) | 14 | 8 | 37.3 | 45.74 | *ilvC* | cytosolic |
| BCJ10546.1 | hypothetical protein SMNM65_09780 | 14 | 4 | 5.7 | 34.68 |  | cytosolic |
| BCJ11384.1 | DNA polymerase III PolC-type | 13 | 12 | 145.7 | 35.59 | *polC* | cytosolic |
| BCJ11417.1 | acetate kinase | 13 | 9 | 43.3 | 37.76 | *ackA* | cytosolic |
| BCJ10737.1 | ribosomal RNA small subunit methyltransferase I | 13 | 8 | 32.8 | 34.72 | *rsmI* | cytosolic |
| BCJ11575.1 | ribosome-associated protein | 13 | 6 | 21.1 | 44.98 | *hpf; raiA* | cytosolic |
| BCJ10212.1 | cell division protein SepF | 13 | 5 | 20.6 | 45.55 | *sepF* | cytosolic |
| BCJ09832.1 | 30S ribosomal protein S8 | 13 | 6 | 14.8 | 26.16 | *rpsH* | cytosolic |
| BCJ09817.1 | 30S ribosomal protein S10 | 13 | 6 | 11.6 | 24.67 | *rpsJ* | cytosolic |
| BCJ11144.1 | ATP-dependent Clp protease ATP-binding subunit ClpX | 13 | 7 | 45.7 | 28.25 | *clpX* | cytosolic |
| BCJ10889.1 | adenylate cyclase | 13 | 6 | 22.2 | 36.23 |  | cytosolic |
| BCJ11430.1 | queuine tRNA-ribosyltransferase | 13 | 5 | 43.1 | 42.39 | *tgt* | cytosolic |
| BCJ11589.1 | hypothetical protein SMNM65_20210 | 13 | 4 | 14.7 | 49.34 |  | cytosolic |
| BCJ09829.1 | 50S ribosomal protein L24 | 12 | 4 | 11 | 39.52 | *rplX* | cytosolic |
| BCJ09827.1 | 30S ribosomal protein S17 | 12 | 2 | 10 | 28.37 | *rplN* | cytosolic |
| BCJ09980.1 | UDP-N-acetylglucosamine 1-carboxyvinyltransferase | 12 | 9 | 46 | 33.14 | *murA* | cytosolic |
| BCJ10356.1 | spermidine/putrescine ABC transporter substrate-binding protein | 12 | 9 | 41 | 36.11 |  | cytosolic |
| BCJ11030.1 | amino acid ABC transporter substrate-binding protein | 12 | 8 | 31.3 | 39.8 |  | cytosolic |
| BCJ09843.1 | DNA-directed RNA polymerase subunit alpha | 12 | 7 | 34.2 | 12.43 | *rpoA* | cytosolic |
| BCJ11582.1 | amidase | 12 | 9 | 41.6 | 33.94 | *gsp-781* | cytosolic |
| BCJ09741.1 | amylovoran biosynthesis protein AmsE | 12 | 7 | 31.7 | 40.04 |  | cytosolic |
| BCJ11360.1 | 50S ribosomal protein L13 | 11 | 5 | 16.1 | 32.77 | *rplM* | cytosolic |
| BCJ09837.1 | 50S ribosomal protein L15 | 11 | 5 | 15.4 | 18.32 | *rplO* | cytosolic |
| BCJ11262.1 | acetyl-coenzyme A carboxylase carboxyl transferase subunit beta | 11 | 7 | 31.9 | 27.83 | *accD* | cytosolic |
| BCJ10404.1 | 50S ribosomal protein L7/L12 | 11 | 7 | 12.4 | 34.51 | *rplL* | cytosolic |
| BCJ09819.1 | 50S ribosomal protein L4 | 11 | 6 | 22.2 | 26.03 | *rplD* | cytosolic |
| BCJ11075.1 | nicotinate phosphoribosyltransferase | 11 | 9 | 55 | 32.26 |  | cytosolic |
| BCJ11143.1 | putative GTP-binding protein EngB | 11 | 8 | 22.3 | 30.73 | *engB* | cytosolic |
| BCJ10065.1 | hypothetical protein SMNM65_04970 | 11 | 8 | 40.5 | 35.04 |  | cytosolic |
| BCJ10669.1 | RNA-binding transcriptional accessory protein | 11 | 8 | 78.6 | 33.45 | *tex* | cytosolic |
| BCJ10008.1 | UDP-glucose 4-epimerase | 11 | 8 | 37.1 | 32.33 | *galE* | cytosolic |
| BCJ10500.1 | hypothetical protein SMNM65_09320 | 11 | 8 | 45.5 | 28.33 |  | cytosolic |
| BCJ10606.1 | transcription antiterminator lact | 11 | 7 | 32.9 | 24.34 | *lacT* | cytosolic |
| BCJ10317.1 | hypothetical protein SMNM65_07490 | 11 | 4 | 14.6 | 39.15 |  | cytosolic |
| BCJ09844.1 | 50S ribosomal protein L17 | 10 | 6 | 14.5 | 26.29 | *rplQ* | cytosolic |
| BCJ11103.1 | 30S ribosomal protein S18 | 10 | 6 | 9.2 | 29.4 | *rpsR* | cytosolic |
| BCJ09759.1 | ribonuclease J | 10 | 9 | 61 | 25.55 | *rnj_1* | cytosolic |
| BCJ10082.1 | 60 kDa chaperonin | 10 | 9 | 57 | 25.94 | *groL* | cytosolic |
| BCJ11208.1 | guanylate kinase | 10 | 8 | 23.8 | 30.85 | *gmk* | cytosolic |
| BCJ10805.1 | NAD kinase | 10 | 8 | 30.9 | 28.45 | *nadK* | cytosolic |
| BCJ10347.1 | phosphate transport system regulatory protein PhoU | 10 | 8 | 25 | 18.07 | *phoU* | cytosolic |
| BCJ09959.1 | pyruvate formate-lyase-activating enzyme | 10 | 6 | 30.1 | 27.25 | *pflA* | cytosolic |
| BCJ11280.1 | endonuclease MutS2 | 10 | 10 | 87.4 | 29.54 | *mutS2* | cytosolic |
| BCJ09915.1 | acetolactate synthase | 10 | 9 | 61.7 | 30.94 | *ilvB* | cytosolic |
| BCJ10234.1 | endopeptidase | 10 | 8 | 71.8 | 30.44 |  | cytosolic |
| BCJ10768.1 | glucose-1-phosphate adenylyltransferase | 10 | 8 | 41.5 | 32.15 | *glgC* | cytosolic |
| BCJ10556.1 | alpha-monoglucosyldiacylglycerol synthase | 10 | 8 | 50.4 | 21.87 |  | cytosolic |
| BCJ10383.1 | chorismate synthase | 10 | 7 | 42.8 | 26.68 | *aroC* | cytosolic |
| BCJ09955.1 | DNA recombination protein RmuC | 10 | 7 | 47.8 | 18.16 |  | cytosolic |
| BCJ11574.1 | DHH family phosphoesterase | 10 | 7 | 70.7 | 19.94 |  | cytosolic |
| BCJ10863.1 | phosphorylcholine transferase LicD | 10 | 7 | 31.9 | 26.78 | *licD1* | cytosolic |
| BCJ10277.1 | UDP-N-acetylmuramyl peptide synthase | 10 | 7 | 49.6 | 24.8 | *murT* | cytosolic |
| BCJ10371.1 | UDP-N-acetylenolpyruvoylglucosamine reductase | 10 | 6 | 18.8 | 31.31 |  | cytosolic |
| BCJ10007.1 | pseudouridine synthase | 10 | 6 | 27.1 | 24.59 | *rluB* | cytosolic |
| BCJ10751.1 | transcriptional regulator | 10 | 5 | 9.6 | 38.97 | *hup* | cytosolic |
| BCJ10999.1 | acetoin utilization protein AcuB | 10 | 5 | 24.1 | 32.88 |  | cytosolic |
| BCJ10559.1 | GTPase Obg | 9 | 8 | 48.4 | 26.15 | *obg* | cytosolic |
| BCJ10352.1 | UDP-N-acetylenolpyruvoylglucosamine reductase | 9 | 8 | 32.2 | 10.61 | *murB* | cytosolic |
| BCJ11170.1 | ribonuclease J | 9 | 8 | 60.8 | 30.06 | *rni* | cytosolic |
| BCJ11440.1 | glutamate--tRNA ligase | 9 | 8 | 55.8 | 24.26 | *gltX* | cytosolic |
| BCJ10992.1 | S-adenosylmethionine synthase | 9 | 6 | 43 | 18.65 | *metK* | cytosolic |
| BCJ10024.1 | chaperone protein DnaJ | 9 | 6 | 40.3 | 22.7 | *dnaJ* | cytosolic |
| BCJ11362.1 | universal stress protein UspA | 9 | 5 | 16.6 | 20.34 |  | cytosolic |
| BCJ10865.1 | carbamoyl-phosphate synthase (glutamine-hydrolyzing) | 9 | 8 | 116.2 | 20.91 | *carB* | cytosolic |
| BCJ09669.1 | aminotransferase | 9 | 8 | 43.2 | 20.65 | *aspC* | cytosolic |
| BCJ09918.1 | L-threonine dehydratase | 9 | 8 | 45.4 | 27.29 | *ilvA* | cytosolic |
| BCJ10984.1 | FMN reductase | 9 | 7 | 22.6 | 20.76 | *azr_1* | cytosolic |
| BCJ11563.1 | DUF4177 domain-containing protein | 9 | 4 | 9.5 | 22.11 |  | cytosolic |
| BCJ09834.1 | 50S ribosomal protein L18 | 8 | 3 | 12.9 | 23.74 | *rplR* | cytosolic |
| BCJ11457.1 | tyrosine--tRNA ligase | 8 | 7 | 47.4 | 27.25 | *tyrS* | cytosolic |
| BCJ10142.1 | ribosomal RNA small subunit methyltransferase E | 8 | 6 | 27.1 | 22.07 |  | cytosolic |
| BCJ11060.1 | thymidylate synthase | 8 | 6 | 32.5 | 19.38 | *thyA* | cytosolic |
| BCJ10988.1 | glycine--tRNA ligase alpha subunit | 8 | 5 | 34.8 | 26.48 | *glyQ* | cytosolic |
| BCJ11258.1 | elongation factor P | 8 | 5 | 20.6 | 22.51 | *efp* | cytosolic |
| BCJ10553.1 | RNA polymerase sigma factor SigA | 8 | 5 | 42 | 23.63 | *rpoD* | cytosolic |
| BCJ11097.1 | putative manganese-dependent inorganic pyrophosphatase | 8 | 5 | 33.3 | 28.88 | *ppaC* | cytosolic |
| BCJ10312.1 | hypothetical protein SMNM65_07440 | 8 | 3 | 7 | 20.45 | *rpsU* | cytosolic |
| BCJ11110.1 | hypothetical protein SMNM65_15420 | 8 | 8 | 89.9 | 23.52 |  | cytosolic |
| BCJ10513.1 | Fe-S cluster assembly protein SufD | 8 | 7 | 46.2 | 16.02 | *sufD* | cytosolic |
| BCJ11267.1 | beta-ketoacyl-ACP reductase | 8 | 7 | 25.6 | 17.16 | *fabG_2* | cytosolic |
| BCJ10314.1 | HPr kinase/phosphorylase | 8 | 6 | 34.8 | 20.93 | *hprK* | cytosolic |
| BCJ10414.1 | hypothetical protein SMNM65_08460 | 8 | 6 | 31.8 | 15.81 |  | cytosolic |
| BCJ10754.1 | riboflavin biosynthesis protein | 8 | 5 | 28.6 | 25.68 | *ribF* | cytosolic |
| BCJ10275.1 | dipeptidase | 8 | 4 | 40.3 | 20.29 | *pepQ* | cytosolic |
| BCJ11491.1 | PadR family transcriptional regulator | 8 | 4 | 12.4 | 23.3 |  | cytosolic |
| BCJ09789.1 | DNA mismatch repair protein HexB | 7 | 7 | 73.5 | 22.28 | *hexB* | cytosolic |
| BCJ09916.1 | acetolactate synthase small subunit | 7 | 6 | 17.7 | 18.6 | *ilvH* | cytosolic |
| BCJ11057.1 | GTPase HflX | 7 | 6 | 46.7 | 16.38 | *hflX* | cytosolic |
| BCJ10308.1 | S-adenosylmethionine:tRNA ribosyltransferase-isomerase | 7 | 5 | 38.5 | 13.46 | *queA* | cytosolic |
| BCJ10965.1 | 23S rRNA (guanosine(2251)-2'-O)-methyltransferase RlmB | 7 | 5 | 24.4 | 18.45 | *trmH* | cytosolic |
| BCJ10812.1 | redox-sensing transcriptional repressor Rex | 7 | 5 | 24.1 | 16.94 | *rex* | cytosolic |
| BCJ11573.1 | 50S ribosomal protein L9 | 7 | 6 | 16.5 | 19.51 | *rplI* | cytosolic |
| BCJ11467.1 | 4-alpha-glucanotransferase | 7 | 7 | 58.1 | 22.83 | *malM* | cytosolic |
| BCJ10040.1 | aminoglycoside phosphotransferase | 7 | 7 | 31.2 | 19.3 | *cotS* | cytosolic |
| BCJ09982.1 | DNA-entry nuclease | 7 | 6 | 29.9 | 22.52 | *endA* | cytosolic |
| BCJ11427.1 | alcohol dehydrogenase | 7 | 6 | 38 | 17.92 | *adh* | cytosolic |
| BCJ11263.1 | acetyl-CoA carboxylase biotin carboxylase subunit | 7 | 6 | 49.5 | 14.47 |  | cytosolic |
| BCJ10147.1 | acetoin reductase | 7 | 6 | 26.5 | 16.29 | *budC* | cytosolic |
| BCJ10586.1 | DNA repair protein RecN | 7 | 6 | 62.7 | 17.45 | *recN* | cytosolic |
| BCJ11532.1 | tRNA-dihydrouridine synthase | 7 | 6 | 35.7 | 18.05 |  | cytosolic |
| BCJ11205.1 | methionyl-tRNA formyltransferase | 7 | 5 | 33.9 | 28.57 | *fmt* | cytosolic |
| BCJ10803.1 | phosphate acetyltransferase | 7 | 5 | 35 | 22.45 | *eutD; pta* | cytosolic |
| BCJ10752.1 | hypothetical protein SMNM65_11840 | 7 | 5 | 30.5 | 15.02 |  | cytosolic |
| BCJ11162.1 | dipeptidase PepV | 7 | 5 | 50.8 | 21.85 |  | cytosolic |
| BCJ11202.1 | protein phosphatase PhpP | 7 | 4 | 27.1 | 15.94 | *phpP* | cytosolic |
| BCJ10769.1 | 1,4-alpha-glucan branching enzyme GlgB | 7 | 4 | 75.5 | 18.33 | *glgB* | cytosolic |
| BCJ10595.1 | galactose-6-phosphate isomerase subunit LacB | 7 | 3 | 18.9 | 32.24 | *lacB* | cytosolic |
| BCJ11105.1 | 30S ribosomal protein S6 | 6 | 5 | 11.1 | 12.95 | *rpsF* | cytosolic |
| BCJ11498.1 | hypothetical protein SMNM65_19300 | 6 | 6 | 399.4 | 7.56 |  | cytosolic |
| BCJ10059.1 | valine--tRNA ligase | 6 | 6 | 101 | 12.53 | *valS* | cytosolic |
| BCJ09758.1 | tRNA uridine 5-carboxymethylaminomethyl modification enzyme MnmG | 6 | 5 | 71.1 | 19.23 | *mnmG* | cytosolic |
| BCJ10702.1 | peptide chain release factor 1 | 6 | 5 | 40.6 | 12.1 | *prfA* | cytosolic |
| BCJ10734.1 | ribosome-recycling factor | 6 | 5 | 20.6 | 11.79 | *frr* | cytosolic |
| BCJ10826.1 | UvrABC system protein B | 6 | 5 | 75.7 | 16.71 | *uvrB* | cytosolic |
| BCJ09867.1 | hypothetical protein SMNM65_02990 | 6 | 5 | 40.7 | 10.04 |  | cytosolic |
| BCJ10713.1 | ferrochelatase | 6 | 4 | 42.5 | 23.26 | *hemH* | cytosolic |
| BCJ11129.1 | 4-hydroxy-tetrahydrodipicolinate reductase | 6 | 4 | 27.9 | 16.4 | *dapB* | cytosolic |
| BCJ09868.1 | hypothetical protein SMNM65_03000 | 6 | 4 | 56.1 | 21.98 |  | cytosolic |
| BCJ10901.1 | 50S ribosomal protein L31 type B | 6 | 4 | 9.4 | 24.34 | *rpmE* | cytosolic |
| BCJ09831.1 | 30S ribosomal protein S14 type Z | 6 | 2 | 7.1 | 5.78 | *rpsN* | cytosolic |
| BCJ10523.1 | 50S ribosomal protein L21 | 6 | 2 | 11.2 | 12.86 | *rplU* | cytosolic |
| BCJ10525.1 | 50S ribosomal protein L27 | 6 | 2 | 10.5 | 19.21 | *rpmA* | cytosolic |
| BCJ11595.1 | inosine-5'-monophosphate dehydrogenase | 6 | 6 | 52.5 | 17.89 | *guaB* | cytosolic |
| BCJ11365.1 | catabolite control protein A | 6 | 6 | 37.1 | 14.48 | *ccpA* | cytosolic |
| BCJ10808.1 | ribose-phosphate pyrophosphokinase 2 | 6 | 5 | 32.2 | 26.78 | *prs2* | cytosolic |
| BCJ09679.1 | DNA-binding response regulator | 6 | 5 | 28.6 | 11.78 |  | cytosolic |
| BCJ11114.1 | aminotransferase | 6 | 5 | 43.5 | 18.45 | *aspB* | cytosolic |
| BCJ10954.1 | aminotransferase V | 6 | 5 | 37.7 | 19.61 |  | cytosolic |
| BCJ11239.1 | hypothetical protein SMNM65_16710 | 6 | 4 | 34.3 | 9 |  | cytosolic |
| BCJ11163.1 | nitroreductase | 6 | 4 | 22.7 | 16.95 |  | cytosolic |
| BCJ11059.1 | DUF3042 domain-containing protein | 6 | 4 | 6 | 14.98 |  | cytosolic |
| BCJ09764.1 | tRNA (adenosine(37)-N6)-threonylcarbamoyltransferase complex dimerization subunit type 1 TsaB | 6 | 4 | 25.4 | 16.15 | *ydiC* | cytosolic |
| BCJ09699.1 | GntR family transcriptional regulator | 6 | 4 | 27.8 | 11.77 |  | cytosolic |
| BCJ10278.1 | pyridine nucleotide-disulfide oxidoreductase | 6 | 4 | 47.1 | 22.44 | *merA* | cytosolic |
| BCJ10089.1 | bifunctional ligase/repressor BirA | 6 | 4 | 35.1 | 18.7 | *birA* | cytosolic |
| BCJ09878.1 | isoprenyl transferase | 5 | 5 | 28.7 | 12.85 | *uppS* | cytosolic |
| BCJ10725.1 | translation initiation factor IF-3 | 5 | 3 | 21.2 | 16.59 | *infC* | cytosolic |
| BCJ10841.1 | chromosome partition protein Smc | 5 | 5 | 134 | 18.39 | *smc* | cytosolic |
| BCJ11038.1 | UDP-N-acetylmuramoylalanine--D-glutamate ligase | 5 | 5 | 48.4 | 13.8 | *murD* | cytosolic |
| BCJ11084.1 | UDP-N-acetylmuramate--L-alanine ligase | 5 | 4 | 49.9 | 15.44 | *murC* | cytosolic |
| BCJ09839.1 | adenylate kinase | 5 | 4 | 23.7 | 8.17 | *adk* | cytosolic |
| BCJ10477.1 | phosphopentomutase | 5 | 3 | 44.1 | 15.46 | *deoB* | cytosolic |
| BCJ10638.1 | ribosome biogenesis GTPase A | 5 | 3 | 32.1 | 16.75 |  | cytosolic |
| BCJ10078.1 | PTS fructose transporter subunit IIB | 5 | 3 | 18.3 | 10.77 | *levE* | cytosolic |
| BCJ10842.1 | ribonuclease 3 | 5 | 3 | 26.1 | 14.59 | *rnc* | cytosolic |
| BCJ11155.1 | 50S ribosomal protein L11 | 5 | 4 | 14.8 | 8.83 | *rplK* | cytosolic |
| BCJ10141.1 | ribosomal protein L11 methyltransferase | 5 | 4 | 34.7 | 11.38 | *prmA* | cytosolic |
| BCJ11428.1 | N-acetylglucosamine-6-phosphate deacetylase | 5 | 5 | 41.8 | 12.2 | *nagA* | cytosolic |
| BCJ11504.1 | lactaldehyde reductase | 5 | 5 | 41.2 | 12.73 | *adh2* | cytosolic |
| BCJ10529.1 | hypothetical protein SMNM65_09610 | 5 | 5 | 63.3 | 13.17 | *pavA* | cytosolic |
| BCJ10555.1 | glycosyl transferase | 5 | 5 | 39 | 11.69 | *cpoA* | cytosolic |
| BCJ10397.1 | homoserine dehydrogenase | 5 | 5 | 46.2 | 10.22 | *thrA* | cytosolic |
| BCJ10489.1 | 16S rRNA methyltransferase | 5 | 5 | 21.4 | 13.41 |  | cytosolic |
| BCJ11301.1 | RNA methyltransferase | 5 | 5 | 43.2 | 12.78 |  | cytosolic |
| BCJ09958.1 | diaminopimelate decarboxylase | 5 | 5 | 46.5 | 15.58 | *lysA* | cytosolic |
| BCJ10708.1 | aspartate-semialdehyde dehydrogenase | 5 | 4 | 38.8 | 15.53 | *asd* | cytosolic |
| BCJ10503.1 | GTP pyrophosphokinase | 5 | 4 | 22.3 | 9.95 |  | cytosolic |
| BCJ10153.1 | thioredoxin | 5 | 4 | 11.4 | 13.79 |  | cytosolic |
| BCJ11472.1 | HTH-type transcriptional regulator MalR | 5 | 4 | 37 | 16.36 | *malR* | cytosolic |
| BCJ09871.1 | transcriptional regulator | 5 | 3 | 17.6 | 17.45 |  | cytosolic |
| BCJ11169.1 | acetylesterase | 5 | 3 | 29.8 | 16.39 |  | cytosolic |
| BCJ10795.1 | thiol reductase thioredoxin | 5 | 3 | 12.8 | 14.71 | *bta* | cytosolic |
| BCJ11142.1 | hypothetical protein SMNM65_15740 | 5 | 3 | 13.7 | 12.21 | *aldR* | cytosolic |
| BCJ11450.1 | UTP--glucose-1-phosphate uridylyltransferase | 5 | 3 | 33.1 | 7.12 | *galU* | cytosolic |
| BCJ10171.1 | phospho-2-dehydro-3-deoxyheptonate aldolase | 5 | 3 | 38.9 | 13.26 |  | cytosolic |
| BCJ10486.1 | 30S ribosomal protein S20 | 4 | 2 | 8.5 | 12.01 | *rpsT* | cytosolic |
| BCJ09828.1 | 50S ribosomal protein L14 | 4 | 3 | 13 | 11.82 | *rplN* | cytosolic |
| BCJ10967.1 | thioredoxin reductase | 4 | 4 | 33.1 | 7.29 | *trxB* | cytosolic |
| BCJ11165.1 | UvrABC system protein C | 4 | 4 | 70.6 | 2.41 | *uvrC* | cytosolic |
| BCJ10022.1 | protein GrpE | 4 | 4 | 20 | 14.51 | *grpE* | cytosolic |
| BCJ10387.1 | 3-phosphoshikimate 1-carboxyvinyltransferase | 4 | 4 | 45.8 | 12.74 | *aroA* | cytosolic |
| BCJ09569.1 | chromosomal replication initiator protein DnaA | 4 | 3 | 51.7 | 13.88 | *dnaA* | cytosolic |
| BCJ10458.1 | DNA gyrase subunit B | 4 | 3 | 72.2 | 10.76 | *gyrB* | cytosolic |
| BCJ11449.1 | glycerol-3-phosphate dehydrogenase [NAD(P)+] | 4 | 3 | 36.7 | 13.97 | *gpsA* | cytosolic |
| BCJ10398.1 | homoserine kinase | 4 | 3 | 31.4 | 12.2 | *thrB* | cytosolic |
| BCJ10815.1 | DNA helicase | 4 | 3 | 86 | 9.3 | *pcrA* | cytosolic |
| BCJ09581.1 | hypothetical protein SMNM65_00130 | 4 | 2 | 14.9 | 10.1 |  | cytosolic |
| BCJ11074.1 | NH(3)-dependent NAD(+) synthetase | 4 | 2 | 30.3 | 8.01 | *nadE* | cytosolic |
| BCJ10425.1 | 30S ribosomal protein S16 | 4 | 2 | 10.2 | 14.65 | *rpsP* | cytosolic |
| BCJ10597.1 | tagatose 1,6-diphosphate aldolase | 4 | 4 | 36.3 | 6.01 | *lacD* | cytosolic |
| BCJ11445.1 | DNA mismatch repair protein HexA | 4 | 4 | 94.8 | 9.05 | *hexA* | cytosolic |
| BCJ10846.1 | translation factor Sua5 | 4 | 4 | 28.8 | 10.56 |  | cytosolic |
| BCJ11132.1 | phosphoglucosamine mutase | 4 | 4 | 48.1 | 14.56 | *glmM* | cytosolic |
| BCJ10263.1 | FAD-dependent oxidoreductase | 4 | 4 | 40.1 | 10.98 |  | cytosolic |
| BCJ10860.1 | ribulose-5-phosphate reductase | 4 | 4 | 38.8 | 8.75 | *tarJ* | cytosolic |
| BCJ11080.1 | transcription elongation factor GreA | 4 | 4 | 17.5 | 10.47 | *greA* | cytosolic |
| BCJ11241.1 | hypothetical protein SMNM65_16730 | 4 | 4 | 79.8 | 6.91 |  | cytosolic |
| BCJ11368.1 | GTP cyclohydrolase 1 | 4 | 4 | 21 | 6.67 | *folE* | cytosolic |
| BCJ10083.1 | hypothetical protein SMNM65_05150 | 4 | 4 | 29.7 | 11.76 | *queH* | cytosolic |
| BCJ10016.1 | type I glutamate--ammonia ligase | 4 | 4 | 50.3 | 7.96 | *glnA* | cytosolic |
| BCJ10476.1 | ribose-5-phosphate isomerase A | 4 | 4 | 24.9 | 11.55 | *rpiA* | cytosolic |
| BCJ11197.1 | hydroxymethylglutaryl-CoA synthase | 4 | 3 | 43.5 | 6.03 |  | cytosolic |
| BCJ10181.1 | hypothetical protein SMNM65_06130 | 4 | 3 | 58.7 | 11.07 |  | cytosolic |
| BCJ11220.1 | haloacid dehalogenase | 4 | 3 | 20.2 | 6.49 |  | cytosolic |
| BCJ10189.1 | N-acetylneuraminate lyase | 4 | 3 | 33.4 | 8.29 | *dapA* | cytosolic |
| BCJ11579.1 | cysteine synthase | 4 | 3 | 32 | 12.11 | *cysK* | cytosolic |
| BCJ11141.1 | nucleotide-binding protein | 4 | 3 | 33.9 | 10.93 |  | cytosolic |
| BCJ11131.1 | transposase | 4 | 3 | 14.4 | 11.97 |  | cytosolic |
| BCJ09804.1 | UPF0297 protein | 4 | 3 | 10.2 | 8.19 |  | cytosolic |
| BCJ10413.1 | hypothetical protein SMNM65_08450 | 4 | 2 | 16.9 | 15.67 |  | cytosolic |
| BCJ10613.1 | ribonucleoside-diphosphate reductase subunit beta | 3 | 3 | 36.9 | 8.93 | *nrdB* | cytosolic |
| BCJ11529.1 | glycerol kinase | 3 | 3 | 55.8 | 8.69 | *glpK* | cytosolic |
| BCJ10537.1 | ribonuclease R | 3 | 3 | 89.3 | 7 | *rnr* | cytosolic |
| BCJ10760.1 | UDP-N-acetylglucosamine 1-carboxyvinyltransferase 2 | 3 | 3 | 44.9 | 6.73 | *murA2* | cytosolic |
| BCJ11572.1 | replicative DNA helicase | 3 | 3 | 50.2 | 6.97 | *dnaB* | cytosolic |
| BCJ11447.1 | arginine--tRNA ligase | 3 | 3 | 63.3 | 7.39 | *argS* | cytosolic |
| BCJ09952.1 | ribosomal RNA small subunit methyltransferase A | 3 | 3 | 32.2 | 8.34 | *rsmA* | cytosolic |
| BCJ11062.1 | glucokinase | 3 | 2 | 33.5 | 12.4 | *glk_2* | cytosolic |
| BCJ10428.1 | tRNA (guanine-N(1)-)-methyltransferase | 3 | 2 | 27.6 | 8.28 | *trmD* | cytosolic |
| BCJ09905.1 | putative transcriptional regulatory protein | 3 | 2 | 25.8 | 8.21 |  | cytosolic |
| BCJ10853.1 | copper homeostasis protein | 3 | 2 | 23 | 8.86 | *cutC* | cytosolic |
| BCJ09575.1 | transcription-repair-coupling factor | 3 | 2 | 122.5 | 5.02 | *mfd* | cytosolic |
| BCJ11490.1 | 50S ribosomal protein L33 type 3 | 3 | 2 | 5.9 | 4.25 | *rpmG* | cytosolic |
| BCJ09912.1 | 50S ribosomal protein L28 | 3 | 2 | 6.9 | 6.97 | *rpmB* | cytosolic |
| BCJ10505.1 | branched-chain-amino-acid aminotransferase | 3 | 3 | 37.4 | 7.85 | *ilvE* | cytosolic |
| BCJ11274.1 | aspartokinase | 3 | 3 | 50.1 | 6.06 | *thrA* | cytosolic |
| BCJ11313.1 | ATP-dependent Clp protease ATP-binding subunit | 3 | 3 | 77.5 | 8.6 | *clpL* | cytosolic |
| BCJ09936.1 | transcription antiterminator BglG | 3 | 3 | 32.9 | 4.75 | *licT* | cytosolic |
| BCJ10959.1 | tagatose-6-phosphate kinase | 3 | 3 | 32.7 | 6.85 | *fruB* | cytosolic |
| BCJ11260.1 | N utilization substance protein B | 3 | 3 | 16.2 | 10.06 | *nusB* | cytosolic |
| BCJ11130.1 | DegV domain-containing protein | 3 | 3 | 30.9 | 7.68 |  | cytosolic |
| BCJ10211.1 | YggS family pyridoxal phosphate enzyme | 3 | 3 | 25.6 | 7.66 |  | cytosolic |
| BCJ10087.1 | 23S rRNA (uracil-5-)-methyltransferase RumA | 3 | 3 | 50.7 | 6.03 |  | cytosolic |
| BCJ11517.1 | DeoR family transcriptional regulator | 3 | 3 | 22.9 | 7.85 | *fcsR* | cytosolic |
| BCJ11357.1 | ATPase | 3 | 3 | 46.9 | 8.49 |  | cytosolic |
| BCJ11240.1 | glycosyl transferase family A | 3 | 3 | 35.3 | 6.54 | *cpsJ* | cytosolic |
| BCJ09849.1 | phosphoglycerate mutase | 3 | 3 | 25.6 | 5.75 | *gpmB* | cytosolic |
| BCJ10818.1 | phosphopantothenoylcysteine decarboxylase | 3 | 3 | 16.3 | 6.34 |  | cytosolic |
| BCJ10119.1 | tryptophan synthase beta chain | 3 | 3 | 44.2 | 3.83 | *trpB* | cytosolic |
| BCJ10731.1 | phosphate starvation protein PhoH | 3 | 3 | 37.6 | 9.52 |  | cytosolic |
| BCJ10379.1 | methyltransferase | 3 | 3 | 44.1 | 6.89 |  | cytosolic |
| BCJ11486.1 | dihydroxy-acid dehydratase | 3 | 3 | 59.9 | 10.37 | *ilvD* | cytosolic |
| BCJ10859.1 | choline kinase | 3 | 3 | 30.6 | 7.61 | *licA* | cytosolic |
| BCJ11065.1 | aminodeoxychorismate synthase, component I | 3 | 3 | 65.6 | 5.94 | *pabB* | cytosolic |
| BCJ10164.1 | transcriptional repressor NrdR | 3 | 3 | 18.4 | 10.25 | *nrdR* | cytosolic |
| BCJ10452.1 | protease | 3 | 3 | 35.5 | 6.31 | *yhbU_1* | cytosolic |
| BCJ10571.1 | hypothetical protein SMNM65_10030 | 3 | 3 | 16.3 | 8.88 |  | cytosolic |
| BCJ11437.1 | threonine synthase | 3 | 3 | 53.6 | 7.03 | *thrC* | cytosolic |
| BCJ10456.1 | 4-methyl-5(B-hydroxyethyl)-thiazole monophosphate biosynthesis protein | 3 | 3 | 20 | 9.28 |  | cytosolic |
| BCJ11118.1 | FMN reductase | 3 | 3 | 19.7 | 7.28 |  | cytosolic |
| BCJ11363.1 | alanine aminotransferase | 3 | 2 | 52.2 | 8.87 |  | cytosolic |
| BCJ10392.1 | glycosyl transferase | 3 | 2 | 42.3 | 8.26 |  | cytosolic |
| BCJ11216.1 | hypothetical protein SMNM65_16480 | 3 | 2 | 15 | 8.32 |  | cytosolic |
| BCJ11398.1 | transcription termination/antitermination protein NusG | 3 | 2 | 20.8 | 7.73 | *nusG* | cytosolic |
| BCJ10245.1 | TetR family transcriptional regulator | 3 | 2 | 21.7 | 5.47 |  | cytosolic |
| BCJ10612.1 | DeoR family transcriptional regulator | 3 | 2 | 28.4 | 8.63 | *lacR* | cytosolic |
| BCJ09756.1 | tRNA-specific 2-thiouridylase MnmA | 2 | 2 | 41.5 | 5.02 | *trmU* | cytosolic |
| BCJ10205.1 | D-alanine--D-alanine ligase | 2 | 2 | 38.7 | 2.53 | *ddl* | cytosolic |
| BCJ10098.1 | galactokinase | 2 | 2 | 43.6 | 9.17 | *galK* | cytosolic |
| BCJ11271.1 | 3-oxoacyl-[acyl-carrier-protein] synthase 3 | 2 | 2 | 34.9 | 9.86 | *fabH* | cytosolic |
| BCJ11528.1 | glycerol-3-phosphate dehydrogenase | 2 | 2 | 65 | 2.77 | *glpD* | cytosolic |
| BCJ10538.1 | SsrA-binding protein | 2 | 2 | 17.4 | 6.43 | *smpB* | cytosolic |
| BCJ10855.1 | DNA topoisomerase 1 | 2 | 2 | 76.7 | 0 | *topA* | cytosolic |
| BCJ11173.1 | glutamine ABC transporter substrate-binding protein | 2 | 2 | 29.6 | 3.15 | *glnH_2* | cytosolic |
| BCJ11214.1 | ribosomal silencing factor RsfS | 2 | 2 | 12.9 | 5.54 | *rsfS* | cytosolic |
| BCJ11605.1 | ribosomal RNA large subunit methyltransferase H | 2 | 2 | 17.9 | 8.07 | *rlmH* | cytosolic |
| BCJ10081.1 | 10 kDa chaperonin | 2 | 2 | 9.9 | 5.17 | *groS* | cytosolic |
| BCJ09975.1 | RNA methyltransferase | 2 | 2 | 23 | 0 |  | cytosolic |
| BCJ10043.1 | transcription termination/antitermination protein NusA | 2 | 2 | 42.7 | 6.68 | *nusA* | cytosolic |
| BCJ10510.1 | DNA polymerase III subunit gamma/tau | 2 | 2 | 62.4 | 8.83 | *dnaX* | cytosolic |
| BCJ11418.1 | DNA methyltransferase | 2 | 2 | 35.5 | 8.14 |  | cytosolic |
| BCJ10380.1 | 3-dehydroquinate dehydratase | 2 | 2 | 25.7 | 3.18 | *aroD* | cytosolic |
| BCJ10202.1 | RpiR family transcriptional regulator | 2 | 2 | 32.7 | 7.45 |  | cytosolic |
| BCJ11089.1 | cystathionine gamma-synthase | 2 | 2 | 40.1 | 5.27 | *metB* | cytosolic |
| BCJ10868.1 | bifunctional protein PyrR | 2 | 2 | 19.5 | 3.53 | *pyrR* | cytosolic |
| BCJ09859.1 | DeoR family transcriptional regulator | 2 | 2 | 27.9 | 7.12 | *deoR* | cytosolic |
| BCJ10563.1 | hypothetical protein SMNM65_09950 | 2 | 2 | 51.4 | 6.34 |  | cytosolic |
| BCJ10009.1 | glutamyl aminopeptidase | 2 | 2 | 29.2 | 7.1 | *pepA* | cytosolic |
| BCJ10183.1 | putative N-acetylmannosamine-6-phosphate 2-epimerase 2 | 2 | 2 | 25.4 | 5.51 | *nanE2* | cytosolic |
| BCJ10520.1 | RNA helicase | 2 | 2 | 41.2 | 7.03 | *DEAD/H* | cytosolic |
| BCJ09715.1 | TetR family transcriptional regulator | 2 | 2 | 20.9 | 3.56 |  | cytosolic |
| BCJ10130.1 | hypothetical protein SMNM65_05620 | 2 | 2 | 11.2 | 6.32 |  | cytosolic |
| BCJ11016.1 | 6-phospho-beta-glucosidase | 2 | 2 | 55.1 | 0 | *arb* | cytosolic |
| BCJ10960.1 | DeoR family transcriptional regulator | 2 | 2 | 27.2 | 5.68 | *fruR* | cytosolic |
| BCJ09812.1 | anaerobic ribonucleoside triphosphate reductase | 2 | 2 | 83.8 | 2.19 | *nrdD* | cytosolic |
| BCJ10426.1 | UPF0109 protein | 2 | 2 | 8.9 | 6.28 | *khpA* | cytosolic |
| BCJ10420.1 | transcriptional regulator | 2 | 2 | 21 | 4.57 |  | cytosolic |
| BCJ11071.1 | hypothetical protein SMNM65_15030 | 2 | 2 | 20.7 | 5.08 |  | cytosolic |
| BCJ10694.1 | 23S rRNA (uracil-5-)-methyltransferase RumA | 2 | 2 | 50.9 | 3.62 |  | cytosolic |
| BCJ11034.1 | orotate phosphoribosyltransferase | 2 | 2 | 22.8 | 5.41 | *pyrE* | cytosolic |
| BCJ10915.1 | 5'-methylthioadenosine/S-adenosylhomocysteine nucleosidase | 2 | 2 | 24.6 | 7.24 | *pfs* | cytosolic |
| BCJ11185.1 | 5-methyltetrahydropteroyltriglutamate--homocysteine methyltransferase | 2 | 2 | 84.6 | 1.69 | *metE* | cytosolic |
| BCJ11164.1 | ser/threonine protein phosphatase | 2 | 2 | 32.3 | 5.99 |  | cytosolic |
| BCJ09956.1 | 3'-5' exoribonuclease YhaM | 2 | 2 | 35.8 | 5.6 | *cbf1* | cytosolic |
| BCJ09705.1 | tagatose-6-phosphate ketose isomerase | 2 | 2 | 43 | 5.36 | *agaS* | cytosolic |
| BCJ10340.1 | 23S rRNA methyltransferase | 2 | 2 | 48.1 | 3.68 |  | cytosolic |
| BCJ11268.1 | malonyl CoA-acyl carrier protein transacylase | 2 | 2 | 33.1 | 6.88 | *fabD* | cytosolic |
| BCJ10543.1 | oligoendopeptidase F | 2 | 2 | 69.8 | 6.34 | *pepB* | cytosolic |
| BCJ11009.1 | TetR family transcriptional regulator | 2 | 2 | 21.8 | 6.26 | *mtrR* | cytosolic |
| BCJ09864.1 | glycyl radical enzyme | 2 | 2 | 91.3 | 5.52 | *pflF* | cytosolic |
| BCJ10143.1 | oligoendopeptidase F | 2 | 2 | 68.6 | 6.63 |  | cytosolic |
| BCJ11381.1 | aminopeptidase | 2 | 2 | 45 | 3.1 | *pepS* | cytosolic |
| BCJ10884.1 | phosphohydrolase | 2 | 2 | 40.6 | 3.9 |  | cytosolic |
| BCJ11064.1 | acyl-CoA thioester hydrolase | 2 | 2 | 22.8 | 5.3 |  | cytosolic |
| BCJ09775.1 | lipoprotein | 41 | 14 | 31.1 | 133.32 |  | lipoproteins |
| BCJ09650.1 | ribose-phosphate pyrophosphokinase | 14 | 12 | 35.4 | 42.75 | *prs* | lipoproteins |
| BCJ10315.1 | prolipoprotein diacylglyceryl transferase | 7 | 3 | 30.3 | 18.47 | *lgt* | lipoproteins |
| BCJ10545.1 | foldase protein PrsA | 128 | 23 | 28 | 429.6 | *prsA_2* | lipoptorein |
| BCJ09885.1 | pullulanase | 2 | 2 | 153.7 | 4.46 | *pulA_1* | lipoptorein |
| BCJ10480.1 | hypothetical protein SMNM65_09120 | 224 | 61 | 78.9 | 709.34 | *mlc* | secreted protein |
| BCJ10761.1 | enolase | 117 | 27 | 47.1 | 298.57 | *eno* | secreted protein |
| BCJ10528.1 | endo-beta-N-acetylglucosaminidase | 41 | 24 | 65.5 | 134.62 | *lytB* | secreted protein |
| BCJ11583.1 | cell shape-determining protein MreC | 25 | 13 | 29.8 | 90.15 | *mreC* | secreted protein |
| BCJ09886.1 | hPAF protein | 17 | 12 | 73.6 | 48.17 | *samhpaf* | secreted protein |
| BCJ09904.1 | mitilysin | 6 | 5 | 52.9 | 14.54 | *mly* | secreted protein |
| BCJ10493.1 | BMP family ABC transporter substrate-binding protein | 551 | 28 | 36.7 | 1776.05 |  | transmembrane |
| BCJ11468.1 | sugar ABC transporter substrate-binding protein | 344 | 32 | 45.3 | 1141.53 | *malX* | transmembrane |
| BCJ10231.1 | metal ABC transporter substrate-binding protein | 331 | 26 | 34.5 | 1070.7 | *psaA* | transmembrane |
| BCJ11004.1 | branched-chain amino acid ABC transporter substrate-binding protein | 255 | 27 | 40.3 | 872.25 | *livJ* | transmembrane |
| BCJ10434.1 | transporter | 145 | 25 | 41.7 | 486.3 | *acrA* | transmembrane |
| BCJ11201.1 | serine/threonine-protein kinase StkP | 141 | 41 | 72.4 | 441.8 | *stkP* | transmembrane |
| BCJ11305.1 | penicillin-binding protein 1A | 117 | 30 | 72.5 | 371.33 | *pbpA* | transmembrane |
| BCJ10184.1 | sugar ABC transporter substrate-binding protein | 116 | 26 | 48.2 | 376.83 |  | transmembrane |
| BCJ11081.1 | aminodeoxychorismate lyase | 109 | 29 | 54.2 | 329.45 | *mltG* | transmembrane |
| BCJ10170.1 | protein translocase subunit SecA | 90 | 44 | 94.9 | 250.08 | *secA_1* | transmembrane |
| BCJ10931.1 | ATP synthase subunit beta | 88 | 27 | 50.9 | 300.84 | *atpD* | transmembrane |
| BCJ10958.1 | PTS fructose transporter subunit IIC | 88 | 18 | 66.9 | 234.08 | *fruA* | transmembrane |
| BCJ11494.1 | membrane protein | 86 | 19 | 40.5 | 301.74 |  | transmembrane |
| BCJ09723.1 | 30S ribosomal protein S4 | 83 | 16 | 23 | 222.93 | *rpsD* | transmembrane |
| BCJ09774.1 | amino acid ABC transporter substrate-binding protein | 77 | 14 | 30.5 | 211.84 | *tcyA* | transmembrane |
| BCJ09582.1 | ATP-dependent zinc metalloprotease FtsH | 72 | 31 | 71.4 | 208 | *ftsH* | transmembrane |
| BCJ11456.1 | penicillin-binding protein 1B | 65 | 35 | 89.3 | 178.1 | *pbp1b* | transmembrane |
| BCJ10828.1 | amino acid ABC transporter permease | 65 | 26 | 78.3 | 198.07 | *glnP_2* | transmembrane |
| BCJ10991.1 | peptidoglycan GlcNAc deacetylase | 65 | 25 | 50.9 | 200.92 | *pgdA* | transmembrane |
| BCJ11458.1 | ATPase P | 64 | 27 | 73.4 | 199.38 | *ctpC* | transmembrane |
| BCJ11125.1 | ATPase | 63 | 27 | 96.9 | 213.52 | *pacL* | transmembrane |
| BCJ09937.1 | PTS beta-glucoside transporter subunit EIIBCA | 63 | 20 | 65.7 | 209.66 |  | transmembrane |
| BCJ11306.1 | peptide ABC transporter ATP-binding protein | 61 | 33 | 72.7 | 194.11 | *aliA* | transmembrane |
| BCJ09984.1 | peptide ABC transporter substrate-binding protein | 61 | 32 | 72.5 | 180.2 | *amiA* | transmembrane |
| BCJ11411.1 | preprotein translocase subunit YajC | 55 | 4 | 11.1 | 185.62 | *yajC* | transmembrane |
| BCJ11375.1 | PTS mannose transporter subunit EIIAB | 52 | 15 | 35.4 | 123.33 | *manX* | transmembrane |
| BCJ10549.1 | ABC transporter substrate-binding protein | 52 | 16 | 34.6 | 170.23 |  | transmembrane |
| BCJ11091.1 | oligopeptide-binding protein AliB | 51 | 27 | 72.6 | 150.68 | *aliB_1* | transmembrane |
| BCJ09921.1 | glutamine ABC transporter substrate-binding protein | 46 | 15 | 57.4 | 165.58 | *glnH_1* | transmembrane |
| BCJ09945.1 | peptide-binding protein | 46 | 26 | 72.7 | 138.68 |  | transmembrane |
| BCJ11503.1 | protease | 46 | 21 | 33.7 | 127.88 |  | transmembrane |
| BCJ11177.1 | ABC transporter ATP-binding protein | 46 | 19 | 41.8 | 131.9 | *sugC* | transmembrane |
| BCJ10506.1 | peptide-binding protein | 45 | 24 | 72.7 | 146.96 |  | transmembrane |
| BCJ10395.1 | hypothetical protein SMNM65_08270 | 45 | 16 | 38.7 | 125.91 |  | transmembrane |
| BCJ10203.1 | penicillin-binding protein 2B | 44 | 27 | 73.7 | 128.38 | *pbp2B* | transmembrane |
| BCJ10933.1 | ATP synthase subunit alpha | 43 | 18 | 54.6 | 110.47 | *atpA* | transmembrane |
| BCJ10494.1 | ABC transporter ATP-binding protein | 43 | 23 | 55.2 | 107.64 | *mglA* | transmembrane |
| BCJ10209.1 | cell division protein FtsA | 42 | 17 | 49.5 | 129.77 | *ftsA* | transmembrane |
| BCJ11493.1 | hypothetical protein SMNM65_19250 | 41 | 16 | 33.9 | 107.47 |  | transmembrane |
| BCJ10481.1 | hemolysin | 40 | 12 | 19.4 | 114.47 |  | transmembrane |
| BCJ11284.1 | signal peptidase I | 40 | 9 | 19 | 84.43 | *lepB; tig* | transmembrane |
| BCJ10375.1 | ABC transporter ATP-binding protein | 40 | 11 | 27.6 | 116.61 |  | transmembrane |
| BCJ09742.1 | short-chain dehydrogenase | 39 | 19 | 69.2 | 100.82 |  | transmembrane |
| BCJ11300.1 | Mid-cell-anchored protein Z | 39 | 16 | 51.9 | 129.66 | *mapZ* | transmembrane |
| BCJ11518.1 | zinc-binding protein | 39 | 20 | 56.3 | 109.92 | *adcA* | transmembrane |
| BCJ11309.1 | peptide-binding protein | 38 | 20 | 72.6 | 105.78 |  | transmembrane |
| BCJ09989.1 | ABC transporter ATP-binding protein | 38 | 15 | 34.8 | 89.11 | *amiF* | transmembrane |
| BCJ10162.1 | ABC transporter ATP-binding protein | 38 | 14 | 25.6 | 106.69 |  | transmembrane |
| BCJ09784.1 | hypothetical protein SMNM65_02160 | 37 | 2 | 19.7 | 119.12 |  | transmembrane |
| BCJ10256.1 | metal ABC transporter substrate-binding protein | 37 | 21 | 36.8 | 111.25 | *fecB* | transmembrane |
| BCJ09985.1 | oligopeptide transport system permease protein AmiC | 36 | 13 | 55.5 | 101.61 | *amiC* | transmembrane |
| BCJ11308.1 | peptide-binding protein | 36 | 18 | 72.5 | 102.12 | *aliB_2* | transmembrane |
| BCJ10348.1 | amino acid ABC transporter substrate-binding protein | 36 | 16 | 26.9 | 117.64 |  | transmembrane |
| BCJ11327.1 | ABC transporter substrate-binding protein | 35 | 22 | 58.6 | 97.12 |  | transmembrane |
| BCJ09811.1 | damage-inducible protein CinA | 34 | 10 | 57.8 | 109.94 |  | transmembrane |
| BCJ11459.1 | hypothetical protein SMNM65_18910 | 34 | 8 | 10 | 79.93 |  | transmembrane |
| BCJ11377.1 | PTS mannose transporter subunit IID | 34 | 13 | 33.8 | 104.21 | *manN* | transmembrane |
| BCJ11606.1 | serine protease | 33 | 17 | 41.6 | 113.9 | *htrA* | transmembrane |
| BCJ11522.1 | D-alanyl-lipoteichoic acid biosynthesis protein DltD | 33 | 17 | 48.9 | 91.25 | *dltD* | transmembrane |
| BCJ11567.1 | ABC transporter substrate-binding protein | 33 | 18 | 37.8 | 96.01 |  | transmembrane |
| BCJ10997.1 | cell division ATP-binding protein FtsE | 32 | 15 | 25.8 | 85.01 | *ftsE* | transmembrane |
| BCJ10608.1 | PTS lactose transporter subunit IIBC | 30 | 13 | 61 | 85 | *lacE* | transmembrane |
| BCJ11315.1 | penicillin-binding protein 2X | 30 | 22 | 81.8 | 85.59 | *pbp2x* | transmembrane |
| BCJ10995.1 | PTS glucose transporter subunit IIABC | 29 | 11 | 77.8 | 87.86 | *ptsG* | transmembrane |
| BCJ10460.1 | hypothetical protein SMNM65_08920 | 29 | 15 | 43.6 | 87.24 |  | transmembrane |
| BCJ10390.1 | LytR family transcriptional regulator | 28 | 13 | 47.6 | 86.66 |  | transmembrane |
| BCJ10996.1 | cell division protein FtsX | 28 | 11 | 34.2 | 78.92 | *ftsX* | transmembrane |
| BCJ09988.1 | ABC transporter ATP-binding protein | 28 | 13 | 38.6 | 84.06 | *amiE* | transmembrane |
| BCJ11492.1 | membrane protein | 27 | 7 | 21.2 | 86.08 |  | transmembrane |
| BCJ10532.1 | GTPase Era | 26 | 14 | 34 | 77.31 | *era* | transmembrane |
| BCJ10233.1 | manganese ABC transporter ATP-binding protein | 26 | 12 | 26.9 | 75.98 |  | transmembrane |
| BCJ10012.1 | sodium-dependent phosphate transporter | 25 | 11 | 59.4 | 82.54 |  | transmembrane |
| BCJ09572.1 | ribosome-binding ATPase YchF | 25 | 13 | 41.1 | 84.37 | *ychF* | transmembrane |
| BCJ10435.1 | ABC transporter ATP-binding protein | 24 | 9 | 25.7 | 76.59 | *salX* | transmembrane |
| BCJ11172.1 | arginine ABC transporter ATP-binding protein | 23 | 10 | 28.2 | 47.05 |  | transmembrane |
| BCJ11123.1 | hypothetical protein SMNM65_15550 | 22 | 16 | 59.4 | 64.03 |  | transmembrane |
| BCJ10378.1 | alkaline phosphatase | 22 | 15 | 81.5 | 65.07 |  | transmembrane |
| BCJ10436.1 | membrane protein | 22 | 9 | 45.1 | 57.69 |  | transmembrane |
| BCJ10932.1 | ATP synthase gamma chain | 22 | 9 | 32.3 | 67.21 | *atpG* | transmembrane |
| BCJ11120.1 | hypothetical protein SMNM65_15520 | 22 | 21 | 361.4 | 63.45 |  | transmembrane |
| BCJ11402.1 | hypothetical protein SMNM65_18340 | 21 | 11 | 22.6 | 42.01 |  | transmembrane |
| BCJ10949.1 | aquaporin | 21 | 4 | 30.7 | 77.11 | *glpF_1* | transmembrane |
| BCJ11036.1 | cell division protein DivIB | 21 | 12 | 47.3 | 67.61 | *div1B* | transmembrane |
| BCJ11000.1 | ABC transporter ATP-binding protein | 21 | 9 | 25.7 | 64.07 | *livF* | transmembrane |
| BCJ10286.1 | hypothetical protein SMNM65_07180 | 20 | 17 | 189.5 | 55.99 |  | transmembrane |
| BCJ09920.1 | peptide ABC transporter ATP-binding protein | 20 | 11 | 27.9 | 56.72 |  | transmembrane |
| BCJ10450.1 | two-component sensor histidine kinase | 19 | 11 | 49.7 | 64.81 | *ciaH* | transmembrane |
| BCJ10267.1 | hypothetical protein SMNM65_06990 | 19 | 6 | 17.2 | 69.79 |  | transmembrane |
| BCJ09838.1 | protein translocase subunit SecY | 19 | 5 | 47.4 | 28.88 | *secY* | transmembrane |
| BCJ09777.1 | methionine import ATP-binding protein MetN | 19 | 12 | 38.7 | 50.01 | *metN* | transmembrane |
| BCJ10877.1 | membrane protein | 18 | 10 | 20.6 | 49.94 | *lemA* | transmembrane |
| BCJ11023.1 | copper-translocating P-type ATPase | 18 | 16 | 80.4 | 37.84 | *ctpA* | transmembrane |
| BCJ11314.1 | phospho-N-acetylmuramoyl-pentapeptide-transferase | 18 | 5 | 35.9 | 34.58 | *mraY* | transmembrane |
| BCJ10969.1 | amino acid ABC transporter ATP-binding protein | 18 | 11 | 27.4 | 44.54 | *tcyN* | transmembrane |
| BCJ09880.1 | putative zinc metalloprotease | 17 | 13 | 45.8 | 44.92 |  | transmembrane |
| BCJ10258.1 | 1-acyl-sn-glycerol-3-phosphate acyltransferase | 17 | 10 | 28.8 | 35.33 |  | transmembrane |
| BCJ11044.1 | NADH dehydrogenase | 17 | 8 | 14.7 | 46.33 | *moeZ* | transmembrane |
| BCJ10829.1 | peptide ABC transporter ATP-binding protein | 17 | 9 | 27.3 | 54.8 | *glnQ_2* | transmembrane |
| BCJ11599.1 | hypothetical protein SMNM65_20310 | 16 | 7 | 51.3 | 30.11 |  | transmembrane |
| BCJ10291.1 | ABC transporter permease | 16 | 12 | 49.9 | 49.97 | *vexp3* | transmembrane |
| BCJ09853.1 | iron ABC transporter substrate-binding protein | 16 | 8 | 37.8 | 45.57 |  | transmembrane |
| BCJ11487.1 | membrane protein | 15 | 12 | 36.3 | 42.31 |  | transmembrane |
| BCJ11037.1 | UDP-N-acetylglucosamine--N-acetylmuramyl-(pentapeptide) pyrophosphoryl-undecaprenol N-acetylglucosamine transferase | 15 | 11 | 39.3 | 45.4 | *murG* | transmembrane |
| BCJ10936.1 | ATP synthase subunit b | 15 | 9 | 17.9 | 36.07 | *atpF* | transmembrane |
| BCJ10259.1 | ATPase | 14 | 10 | 86 | 38.15 |  | transmembrane |
| BCJ09943.1 | sodium ABC transporter permease | 14 | 8 | 44 | 37.48 | *natB* | transmembrane |
| BCJ10376.1 | hypothetical protein SMNM65_08080 | 14 | 8 | 61.8 | 38.6 |  | transmembrane |
| BCJ09748.1 | hypothetical protein SMNM65_01800 | 14 | 8 | 23.1 | 39.75 |  | transmembrane |
| BCJ11209.1 | ribonuclease Y | 14 | 11 | 60.3 | 37.92 | *rny* | transmembrane |
| BCJ10588.1 | elongation factor 4 | 14 | 10 | 67.6 | 39.14 | *lepA* | transmembrane |
| BCJ09869.1 | ABC transporter ATP-binding protein | 14 | 10 | 32.6 | 35.6 |  | transmembrane |
| BCJ10467.1 | peptide ABC transporter ATP-binding protein | 14 | 10 | 27 | 32.48 |  | transmembrane |
| BCJ09942.1 | sodium ABC transporter ATP-binding protein | 14 | 8 | 33.8 | 22.54 | *ecsA* | transmembrane |
| BCJ10027.1 | ABC transporter ATP-binding protein | 14 | 7 | 27.1 | 31.29 |  | transmembrane |
| BCJ11115.1 | peptidase | 13 | 8 | 18.3 | 38.22 |  | transmembrane |
| BCJ11443.1 | multidrug ABC transporter permease | 13 | 7 | 65.6 | 44.68 |  | transmembrane |
| BCJ09973.1 | membrane protein insertase YidC 1 | 13 | 6 | 33.8 | 49.32 | *yidC1* | transmembrane |
| BCJ10094.1 | ABC transporter permease | 13 | 11 | 55.5 | 34.39 |  | transmembrane |
| BCJ10353.1 | spermidine/putrescine import ATP-binding protein PotA | 13 | 11 | 44.1 | 35.01 | *potA* | transmembrane |
| BCJ10957.1 | cell division protein FtsK | 13 | 10 | 81.5 | 28.8 | *ftsK* | transmembrane |
| BCJ10459.1 | hypothetical protein SMNM65_08910 | 13 | 6 | 20.5 | 52.53 |  | transmembrane |
| BCJ11406.1 | hypothetical protein SMNM65_18380 | 13 | 8 | 25.3 | 20.75 |  | transmembrane |
| BCJ09907.1 | ABC transporter | 12 | 11 | 65.3 | 43.36 |  | transmembrane |
| BCJ10393.1 | galactosyl transferase | 12 | 9 | 38.2 | 32.62 |  | transmembrane |
| BCJ11221.1 | magnesium transporter | 12 | 6 | 34.8 | 34 | *corA_1* | transmembrane |
| BCJ09999.1 | UPF0154 protein | 12 | 6 | 9.1 | 27.67 |  | transmembrane |
| BCJ10592.1 | PTS galactitol transporter subunit IIC | 12 | 4 | 53.1 | 35.29 |  | transmembrane |
| BCJ11405.1 | permease | 11 | 7 | 46.1 | 28.38 |  | transmembrane |
| BCJ09702.1 | PTS fructose transporter subunit IIC | 11 | 5 | 32.1 | 22.92 |  | transmembrane |
| BCJ09634.1 | hypothetical protein SMNM65_00660 | 10 | 8 | 17.6 | 31.6 |  | transmembrane |
| BCJ09911.1 | hypothetical protein SMNM65_03430 | 10 | 7 | 24.7 | 29.04 |  | transmembrane |
| BCJ10785.1 | hypothetical protein SMNM65_12170 | 10 | 4 | 86.5 | 21.17 |  | transmembrane |
| BCJ10108.1 | DUF421 domain-containing protein | 10 | 6 | 23.3 | 29.9 |  | transmembrane |
| BCJ10495.1 | sugar ABC transporter permease | 10 | 4 | 37.4 | 31.79 |  | transmembrane |
| BCJ11597.1 | ABC-F family ATPase | 10 | 7 | 60.8 | 37.44 |  | transmembrane |
| BCJ10551.1 | ABC transporter ATP-binding protein | 10 | 7 | 28.3 | 27.09 |  | transmembrane |
| BCJ10290.1 | ABC transporter ATP-binding protein | 10 | 5 | 23.9 | 27.5 | *vex2* | transmembrane |
| BCJ11019.1 | hypothetical protein SMNM65_14510 | 9 | 7 | 42.7 | 27.34 |  | transmembrane |
| BCJ09801.1 | magnesium-translocating P-type ATPase | 9 | 8 | 98.2 | 26.88 | *mgtA* | transmembrane |
| BCJ09986.1 | hypothetical protein SMNM65_04180 | 9 | 4 | 15.7 | 25.73 |  | transmembrane |
| BCJ10504.1 | DNA topoisomerase 4 subunit A | 9 | 8 | 92.3 | 19.4 | *parC* | transmembrane |
| BCJ11478.1 | ABC transporter ATP-binding protein | 9 | 7 | 30.5 | 26.68 |  | transmembrane |
| BCJ11520.1 | zinc ABC transporter ATP-binding protein | 9 | 5 | 26.6 | 24.59 | *adcC* | transmembrane |
| BCJ11018.1 | peptide ABC transporter ATP-binding protein | 9 | 5 | 24.2 | 20.23 |  | transmembrane |
| BCJ09626.1 | phage tail tape measure protein | 8 | 8 | 100.2 | 18.89 |  | transmembrane |
| BCJ09922.1 | hypothetical protein SMNM65_03540 | 8 | 6 | 67 | 20.48 |  | transmembrane |
| BCJ10577.1 | hypothetical protein SMNM65_10090 | 8 | 6 | 30.8 | 21.84 |  | transmembrane |
| BCJ09901.1 | hypothetical protein SMNM65_03330 | 8 | 5 | 17.5 | 20.87 |  | transmembrane |
| BCJ10934.1 | hypothetical protein SMNM65_13660 | 8 | 3 | 9.4 | 21.06 |  | transmembrane |
| BCJ10930.1 | ATP synthase epsilon chain | 8 | 5 | 15.6 | 21.55 | *atpC* | transmembrane |
| BCJ11003.1 | branched-chain amino acid ABC transporter permease | 8 | 2 | 30.8 | 17.6 | *livH* | transmembrane |
| BCJ11228.1 | glycosyltransferase stabilizing protein Gtf2 | 8 | 6 | 51.1 | 23.42 | *gtf2* | transmembrane |
| BCJ10666.1 | ABC transporter ATP-binding protein | 8 | 8 | 28.1 | 24.11 | *macB* | transmembrane |
| BCJ09751.1 | amino acid ABC transporter permease | 7 | 6 | 79.2 | 20.39 |  | transmembrane |
| BCJ10664.1 | hypothetical protein SMNM65_10960 | 7 | 4 | 24.7 | 15.94 |  | transmembrane |
| BCJ10955.1 | hypothetical protein SMNM65_13870 | 7 | 3 | 11 | 16.02 |  | transmembrane |
| BCJ09796.1 | hypothetical protein SMNM65_02280 | 7 | 3 | 25.4 | 18.34 |  | transmembrane |
| BCJ11586.1 | energy-coupling factor transporter ATP-binding protein EcfA | 7 | 6 | 31.1 | 21.56 | *ecfA* | transmembrane |
| BCJ09703.1 | PTS fructose transporter subunit IID | 7 | 3 | 30.3 | 9.81 |  | transmembrane |
| BCJ11229.1 | glycosyltransferase Gtf1 | 7 | 5 | 57.7 | 16.87 | *gtf1* | transmembrane |
| BCJ10831.1 | signal recognition particle receptor FtsY | 7 | 5 | 43.8 | 18.26 | *ftsY* | transmembrane |
| BCJ10124.1 | sugar ABC transporter substrate-binding protein | 7 | 7 | 48.4 | 19.31 |  | transmembrane |
| BCJ10224.1 | hypothetical protein SMNM65_06560 | 6 | 6 | 45.1 | 18.01 |  | transmembrane |
| BCJ10940.1 | hypothetical protein SMNM65_13720 | 6 | 6 | 162.5 | 17.47 |  | transmembrane |
| BCJ10512.1 | ABC transporter ATP-binding protein | 6 | 5 | 28.4 | 16.69 |  | transmembrane |
| BCJ11126.1 | cation transporter | 6 | 5 | 44.5 | 12.61 |  | transmembrane |
| BCJ10928.1 | AI-2E family transporter | 6 | 4 | 43.3 | 14.24 |  | transmembrane |
| BCJ11234.1 | hypothetical protein SMNM65_16660 | 6 | 4 | 30.9 | 13.71 |  | transmembrane |
| BCJ09809.1 | cardiolipin synthase | 6 | 6 | 58.7 | 16.5 | *cls* | transmembrane |
| BCJ11193.1 | PTS beta-glucoside transporter subunit EIIBCA | 6 | 6 | 65.6 | 10.71 |  | transmembrane |
| BCJ09931.1 | putative ABC transporter ATP-binding protein | 6 | 5 | 62.7 | 19.74 |  | transmembrane |
| BCJ10876.1 | protease HtpX | 6 | 4 | 33.1 | 13.51 | *htpX* | transmembrane |
| BCJ10496.1 | ABC transporter permease | 6 | 3 | 33.7 | 17.11 |  | transmembrane |
| BCJ10499.1 | glycerol-3-phosphate acyltransferase | 6 | 2 | 22.9 | 19.1 | *plsY* | transmembrane |
| BCJ11316.1 | cell division protein FtsL | 6 | 3 | 12.2 | 17.42 | *ftsL* | transmembrane |
| BCJ10775.1 | ABC transporter ATP-binding protein | 6 | 6 | 59.7 | 15.77 |  | transmembrane |
| BCJ10225.1 | ABC transporter ATP-binding protein | 6 | 6 | 24.4 | 19.86 |  | transmembrane |
| BCJ09718.1 | potassium transporter Trk | 6 | 4 | 25 | 17.72 | *trkA1* | transmembrane |
| BCJ10321.1 | hypothetical protein SMNM65_07530 | 5 | 5 | 146.6 | 8.27 |  | transmembrane |
| BCJ11040.1 | hypothetical protein SMNM65_14720 | 5 | 5 | 76 | 14.45 |  | transmembrane |
| BCJ11444.1 | multidrug ABC transporter ATP-binding protein | 5 | 5 | 64.1 | 8.12 |  | transmembrane |
| BCJ11429.1 | acyltransferase | 5 | 5 | 67.4 | 15.87 |  | transmembrane |
| BCJ10111.1 | glycosyl transferase | 5 | 5 | 26.7 | 12.16 |  | transmembrane |
| BCJ09908.1 | ABC transporter | 5 | 5 | 64.8 | 10.07 |  | transmembrane |
| BCJ09753.1 | amino acid ABC transporter ATP-binding protein | 5 | 4 | 29.2 | 12.88 |  | transmembrane |
| BCJ10345.1 | hypothetical protein SMNM65_07770 | 5 | 4 | 22.5 | 14.62 |  | transmembrane |
| BCJ09577.1 | septum formation initiation protein | 5 | 3 | 14.9 | 17.35 |  | transmembrane |
| BCJ10916.1 | hypothetical protein SMNM65_13480 | 5 | 3 | 11.6 | 8.52 |  | transmembrane |
| BCJ11471.1 | maltodextrose utilization protein MalA | 5 | 2 | 30.6 | 15.61 | *malA* | transmembrane |
| BCJ11159.1 | branched-chain amino acid transport system carrier protein | 5 | 4 | 46.6 | 8.76 |  | transmembrane |
| BCJ10970.1 | amino acid ABC transporter permease | 5 | 4 | 28.9 | 13.44 |  | transmembrane |
| BCJ11279.1 | sodium:alanine symporter | 5 | 2 | 46.7 | 15.28 | *dagA* | transmembrane |
| BCJ11524.1 | D-alanyl-lipoteichoic acid biosynthesis protein DltB | 5 | 2 | 48.8 | 7.1 | *dltB* | transmembrane |
| BCJ11001.1 | ABC transporter ATP-binding protein | 5 | 4 | 28.2 | 5.04 | *livG* | transmembrane |
| BCJ10306.1 | sodium:hydrogen antiporter | 4 | 4 | 77.7 | 10.38 |  | transmembrane |
| BCJ10346.1 | phosphate import ATP-binding protein PstB | 4 | 4 | 28 | 14.04 | *pstB1* | transmembrane |
| BCJ10924.1 | glutamine ABC transporter substrate-binding protein | 4 | 4 | 31.8 | 9.6 |  | transmembrane |
| BCJ10331.1 | hypothetical protein SMNM65_07630 | 4 | 4 | 271.9 | 5.06 |  | transmembrane |
| BCJ10160.1 | ABC transporter ATP-binding protein | 4 | 4 | 58.4 | 14.41 |  | transmembrane |
| BCJ09981.1 | DNA-directed RNA polymerase subunit beta | 4 | 2 | 7 | 4.49 |  | transmembrane |
| BCJ09797.1 | magnesium transporter | 4 | 2 | 36.4 | 12.55 | *corA* | transmembrane |
| BCJ10854.1 | hypothetical protein SMNM65_12860 | 4 | 2 | 13.7 | 9.46 |  | transmembrane |
| BCJ11079.1 | amino acid transporter | 4 | 2 | 49.3 | 11.6 |  | transmembrane |
| BCJ09708.1 | DUF4956 domain-containing protein | 4 | 2 | 25.3 | 12.26 |  | transmembrane |
| BCJ09852.1 | ABC transporter ATP-binding protein | 4 | 4 | 40.6 | 10.41 |  | transmembrane |
| BCJ10080.1 | PTS fructose transporter subunit IID | 4 | 4 | 30.1 | 9.26 | *manZ_4* | transmembrane |
| BCJ09928.1 | hypothetical protein SMNM65_03600 | 4 | 3 | 38.6 | 16.69 |  | transmembrane |
| BCJ09987.1 | hypothetical protein SMNM65_04190 | 4 | 3 | 18.5 | 7.13 |  | transmembrane |
| BCJ11372.1 | xanthine/uracil permease | 4 | 3 | 49.2 | 11.68 |  | transmembrane |
| BCJ11134.1 | ABC transporter permease | 4 | 2 | 31.8 | 12.22 | *dacA* | transmembrane |
| BCJ11092.1 | polysaccharide biosynthesis protein | 4 | 2 | 60.2 | 4.17 |  | transmembrane |
| BCJ11230.1 | protein translocase subunit SecA | 4 | 3 | 89.3 | 11.71 | *secA_2* | transmembrane |
| BCJ11127.1 | antibiotic ABC transporter ATP-binding protein | 4 | 4 | 70.7 | 14.77 |  | transmembrane |
| BCJ10093.1 | ABC transporter ATP-binding protein | 4 | 3 | 27.1 | 11.14 | *proV* | transmembrane |
| BCJ09752.1 | bacteriocin ABC transporter ATP-binding protein | 4 | 3 | 23.6 | 14.06 |  | transmembrane |
| BCJ09950.1 | ABC transporter ATP-binding protein | 4 | 2 | 23.9 | 10.95 |  | transmembrane |
| BCJ11166.1 | peptidase | 3 | 2 | 26.9 | 10.94 |  | transmembrane |
| BCJ11066.1 | zinc metalloprotease | 3 | 3 | 216.6 | 7.68 | *zmpB* | transmembrane |
| BCJ10316.1 | DUF948 domain containing protein | 3 | 3 | 14.5 | 8.7 |  | transmembrane |
| BCJ10394.1 | membrane protein | 3 | 3 | 56.3 | 9.12 |  | transmembrane |
| BCJ10242.1 | membrane protein | 3 | 3 | 39.2 | 8.29 |  | transmembrane |
| BCJ11070.1 | peptide-methionine (R)-S-oxide reductase | 3 | 3 | 42 | 11.17 | *msrB* | transmembrane |
| BCJ10726.1 | ABC transporter permease | 3 | 3 | 45 | 11.92 |  | transmembrane |
| BCJ10107.1 | hypothetical protein SMNM65_05390 | 3 | 3 | 17 | 8.74 |  | transmembrane |
| BCJ11020.1 | hypothetical protein SMNM65_14520 | 3 | 3 | 27.9 | 5.76 |  | transmembrane |
| BCJ10862.1 | polysaccharide biosynthesis protein MviN | 3 | 3 | 56.4 | 6.02 | *tacF* | transmembrane |
| BCJ10161.1 | membrane protein | 3 | 2 | 28.7 | 3.61 |  | transmembrane |
| BCJ10250.1 | membrane protein | 3 | 2 | 38.7 | 8.86 |  | transmembrane |
| BCJ10827.1 | CAAX amino protease | 3 | 2 | 35.5 | 7.06 |  | transmembrane |
| BCJ09976.1 | membrane protein | 3 | 2 | 24.8 | 4.35 |  | transmembrane |
| BCJ09649.1 | protease PrsW | 3 | 2 | 30.3 | 6.81 |  | transmembrane |
| BCJ10683.1 | hypothetical protein SMNM65_11150 | 3 | 2 | 18.2 | 13.46 |  | transmembrane |
| BCJ10919.1 | glycosyl hydrolase family 25 | 3 | 2 | 30.2 | 7.27 |  | transmembrane |
| BCJ10341.1 | phosphate-binding protein | 3 | 3 | 27.5 | 8.64 | *pstS* | transmembrane |
| BCJ10221.1 | ABC transporter permease | 3 | 3 | 97.3 | 6.83 |  | transmembrane |
| BCJ10665.1 | ABC transporter permease | 3 | 2 | 74.5 | 4.94 |  | transmembrane |
| BCJ10186.1 | sugar ABC transporter permease | 3 | 2 | 24.9 | 6.65 |  | transmembrane |
| BCJ11469.1 | sugar ABC transporter permease | 3 | 2 | 47.7 | 8.61 | *malC* | transmembrane |
| BCJ10466.1 | glutamine ABC transporter permease | 3 | 2 | 24.9 | 5.79 | *artQ* | transmembrane |
| BCJ11223.1 | serine/threonine transporter SstT | 3 | 2 | 42 | 7.98 | *sstT* | transmembrane |
| BCJ09964.1 | multidrug ABC transporter ATP-binding protein | 3 | 3 | 33.1 | 10.01 |  | transmembrane |
| BCJ09996.1 | ABC transporter substrate-binding protein | 3 | 3 | 35.4 | 10.12 |  | transmembrane |
| BCJ10422.1 | ABC transporter ATP-binding protein | 3 | 3 | 58.3 | 9.63 |  | transmembrane |
| BCJ10607.1 | PTS lactose IIA component | 3 | 3 | 11.4 | 9.43 | *lacF* | transmembrane |
| BCJ10298.1 | ABC transporter ATP-binding protein | 3 | 2 | 37.7 | 5.3 |  | transmembrane |
| BCJ10655.1 | two-component sensor histidine kinase | 3 | 2 | 34.1 | 7.66 |  | transmembrane |
| BCJ10925.1 | amino acid ABC transporter ATP-binding protein | 3 | 2 | 23.2 | 11.16 | *glnQ_3* | transmembrane |
| BCJ09678.1 | hypothetical protein SMNM65_01100 | 2 | 2 | 31 | 6.31 |  | transmembrane |
| BCJ10614.1 | hypothetical protein SMNM65_10460 | 2 | 2 | 30.3 | 2.91 |  | transmembrane |
| BCJ10570.1 | hypothetical protein SMNM65_10020 | 2 | 2 | 6.8 | 3.9 |  | transmembrane |
| BCJ10270.1 | hypothetical protein SMNM65_07020 | 2 | 2 | 24.4 | 3.67 |  | transmembrane |
| BCJ11076.1 | hypothetical protein SMNM65_15080 | 2 | 2 | 19 | 4.94 |  | transmembrane |
| BCJ09949.1 | hypothetical protein SMNM65_03810 | 2 | 2 | 75.3 | 2.66 |  | transmembrane |
| BCJ11448.1 | hypothetical protein SMNM65_18800 | 2 | 2 | 9.7 | 5 |  | transmembrane |
| BCJ10455.1 | rod shape-determining protein RodA | 2 | 2 | 45.3 | 5.14 | *rodA* | transmembrane |
| BCJ09870.1 | ABC transporter permease | 2 | 2 | 31.6 | 4.76 |  | transmembrane |
| BCJ11390.1 | sensor histidine kinase | 2 | 2 | 42.2 | 2.61 | *hk11* | transmembrane |
| BCJ10227.1 | hypothetical protein SMNM65_06590 | 2 | 2 | 34.1 | 1.78 |  | transmembrane |
| BCJ10547.1 | cell division protein FtsW | 2 | 2 | 45.2 | 6.8 | *ftsW* | transmembrane |
| BCJ11470.1 | sugar ABC transporter permease | 2 | 2 | 31.5 | 4.4 | *malD* | transmembrane |
| BCJ10185.1 | sugar ABC transporter permease | 2 | 2 | 33.7 | 5.26 |  | transmembrane |
| BCJ10536.1 | preprotein translocase subunit SecG | 2 | 2 | 8.5 | 6.32 | *secG* | transmembrane |
| BCJ10753.1 | membrane protein | 2 | 2 | 25.4 | 5.06 |  | transmembrane |
| BCJ11585.1 | energy-coupling factor transporter ATP-binding protein EcfA2 | 2 | 2 | 30.5 | 6.17 | *ecfA2* | transmembrane |
| BCJ09998.1 | ABC transporter ATP-binding protein | 2 | 2 | 29.6 | 4.56 | *cmpC* | transmembrane |
| BCJ10255.1 | ABC transporter | 2 | 2 | 28.5 | 6.85 | *fecE* | transmembrane |
| BCJ11231.1 | accessory Sec system protein Asp3 | 2 | 2 | 17.2 | 2.61 |  | transmembrane |
| BCJ11233.1 | hypothetical protein SMNM65_16650 | 2 | 2 | 31.9 | 8.05 |  | transmembrane |
| BCJ11031.1 | ABC transporter ATP-binding protein | 2 | 2 | 23.8 | 2 |  | transmembrane |
| BCJ10228.1 | ABC transporter ATP-binding protein | 2 | 2 | 23.8 | 8.52 |  | transmembrane |
| BCJ09656.1 | hypothetical protein SMNM65_00880 | 1358 | 24 | 35.1 | 1692.77 |  |  |
| BCJ09657.1 | hypothetical protein SMNM65_00890 | 369 | 36 | 48.4 | 1083.58 |  |  |
| BCJ09675.1 | hypothetical protein SMNM65_01070 | 210 | 18 | 34 | 555.59 |  |  |
| BCJ09658.1 | hypothetical protein SMNM65_00900 | 190 | 40 | 59.4 | 681.72 |  |  |
| BCJ10788.1 | hypothetical protein SMNM65_12200 | 76 | 44 | 125.2 | 232.1 |  |  |
| BCJ11251.1 | hypothetical protein SMNM65_16830 | 70 | 52 | 282.3 | 192.37 |  |  |
| BCJ09896.1 | hypothetical protein SMNM65_03280 | 53 | 12 | 28.6 | 165.82 |  |  |
| BCJ09659.1 | hypothetical protein SMNM65_00910 | 49 | 18 | 24.8 | 144.09 |  |  |
| BCJ11033.1 | hypothetical protein SMNM65_14650 | 48 | 8 | 14.4 | 189.62 |  |  |
| BCJ09848.1 | hypothetical protein SMNM65_02800 | 43 | 17 | 36.7 | 137.73 |  |  |
| BCJ11311.1 | UPF0371 protein | 40 | 19 | 50.6 | 122.26 |  |  |
| BCJ11541.1 | hypothetical protein SMNM65_19730 | 36 | 9 | 24.1 | 112.73 |  |  |
| BCJ10129.1 | hypothetical protein SMNM65_05610 | 35 | 25 | 95.7 | 106.75 |  |  |
| BCJ11401.1 | hypothetical protein SMNM65_18330 | 33 | 18 | 57.9 | 95.11 |  |  |
| BCJ10695.1 | hypothetical protein SMNM65_11270 | 29 | 17 | 35.3 | 75.86 |  |  |
| BCJ10326.1 | hypothetical protein SMNM65_07580 | 28 | 16 | 214.4 | 85.23 |  |  |
| BCJ10429.1 | hypothetical protein SMNM65_08610 | 28 | 9 | 12.9 | 77.26 | *nrdD_2* |  |
| BCJ09803.1 | hypothetical protein SMNM65_02350 | 26 | 10 | 21 | 79.04 |  |  |
| BCJ10909.1 | hypothetical protein SMNM65_13410 | 20 | 12 | 80.4 | 74.95 |  |  |
| BCJ10824.1 | hypothetical protein SMNM65_12560 | 17 | 11 | 25 | 38.52 |  |  |
| BCJ09589.1 | hypothetical protein SMNM65_00210 | 14 | 9 | 27.5 | 47.46 |  |  |
| BCJ10696.1 | hypothetical protein SMNM65_11280 | 13 | 6 | 22.9 | 48.79 |  |  |
| BCJ10369.1 | hypothetical protein SMNM65_08010 | 12 | 10 | 49.8 | 29.41 |  |  |
| BCJ09847.1 | hypothetical protein SMNM65_02790 | 12 | 6 | 23.9 | 40.08 |  |  |
| BCJ10324.1 | hypothetical protein SMNM65_07560 | 11 | 9 | 35.3 | 35.02 |  |  |
| BCJ09736.1 | hypothetical protein SMNM65_01680 | 11 | 9 | 36 | 29.61 |  |  |
| BCJ10750.1 | hypothetical protein SMNM65_11820 | 11 | 7 | 15.8 | 33.82 |  |  |
| BCJ10935.1 | hypothetical protein SMNM65_13670 | 11 | 4 | 5.5 | 28.75 |  |  |
| BCJ09628.1 | hypothetical protein SMNM65_00600 | 10 | 8 | 68.6 | 27.57 |  |  |
| BCJ10845.1 | hypothetical protein SMNM65_12770 | 9 | 7 | 64.4 | 25.24 |  |  |
| BCJ10648.1 | hypothetical protein SMNM65_10800 | 8 | 7 | 131.4 | 20.72 |  |  |
| BCJ10287.1 | hypothetical protein SMNM65_07190 | 8 | 6 | 356.5 | 19.36 |  |  |
| BCJ10048.1 | hypothetical protein SMNM65_04800 | 8 | 6 | 18.7 | 18.2 |  |  |
| BCJ10825.1 | hypothetical protein SMNM65_12570 | 8 | 5 | 22 | 23.9 |  |  |
| BCJ11107.1 | hypothetical protein SMNM65_15390 | 6 | 6 | 60.1 | 15.75 |  |  |
| BCJ11085.1 | hypothetical protein SMNM65_15170 | 6 | 6 | 23.8 | 16.93 |  |  |
| BCJ10060.1 | hypothetical protein SMNM65_04920 | 6 | 6 | 66.1 | 13.4 |  |  |
| BCJ09629.1 | hypothetical protein SMNM65_00610 | 6 | 5 | 29.1 | 11.89 |  |  |
| BCJ09710.1 | hypothetical protein SMNM65_01420 | 6 | 5 | 41.7 | 18.13 |  |  |
| BCJ10320.1 | hypothetical protein SMNM65_07520 | 6 | 5 | 40 | 15.05 |  |  |
| BCJ09674.1 | hypothetical protein SMNM65_01060 | 6 | 3 | 25.3 | 11.75 |  |  |
| BCJ09661.1 | hypothetical protein SMNM65_00930 | 6 | 2 | 16.9 | 12.35 |  |  |
| BCJ10941.1 | hypothetical protein SMNM65_13730 | 5 | 4 | 43.9 | 15.49 |  |  |
| BCJ10025.1 | hypothetical protein SMNM65_04570 | 5 | 3 | 11 | 16.74 |  |  |
| BCJ10292.1 | hypothetical protein SMNM65_07240 | 5 | 3 | 91.6 | 10.86 |  |  |
| BCJ11090.1 | hypothetical protein SMNM65_15220 | 5 | 3 | 17.8 | 13.18 |  |  |
| BCJ09895.1 | hypothetical protein SMNM65_03270 | 5 | 3 | 10.2 | 14.46 |  |  |
| BCJ10823.1 | hypothetical protein SMNM65_12550 | 4 | 4 | 23.4 | 11.4 |  |  |
| BCJ11133.1 | hypothetical protein SMNM65_15650 | 4 | 4 | 28 | 11.91 |  |  |
| BCJ10649.1 | hypothetical protein SMNM65_10810 | 4 | 4 | 45.4 | 11.59 |  |  |
| BCJ11259.1 | hypothetical protein SMNM65_16910 | 4 | 3 | 14.2 | 5.56 |  |  |
| BCJ10956.1 | hypothetical protein SMNM65_13880 | 4 | 2 | 9 | 10.2 |  |  |
| BCJ09799.1 | hypothetical protein SMNM65_02310 | 4 | 2 | 16.6 | 14.05 |  |  |
| BCJ10975.1 | hypothetical protein SMNM65_14070 | 4 | 2 | 16.8 | 8.1 |  |  |
| BCJ09627.1 | hypothetical protein SMNM65_00590 | 3 | 3 | 27 | 9.15 |  |  |
| BCJ09616.1 | hypothetical protein SMNM65_00480 | 3 | 3 | 21.6 | 8.59 | *pi232* |  |
| BCJ11178.1 | hypothetical protein SMNM65_16100 | 3 | 3 | 33.8 | 10.01 |  |  |
| BCJ09604.1 | hypothetical protein SMNM65_00360 | 3 | 3 | 27.6 | 9.52 |  |  |
| BCJ10927.1 | hypothetical protein SMNM65_13590 | 3 | 3 | 47.5 | 9.17 |  |  |
| BCJ10051.1 | hypothetical protein SMNM65_04830 | 3 | 3 | 28.1 | 7.46 |  |  |
| BCJ11109.1 | hypothetical protein SMNM65_15410 | 3 | 3 | 27.4 | 7.79 |  |  |
| BCJ10139.1 | hypothetical protein SMNM65_05710 | 3 | 3 | 18 | 7.41 |  |  |
| BCJ11385.1 | hypothetical protein SMNM65_18170 | 3 | 3 | 16.7 | 6.85 |  |  |
| BCJ10929.1 | hypothetical protein SMNM65_13610 | 3 | 3 | 37.8 | 7.31 |  |  |
| BCJ09631.1 | hypothetical protein SMNM65_00630 | 3 | 3 | 53.8 | 8.63 |  |  |
| BCJ10461.1 | hypothetical protein SMNM65_08930 | 3 | 2 | 11.3 | 9.74 |  |  |
| BCJ09622.1 | hypothetical protein SMNM65_00540 | 3 | 2 | 12.6 | 12.39 |  |  |
| BCJ09635.1 | hypothetical protein SMNM65_00670 | 3 | 2 | 17.2 | 9.38 |  |  |
| BCJ11242.1 | hypothetical protein SMNM65_16740 | 2 | 2 | 47.8 | 4.56 |  |  |
| BCJ09637.1 | hypothetical protein SMNM65_00690 | 2 | 2 | 24.1 | 5.57 |  |  |
| BCJ10361.1 | hypothetical protein SMNM65_07930 | 2 | 2 | 67.3 | 7.21 |  |  |
| BCJ09574.1 | hypothetical protein SMNM65_00060 | 2 | 2 | 11.4 | 5.38 |  |  |
| BCJ10084.1 | UPF0374 protein | 2 | 2 | 21.3 | 3 |  |  |
| BCJ11188.1 | hypothetical protein SMNM65_16200 | 2 | 2 | 65.9 | 4.02 |  |  |
| BCJ11588.1 | hypothetical protein SMNM65_20200 | 2 | 2 | 16.3 | 6.29 |  |  |
| BCJ10062.1 | hypothetical protein SMNM65_04940 | 2 | 2 | 60 | 3.77 |  |  |
| BCJ10131.1 | hypothetical protein SMNM65_05630 | 2 | 2 | 48.3 | 4.72 |  |  |
| BCJ10914.1 | hypothetical protein SMNM65_13460 | 2 | 2 | 8.2 | 3.51 |  |  |
| BCJ11461.1 | hypothetical protein SMNM65_18930 | 2 | 2 | 23.5 | 8.1 |  |  |

Accession, Identification numbers assigned to the nucleotide sequences of genes published by the DNA Data Base of Japan. #PSM, Peptide-spectrum match. #peptides, Different types of detection peptides derived from the same protein. Score, Values obtained from mass spectrometry measurement results.
