# Supplementary material for: Functional characteristics of membrane vesicles produced by Streptococcus mitis
Source: J Oral Microbiol. 2025 Sep 23;17(1):2557962. doi: 10.1080/20002297.2025.2557962 (PMC12459155; doi:10.1080/20002297.2025.2557962)
Supplement: Supplementary material — Supplementary Table 4 [file ZJOM_A_2557962_SM1729.docx]

**Supplementary Table 4.** Mass spectrometry identification of *S. mitis* Nm-76 proteins present in MVs and their topology prediction

| Accession | Description | # PSMs | # Peptides | MW [kDa] | Score | Gene | Localization |
| --- | --- | --- | --- | --- | --- | --- | --- |
| EGP70539.1 | cell wall-binding repeat protein | 268 | 22 | 43.5 | 788.14 |  | cell wall |
| EGP70855.1 | periplasmic-binding protein | 42 | 17 | 36.7 | 93.92 |  | cell wall |
| EGP68176.1 | cell wall-binding repeat protein | 29 | 13 | 41.5 | 58.79 |  | cell wall |
| EGP70015.1 | gram positive anchor | 12 | 9 | 193.4 | 24.65 |  | cell wall |
| EGP70021.1 | cell wall-binding repeat protein | 7 | 2 | 37.3 | 18 |  | cell wall |
| EGP69220.1 | gram positive anchor | 7 | 7 | 234.6 | 17.91 |  | cell wall |
| EGP65453.1 | gram positive anchor | 5 | 4 | 230.5 | 7.97 |  | cell wall |
| EGP70759.1 | CobB/CobQ-like glutamine amidotransferase domain protein | 4 | 3 | 29.1 | 7.74 | gatD | cell wall |
| EGP70789.1 | ABC transporter, solute-binding protein | 126 | 27 | 49.6 | 359.98 |  | cytosolic |
| EGP65168.1 | translation elongation factor Tu | 61 | 20 | 44 | 178.54 | tuf | cytosolic |
| EGP69219.1 | formate C-acetyltransferase | 60 | 30 | 91.3 | 147.28 |  | cytosolic |
| EGP70677.1 | glyceraldehyde-3-phosphate dehydrogenase, type I | 57 | 14 | 35.9 | 147.04 | gap | cytosolic |
| EGP70625.1 | chaperone protein DnaK | 45 | 25 | 64.7 | 112.16 | dnaK | cytosolic |
| EGP69096.1 | formate C-acetyltransferase | 47 | 22 | 87.8 | 110.14 | pflB | cytosolic |
| EGP70632.1 | aldehyde-alcohol dehydrogenase 2 | 41 | 19 | 97.1 | 106.5 | adh | cytosolic |
| EGP69555.1 | endo-beta-N-acetylglucosaminidase domain protein | 40 | 9 | 50.8 | 104.74 |  | cytosolic |
| EGP68963.1 | DNA-directed RNA polymerase, beta' subunit | 46 | 32 | 137 | 93.64 | rpoC | cytosolic |
| EGP68916.1 | ribosomal protein S2 | 33 | 12 | 28.8 | 89.57 | rpsB | cytosolic |
| EGP65164.1 | metallo-beta-lactamase domain protein | 31 | 9 | 72.1 | 74.72 |  | cytosolic |
| EGP69927.1 | peptidyl-prolyl cis-trans isomerase, cyclophilin-type | 23 | 10 | 29.1 | 70.01 |  | cytosolic |
| EGP67804.1 | pyruvate oxidase | 27 | 16 | 65.2 | 69.5 | pox5 | cytosolic |
| EGP68595.1 | phosphoenolpyruvate-protein phosphotransferase | 25 | 10 | 63.2 | 69.36 | ptsI; ptsP | cytosolic |
| EGP69007.1 | NMT1/THI5-like protein | 27 | 13 | 37.8 | 68.88 |  | cytosolic |
| EGP67618.1 | ABC transporter substrate binding protein | 25 | 14 | 34.6 | 67.83 |  | cytosolic |
| EGP69769.1 | phosphoglycerate kinase | 26 | 12 | 41.9 | 64.64 | pgk | cytosolic |
| EGP69654.1 | serine-type D-Ala-D-Ala carboxypeptidase | 26 | 10 | 26.4 | 64.57 |  | cytosolic |
| EGP68599.1 | pyruvate kinase | 27 | 14 | 54.7 | 61.11 | pyk | cytosolic |
| EGP69591.1 | putative mannosyl-glycoprotein endo-beta-N-acetylglucosaminidase | 27 | 5 | 23.1 | 61.04 |  | cytosolic |
| EGP65305.1 | phosphoglucomutase | 21 | 14 | 62.6 | 59.21 | manB; | cytosolic |
| EGP69110.1 | ABC transporter, solute-binding protein | 21 | 10 | 37.7 | 55 |  | cytosolic |
| EGP69112.1 | ABC transporter, substrate-binding protein, family 3 | 23 | 12 | 30.6 | 53.95 |  | cytosolic |
| EGP69771.1 | putative DNA/RNA non-specific endonuclease | 16 | 8 | 29.9 | 53.39 |  | cytosolic |
| EGP68906.1 | DNA-directed RNA polymerase, beta subunit | 22 | 16 | 134.2 | 52.62 | rpoB | cytosolic |
| EGP70788.1 | phosphoglycerate mutase 1 family | 18 | 9 | 26 | 50.48 | gpmA; | cytosolic |
| EGP70567.1 | tyrosine--tRNA ligase | 2 | 2 | 47.4 | 50.39 | tyrS | cytosolic |
| EGP69925.1 | ribosomal protein L10 | 10 | 6 | 17.5 | 49.14 | rplJ | cytosolic |
| EGP68979.1 | 50S ribosomal protein L5 | 18 | 9 | 19.8 | 48.91 | rplE | cytosolic |
| EGP69198.1 | LytTr DNA-binding domain protein | 13 | 9 | 29.9 | 48.07 |  | cytosolic |
| EGP70545.1 | ribosomal protein S4 | 21 | 10 | 23 | 47.36 | rpsD | cytosolic |
| EGP70837.1 | ABC transporter, ATP-binding protein | 19 | 9 | 25.6 | 46.92 | skfE | cytosolic |
| EGP70860.1 | DivIVA protein | 16 | 6 | 32.9 | 46.59 |  | cytosolic |
| EGP70802.1 | threonine--tRNA ligase | 17 | 10 | 74.6 | 45.49 | thrS | cytosolic |
| EGP69793.1 | translation elongation factor G | 16 | 14 | 76.8 | 45.3 | fus; fusA | cytosolic |
| EGP69099.1 | DAK2 domain fusion protein YloV | 18 | 12 | 60.1 | 45.17 |  | cytosolic |
| EGP70766.1 | fructose-1,6-bisphosphate aldolase, class II | 20 | 10 | 31.5 | 42.68 | fba | cytosolic |
| EGP69116.1 | translation elongation factor Ts | 16 | 8 | 37.4 | 41.97 | tsf | cytosolic |
| EGP69161.1 | ribosomal protein S3 | 18 | 9 | 24 | 41.85 | rpsC | cytosolic |
| EGP70491.1 | cysteine-rich secretory family protein | 14 | 6 | 43.4 | 41.22 |  | cytosolic |
| EGP70694.1 | ABC transporter, solute-binding protein | 13 | 7 | 39.1 | 39.68 |  | cytosolic |
| EGP65252.1 | putative pneumococcal vaccine antigen A | 12 | 6 | 22.9 | 39.02 |  | cytosolic |
| EGP68624.1 | L-lactate dehydrogenase | 14 | 7 | 35.3 | 38.94 | ldh | cytosolic |
| EGP69193.1 | sugar-binding domain protein | 16 | 10 | 37.2 | 37.62 |  | cytosolic |
| EGP65296.1 | glycine hydroxymethyltransferase | 15 | 11 | 45.2 | 36.57 | glyA | cytosolic |
| EGP65428.1 | spermidine/putrescine ABC transporter, periplasmic spermidine/putrescine-binding protein PotD | 15 | 9 | 41 | 36.41 | potD | cytosolic |
| EGP70022.1 | phosphogluconate dehydrogenase (decarboxylating) | 15 | 9 | 52.6 | 36.02 | cbpJ | cytosolic |
| EGP69206.1 | ribosomal protein L16 | 12 | 5 | 15.4 | 35.96 | rplP | cytosolic |
| EGP69761.1 | ribosomal protein S7 | 12 | 7 | 17.7 | 35.55 | rpsG | cytosolic |
| EGP68181.1 | translation initiation factor IF-2 | 10 | 9 | 102.6 | 32.58 | infB | cytosolic |
| EGP69180.1 | ribosomal protein L2 | 26 | 8 | 29.9 | 32.04 | rplB | cytosolic |
| EGP69471.1 | FemAB family protein | 4 | 2 | 47.6 | 31.24 | murM | cytosolic |
| EGP69118.1 | glycerol dehydrogenase | 12 | 8 | 39.2 | 31.2 | gldA | cytosolic |
| EGP68616.1 | 6-phosphofructokinase | 11 | 7 | 35.3 | 30.08 | pfkA | cytosolic |
| EGP67793.1 | CBS domain protein | 9 | 4 | 24.2 | 29.82 |  | cytosolic |
| EGP68620.1 | sortase family protein | 10 | 5 | 28.2 | 29.66 |  | cytosolic |
| EGP69626.1 | DNA protection during starvation protein | 10 | 4 | 19.3 | 29.23 | dps | cytosolic |
| EGP67747.1 | L-lactate oxidase | 12 | 8 | 41.5 | 29.19 | lctO | cytosolic |
| EGP68231.1 | hypothetical protein HMPREF9958_0440 | 14 | 4 | 14.4 | 28.6 |  | cytosolic |
| EGP69051.1 | 50S ribosomal protein L3 | 8 | 2 | 22.2 | 28.06 | rplC | cytosolic |
| EGP70601.1 | glucose-6-phosphate isomerase | 12 | 8 | 49.9 | 27.78 | gpi; pgi | cytosolic |
| EGP69442.1 | transcription elongation factor GreA | 2 | 2 | 17.5 | 27.5 | greA | cytosolic |
| EGP67705.1 | uracil phosphoribosyltransferase | 9 | 5 | 23.6 | 27.48 | upp | cytosolic |
| EGP67751.1 | flavin reductase | 12 | 8 | 45.7 | 27.47 |  | cytosolic |
| EGP70841.1 | cell division protein FtsZ | 9 | 5 | 44.1 | 27.15 | ftsZ | cytosolic |
| EGP69807.1 | UDP-glucose 4-epimerase | 9 | 5 | 37.1 | 24.82 | galE | cytosolic |
| EGP68913.1 | ribosomal protein L17 | 10 | 5 | 14.5 | 24.75 | rplQ | cytosolic |
| EGP69751.1 | putative aminotransferase AlaT | 8 | 6 | 45.7 | 24.27 | alaA; | cytosolic |
| EGP69505.1 | triose-phosphate isomerase | 9 | 6 | 26.5 | 23.76 | tpiA | cytosolic |
| EGP70680.1 | transketolase | 10 | 9 | 71.1 | 23.35 | tkt | cytosolic |
| EGP65293.1 | LICD family protein | 9 | 6 | 32 | 23.25 |  | cytosolic |
| EGP69139.1 | ribosomal protein L6 | 8 | 5 | 19.5 | 23.03 | rplF | cytosolic |
| EGP69535.1 | putative enoyl-[acyl-carrier-protein] reductase II | 8 | 6 | 34 | 22.72 |  | cytosolic |
| EGP68586.1 | ribonucleoside-diphosphate reductase, alpha subunit | 9 | 7 | 81.5 | 22.49 |  | cytosolic |
| EGP69575.1 | ribosomal protein L1 | 11 | 7 | 24.5 | 21.64 | rl1; rplA | cytosolic |
| EGP65330.1 | signal recognition particle protein | 10 | 7 | 57.7 | 20.95 | ffh | cytosolic |
| EGP68451.1 | 30S ribosomal protein S1 | 10 | 8 | 43.8 | 20.75 | rpsA | cytosolic |
| EGP69611.1 | inorganic pyrophosphatase PpaC | 8 | 7 | 33.4 | 20.63 | ppaC | cytosolic |
| EGP67611.1 | ribosomal protein L20 | 8 | 4 | 13.7 | 20.57 | rplT | cytosolic |
| EGP70816.1 | neprilysin | 9 | 7 | 71.8 | 20.44 | pepO | cytosolic |
| EGP69148.1 | putative proline--tRNA ligase | 9 | 8 | 68.6 | 19.94 | proS | cytosolic |
| EGP70586.1 | putative nodulation ATP-binding protein I | 8 | 6 | 30.4 | 19.85 |  | cytosolic |
| EGP69147.1 | ribosomal protein S5 | 12 | 9 | 17 | 19.43 | rpsE | cytosolic |
| EGP69188.1 | 30S ribosomal protein S11 | 7 | 4 | 13.4 | 19.13 | rpsK; | cytosolic |
| EGP69916.1 | ribosomal protein L7/L12 | 6 | 5 | 12.4 | 18.77 | rplL | cytosolic |
| EGP65267.1 | thiol-disulfide oxidoreductase ResA family protein | 9 | 6 | 21.1 | 18.47 | resA | cytosolic |
| EGP70806.1 | DEAD-box ATP-dependent RNA helicase CshA | 8 | 8 | 58.8 | 18.33 | cshA | cytosolic |
| EGP67780.1 | glycine--tRNA ligase, beta subunit | 7 | 5 | 75.4 | 18.3 | glyS | cytosolic |
| EGP69040.1 | leucine--tRNA ligase | 6 | 5 | 94.1 | 18.19 | leuS | cytosolic |
| EGP70729.1 | ribosomal protein S15 | 7 | 3 | 10.5 | 18.04 | rpsO | cytosolic |
| EGP69515.1 | endo-beta-N-acetylglucosaminidase domain protein | 9 | 6 | 61.5 | 17.93 |  | cytosolic |
| EGP68784.1 | tyrosine-protein kinase CpsD | 7 | 3 | 25.6 | 17.9 | cpsD | cytosolic |
| EGP69541.1 | GTP-binding protein TypA | 8 | 6 | 69 | 17.82 | bipA | cytosolic |
| EGP69477.1 | conserved domain protein | 7 | 5 | 44.8 | 17.66 |  | cytosolic |
| EGP70566.1 | ribose-phosphate diphosphokinase | 5 | 4 | 35.4 | 17.48 |  | cytosolic |
| EGP69497.1 | peptide chain release factor 3 | 7 | 7 | 58.4 | 17.41 | prfC | cytosolic |
| EGP65312.1 | glucose-1-phosphate adenylyltransferase, GlgD subunit | 7 | 6 | 42.6 | 17.13 | glgD | cytosolic |
| EGP69745.1 | universal stress family protein | 10 | 6 | 16.6 | 16.81 |  | cytosolic |
| EGP67718.1 | bacterial capsule synthesis protein | 9 | 8 | 50.3 | 16.8 |  | cytosolic |
| EGP69011.1 | ribosomal protein L23 | 7 | 4 | 10.8 | 16.36 | rplW | cytosolic |
| EGP69173.1 | biofilm regulatory protein A | 9 | 3 | 39 | 16.24 | brpA | cytosolic |
| EGP67566.1 | phosphoenolpyruvate carboxylase | 6 | 5 | 103.2 | 16.22 | ppc | cytosolic |
| EGP69914.1 | superoxide dismutase | 5 | 4 | 22.4 | 16.14 | sodA | cytosolic |
| EGP69786.1 | aspartate--ammonia ligase | 7 | 7 | 37.5 | 16.03 | asnA | cytosolic |
| EGP69283.1 | Rib/alpha-like repeat protein, partial | 7 | 5 | 55.5 | 15.89 |  | cytosolic |
| EGP68969.1 | ribosomal protein L22 | 5 | 3 | 12.2 | 15.68 | rplV | cytosolic |
| EGP70763.1 | oxidoreductase, NAD-binding domain protein | 7 | 7 | 41.1 | 15.14 |  | cytosolic |
| EGP69597.1 | asparagine--tRNA ligase | 6 | 6 | 51 | 14.97 | asnS | cytosolic |
| EGP69048.1 | ribosomal subunit interface protein | 5 | 5 | 21.1 | 14.92 | hpf; raiA | cytosolic |
| EGP65053.1 | 30S ribosomal protein S12 domain protein, partial | 7 | 3 | 6.2 | 14.54 |  | cytosolic |
| EGP67702.1 | flavin reductase | 5 | 4 | 22.6 | 14.4 | azr_1 | cytosolic |
| EGP69047.1 | ketol-acid reductoisomerase | 4 | 3 | 37.3 | 14.21 | ilvC | cytosolic |
| EGP69472.1 | YbbR-like protein | 5 | 3 | 27.9 | 13.76 |  | cytosolic |
| EGP67686.1 | NADH oxidase | 5 | 4 | 50.2 | 13.65 | noxE | cytosolic |
| EGP69504.1 | beta-ketoacyl-acyl-carrier-protein synthase II | 6 | 6 | 43.9 | 13.52 | fabF | cytosolic |
| EGP67588.1 | UMP kinase | 6 | 4 | 26.4 | 13.51 | pyrH | cytosolic |
| EGP67608.1 | RNA polymerase sigma factor RpoD | 5 | 4 | 42 | 13.37 | rpoD | cytosolic |
| EGP68182.1 | CTP synthase | 6 | 4 | 59.2 | 13.22 | pyrG | cytosolic |
| EGP69641.1 | phosphoglucosamine mutase | 6 | 4 | 48 | 12.99 | glmM | cytosolic |
| EGP70495.1 | adenylosuccinate synthase | 6 | 5 | 49.2 | 12.9 | purA | cytosolic |
| EGP69186.1 | ribosomal protein L13 | 5 | 2 | 16.1 | 12.9 | rplM | cytosolic |
| EGP70642.1 | acetate kinase | 6 | 6 | 43.3 | 12.85 | ackA | cytosolic |
| EGP69174.1 | phenylalanine--tRNA ligase, alpha subunit | 8 | 5 | 40.9 | 12.76 | pheS | cytosolic |
| EGP67568.1 | glycosyltransferase, group 1 family protein | 5 | 5 | 41.7 | 12.67 |  | cytosolic |
| EGP65251.1 | ribosomal protein L31 | 6 | 3 | 9.4 | 12.67 | rpmE | cytosolic |
| EGP70861.1 | UDP-glucose 4-epimerase | 7 | 6 | 37.4 | 12.6 | galE | cytosolic |
| EGP69795.1 | pur operon repressor PurR | 4 | 3 | 29.2 | 12.58 | purR | cytosolic |
| EGP69998.1 | cell cycle protein GpsB | 5 | 3 | 12.5 | 12.57 | gpsB | cytosolic |
| EGP70500.1 | fatty acid/phospholipid synthesis protein PlsX | 5 | 4 | 35 | 12.41 | plsX | cytosolic |
| EGP69928.1 | NAD(P)-specific glutamate dehydrogenase | 5 | 5 | 48.8 | 12.36 | gdhA | cytosolic |
| EGP69189.1 | putative fructose-6-phosphate aldolase | 5 | 5 | 24.3 | 12.25 |  | cytosolic |
| EGP69573.1 | ribosomal protein S6 | 4 | 3 | 11.1 | 12.04 | rpsF | cytosolic |
| EGP69651.1 | NAD+ synthetase | 4 | 4 | 30.2 | 11.7 | nadE | cytosolic |
| EGP69035.1 | ribosomal protein S19 | 4 | 3 | 10.7 | 11.69 | rpsS | cytosolic |
| EGP69121.1 | 30S ribosomal protein S13 | 5 | 5 | 13.4 | 11.59 | rpsM | cytosolic |
| EGP68923.1 | glutamine-fructose-6-phosphate transaminase (isomerizing) | 7 | 6 | 65.4 | 11.42 | glmS | cytosolic |
| EGP69156.1 | peptidoglycan DL-endopeptidase CwlO family protein | 4 | 3 | 43.8 | 11.33 |  | cytosolic |
| EGP70589.1 | 2,3,4,5-tetrahydropyridine-2,6-dicarboxylate N-acetyltransferase | 4 | 3 | 23.9 | 11.19 | dapD | cytosolic |
| EGP68965.1 | phenylalanine--tRNA ligase, beta subunit | 5 | 3 | 87 | 11.15 | pheT | cytosolic |
| EGP69122.1 | ribosomal protein S8 | 5 | 4 | 14.8 | 11.01 | rpsH | cytosolic |
| EGP69791.1 | alcohol dehydrogenase, propanol-preferring | 4 | 4 | 35.7 | 10.66 | adhA | cytosolic |
| EGP69744.1 | ribosomal protein S12-like protein, partial | 4 | 2 | 6.7 | 10.6 |  | cytosolic |
| EGP69763.1 | glutamine synthetase, type I | 3 | 2 | 50.3 | 10.51 | glnA | cytosolic |
| EGP69546.1 | protein phosphatase 2C | 6 | 4 | 26.9 | 10.1 |  | cytosolic |
| EGP68591.1 | dihydroxyacetone kinase, DhaK subunit | 3 | 3 | 35.2 | 10.05 | dhaK | cytosolic |
| EGP69524.1 | trigger factor | 17 | 11 | 47.4 | 9.93 | tig | cytosolic |
| EGP65439.1 | phosphate transport system regulatory protein PhoU | 4 | 4 | 25 | 9.92 | phoU | cytosolic |
| EGP69639.1 | matrixin | 5 | 3 | 26.7 | 9.91 |  | cytosolic |
| EGP70484.1 | arginine--tRNA ligase | 3 | 3 | 63.4 | 9.9 | argS | cytosolic |
| EGP69159.1 | ribosomal protein L24 | 3 | 3 | 11 | 9.85 | rplX | cytosolic |
| EGP68199.1 | chaperonin GroL | 5 | 4 | 57 | 9.61 | groEL | cytosolic |
| EGP68920.1 | ribosomal protein L18 | 5 | 3 | 12.9 | 9.57 | rplR | cytosolic |
| EGP65172.1 | beta-lactamase-like protein | 3 | 3 | 45.1 | 9.54 |  | cytosolic |
| EGP69100.1 | 50S ribosomal protein L4 | 3 | 2 | 22.2 | 9.52 | rplD | cytosolic |
| EGP70568.1 | histidine--tRNA ligase | 3 | 3 | 48.2 | 9.49 | hisS | cytosolic |
| EGP69176.1 | adenylate kinase | 3 | 3 | 23.7 | 9.09 | adk | cytosolic |
| EGP68174.1 | valine--tRNA ligase | 3 | 3 | 100.9 | 9.03 | valS | cytosolic |
| EGP70701.1 | putative spore coat polysaccharide biosynthesis protein SpsC | 4 | 4 | 45.5 | 9.02 |  | cytosolic |
| EGP68977.1 | ribosomal protein S10 | 6 | 4 | 11.6 | 8.81 | rpsJ | cytosolic |
| EGP65189.1 | LICD family protein | 3 | 3 | 31.9 | 8.48 |  | cytosolic |
| EGP69210.1 | pullulanase, extracellular | 4 | 4 | 144.5 | 8.45 |  | cytosolic |
| EGP69932.1 | ribosomal protein S16 | 3 | 2 | 10.2 | 8.3 | rpsP | cytosolic |
| EGP69494.1 | ribosomal protein S18 | 4 | 2 | 9.2 | 8.24 | rpsR | cytosolic |
| EGP69460.1 | guanylate kinase | 3 | 2 | 23.8 | 8.13 | gmk | cytosolic |
| EGP69673.1 | aspartyl/glutamyl-tRNA(Asn/Gln) amidotransferase, A subunit | 3 | 3 | 52.1 | 8.02 | gatA | cytosolic |
| EGP70538.1 | aspartate--tRNA ligase | 3 | 3 | 66.1 | 7.95 | aspS | cytosolic |
| EGP69734.1 | RmuC domain protein | 3 | 3 | 47.8 | 7.89 | rmuC | cytosolic |
| EGP69019.1 | ribosomal protein S9 | 5 | 2 | 14.2 | 7.86 | rpsI | cytosolic |
| EGP69172.1 | inosine-5'-monophosphate dehydrogenase | 3 | 2 | 52.5 | 7.81 | guaB | cytosolic |
| EGP67794.1 | glycine--tRNA ligase, alpha subunit | 3 | 3 | 34.9 | 7.66 | glyQ | cytosolic |
| EGP65328.1 | phosphate acetyltransferase | 5 | 5 | 35.1 | 7.49 | pta | cytosolic |
| EGP65315.1 | lysine--tRNA ligase | 6 | 4 | 56.6 | 7.48 | lysS | cytosolic |
| EGP69618.1 | excalibur domain protein | 4 | 3 | 26 | 7.17 |  | cytosolic |
| EGP68412.1 | hypothetical protein HMPREF9958_0340 | 3 | 2 | 11.2 | 7.1 |  | cytosolic |
| EGP65230.1 | glucose-1-phosphate adenylyltransferase | 5 | 3 | 41.5 | 7.01 | glgC | cytosolic |
| EGP68458.1 | phosphopentomutase | 3 | 3 | 44.1 | 6.95 | deoB | cytosolic |
| EGP69478.1 | translation elongation factor P | 4 | 4 | 20.6 | 6.93 | efp | cytosolic |
| EGP68611.1 | putative TPP-dependent acetoin dehydrogenase complex | 3 | 3 | 38.1 | 6.82 |  | cytosolic |
| EGP69154.1 | chaperone protein ClpB | 3 | 2 | 90.2 | 6.81 | clpB | cytosolic |
| EGP65450.1 | HPr(Ser) kinase/phosphatase | 2 | 2 | 34.8 | 6.69 | hprK | cytosolic |
| EGP70745.1 | putative N-acetylneuraminate lyase | 4 | 3 | 33.4 | 6.68 | nanA_4 | cytosolic |
| EGP68975.1 | ribosomal protein L15 | 4 | 2 | 15.4 | 6.58 | rplO | cytosolic |
| EGP70001.1 | UDP-galactopyranose mutase, partial | 3 | 3 | 37.8 | 6.46 | glf | cytosolic |
| EGP65212.1 | ribosomal protein L19 | 5 | 3 | 13.1 | 6.46 | rplS | cytosolic |
| EGP68453.1 | purine nucleoside phosphorylase | 2 | 2 | 26.2 | 6.39 | deoD | cytosolic |
| EGP68442.1 | negative regulator of genetic competence ClpC/MecB | 3 | 2 | 83.8 | 6.35 | clpC | cytosolic |
| EGP69463.1 | aspartyl/glutamyl-tRNA(Asn/Gln) amidotransferase, B subunit | 4 | 3 | 53.6 | 6.31 | gatB | cytosolic |
| EGP69514.1 | glucokinase | 3 | 2 | 33.5 | 6.26 | glk | cytosolic |
| EGP69513.1 | serine--tRNA ligase | 12 | 10 | 47.6 | 6.01 | serS | cytosolic |
| EGP69757.1 | pyruvate formate-lyase 1-activating enzyme | 3 | 3 | 30.1 | 5.84 | pflA | cytosolic |
| EGP65165.1 | ribosomal protein L21 | 3 | 2 | 11.2 | 5.82 | rplU | cytosolic |
| EGP69223.1 | tRNA uridine 5-carboxymethylaminomethyl modification enzyme GidA | 2 | 2 | 71 | 5.74 | mnmG | cytosolic |
| EGP69000.1 | GTP-binding protein YchF | 3 | 3 | 41.2 | 5.68 | ychF | cytosolic |
| EGP69502.1 | ABC transporter, substrate-binding protein, family 3 | 3 | 2 | 29.7 | 5.54 |  | cytosolic |
| EGP69105.1 | excinuclease ABC, A subunit | 4 | 4 | 104 | 5.53 | uvrA | cytosolic |
| EGP65274.1 | peptide chain release factor 1 | 2 | 2 | 40.6 | 5.53 | prfA | cytosolic |
| EGP69726.1 | glutamyl aminopeptidase | 2 | 2 | 37.9 | 5.52 | pepA | cytosolic |
| EGP67714.1 | transcriptional regulator, TetR family | 2 | 2 | 21.7 | 5.32 | ethR | cytosolic |
| EGP68446.1 | FeS assembly protein SufD | 2 | 2 | 46.2 | 5.3 | sufD | cytosolic |
| EGP65271.1 | aspartate-semialdehyde dehydrogenase | 4 | 4 | 38.8 | 5.08 | asd | cytosolic |
| EGP69774.1 | aminopeptidase C | 2 | 2 | 50.2 | 4.97 | pepC | cytosolic |
| EGP67715.1 | putative C protein alpha-antigen | 3 | 3 | 226.2 | 4.95 |  | cytosolic |
| EGP68425.1 | ribose 5-phosphate isomerase A | 2 | 2 | 24.9 | 4.82 | rpiA | cytosolic |
| EGP70636.1 | R3H domain protein | 3 | 2 | 37 | 4.81 |  | cytosolic |
| EGP69955.1 | membrane alanyl aminopeptidase | 2 | 2 | 95.4 | 4.78 | pepN | cytosolic |
| EGP68572.1 | DNA gyrase, A subunit | 4 | 4 | 92 | 4.77 | gyrA | cytosolic |
| EGP69915.1 | ATP cone domain protein | 4 | 4 | 12.9 | 4.77 | nrdD_2 | cytosolic |
| EGP70768.1 | isoleucine--tRNA ligase | 6 | 4 | 105.2 | 4.75 | ileS | cytosolic |
| EGP69450.1 | acetyl-CoA carboxylase, carboxyl transferase, alpha subunit | 2 | 2 | 28.1 | 4.74 | accA | cytosolic |
| EGP65201.1 | carbamoyl-phosphate synthase, large subunit | 3 | 3 | 116.2 | 4.61 | carB | cytosolic |
| EGP68569.1 | tagatose-bisphosphate aldolase | 2 | 2 | 36.3 | 4.54 | lacD | cytosolic |
| EGP65254.1 | glycogen/starch synthase, ADP-glucose type | 2 | 2 | 54.2 | 4.48 | glgA | cytosolic |
| EGP70628.1 | co-chaperone GrpE | 2 | 2 | 20 | 4.44 | grpE | cytosolic |
| EGP69458.1 | adenine phosphoribosyltransferase | 2 | 2 | 18.7 | 4.4 | apt | cytosolic |
| EGP69484.1 | ribosomal protein L11 | 2 | 2 | 14.8 | 4.35 | rplK | cytosolic |
| EGP65279.1 | glucose-6-phosphate dehydrogenase | 2 | 2 | 56.6 | 4.08 | zwf | cytosolic |
| EGP70681.1 | N-acetylglucosamine-6-phosphate deacetylase | 2 | 2 | 41.8 | 3.97 | nagA | cytosolic |
| EGP67795.1 | ATP-dependent Clp protease, proteolytic subunit ClpP | 2 | 2 | 21.3 | 3.92 | clpP | cytosolic |
| EGP70624.1 | chaperone protein DnaJ | 2 | 2 | 40.3 | 3.87 | dnaJ | cytosolic |
| EGP65295.1 | hypothetical protein HMPREF9958_1101 | 2 | 2 | 30.6 | 3.68 |  | cytosolic |
| EGP68757.1 | ATPase, AAA family | 2 | 2 | 77.5 | 3.55 |  | cytosolic |
| EGP69501.1 | FemAB family protein | 2 | 2 | 47.1 | 3.23 |  | cytosolic |
| EGP67816.1 | GMP synthase (glutamine-hydrolyzing), C-terminal domain protein | 3 | 3 | 57.5 | 2.81 | guaA | cytosolic |
| EGP68582.1 | ribonucleoside-diphosphate reductase, beta subunit | 2 | 2 | 36.9 | 2.77 |  | cytosolic |
| EGP69756.1 | UDP-N-acetylglucosamine 1-carboxyvinyltransferase | 2 | 2 | 46 | 2.22 | murA | cytosolic |
| EGP69582.1 | nicotinate phosphoribosyltransferase | 3 | 2 | 55 | 2.18 | pncB | cytosolic |
| EGP67580.1 | mannosyl-glycoprotein endo-beta-N-acetylglucosaminidase | 2 | 2 | 16.6 | 2.17 |  | cytosolic |
| EGP69566.1 | thiol-disulfide oxidoreductase ResA family protein | 2 | 2 | 20.8 | 2.15 |  | cytosolic |
| EGP69493.1 | HAD phosphatase, family IIIA | 2 | 2 | 20.1 | 2.05 |  | cytosolic |
| EGP70551.1 | DNA mismatch repair protein MutS | 2 | 2 | 94.7 | 2.04 | mutS | cytosolic |
| EGP70018.1 | dTDP-glucose 4,6-dehydratase | 2 | 2 | 39.2 | 1.89 | rfbB | cytosolic |
| EGP68984.1 | ribosomal protein S14p/S29e | 2 | 2 | 7.1 | 1.75 | rpsN | cytosolic |
| EGP69570.1 | polyribonucleotide nucleotidyltransferase | 2 | 2 | 80.9 | 1.71 | pnp | cytosolic |
| EGP69145.1 | ribosomal protein L14 | 2 | 2 | 13 | 1.68 | rplN | cytosolic |
| EGP70518.1 | glutamate--tRNA ligase | 2 | 2 | 56 | 0 | gltX | cytosolic |
| EGP67691.1 | phosphomannose isomerase type I | 2 | 2 | 35.2 | 0 | gmuF | cytosolic |
| EGP68967.1 | hypothetical protein HMPREF9958_1975 | 2 | 2 | 9.3 | 0 | rpoY | cytosolic |
| EGP69131.1 | putative lipoprotein | 41 | 17 | 54.7 | 113.79 |  | lipoproteins |
| EGP68936.1 | putative lipoprotein | 20 | 8 | 21 | 55.07 |  | lipoproteins |
| EGP65232.1 | hypothetical protein HMPREF9958_1216 | 5 | 3 | 11.7 | 13.65 | prsA | lipoproteins |
| EGP69113.1 | putative lipoprotein | 4 | 2 | 24.3 | 10.47 |  | lipoproteins |
| BCT97347.1 | discoidinolysin [Streptococcus mitis] | 65 | 29 | 75.9 | 193.91 |  | secreted proteins |
| EGP65326.1 | phosphopyruvate hydratase | 47 | 16 | 46.9 | 106.53 | eno | secreted proteins |
| EGP69081.1 | rod shape-determining protein MreC | 14 | 5 | 29.7 | 36.78 | mreC | secreted proteins |
| EGP67743.1 | receptor family ligand-binding region | 224 | 21 | 40.3 | 589.97 | livJ | transmembrane |
| EGP67619.1 | putative foldase protein PrsA | 95 | 25 | 34.2 | 292.26 | prsA | transmembrane |
| EGP70605.1 | NlpC/P60 family protein | 122 | 11 | 37.6 | 278.82 |  | transmembrane |
| EGP67763.1 | ATP synthase F1, beta subunit | 76 | 21 | 50.9 | 227.56 | atpD | transmembrane |
| EGP69942.1 | efflux transporter, RND family, MFP subunit | 87 | 20 | 41.9 | 227.15 |  | transmembrane |
| EGP67792.1 | PTS system fructose-specific EIIABC component | 89 | 14 | 67 | 217.37 | fruA | transmembrane |
| EGP68420.1 | basic membrane protein | 90 | 9 | 36.7 | 210.55 |  | transmembrane |
| EGP68423.1 | oligopeptide-binding protein SarA | 47 | 24 | 72.7 | 191.74 | sarA | transmembrane |
| EGP70531.1 | maltodextrin-binding protein MdxE | 84 | 17 | 45.3 | 189.98 | malX | transmembrane |
| EGP69752.1 | oligopeptide-binding protein AmiA | 63 | 28 | 72.6 | 164.91 | amiA | transmembrane |
| EGP69002.1 | cell division protease FtsH | 67 | 30 | 69.7 | 158.49 | hflB | transmembrane |
| EGP70778.1 | SecA cross-linking domain protein | 47 | 28 | 94.7 | 131.89 | secA | transmembrane |
| EGP67681.1 | oligopeptide-binding protein SarA | 68 | 21 | 72.7 | 120.7 | sarA | transmembrane |
| EGP69063.1 | ABC transporter, permease protein | 38 | 18 | 57.3 | 115.05 |  | transmembrane |
| EGP70000.1 | oligopeptide-binding protein SarA | 35 | 13 | 73 | 113.23 | sarA | transmembrane |
| EGP69095.1 | SPFH/Band 7/PHB domain protein | 47 | 18 | 33.5 | 109.71 | hflK | transmembrane |
| EGP69733.1 | putative PTS system mannose-specific EIIAB component | 42 | 14 | 35.4 | 106.13 | manX | transmembrane |
| EGP69532.1 | putative calcium-translocating P-type ATPase, PMCA-type | 39 | 19 | 96.9 | 105.61 |  | transmembrane |
| EGP67709.1 | ATP synthase F1, alpha subunit | 41 | 16 | 54.6 | 105.48 | atpA | transmembrane |
| EGP69042.1 | putative D-methionine-binding lipoprotein MetQ | 34 | 12 | 31.1 | 105.41 |  | transmembrane |
| EGP70824.1 | manganese ABC transporter substrate-binding lipoprotein | 33 | 9 | 34.5 | 96.86 | psaA | transmembrane |
| EGP69796.1 | mannose permease IID component | 34 | 12 | 33.8 | 93.29 | manZ | transmembrane |
| EGP69561.1 | TOBE domain protein | 44 | 17 | 41.8 | 91.14 |  | transmembrane |
| EGP69815.1 | oligopeptide-binding protein SarA | 46 | 24 | 73 | 91 | sarA | transmembrane |
| EGP65294.1 | ABC transporter, permease protein | 35 | 19 | 78.3 | 83.02 |  | transmembrane |
| EGP69789.1 | oligopeptide ABC transporter, ATP-binding protein OppF | 36 | 15 | 34.8 | 80.07 | amiF; | transmembrane |
| EGP69026.1 | serine protease do-like HtrA | 25 | 10 | 41.6 | 78.93 | htrA | transmembrane |
| EGP69750.1 | lon protease S16 C-terminal proteolytic domain protein | 25 | 13 | 37.5 | 76.67 | ylbL | transmembrane |
| EGP68411.1 | ABC transporter, ATP-binding protein | 31 | 16 | 55.3 | 76.53 |  | transmembrane |
| EGP69453.1 | oligopeptide-binding protein SarA | 18 | 9 | 72.5 | 76.42 | sarA | transmembrane |
| EGP69766.1 | oligopeptide ABC transporter, ATP-binding protein OppD | 28 | 13 | 39.5 | 72.48 | amiE | transmembrane |
| EGP69794.1 | ABC transporter, permease protein | 25 | 11 | 55.8 | 69.2 | oppB | transmembrane |
| EGP67801.1 | ABC transporter, substrate-binding protein, family 3 | 25 | 13 | 31.4 | 65.38 |  | transmembrane |
| EGP65430.1 | ABC transporter, substrate-binding protein, family 3 | 21 | 10 | 29.3 | 61.78 |  | transmembrane |
| EGP70669.1 | penicillin-binding protein, 1A family | 26 | 14 | 80.6 | 58.83 |  | transmembrane |
| EGP69519.1 | signal peptidase I | 30 | 8 | 23.5 | 58.4 | lepB | transmembrane |
| EGP67777.1 | ATP synthase F1, gamma subunit | 17 | 6 | 32.3 | 54.67 | atpG | transmembrane |
| EGP65213.1 | LemA family protein | 20 | 10 | 20.6 | 54.38 |  | transmembrane |
| EGP69778.1 | Na/Pi-cotransporter II-like protein | 23 | 13 | 59.3 | 53.24 |  | transmembrane |
| EGP69963.1 | septation ring formation regulator EzrA | 15 | 5 | 66.7 | 53.06 | ezrA | transmembrane |
| EGP69062.1 | polysaccharide biosynthesis protein | 20 | 12 | 69.2 | 52.9 |  | transmembrane |
| EGP69913.1 | ATPase/histidine kinase/DNA gyrase B/HSP90 domain protein | 19 | 9 | 46.3 | 50.57 |  | transmembrane |
| EGP69938.1 | putative bacteriocin ABC transporter | 20 | 8 | 25.7 | 50.23 |  | transmembrane |
| EGP68758.1 | hypothetical protein HMPREF9958_0312 | 20 | 11 | 49.8 | 50.11 |  | transmembrane |
| EGP67783.1 | high-affinity branched-chain amino acid ABC transporter, ATP-binding protein LivF | 17 | 10 | 25.7 | 49.55 | livF | transmembrane |
| EGP68762.1 | oligopeptide-binding protein SarA | 33 | 21 | 72.5 | 47.7 | sarA | transmembrane |
| EGP69671.1 | hypothetical protein HMPREF9958_1615 | 17 | 12 | 59.4 | 46.7 |  | transmembrane |
| EGP67796.1 | arginine ABC transporter, ATP-binding protein ArtM | 15 | 7 | 27.4 | 45.63 | artM | transmembrane |
| EGP69629.1 | glycosyltransferase family 28 N-terminal domain protein | 16 | 11 | 39.4 | 45.27 | murG | transmembrane |
| EGP65224.1 | arginine ABC transporter, ATP-binding protein ArtM | 15 | 5 | 27.3 | 43.92 | artM | transmembrane |
| EGP69543.1 | YmdA/YtgF family protein | 15 | 12 | 60.2 | 42.73 | rny | transmembrane |
| EGP67721.1 | PTS system IIBC component | 19 | 9 | 77.8 | 42.55 |  | transmembrane |
| EGP70807.1 | penicillin-binding protein, transpeptidase domain protein | 16 | 10 | 73.4 | 41.63 |  | transmembrane |
| EGP69929.1 | efflux ABC transporter, permease protein | 15 | 9 | 44.8 | 38.89 |  | transmembrane |
| EGP69730.1 | putative oligopeptide ABC transporter, permease protein OppC | 14 | 8 | 34.5 | 38.42 | amiD | transmembrane |
| EGP69031.1 | glutamine ABC transporter, ATP-binding protein GlnQ | 16 | 9 | 27.9 | 36.07 |  | transmembrane |
| EGP70492.1 | ABC transporter transmembrane region | 15 | 11 | 65.5 | 35.66 |  | transmembrane |
| EGP69182.1 | ATPase/histidine kinase/DNA gyrase B/HSP90 domain protein | 12 | 8 | 36.1 | 34.82 |  | transmembrane |
| EGP69764.1 | periplasmic-binding protein | 47 | 20 | 34.8 | 34.38 |  | transmembrane |
| EGP69797.1 | putative stage III sporulation protein J | 10 | 5 | 34 | 34.18 |  | transmembrane |
| EGP70756.1 | cell division protein FtsA | 17 | 11 | 49.8 | 31.5 | ftsA | transmembrane |
| EGP65484.1 | ABC transporter, ATP-binding protein | 12 | 6 | 27.6 | 31.26 |  | transmembrane |
| EGP65480.1 | glycerol phosphate lipoteichoic acid synthase 1 | 11 | 8 | 81.4 | 30.69 | ltaS | transmembrane |
| EGP67805.1 | polysaccharide deacetylase | 12 | 8 | 50.7 | 30.3 |  | transmembrane |
| EGP68774.1 | penicillin-binding protein 2X | 12 | 9 | 82.3 | 29.68 | pbpX | transmembrane |
| EGP67628.1 | ABC transporter, ATP-binding protein EcsA | 13 | 6 | 27.1 | 29.54 | ecsA | transmembrane |
| EGP65431.1 | hypothetical protein HMPREF9958_0756 | 11 | 8 | 48.8 | 28.84 |  | transmembrane |
| EGP70676.1 | preprotein translocase, YajC subunit | 9 | 3 | 11.2 | 28.4 | yajC | transmembrane |
| EGP68454.1 | branched-chain amino acid ABC transporter, permease protein | 12 | 5 | 37.4 | 28.04 | mglC | transmembrane |
| EGP68585.1 | PTS system, lactose-specific IIC component | 13 | 7 | 61 | 27.31 | lacE | transmembrane |
| EGP68587.1 | PTS system sugar-specific permease protein | 11 | 4 | 53 | 27.16 |  | transmembrane |
| EGP70777.1 | putative membrane protein | 8 | 4 | 38.4 | 26.53 |  | transmembrane |
| EGP65489.1 | polyamine ABC transporter, ATP-binding protein | 12 | 11 | 44.1 | 26.4 | potA | transmembrane |
| EGP70814.1 | putative membrane protein | 9 | 4 | 48.7 | 26.09 |  | transmembrane |
| EGP70799.1 | acyltransferase | 13 | 9 | 28.9 | 25.81 |  | transmembrane |
| EGP70010.1 | penicillin-binding protein 1A | 13 | 6 | 80.5 | 24.46 | pbpA | transmembrane |
| EGP69569.1 | YceG family protein | 12 | 5 | 64.2 | 24.02 | mltG | transmembrane |
| EGP69653.1 | PTS system sucrose-specific IIBC component | 4 | 4 | 65.4 | 23.93 |  | transmembrane |
| EGP67813.1 | efflux ABC transporter, permease protein | 9 | 6 | 34.2 | 23.61 |  | transmembrane |
| EGP69598.1 | rhodanese-like protein | 10 | 5 | 14.8 | 23.5 |  | transmembrane |
| EGP69052.1 | TOBE domain protein | 9 | 8 | 40.6 | 23.27 |  | transmembrane |
| EGP68434.1 | putative phosphate ABC transporter, ATP-binding protein | 10 | 4 | 26.9 | 22.85 |  | transmembrane |
| EGP70558.1 | ABC-type bacteriocin transporter | 11 | 10 | 80.1 | 22.33 |  | transmembrane |
| EGP70559.1 | ABC transporter transmembrane region | 9 | 6 | 62.5 | 22.27 |  | transmembrane |
| EGP70651.1 | ABC transporter, ATP-binding protein | 11 | 8 | 25.3 | 22.18 |  | transmembrane |
| EGP68952.1 | hypothetical protein HMPREF9958_2017 | 10 | 7 | 56.2 | 21.92 |  | transmembrane |
| EGP69012.1 | hypothetical protein HMPREF9958_1992 | 9 | 4 | 21.3 | 21.56 |  | transmembrane |
| EGP70464.1 | putative bacteriocin ABC transporter | 7 | 6 | 28.1 | 21.16 |  | transmembrane |
| EGP67735.1 | ATP synthase F0, B subunit | 8 | 5 | 17.8 | 21.14 | atpF | transmembrane |
| EGP67803.1 | ATP synthase F1, delta subunit | 10 | 5 | 20.6 | 21.01 | atpH | transmembrane |
| EGP70705.1 | bacterial sugar transferase | 11 | 6 | 26.7 | 20.14 |  | transmembrane |
| EGP67695.1 | cell division ATP-binding protein FtsE | 8 | 4 | 25.7 | 19.69 | ftsE | transmembrane |
| EGP69003.1 | hypothetical protein HMPREF9958_2064 | 7 | 2 | 30.3 | 19.21 |  | transmembrane |
| EGP68989.1 | ABC transporter, ATP-binding protein | 11 | 6 | 33.9 | 19.14 |  | transmembrane |
| EGP70544.1 | putative membrane protein | 6 | 4 | 51.4 | 18.82 |  | transmembrane |
| EGP68918.1 | ABC transporter, ATP-binding protein | 10 | 8 | 38.6 | 18.7 |  | transmembrane |
| EGP68777.1 | polysaccharide export protein, MPA1 family | 7 | 5 | 25.5 | 18.66 |  | transmembrane |
| EGP65227.1 | peptidase, M48 family | 9 | 3 | 33 | 18.59 | htpX | transmembrane |
| EGP67713.1 | glycerol facilitator-aquaporin | 5 | 2 | 31.8 | 18.22 | gla | transmembrane |
| EGP67685.1 | branched-chain amino acid ABC transporter, permease protein | 7 | 2 | 30.8 | 17.31 | livH | transmembrane |
| EGP69536.1 | glutamine ABC transporter, ATP-binding protein GlnQ | 12 | 6 | 28.1 | 16.79 |  | transmembrane |
| EGP69747.1 | hypothetical protein HMPREF9958_0854 | 7 | 5 | 9.2 | 16.54 |  | transmembrane |
| EGP69608.1 | TIGR00159 family protein | 6 | 4 | 34.3 | 15.79 | dacA | transmembrane |
| EGP69801.1 | mannose permease IIC component | 6 | 2 | 27.2 | 15.39 | manY | transmembrane |
| EGP69652.1 | hypothetical protein HMPREF9958_1610 | 5 | 4 | 18.3 | 14.59 |  | transmembrane |
| EGP69149.1 | putative manganese transport system ATP-binding protein MntA | 7 | 4 | 26.6 | 14.56 | adcC | transmembrane |
| EGP69989.1 | ABC transporter, quaternary amine uptake transporter family, substrate-binding protein | 6 | 5 | 55.5 | 14.17 |  | transmembrane |
| EGP68964.1 | RIP metalloprotease RseP | 10 | 7 | 45.7 | 14.02 | rseP | transmembrane |
| EGP65285.1 | periplasmic solute-binding family protein | 8 | 6 | 33.9 | 13.45 | adcAII | transmembrane |
| EGP69195.1 | hypothetical protein HMPREF9958_1940 | 5 | 2 | 25.4 | 13.29 |  | transmembrane |
| EGP70475.1 | transglycosylase | 5 | 2 | 89.1 | 12.98 |  | transmembrane |
| EGP67757.1 | putative membrane protein | 7 | 4 | 43.4 | 12.93 |  | transmembrane |
| EGP68779.1 | phospho-N-acetylmuramoyl-pentapeptide-transferase | 6 | 3 | 36 | 12.83 | mraY | transmembrane |
| EGP67722.1 | ATP synthase F1, epsilon subunit | 4 | 2 | 15.5 | 12.18 | atpC | transmembrane |
| EGP69178.1 | hypothetical protein HMPREF9958_1994 | 6 | 2 | 40.5 | 11.28 |  | transmembrane |
| EGP67724.1 | ABC transporter, permease protein | 4 | 3 | 28.9 | 11.22 |  | transmembrane |
| EGP65475.1 | phosphate ABC transporter, ATP-binding protein | 5 | 4 | 28.1 | 10.27 | pstB | transmembrane |
| EGP69023.1 | histidine kinase | 4 | 3 | 57.4 | 9.99 |  | transmembrane |
| EGP69585.1 | transporter, dicarboxylate/amino acid:cation Na+/H+ symporter family protein | 4 | 3 | 41.9 | 9.9 | sstT | transmembrane |
| EGP69132.1 | conserved domain protein | 4 | 4 | 70.6 | 9.75 |  | transmembrane |
| EGP67811.1 | stage III sporulation protein E | 4 | 4 | 85.8 | 9.59 | spoIIIE | transmembrane |
| EGP69061.1 | magnesium-importing ATPase | 5 | 5 | 98.1 | 9.1 | mgtA | transmembrane |
| EGP69551.1 | CorA-like protein | 3 | 2 | 34.8 | 9.061 |  | transmembrane |
| EGP65334.1 | glycosyl hydrolase family 25 | 3 | 2 | 30.2 | 8.88 |  | transmembrane |
| EGP69202.1 | ABC transporter transmembrane region | 7 | 4 | 65.4 | 8.81 |  | transmembrane |
| EGP70734.1 | putative manganese transport system ATP-binding protein MntA | 4 | 4 | 26.8 | 8.67 |  | transmembrane |
| EGP70452.1 | efflux ABC transporter, permease protein | 4 | 4 | 74.3 | 8.57 |  | transmembrane |
| EGP68988.1 | bacterial SH3 domain protein | 4 | 2 | 41.4 | 8.53 |  | transmembrane |
| EGP69754.1 | hypothetical protein HMPREF9958_0868 | 4 | 2 | 49.8 | 8.52 | corC | transmembrane |
| EGP70005.1 | hypothetical protein HMPREF9958_0288 | 4 | 3 | 51.8 | 8.22 | mapZ | transmembrane |
| EGP69092.1 | putative cardiolipin synthetase | 3 | 3 | 58.6 | 8.1 |  | transmembrane |
| EGP69565.1 | branched-chain amino acid transport system II carrier protein | 5 | 4 | 46.6 | 7.99 | brnQ | transmembrane |
| EGP70455.1 | VIT family protein | 2 | 2 | 24.7 | 7.68 |  | transmembrane |
| EGP65434.1 | phosphate binding protein | 3 | 3 | 32.6 | 7.61 | pstS | transmembrane |
| EGP70555.1 | hypothetical protein HMPREF9958_1251 | 3 | 2 | 10 | 7.61 | spr1912 | transmembrane |
| EGP69025.1 | DHHA1 domain protein | 4 | 3 | 70.4 | 7.51 |  | transmembrane |
| EGP65265.1 | hypothetical protein HMPREF9958_1170 | 4 | 2 | 13.7 | 7.19 | ybaN | transmembrane |
| EGP70722.1 | efflux ABC transporter, permease protein | 2 | 2 | 50 | 7.15 |  | transmembrane |
| EGP67765.1 | copper-exporting ATPase | 4 | 4 | 80.2 | 7.11 |  | transmembrane |
| EGP69552.1 | putative serine/threonine-protein kinase PrkC | 3 | 2 | 69.3 | 7.01 |  | transmembrane |
| EGP69951.1 | ABC transporter transmembrane region | 3 | 3 | 64.4 | 6.87 |  | transmembrane |
| EGP69777.1 | permease family protein | 2 | 2 | 51.4 | 6.71 |  | transmembrane |
| EGP68580.1 | chloride transporter, ClC family | 3 | 2 | 56.3 | 6.69 |  | transmembrane |
| EGP70856.1 | ABC transporter, permease protein | 3 | 3 | 33.6 | 6.68 |  | transmembrane |
| EGP69968.1 | hypothetical protein HMPREF9958_0271 | 2 | 2 | 16.9 | 6.66 |  | transmembrane |
| EGP68410.1 | FeS assembly ATPase SufC | 3 | 3 | 30.2 | 6.43 | sufC | transmembrane |
| EGP69135.1 | CorA-like protein | 2 | 2 | 36.4 | 6.36 | corA | transmembrane |
| EGP67575.1 | ABC transporter, ATP-binding protein | 4 | 4 | 28.3 | 6.14 |  | transmembrane |
| EGP69001.1 | TIGR02185 family protein | 4 | 2 | 20.1 | 6.06 |  | transmembrane |
| EGP69999.1 | histidine kinase | 2 | 2 | 37.8 | 5.96 |  | transmembrane |
| EGP69665.1 | D-serine/D-alanine/glycine transporter | 2 | 2 | 51.2 | 5.92 | cycA | transmembrane |
| EGP70480.1 | heavy metal translocating P-type ATPase | 2 | 2 | 77.3 | 5.86 |  | transmembrane |
| EGP69760.1 | ABC transporter substrate binding protein | 2 | 2 | 35.4 | 5.83 |  | transmembrane |
| EGP70800.1 | ABC transporter, ATP-binding protein | 2 | 2 | 25.8 | 5.83 |  | transmembrane |
| EGP68956.1 | cobalt transport protein | 2 | 2 | 29.5 | 5.54 | ecfT | transmembrane |
| EGP67620.1 | GTP-binding protein Era | 2 | 2 | 34 | 5.44 | era | transmembrane |
| EGP69972.1 | ABC transporter, ATP-binding protein | 2 | 2 | 27.1 | 5.43 | opuBA | transmembrane |
| EGP68933.1 | ATP-binding cassette cobalt transporter | 3 | 2 | 62.6 | 5.41 |  | transmembrane |
| EGP69004.1 | ABC transporter, ATP-binding protein | 3 | 2 | 60.8 | 5.25 | yheS_2 | transmembrane |
| EGP67741.1 | ABC transporter, substrate-binding protein, family 3 | 3 | 3 | 31.7 | 5.21 |  | transmembrane |
| EGP67817.1 | glutamine ABC transporter, ATP-binding protein GlnQ | 3 | 2 | 23.2 | 5.09 | glnQ_1 | transmembrane |
| EGP69507.1 | POTRA domain protein, FtsQ-type | 2 | 2 | 47.8 | 4.83 | divIB | transmembrane |
| EGP68994.1 | D-alanyl-lipoteichoic acid biosynthesis protein DltD | 2 | 2 | 49.3 | 4.79 | dltD | transmembrane |
| EGP65311.1 | membrane domain of glycerophosphoryl diester phosphodiesterase | 2 | 2 | 68.8 | 4.68 |  | transmembrane |
| EGP70649.1 | acyltransferase | 3 | 3 | 67.5 | 4.63 |  | transmembrane |
| EGP70776.1 | hypothetical protein HMPREF9958_1509 | 13 | 3 | 17.5 | 4.53 |  | transmembrane |
| EGP65321.1 | polysaccharide biosynthesis protein | 3 | 3 | 56.4 | 4.45 |  | transmembrane |
| EGP69969.1 | hypothetical protein HMPREF9958_0272 | 3 | 2 | 23.3 | 4.32 |  | transmembrane |
| EGP65219.1 | signal recognition particle-docking protein FtsY | 3 | 3 | 47.6 | 4.31 | ftsY | transmembrane |
| EGP68459.1 | putative arginine ABC transporter, permease protein ArtQ | 2 | 2 | 24.9 | 4.24 | artQ | transmembrane |
| EGP68782.1 | cell division protein FtsL | 2 | 2 | 12.2 | 4.23 | ftsL | transmembrane |
| EGP68608.1 | GTP-binding protein LepA | 3 | 3 | 67.6 | 3.99 | lepA | transmembrane |
| EGP70528.1 | PTS system sorbose-specific iic component | 2 | 2 | 31.9 | 3.94 |  | transmembrane |
| EGP69648.1 | cation diffusion facilitator family transporter | 3 | 3 | 44.4 | 3.75 |  | transmembrane |
| EGP67730.1 | branched-chain amino acid ABC transporter | 7 | 5 | 28.2 | 3.47 | lptB | transmembrane |
| EGP69144.1 | cobalt ATP-binding cassette C-terminal domain protein | 4 | 2 | 31.2 | 3.17 | cbiO | transmembrane |
| EGP69129.1 | putative membrane protein | 2 | 2 | 43.9 | 2.62 |  | transmembrane |
| EGP69203.1 | putative undecaprenyl-diphosphatase UppP | 2 | 2 | 31.8 | 2.58 | uppP | transmembrane |
| EGP68930.1 | preprotein translocase, SecY subunit | 8 | 6 | 47.4 | 2.56 | secY | transmembrane |
| EGP65468.1 | putative membrane protein | 2 | 2 | 61.7 | 2.31 |  | transmembrane |
| EGP70704.1 | TOBE domain protein | 2 | 2 | 37.4 | 2.12 |  | transmembrane |
| EGP70517.1 | ABC transporter, permease protein | 2 | 2 | 31.5 | 2.11 | malG | transmembrane |
| EGP65298.1 | hypothetical protein HMPREF9958_1073 | 39 | 18 | 35.3 | 100.13 |  |  |
| EGP65459.1 | hypothetical protein HMPREF9958_0708 | 3 | 2 | 14.6 | 8.96 |  |  |
| EGP65297.1 | hypothetical protein HMPREF9958_1214 | 3 | 3 | 18.5 | 5.05 |  |  |
| EGP65234.1 | hypothetical protein HMPREF9958_1109 | 2 | 2 | 16.9 | 4.75 |  |  |
| EGP68787.1 | hypothetical protein HMPREF9958_0315 | 4 | 4 | 54.9 | 4.6 |  |  |
| EGP69590.1 | hypothetical protein HMPREF9958_1625 | 2 | 2 | 14.4 | 4.41 |  |  |

Accession, Identification numbers assigned to the nucleotide sequences of genes published by the DNA Data Base of Japan. #PSM, Peptide-spectrum match. #peptides, Different types of detection peptides derived from the same protein. Score, Values obtained from mass spectrometry measurement results.
